# Isonitrile-Based Multicomponent Synthesis of β-Amino Boronic Acids as β-Lactamase Inhibitors

**DOI:** 10.3390/antibiotics9050249

**Published:** 2020-05-12

**Authors:** Emanuele Bassini, Stefano Gazzotti, Filomena Sannio, Leonardo Lo Presti, Jacopo Sgrignani, Jean-Denis Docquier, Giovanni Grazioso, Alessandra Silvani

**Affiliations:** 1 Dipartimento di Chimica, Università degli Studi di Milano, Via Golgi 19, 20133 Milan, Italy; emanuele.bassini@studenti.unimi.it (E.B.); stefano.gazzotti@unimi.it (S.G.); leonardo.lopresti@unimi.it (L.L.P.); 2 Dipartimento di Biotecnologie Mediche, Università degli Studi di Siena, Viale Bracci 16, 53100 Siena, Italy; filomena.sannio@unisi.it (F.S.); jddocquier@unisi.it (J.-D.D.); 3 Istituto di Ricerca in Biomedicina (IRB), Università della Svizzera Italiana (USI), Via V. Vela 6, CH-6500 Bellinzona, Switzerland; jacopo.sgrignani@irb.usi.ch; 4 Dipartimento di Scienze Farmaceutiche, Università degli Studi di Milano, Via L. Mangiagalli 25, 20133 Milan, Italy; giovanni.grazioso@unimi.it

**Keywords:** multicomponent reactions, β-amino boronic acids, β-lactamases, covalent docking

## Abstract

The application of various isonitrile-based multicomponent reactions to protected (2-oxoethyl)boronic acid (as the carbonyl component) is described. The Ugi reaction, both in the four components and in the four centers–three components versions, and the van Leusen reaction, proved effective at providing small libraries of MIDA-protected β-aminoboronic acids. The corresponding free β-aminoboronic acids, quantitatively recovered through basic mild deprotection, were found to be quite stable and were fully characterized, including by ^11^B-NMR spectroscopy. Single-crystal X-ray diffraction analysis, applied both to a MIDA-protected and a free β-aminoboronic acid derivative, provided evidence for different conformations in the solid-state. Finally, the antimicrobial activities of selected compounds were evaluated by measuring their minimal inhibitory concentration (MIC) values, and the binding mode of the most promising derivative on OXA-23 class D β-lactamase was predicted by a molecular modeling study.

## 1. Introduction

The recent interest in using boron in medicinal chemistry is due to the reliable fact that boron is a versatile atom, potentially not toxic, and thus able to play an important role in drug design [1].

The presence of an empty p-orbital promotes the easily conversion of the boron center from neutral trigonal planar sp^2^ to tetrahedral sp^3^ hybridization, imparting peculiar chemical behaviors to boron-containing compounds. Indeed, and thanks to the empty p-orbital, boronic acids can form tetracoordinate “ate” complexes by interacting with nucleophilic groups, including the side chains of some amino acids such as hydroxyl of serine and threonine. Further, tricoordinate boron can display several types of interaction with active site nucleophiles, leading to various and predictable coordination modes upon biological targets [2].

In the last few years, examples of new bioactive compounds containing boron have been widely reported, mainly in the anti-infective [3] and anticancer fields [4]. Some of them have been developed as drugs and recently received FDA approval, such as bortezomib, for the treatment of multiple myeloma, and vaborbactam, a potent inhibitor of β-lactamase (BL) enzymes, approved for use in combination with meropenem.

Thanks to their ability to form a tetrahedral adduct with the catalytic serine, boronic acid-containing compounds have been widely used in the design of β-lactamase transition state inhibitors [5,6]. Unlike commercially available β-lactam-based BL inhibitors (e.g., clavulanate, sulbactam, and tazobactam), which are effective primarily against class A BL, boronic acid-containing compounds have been demonstrated to strongly inhibit other classes of BLs, such as the clinically-relevant class B metallo-carbapenemases, class C enzymes, and OXA-type (class D) carbapenemases [7].

Since the rapid rise of antimicrobial resistance is currently one of the greatest medical challenges, the development of novel BL inhibitors is of utmost importance, particularly for the treatment of challenging infections caused by β-lactamase-producing Gram-negative species, which are more and more commonly resistant to carbapenems and exhibiting a multi-drug or even pan-drug resistance phenotype.

Looking at their chemical structures, most relevant boron-containing drugs, including bortezomib and vaborbactam, are characterized by the presence of a α-aminoboronic acid moiety. Such fragments mimic the naturally occurring amino acids and can act as their bioisosteres, acquiring huge potential as a privileged motif in peptidomimetic chemistry. A variety of valuable protocols have been established for the synthesis of α-aminoboronic acid derivatives [8], and for exploiting various boron-protecting groups aimed at controlling the high reactivity of the C-B bond and preventing its linkage [9].

Taking into account the pronounced tendency towards degradation of α-aminoboronic acids and aiming to expand the chemical diversity of aminoboronic acids-based compounds, Professor Yudin and coworkers recently explored the reactivity of boron-containing compounds, making use of the versatile *N*-methyliminodiacetyl(MIDA) boron-protecting group [10,11]. In particular, they provided access to novel boron and nitrogen-containing building blocks, developing the new class of β-aminoboronic acid derivatives, characterized by the presence of an additional C-atom between the boronic acid and amino moieties. Activity-based protein profiling of such homologues demonstrated their suitability as chemical probes of human enzymes, paving the way for their possible development as drugs [12,13].

Synthetic methods for preparation of both α and β-aminoboronic acids have been reviewed quite recently [14]. Said review attested to the rapid development in the field, with most of the protocols having been reported in the literature over the last decade. Even if major advances regarding the synthetic accessibility of such derivatives have been made, most of the reported strategies still rely on multi-step sequences, though there is a single example of a multicomponent strategy [15]. Owing to their ability to construct highly functionalized molecular scaffolds from simple precursors in a single step, multicomponent reactions (MCRs) can be considered excellent tools for diversity-oriented synthesis applications in the drug discovery field [16].

**Scheme 1:**
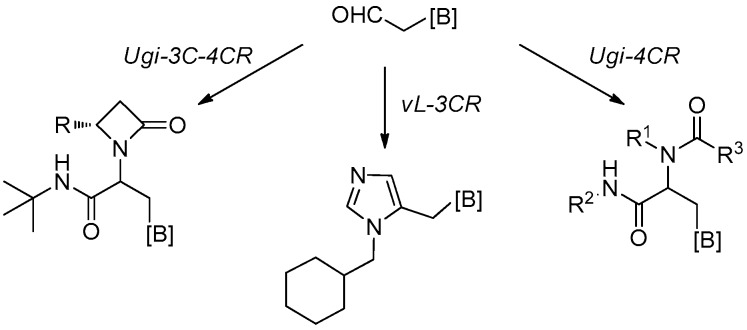
Three multicomponent strategies described in this work.

As part of our ongoing interest in isonitrile-based MCR applied to drug discovery projects [17,18,19,20,21,22,23], we herein report on the application of the Ugi reaction, both in the four components (4CR) and in the four centers-three components (4C–3CR) versions, for the rapid construction of two small libraries of β-aminoboronic acids. We also demonstrate the suitability of the isonitrile-based van Leusen reaction for the preparation of a MIDA-protected methylboronic acid, bearing a substituted imidazole ring (Scheme 1).

By means of simple and rapid protocols, 20 compounds have been prepared, showing a wide degree of appendage diversity, finely modulated with regard to stereoelectronic properties. A selection of such compounds was also tested for the ability to potentiate the activity of β-lactam antibiotics (a series of isolates produced various BL enzymes) to get early insights into and preliminarily information on their potential biological activities. Finally, the putative binding mode of the most promising compound on OXA-23 class D β-lactamase was predicted by a molecular modeling approach.

## 2. Results and Discussion

### 2.1. Synthesis

**Scheme 2:**
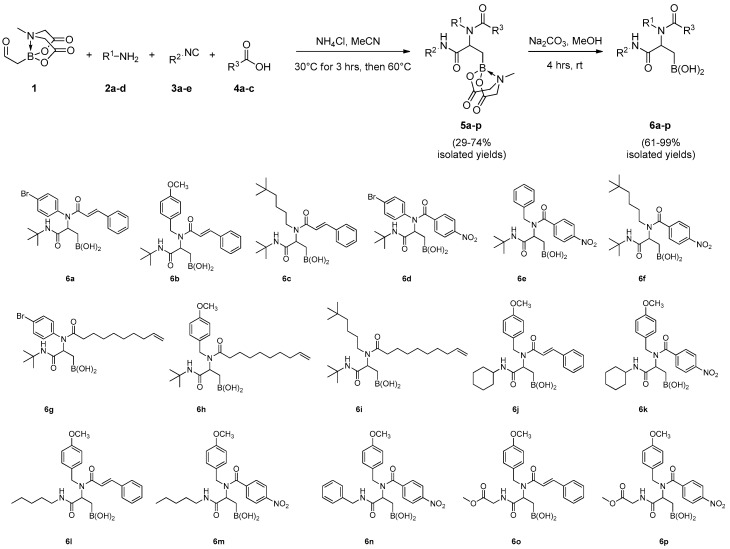
Scope of the Ugi-4CR*^aa^* synthesis of compounds **5a**–**p**: all reactions were performed using MIDA-protected α-boryl aldehyde **1** (0.6 mmol), amine **2** (0.7 mmol), carboxylic acid **4** (0.7 mmol), NH_4_Cl (1.0 mmol), and isocyanide **3** (1.0 mmol) in MeCN (6 mL), under heating (30–60 ^o^C) for 24 h. Synthesis of compounds **6a**–**p**: all reactions were performed using compounds **5a**–**p** (0.5 mmol) and solid Na_2_CO_3_ (9.0 mmol) in MeOH (7 mL), at room temperature, for 4 h.

We based our investigation on MIDA-protected α-boryl aldehyde **1**, employing p-Br-aniline **2a**, t-butyl isocyanide **3a** and *trans*-cinnamic acid **4a** for the initial screening of the Ugi-4CR conditions. Even though MCRs run best in methanol and trifluoroethanol, we looked at aprotic solvents, such as acetonitrile, dioxane, and toluene, in order to preserve the MIDA-protecting group. Acetonitrile proved to be the best choice, affording product **5a** in 46% yield, when the reaction was conducted at 60 °C, for 24 h. A curcumin-based TLC colorimetric method has been successfully employed for the qualitative detection of boron-containing derivatives, during the progress of the reaction [24]. A definite increase of the yield (69%) could be obtained by adding the isocyanide in portions over three h while keeping the reaction at room temperature, and then heating at 60 °C for the remaining time. In this way, the competitive Passerini reaction was completely discouraged. Finally, the addition of the weak Broensted acid ammonium chloride proved to be beneficial, likely through promotion of imine formation and activation to nucleophilic attack from isocyanide [25]. The optimized conditions which facilitated achieving a 74% satisfactory yield for **5a** are reported in Scheme 2. Under such conditions, sixteen MIDA-protected β-aminoboronic acid derivatives (**5a**–**p**) have been easily obtained, changing the amine (**2a**–**d**), isocyanide (**3a**–**e**), and carboxylic acid (**4a**–**c**) components.

Linear and branched alkyl isocyanides, and benzyl isocyanide and isocyano acetate, were successfully employed. Besides p-Br-aniline and trans-cinnamic acid, benzyl amines, lipophilic alkyl amines, benzoic acids, and long chain aliphatic acids proved to be suitable, respectively, as amine and carboxylic acid components, affording the desired MIDA-protected compounds **5a**–**p** in moderate, but satisfactory yields. In addition to HR-MS, ^1^H and ^13^C-NMR characterizations for all compounds, the reference compound **5a** was subjected to ^11^B-NMR and single-crystal X-ray diffraction.

**Figure 1:**
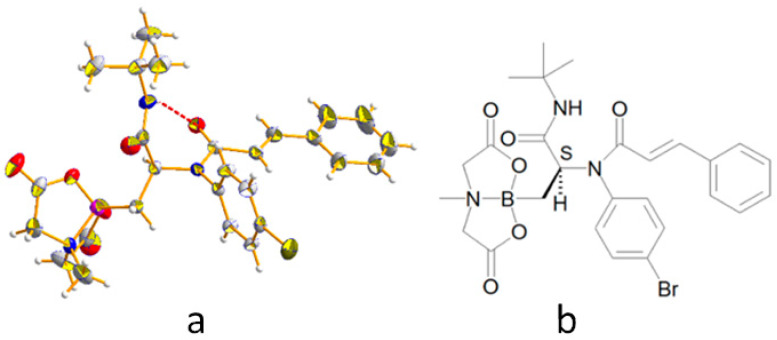
(**a**) Asymmetric unit of **5a**. Thermal ellipsoids at RT were drawn at the 30% probability level (C: gray; H: white; O: red; N: blue; B: purple; Br: dark green). The only relevant NH–O intramolecular hydrogen bond is highlighted as a red dashed line. (**b**) Molecular structure of the S-**5a** enantiomer (also the R enantiomer is present in the crystal in a 1:1 ratio) [26].

As shown in Figure 1, in the solid-state, compound **5a** lacks strong hydrogen bond donors, apart from the amide NH that is involved in an intramolecular contact with the tertiary amide carbonyl. Accordingly, the molecule assumes an extended conformation, with cumbersome phenyl rings mutually orthogonal to each other and the two five-membered rings almost perfectly flat.

In order to provide unprotected boronic acids for biological evaluation, reference compound **5a** was subjected to 3M HCl in acetonitrile, for removal of MIDA according to literature procedure. The corresponding β-aminoboronic acid **6a** could be isolated in a good yield after reverse-phase chromatographic purification, which allowed removing a small quantity of tertiary amide hydrolysis byproduct. Aiming at a cleaner protocol, also suitable for acid-labile compounds, an alternative, basic MIDA-deprotection was applied, treating compound **5a** with excess of sodium carbonate in dry methanol, at room temperature for four h. Compound **6a** was recovered quantitatively, without the need for purification. Said protocol was extended to all MIDA-protected compounds and afforded the desired β-aminoboronic acids **6** in excellent yields, also in the presence of susceptible methyl acetate groups (compounds **6o**–**p**).

**Figure 2:**
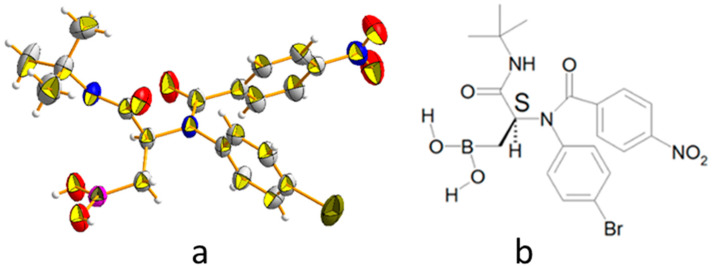
(**a**) Asymmetric unit of **6d**. Thermal ellipsoids at RT were drawn at the 30% probability level. The same atom color code as in Figure 1 was employed. (**b**) Molecular structure of the S–**6d** enantiomer (the R enantiomer is also present in the crystal in a 1:1 ratio) [28].

The free boronic acids **6a**–**p** proved to be quite stable towards the common protodeborylation reaction [27] and were fully characterized by means of HR-MS, ^1^H, ^13^C, and ^11^B-NMR. A single-crystal X-ray diffraction analysis was performed on **6d**, as reported in Figure 2. Unlike **5a**, the free β-aminoboronic acid **6d** contains strong hydrogen bond donors B-OH, which are all involved in extended ribbons networks in the solid state.

**Scheme 3:**
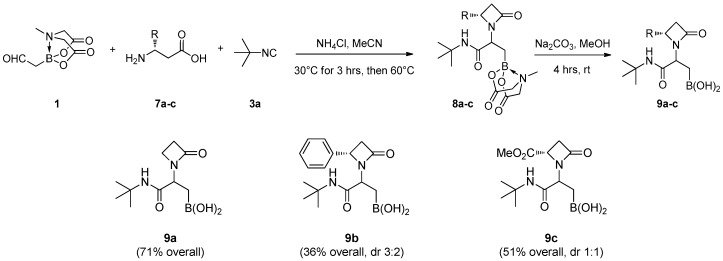
Scope of the Ugi-4C-3CR*^aa^* isolated overall yields over two steps are reported in parentheses. Diastereoisomeric ratios were determined by ^1^H-NMR on isolated products. Synthesis of compounds **8a**–**c**: all reactions were performed using MIDA-protected α-boryl aldehyde **1** (0.6 mmol), t-butyl isocyanide **3a** (1.0 mmol), and β-amino acids **7a**–**c** (0.7 mmol), in MeCN (6 mL), under heating (30-60 ^o^C) for 24 h. Synthesis of compounds **9a**–**c**: all reactions were performed using compounds **8a**–**c** (0.5 mmol) and solid Na_2_CO_3_ (9.0 mmol) in MeOH (7 mL), at room temperature, for 4 h.

Aiming to expand the diversity of peptidomimetic β-aminoboronic acids, including the privileged β-lactam ring in the chemical structure, a 4C–3CR version of the Ugi reaction was investigated (Scheme 3).

Keeping α-boryl aldehyde **1** and t-butyl isocyanide **3a** as fixed components, β-alanine **7a,** or alternatively, enantiomerically pure β-amino acids (*S*)-3-amino-3-phenylpropanoic acid **7b** or (*S*)-3-amino-4-methoxy-4-oxobutanoic acid **7c**, were employed in the reaction. The reaction conditions optimized for the Ugi-4CR proved effective for this three-component version also, affording compounds **8a**–**c** in acceptable yields. MIDA-deprotection with excess of sodium carbonate in dry methanol afforded, quantitatively, the corresponding β-lactam-based β-amino boronic acids **9a**–**c**. The chirality of β-amino acids **7b** and **7c** did not exert any asymmetric induction on the reaction pathway, so compounds **8b** and **8c** (and consequently **9b** and **9c**) were obtained as inseparable mixtures of enantiomerically pure β-lactams diastereoisomers and characterized as such. A unique signal at 31 ppm could be detected in the ^11^B-NMR spectrum, for both diastereoisomers of compounds **9b** and **9c**.

**Scheme 4:**
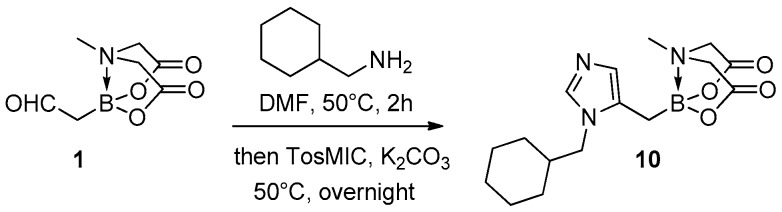
Van Leusen-3CR on aldehyde **1**.

Finally, the possible use of α-boryl aldehyde **1**, together with a primary amine and toluenesulfonylmethyl isocyanide (TosMIC) in a three-component reaction, was briefly explored. Cyclohexylmethanamine was chosen as the amine component and subjected to a precondensation time of two h at 50 °C in DMF with aldehyde **1**. After that, TosMIC and K_2_CO_3_ were added in two portions and the reaction was left at 50 °C overnight. The desired MIDA-protected (imidazolyl)methyl boronic acid **10** could be obtained, establishing the first application of the van Leusen reaction to the synthesis of boron-containing imidazoles (Scheme 4). Given the modest yield (32%) achieved by then, and after a brief screening of reaction conditions, MIDA-deprotection was not carried out, reserving it for further optimizations studies.

### 2.2. Biological Evaluation

Some of the synthesized compounds were subjected to a preliminary characterizations of their biological activities, in which their potential synergistic activities with β-lactam antibiotics were evaluated in a set bacterial strains producing various BLs, including both laboratory strains and clinical isolates (Table 1). As a result, and when tested at a fixed concentration of 16 µg/mL, no or limited potentiation of the antibiotic activity could be observed with BL-producing laboratory strains. Interestingly, compound **6e** showed a four-fold potentiation of the activity of ampicillin when tested on a strain producing the OXA-23 carbapenemase. A modest two-fold potentiation was also observed with some compounds (tested at 32 µg/mL) when tested on clinical isolates. These data overall indicate that the compounds might still lack sufficient inhibitory potency on their β-lactamase target or that they might not be able to accumulate at significant concentrations in the bacterial periplasm (due to, e.g., outer membrane impermeability or active efflux). However, these data are overall encouraging, and indicate that the present family of compounds, and future derivatives, should be further investigated.

### 2.3. Molecular Modeling Studies

Hypothesizing that compound **6e** could exert its antimicrobial activity by the covalent inhibition of the OXA-23 β-lactamase, computational studies were accomplished; we aimed to acquire atomistic details on the compound **6e**/target reciprocal interaction. This study could be useful for the rational design of new and more potent β-lactamase inhibitors.

**Figure 3:**
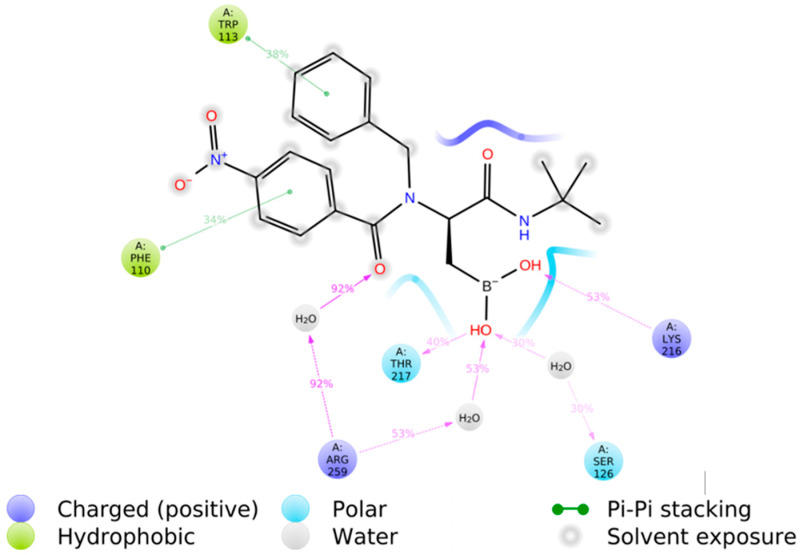
2D representation of the predicted binding mode of compound **6e** in the binding site of OXA-23 β-lactamase. Interactions that occurred for more than 30.0% of the simulation time in the selected trajectory (0.00 through 255.00 nsec), are shown. The picture was acquired by Maestro software.

The OXA-23 molecular model was created following the computational procedure reported in the Materials and Methods section. Since the racemate of **6e** was biologically evaluated, covalent docking of both enantiomers of compound **6e** was initially performed. Hypothesizing that our compounds could act as competitive ligands, the Oγ atom of the catalytic residue Ser79 of OXA-23 was used as an anchor point for the covalent docking of both enantiomers of **6e**; the CovDock algorithm, available on the Maestro modeling suite [29], was used for this calculation. The results suggested that the *(R)*-**6e** enantiomer could be the eutomer, due to a score 0.7 higher than the other enantiomer (−3.3 vs. −2.6, respectively). Then, further investigations were accomplished only on the *(R)***-6e**/Oxa-23 complex, by our undertaking of 250 ns of molecular dynamics (MD) simulations by means of the DESMOND algorithm [30]. By these simulations, the ligand/enzyme reciprocal adaptation was allowed, and finally, the “simulation interactions diagram” tool of Maestro permitted us to gain insights into the putative binding mode of compound *(R)*-**6e**, by processing the trajectory frames acquired during the MD simulation run. As shown in Figure 3, the OXA-23-Arg259, by a dual interaction, made polar contacts with the nitro-benzoyl carbonyl and boronic groups of compound *(R)*-**6e**. These bonds, stable over the majority of MD simulation frames, were mediated by water molecule bridges. Moreover, the OXA-23-Lys216 was hydrogen bound with the boronic group of *(R)*-**6e**, while the OXA-23-Trp113 and OXA-23-Phe110 interacted with the phenyl rings of *(R)*-**6e** by π–π (or Pi-Pi) stackings. Finally, the *N*-*t*-butyl group of *(R)*-**6e** was stabilized in a hydrophobic pocket sized by the Met221 and Trp219 residues of the β-lactamase.

**Figure 4:**
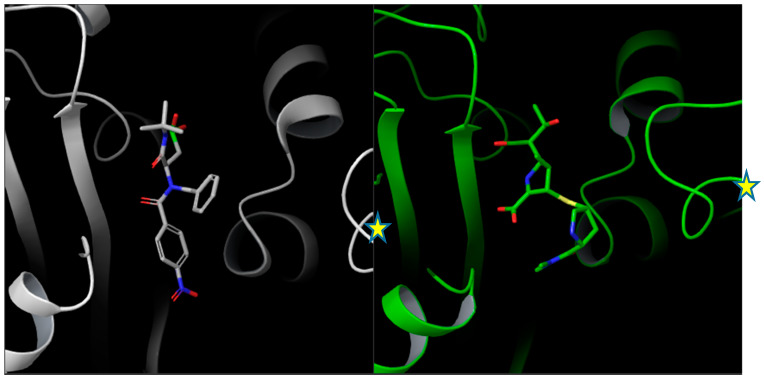
3D comparison between the predicted binding mode of compound **6e** (on the left) and the X-ray structure of the OXA-23/meropenem complex (on the right panel). The secondary structure of the enzyme is shown as a cartoon. The yellow stars highlight the position of Phe110. The 3D structures of the complexes were previously structurally aligned and superimposed; here they are displayed in two panels for clarity. The last frame of MD simulations of the **6e**/OXA-23 complex is represented on the left.

The comparison between the predicted binding mode of **6e** with the one found in the OXA-23/meropenem X-ray complex (see the Materials and Methods) suggested that the benzyl ring of the boronic compound should be substituted by a group with a lower size. In fact, during the MD simulations, a progressive opening of the catalytic crevice of the enzyme, for a repulsion between the benzyl ring and the side chain of OXA-23-Phe110, was observed (Figure 4). Moreover, a group substituting the *p*-nitro-phenyl ring, capable of interacting more efficiently with OXA-23-Arg259, could also ameliorate the affinity of the resulting compound.

## 3. Conclusions

In conclusion, structurally diverse, highly functionalized β-amino boronic acids have been efficiently synthesized via different multicomponent reactions. By means of the Ugi reaction, sixteen peptidomimetic β-amino boronic acids, functionalized with a variety of lipophilic and polar appendages, were prepared. The four centers-three components version of the Ugi reaction afforded three different β-lactam-based β-amino boronic acids, joining in a new scaffold two pharmacophoric moieties that are privileged in antibacterial drug discovery. Finally, the potential of the van Leusen reaction for the synthesis of imidazole-bearing methylboronic acids was demonstrated. The products were obtained in generally good yields, with simple workup procedures and straightforward isolation. They were fully characterized and a selection of them was subjected to biological evaluation, by determination of their in vitro antimicrobial susceptibility. By a molecular modelling investigation on the most active compound **6e**, a plausible binding mode in the binding site of β-lactamase OXA-23 was proposed and compared with the one found in the OXA-23/meropenem X-ray complex, leading to suggestions for further insights aimed at improving the biological activity.

## 4. Materials and Methods

### 4.1. General Methods

All commercial materials and solvents (> 95% purity grade) were purchased from Merck KGaA (Darmstadt, Germany) and used without further purification. All reactions were carried out under a nitrogen atmosphere, unless otherwise noted. All reactions were monitored by thin layer chromatography (TLC) on precoated silica gel 60 F254; spots were visualized with UV light, or by treatment with a 1% aqueous KMnO_4_ solution, or with a hydroalcoholic curcumin solution (100 mg of curcumin in a 100 mL solution of ethanol with 2 N HCl (99:1 *v*/*v*)). Products were purified by flash chromatography (FC) on silica gel 60 (230–400 mesh). Yields refer to isolated compounds estimated to be >95% pure as determined by ^1^H-NMR. NMR spectra were recorded on 300 or 400 MHz Bruker spectrometers, using tetramethylsilane (TMS) as the internal standard. Supplementary materials for ^13^C-NMR, the APT pulse sequence was adopted. Chemical shifts are reported in parts per million relative to the residual solvent. Multiplicities in ^1^H-NMR are reported as follows: s = singlet, d = doublet, t = triplet, m = multiplet, br s = broad singlet. High-resolution MS spectra (HR-MS) were recorded with a Thermo Fisher LCQ Fleet ion trap mass spectrometer, equipped with an ESI source. The purity of compound **6e** was confirmed to be >95% by means of elemental analysis on a CHN PerkinElmer 2400 instrument.

### 4.2. General Procedure for the Ugi-4CR (GP-A)

A test tube for the carousel was equipped with a magnetic stir bar and it was charged with compound **1** (120 mg, 0.6 mmol) and dry acetonitrile (6 mL). The primary amine **2a**–**d** (0.7 mmol), the carboxylic acid **4a**–**c** (0.7 mmol), and ammonium chloride (53 mg, 1.0 mmol) were added, and the reaction mixture was allowed to stir at 30 °C for 15 min. Then, the isocyanide **3a**–**e** (1.0 mmol) was added in portions over a period of three h, after that the reaction mixture was kept at 60 °C for 21 h. The reaction was monitored by TLC (DCM/MeOH, 95:5, curcumin-based colorimetric method). The reaction mixture was concentrated in vacuo; then ethyl acetate (5 mL) was added. The solution was washed with saturated NaHCO_3_ aq (×3), dried over Na_2_SO_4_, and concentrated under reduced pressure, to give a residue that was purified by a silica gravimetric chromatography column (eluent: DCM/MeOH = 99/1 to 96/4).

### 4.3. General Procedure for MIDA Deprotection (GP-B)

Under a nitrogen atmosphere, a round-bottom flask was equipped with a magnetic stir bar and charged with β-amino boronate **5a**–**p** or **8a**–**c** (0.5 mmol) and dry MeOH (7 mL). Solid Na_2_CO_3_ (9.0 mmol) was added. The resulting mixture was stirred for 4 h, and then the suspension was filtered and rinsed with a small amount of MeOH. The filtrate was concentrated in vacuo and the residue was partitioned between saturated aqueous NaHCO_3_ and ethyl acetate. The layers were separated, and the aqueous layer was additionally extracted with ethyl acetate. Combined ethyl acetate layers were dried over Na_2_SO_4_ and concentrated in vacuo.

### 4.4. General Procedure for the Ugi-4C-3CR (GP-C) 

A test tube for carousel was equipped with a magnetic stir bar and it was charged with compound **1** (100 mg, 0.5 mmol) and dry acetonitrile (5 mL). The β-amino acids **7a**–**c** (0.6 mmol) and ammonium chloride (53 mg, 1.0 mmol) were added and the reaction was allowed to stir at 30 °C for 15 min. Then, tert-butyl isocyanide **3a** (1.0 mmol) was added in portions over a period of three h; after that the reaction mixture was kept at 60 °C for 21 h. The reaction was monitored by TLC (DCM/MeOH, 95:5, curcumin-based colorimetric method). The reaction mixture was concentrated in vacuo and it was purified in a silica gravimetric chromatography column (eluent: DCM/MeOH = 99/1 to 95/5).

### 4.5. Procedure for the VL-3CR Reaction (GP-D)

A test tube for carousel was equipped with a magnetic stir bar, and it was charged with compound **1** (100 mg, 0.5 mmol) and dry DMF (5 mL). Cyclohexylmethanamine (0.08 mL, 0.6 mmol), was added and the resulting mixture was kept under stirring for 2 h at 50 °C under an atmosphere of nitrogen. Then, potassium carbonate (104 mg, 0.75 mmol) and TosMIC (148 mg, 0.75 mmol) were added in portions and the reaction was left overnight at 50 °C under stirring. The resulting mixture was concentrated in vacuo, and then it was partitioned between ethyl acetate/water. The aqueous phase was extracted with ethyl acetate (×3) and the organic phase was washed with brine (×5), dried over Na_2_SO_4_, and concentrated in vacuo, to give a residue that was purified by silica gravimetric chromatography column (eluent: DCM/MeOH = 99/1 to 90/10). The purified product was obtained with 32% yield as a brown solid.

*N-(4-bromophenyl)-N-(1-(tert-butylamino)-3-(4-methyl-2,6-dioxotetrahydro-2H-4λ^4^,8λ^4^-[1,3,2]oxazaborolo[2,3-b][1,3,2]oxazaborol-8-yl)-1-oxopropan-2-yl)cinnamamide (**5a**):* Prepared according to GP-A using α-borylaldehyde **1**, 4-bromoaniline **2a**, trans cinnamic acid **4a,** and tert-butyl isocyanide **3a**. Obtained as a white solid (yield = 74%); ^1^H-NMR (400 MHz, CD_3_OD) δ 7.68–7.52 (m, 3H), 7.35–7.25 (m, 7H), 6.22 (d, *J* = 15.6 Hz, 1H), 5.38–5.21 (m, 1H), 4.17 (d, *J* = 17.1 Hz, 1H), 4.13 (d, *J* = 16.8 Hz, 1H), 4.00 (d, *J* = 17.1 Hz, 1H), 3.96 (d, *J* = 16.8 Hz, 1H), 2.99 (s, 3H), 1.36 (s, 9H), 1.18 (d, *J* = 14.4 Hz, 1H), 0.96 (dd, *J* = 14.4, 4.4 Hz, 1H), (NH missed); ^13^C-NMR (101 MHz, CD_3_OD) δ 171.3, 170.0, 169.4, 167.6, 143.2, 138.9, 135.4, 132.9, 132.8, 132.7 (2C), 132.4, 130.4, 129.3 (2C), 128.2 (2C), 123.0, 119.2, 62.6, 62.5, 58.1, 51.6, 46.0, 28.2 (3C); HR-MS (ESI) *m*/*z*: [M + Na]^+^ calcd for C_27_H_31_BBrN_3_NaO_6_ 606.1387; found 606.1399.

*N-(1-(tert-Butylamino)-3-(4-methyl-2,6-dioxotetrahydro-2H-4λ^4^,8λ^4^-[1,3,2]oxazaborolo[2,3-b][1,3,2]oxazaborol-8-yl)-1-oxopropan-2-yl)-N-(4-methoxybenzyl)cinnamamide* (**5b**): Prepared according to GP-A using α-borylaldehyde **1**, 4-methoxybenzylamine **2b**, trans cinnamic acid **4a** and tert-butyl isocyanide **3a**. Obtained as a white solid (yield = 51%); ^1^H-NMR (400 MHz, 115 °C, DMSO-d6) δ 7.54 (m, 3H), 7.37 (d, *J* = 7.2 Hz, 2H), 7.25 (d, *J* = 8.1 Hz, 2H), 7.00 (d, *J* = 16.0 Hz, 1H), 6.87 (d, *J* = 8.1 Hz, 2H), 6.75 (s, 1H), 6.48 (d, *J* = 16.0 Hz, 1H), 4.88–4.81 (m, 2H), 4.54 (d, *J* = 16.2 Hz, 1H), 4.14 (d, *J* = 16.9 Hz, 1H), 4.10 (d, *J* = 16.8 Hz, 1H), 3.96 (d, *J* = 16.9 Hz, 1H), 3.94 (d, *J* = 16.8 Hz, 1H), 3.74 (s, 3H), 2.95 (s, 3H), 1.34–1.27 (m, 1H), 1.17 (s, 9H), 0.95–0.85 (m, 1H); ^13^C-NMR (101 MHz, 25 °C, DMSO-d6, 6:4 rotameric mixture) δ 170.4–166.4 (4C), 158.5, 144.6, 141.7 and 141.5 (1C), 135.9 and 135.5 (1C), 131.8, 129.3 and 129.2 (2C), 128.6, 128.4, 128.2 and 128.1 (1C), 120.0, 114.2 (2C), 62.3–62.0 (2C), 55.5, 55.4, 50.6 and 50.5 (1C), 46.8, 46.3 and 46.1 (1C), 29.4, 28.6 and 28.5 (1C); HR-MS (ESI) *m*/*z*: [M + Na]^+^ calcd for C_29_H_36_BN_3_NaO_7_ 572.2544; found 572.2524.

*N-(1-(tert-Butylamino)-3-(4-methyl-2,6-dioxotetrahydro-2H-4λ^4^,8λ^4^-[1,3,2]oxazaborolo[2,3-b][1,3,2]oxazaborol-8-yl)-1-oxopropan-2-yl)-N-(5,5-dimethylhexyl)cinnamamide* (**5c**): Prepared according to GP-A using α-borylaldehyde **1**, 5,5-dimethylhexan-1-amine **2c**, trans cinnamic acid **4a** and tert-butyl isocyanide **3a**. Obtained as a white solid (yield = 29%); ^1^H-NMR (400 MHz, CD_3_OD) δ 7.75 (d, *J* = 15.4 Hz, 1H), 7.71–7.61 (m, 5H), 7.06 (d, *J* = 15.4 Hz, 1H), 5.02 (m, *J* = 7.6 Hz, 0.7H), 4.50 (m, 0.3H), 4.17 (d, *J* = 17.1 Hz, 1H), 4.13 (d, *J* = 16.8 Hz, 1H), 4.02 (d, *J* = 17.1 Hz, 1H), 4.00 (d, *J* = 16.8 Hz, 1H), 3.67–3.53 (m, 2H), 3.08 (s, 2.1H), 3.01 (s, 0.9H), 1.73–1.58 (m, 4H), 1.45 (dd, *J* = 14.5, 8.8 Hz, 1H), 1.43–1.15 (m, 11H), 1.08 (dd, *J* = 14.5, 6.5 Hz, 1H), 0.90 (s, 9H), (NH missed); ^13^C-NMR (101 MHz, CD_3_OD) δ 171.5, 169.4, 168.8, 168.2, 142.7, 135.2, 130.2 and 129.6 (1C), 128.6 (2C), 128.0–127.6 (2C), 118.3, 61.8 (2C), 57.3, 55.9, 50.7, 45.3, 43.6, 31.4 (2C), 30.5, 28.4 (3C), 27.5 and 27.3 (3C), 21.9 (1C); HR-MS (ESI) *m*/*z*: [M + Na]^+^ calcd for C_29_H_44_BN_3_NaO_6_ 564.3221; found 564.3244.

*N-(4-Bromophenyl)-N-(1-(tert-butylamino)-3-(4-methyl-2,6-dioxotetrahydro-2H-4λ^4^,8λ^4^-[1,3,2]oxazaborolo[2,3-b][1,3,2]oxazaborol-8-yl)-1-oxopropan-2-yl)-4-nitrobenzamide* (**5d**): Prepared according to GP-A using α-borylaldehyde **1**, 4-bromoaniline **2a**, 4-nitrobenzoic acid **4b** and tert-butyl isocyanide **3a**. Obtained as a white solid (yield = 57%); ^1^H-NMR (400 MHz, CD_3_OD) δ 8.08 (d, *J* = 8.2 Hz, 2H), 7.58 (d, *J* = 8.2 Hz, 2H), 7.38 (d, *J* = 8.1 Hz, 2H), 7.21 (d, *J* = 8.1 Hz, 2H), 5.25 (dd, *J* = 7.0 Hz, 1H), 4.22 (d, *J* = 17.1 Hz, 1H), 4.18 (d, *J* = 16.8 Hz, 1H), 4.03 (d, *J* = 17.1 Hz, 1H), 4.01 (d, *J* = 16.8 Hz, 1H), 3.02 (s, 3H), 1.38 (s, 9H), 1.26 (dd, *J* = 7.0, 4.2 Hz, 2H), (NH missed); ^13^C-NMR (75 MHz, CD_3_OD, 2:1 rotameric mixture) δ 174.0–169.6 (4C), 149.7 and 149.6 (1C), 144.4 and 144.1 (1C), 140.9 and 140.2 (1C), 133.7–133.5 (4C), 130.6 (2C), 124.5 and 124.4 (2C), 123.5 and 123.3 (1C), 63.5, 63.4, 61.2 and 60.7 (1C), 58.7, 46.9, 29.2 (3C), (B-C_α_ missed); ^11^B-NMR (128 MHz, CD_3_CN) δ 12.26; HR-MS (ESI) *m*/*z*: [M + Na]^+^ calcd for C_25_H_28_BBrN_4_NaO_8_ 625.1081; found 625.1092.

*N-Benzyl-N-(1-(tert-butylamino)-3-(4-methyl-2,6-dioxotetrahydro-2H-4λ^4^,8λ^4^-[1,3,2]oxazaborolo[2,3-b][1,3,2]oxazaborol-8-yl)-1-oxopropan-2-yl)-4-nitrobenzamide* (**5e**): Prepared according to GP-A using α-borylaldehyde **1**, benzylamine **2d**, 4-nitrobenzoic acid **4b** and tert-butyl isocyanide **3a**. Obtained as a white solid (yield = 51%); ^1^H-NMR (400 MHz, CD_3_OD) δ 8.24 (d, *J* = 7.5 Hz, 2H), 7.75 (d, *J* = 7.5 Hz, 2H), 7.35–7.05 (m, 5H), 4.80 (d, *J* = 15.8 Hz, 1H), 4.71 (m, 1H), 4.48 (d, *J* = 15.8 Hz, 1H), 4.24 (d, *J* = 16.8 Hz, 1H), 4.22 (d, *J* = 16.4 Hz, 1H), 4.14–3.98 (m, 2H), 3.04 (s, 3H), 1.58 (d, *J* = 8.0 Hz, 1H), 1.35 (d, *J* = 8.0 Hz, 1H), 1.29 (s, 9H), (NH missed); ^13^C-NMR (101 MHz, CD_3_OD) δ 171.4–168.8 (4C), 148.2, 142.6, 137.0, 128.5 (2C), 128.2, 127.7 (2C), 127.4, 127.2, 123.3 (2C), 61.7, 61.5, 58.2, 57.3, 52.0, 45.1, 42.5, 27.4 (3C); ^11^B NMR (128 MHz, CD_3_CN) δ 12.33; HR-MS (ESI) *m*/*z*: [M + Na]^+^ calcd for C_26_H_31_BN_4_NaO_8_ 561.2133; found 561.2109.

*N-(1-(tert-Butylamino)-3-(4-methyl-2,6-dioxotetrahydro-2H-4λ^4^,8λ^4^-[1,3,2]oxazaborolo[2,3-b][1,3,2]oxazaborol-8-yl)-1-oxopropan-2-yl)-N-(5,5-dimethylhexyl)-4-nitrobenzamide* (**5f**): Prepared according to GP-A using α-borylaldehyde **1**, 5,5-dimethylhexan-1-amine **2c**, 4-nitrobenzoic acid **4b** and tert-butyl isocyanide **3a**. Obtained as a white solid (yield = 40%); ^1^H-NMR (400 MHz, CD_3_OD) δ 8.36 (d, *J* = 8.5 Hz, 2H), 7.83 (d, *J* = 8.5 Hz, 2H), 4.74 (dd, *J* = 9.6, 5.9 Hz, 1H), 4.21 (d, *J* = 17.1 Hz, 1H), 4.19 (d, *J* = 16.9 Hz, 1H), 4.05 (d, *J* = 17.1 Hz, 1H), 4.03 (d, *J* = 16.9 Hz, 1H), 3.42–3.21 (m, 2H), 3.07 (s, 3H), 1.53 (dd, *J* = 14.0, 9.6 Hz, 1H), 1.48–1.21 (m, 12H), 0.92 (m, 4H), 0.78 (s, 9H), (NH missed); ^13^C-NMR (101 MHz, CD_3_OD) δ 171.8, 171.3, 169.4, 168.9, 148.4, 142.8, 127.8 (2C), 123.5 (2C), 61.7, 61.6, 58.1, 57.1, 50.8, 45.2 (2C), 43.1, 30.0, 28.2 (3C), 27.5 (3C), 21.5 (2C); HR-MS (ESI) *m*/*z*: [M + Na]^+^ calcd for C_27_H_41_BN_4_NaO_8_ 583.2915; found 583.2931.

*N-(4-Bromophenyl)-N-(1-(tert-butylamino)-3-(4-methyl-2,6-dioxotetrahydro-2H-4λ^4^,8λ^4^-[1,3,2]oxazaborolo[2,3-b][1,3,2]oxazaborol-8-yl)-1-oxopropan-2-yl)dec-9-enamide* (**5g**): Prepared according to GP-A using α-borylaldehyde **1**, 4-bromoaniline **2a**, 9-decenoic acid **4c** and tert-butyl isocyanide **3a**. Obtained as a white solid (yield = 50%); ^1^H-NMR (400 MHz, CD_3_OD) δ 7.62 (d, *J* = 8.3 Hz, 2H), 7.23 (d, *J* = 8.3 Hz, 2H), 5.88–5.73 (m, *J* = 13.0 Hz, 1H), 5.20 (m, 1H), 4.95 (dd, *J* =13.0 Hz, 2H), 4.14–4.12 (m, 1H), 4.08 (d, *J* = 16.7 Hz, 1H), 3.97 (d, *J* = 17.6 Hz, 1H), 3.92 (d, *J* = 16.7 Hz, 1H), 2.95 (s, 3H), 2.34–2.26 (m, 1H), 2.09–1.97 (m, 4H), 1.65–1.48 (m, 3H), 1.36 (s, 9H), 1.27–1.13 (m, 6H), 1.04 (dd, *J* = 14.4, 10.5 Hz, 1H), 0.84 (dd, *J* = 14.4, 4.8 Hz, 1H), (NH missed); ^13^C-NMR (75 MHz, CD_3_OD) δ 176.1, 172.7, 171.0, 170.3, 140.4, 140.0, 133.9, 133.7, 133.5, 133.2, 123.9, 114.9, 63.6, 63.4, 58.79, 58.6, 46.9, 36.1 (2C), 35.1, 30.4 (3C), 29.2 (3C), 26.6 (2C); HR-MS (ESI) *m*/*z*: [M + Na]^+^ calcd for C_28_H_41_BBrN_3_NaO_6_ 628.2169; found 628.2154.

*N-(1-(tert-Butylamino)-3-(4-methyl-2,6-dioxotetrahydro-2H-4λ^4^,8λ^4^-[1,3,2]oxazaborolo[2,3-b][1,3,2]oxazaborol-8-yl)-1-oxopropan-2-yl)-N-(4-methoxybenzyl)dec-9-enamide* (**5h**): Prepared according to GP-A using α-borylaldehyde **1**, 4-methoxybenzylamine **2b**, 9-decenoic acid **4c** and tert-butyl isocyanide **3a**. Obtained as a white solid (yield = 61%); ^1^H-NMR (400 MHz, 115 °C, DMSO-d6) δ 7.18 (d, *J* = 8.1 Hz, 2H), 6.85 (d, *J* = 8.1 Hz, 2H), 6.69 (s, 1H), 5.90–5.72 (m, 1H), 4.97 (dd, *J* = 14.4 Hz, 2H), 4.74–4.55 (m, 2H), 4.39 (d, *J* = 16.4 Hz, 1H), 4.16–4.03 (m, 2H), 4.00–3.88 (m, 2H), 3.75 (s, 3H), 2.93 (s, 3H), 2.40–2.17 (m, 2H), 2.09–1.95 (m, 2H), 1.54 (m, 2H), 1.44–1.23 (m, 8H), 1.18 (s, 9H), 0.93–0.81 (m, 2H); ^13^C-NMR (101 MHz, 25 °C, DMSO-d6, 1:1 rotameric mixture) δ 174.6–169.4 (4C), 159.2 and 158.8 (1C), 139.9, 133.0 and 132.0 (1C), 129.4, 128.6, 115.7, 114.8, 114.2, 63.0 and 62.6 (2C), 58.4 (0.5C), 56.2, 55.8 (0.5C), 51.3 and 51.1 (1C), 47.8, 46.9 and 46.8 (1C), 34.2 and 33.5 (2C), 30.1 and 29.5 (4C), 29.2 (3C), 25.9 and 25.7 (2C); HR-MS (ESI) *m*/*z*: [M + Na]^+^ calcd for C_30_H_46_BN_3_NaO_7_ 594.3327; found 594.3320.

*N-(1-(tert-Butylamino)-3-(4-methyl-2,6-dioxotetrahydro-2H-4λ^4^,8λ^4^-[1,3,2]oxazaborolo[2,3-b][1,3,2]oxazaborol-8-yl)-1-oxopropan-2-yl)-N-(5,5-dimethylhexyl)dec-9-enamide* (**5i**): Prepared according to GP-A using α-borylaldehyde **1**, 5,5-dimethylhexan-1-amine **2c**, 9-decenoic acid **4c** and tert-butyl isocyanide **3a**. Obtained as a white solid (yield = 38%); ^1^H-NMR (400 MHz, DMSO-d6, 6:4 rotameric mixture) δ 7.48 (s, 0.4H), 7.04 (s, 0.6H), 5.92–5.68 (m, 1H), 4.96 (dd, *J* =14.2 Hz, 2H), 4.74 (t, *J* = 7.1 Hz, 0.6H), 4.28 (dd, *J* = 8.6, 5.0 Hz, 0.4H), 4.21–4.06 (m, 2H), 4.04–3.87 (m, 2H), 3.29–3.19 (m, 1.2H), 3.17–3.04 (m, 0.8H), 2.96 (s, 1.8H), 2.90 (s, 1.2H), 2.33–2.22 (m, 2H), 2.03–1.93 (m, 2H), 1.59–1.39 (m, 4H), 1.37–1.32 (m, 2.4H), 1.30–1.07 (m, 19.8H), 0.85 (s, 5.5H), 0.84 (s, 3.5H), 0.80 (d, *J* = 7.1 Hz, 0.3H), 0.67 (dd, *J* = 14.4, 5.0 Hz, 0.5H); ^13^C-NMR (101 MHz, DMSO-d6, 6:4 rotameric mixture) δ 174.0, 172.8 and 172.1 (1C), 170.9, 170.0 and 169.6 (1C), 139.9, 115.7, 62.8–62.5(2C), 57.9 and 55.7 (1C), 51.4 and 51.0 (2C), 46.8 and 46.7 (1C), 44.7 and 44.5 (1C), 34.3- 31.2 (6C), 30.3 (3C), 29.4 (3C), 26.1 and 25.9 (2C), 23.2 and 22.9 (2C); ^11^B-NMR (128 MHz, CD_3_CN) δ 12.56; HR-MS (ESI) *m*/*z*: [M + Na]^+^ calcd for C_30_H_54_BN_3_NaO_6_ 586.4003; found 586.4024.

*N-(1-(Cyclohexylamino)-3-(4-methyl-2,6-dioxotetrahydro-2H-4λ^4^,8λ^4^-[1,3,2]oxazaborolo[2,3-b][1,3,2]oxazaborol-8-yl)-1-oxopropan-2-yl)-N-(4-methoxybenzyl)cinnamamide* (**5j**): Prepared according to GP-A using α-borylaldehyde **1**, 4-methoxybenzylamine **2b**, trans cinnamic acid **4a** and cyclohexyl isocyanide **3b**. Obtained as a white solid (yield = 49%); ^1^H-NMR (400 MHz, CD_3_OD) δ 7.67 (d, *J* = 6.9 Hz, 1H), 7.61 (d, *J* = 15.5 Hz, 1H), 7.46 (d, *J* = 3.6 Hz, 1H), 7.40 (t, *J* = 3.6, 6.9 Hz, 2H), 7.35 (d, *J* = 3.6 Hz, 1H), 7.24 (d, *J* = 8.6 Hz, 2H), 6.95 (d, *J* = 15.5 Hz, 1H), 6.90 (d, *J* = 8.6 Hz, 2H), 5.13–5.05 (m, 1H), 4.95 (d, *J* = 17.1 Hz, 1H), 4.74 (d, *J* = 17.1 Hz, 1H), 4.17 (d, *J* = 17.1 Hz, 1H), 4.13 (d, *J* = 17.3 Hz, 1H), 3.99 (d, *J* = 17.1 Hz, 1H), 3.97 (d, *J* = 17.3 Hz, 1H), 3.77 (s, 3H), 3.52–3.44 (m, 1H), 3.03 (s, 2H), 2.98 (s, 1H), 1.89–1.80 (m 1H), 1.79–1.73 (m, 1H), 1.73–1.64 (m, 2H), 1.62–1.52 (m, 2H), 1.47–1.20 (m, 5H), 1.16–1.09 (m, 1H), (NH missed); ^13^C-NMR (75 MHz, CD_3_OD) δ 172.4–169.7 (4C), 160.8, 144.6, 136.7, 131.91, 131.3, 130.2 (2C), 129.5 (2C), 129.3 (2C), 120.2, 115.5 (2C), 63.5 (2C), 57.5, 56.1, 52.3, 47.0, 43.3, 33.7 (2C), 31.0, 26.9, 26.2 (2C); HR-MS (ESI) *m*/*z*: [M + Na]^+^ calcd for C_31_H_38_BN_3_NaO_7_ 598.2701; found 598.2689.

*N-(1-(Cyclohexylamino)-3-(4-methyl-2,6-dioxotetrahydro-2H-4λ^4^,8λ^4^-[1,3,2]oxazaborolo[2,3-b][1,3,2]oxazaborol-8-yl)-1-oxopropan-2-yl)-N-(4-methoxybenzyl)-4-nitrobenzamide* (**5k**): Prepared according to GP-A using α-borylaldehyde **1**, 4-methoxybenzylamine **2b**, 4-nitrobenzoic acid **4b,** and cyclohexyl isocyanide **3b**. Obtained as a white solid (yield = 53%); ^1^H-NMR (400 MHz, CD_3_OD, 6:4 rotameric mixture) δ 8.27 (d, *J* = 8.1 Hz, 2H), 7.77 (d, *J* = 8.1 Hz, 2H), 7.04 (d, *J* = 7.9 Hz, 2H), 6.82 (d, *J* = 7.9 Hz, 2H), 4.74–4.62 (m, 1.4H), 4.57 (s, 0.2H), 4.42 (d, *J* = 15.5 Hz, 0.6H), 4.25 (d, *J* = 17.1 Hz, 1H), 4.23 (d, *J* = 16.7 Hz, 1H), 4.13 (d, *J* = 17.1 Hz, 1H), 4.11 (d, *J* = 16.7 Hz, 1H), 4.06 (m, 0.8H), 3.77 (s, 3H), 3.64–3.47 (m, 1H), 3.04 (s, 3H), 1.87–1.55 (m, 6H), 1.46–1.22 (m, 6H), (NH missed); ^13^C-NMR (101 MHz, CD_3_OD) δ 170.9–168.0 (4C), 159.5, 148.3, 148.2, 129.2 (2C), 128.6, 127.8 (2C), 123.4 (2C), 113.9 (2C), 61.7, 61.6, 57.4, 54.4, 51.7, 45.2, 41.7, 32.1 (2C), 29.3, 25.2 (2C), 24.5; HR-MS (ESI) *m*/*z*: [M + Na]^+^ calcd for C_29_H_35_BN_4_NaO_9_ 617.2395; found 617.2382.

*N-(4-Methoxybenzyl)-N-(3-(4-methyl-2,6-dioxotetrahydro-2H-4λ^4^,8λ^4^-[1,3,2]oxazaborolo[2,3-b][1,3,2]oxazaborol-8-yl)-1-oxo-1-(pentylamino)propan-2-yl)cinnamamide* (**5l**): Prepared according to GP-A using α-borylaldehyde **2**, 4-methoxybenzylamine **3b**, trans cinnamic acid **4a, and** 1-pentyl isocyanide **5e**. Obtained as a white solid (yield = 40%); ^1^H-NMR (400 MHz, CD_3_OD, rotameric mixture) δ 7.80–7.51 (m, 2H), 7.49–7.31 (m, 4H), 7.29–7.21 (m, 2H), 7.13–6.79 (m, 3H), 5.14 (m, 0.5H), 5.00–4.91 (m, 0.6H), 4.71 (m, 1.9H), 4.18 (d, *J* = 16.8 Hz, 1H), 4.14 (d, *J* = 16.0 Hz, 1H), 4.00 (d, *J* = 16.8 Hz, 1H), 3.95–3.90 (m, 1H), 3.81–3.73 (m, 3H), 3.24–3.15 (m, 0.5H), 3.14–2.78 (m, 4.5H), 1.76–1.05 (m, 7H), 1.07–0.73 (m, 4H), (NH missed); ^13^C-NMR (101 MHz, CD_3_OD, rotameric mixture) δ 172.4–168.6 (4C), 159.7, 144.9 and 143.5 (1C), 135.9 and 135.7 (1C), 131.2 and 130.8 (1C) 130.7–129.7 (2C), 129.3 and 129.2 (2C), 128.5 and 128.4 (2C), 128.2, 119.2, and 117.8 (1C), 114.6 and 114.4 (2C), 62.5, 62.5, 56.3, 55.0, 51.3, 46.0, 40.7 and 39.9 (1C), 29.5, 29.1 (2C), 22.7, 13.6; HR-MS (ESI) *m*/*z*: [M + Na]^+^ calcd for C_30_H_38_BN_3_NaO_7_ 586.2701; found 586.2719.

*N-(4-Methoxybenzyl)-N-(3-(4-methyl-2,6-dioxotetrahydro-2H-4λ^4^,8λ^4^-[1,3,2]oxazaborolo[2,3-b][1,3,2]oxazaborol-8-yl)-1-oxo-1-(pentylamino)propan-2-yl)-4-nitrobenzamide* (**5m**): Prepared according to GP-A using α-borylaldehyde **1**, 4-methoxybenzylamine **2b**, 4-nitrobenzoic acid **4b,** and 1-pentyl isocyanide **3c**. Obtained as a white solid (yield = 53%); ^1^H-NMR (400 MHz, 115 °C, DMSO-d6, 9:1 rotameric mixture, only the major rotamer reported here) δ 8.17 (d, *J* = 7.8 Hz, 2H), 7.66 (d, *J* = 7.8 Hz, 2H), 7.24 (s, 1H), 7.11 (s, 2H), 6.78 (d, *J* = 7.8 Hz, 2H), 4.66–4.51 (m, 2H), 4.46 (d, *J* = 15.6 Hz, 1H), 4.15 (d, *J* = 15.3 Hz, 1H), 4.12 (d, *J* = 15.6 Hz, 1H), 3.97 (d, *J* = 15.3 Hz, 1H), 3.93 (d, *J* = 15.6 Hz, 1H), 3.73 (s, 3H), 3.01 (d, *J* = 6.0 Hz, 2H), 2.91 (s, 3H), 1.47–1.21 (m, 8H), 0.89 (t, *J* = 6.0 Hz, 3H); ^13^C-NMR (101 MHz, 25 °C, DMSO-d6, 7:3 rotameric mixture) δ 170.9–168.8 (4C), 158.6 and 158.3 (1C), 148.0 and 147.7 (1C), 144.3 and 143.7 (1C), 131.2 and 129.9 (1C), 129.1 (2C), 128.8, 128.0, 124.0, 123.7, 113.9, 113.7, 62.1 (2C), 55.5, 55.4, 49.9, 46.2, 39.3, 29.4, 29.1 (2C), 22.3, 14.4; HR-MS (ESI) *m*/*z*: [M + Na]^+^ calcd for C_28_H_35_BN_4_NaO_9_ 605.2395; found 605.2380.

*N-(1-(Benzylamino)-3-(4-methyl-2,6-dioxotetrahydro-2H-4λ^4^,8λ^4^-[1,3,2]oxazaborolo[2,3-b][1,3,2]oxazaborol-8-yl)-1-oxopropan-2-yl)-N-(4-methoxybenzyl)-4-nitrobenzamide* (**5n**): Prepared according to GP-A using α-borylaldehyde **1**, 4-methoxybenzylamine **2b**, 4-nitrobenzoic acid **4b, and** benzyl isocyanide **3d**. Obtained as a white solid (yield = 43%); ^1^H-NMR (400 MHz, CD_3_OD) δ 8.24 (d, *J* = 7.7 Hz, 2H), 7.75 (d, *J* = 7.7 Hz, 2H), 7.40–7.17 (m, 5H), 6.99 (d, *J* = 7.2 Hz, 2H), 6.69 (d, *J* = 7.2 Hz, 2H), 4.77–4.69 (m, 1H), 4.66 (d, *J* = 15.3 Hz, 1H), 4.41 (d, *J* = 15.3 Hz, 1H), 4.33 (s, 1.6H), 4.25 (d, *J* = 17.1 Hz, 1H), 4.23 (d, *J* = 16.2 Hz, 1H), 4.15 (d, *J* = 17.1 Hz, 0.2H), 4.13 (d, *J* = 17.1 Hz, 1H), 4.07 (d, *J* = 16.2 Hz, 1H), 3.97 (d, *J* = 17.1 Hz, 0.2H), 3.73 (s, 3H), 3.03 (s, 3H), 1.42–1.24 (m, 2H), (NH missed); ^13^C-NMR (101 MHz, CD_3_OD) δ 171.9, 171.0, 169.4, 168.9, 168.0, 159.3, 148.1, 142.8, 138.3, 129.3 (2C), 128.2 (2C), 127.7 (2C), 127.3, 126.8 (2C), 123.3 (2C), 113.7 (2C), 61.7, 61.5, 57.1, 54.3, 51.9, 45.2, 43.0, 29.3; HR-MS (ESI) *m*/*z*: [M + Na]^+^ calcd for C_30_H_31_BN_4_NaO_9_ 625.2082; found 625.2069.

*Methyl (2-(N-(4-methoxybenzyl)cinnamamido)-3-(4-methyl-2,6-dioxotetrahydro-2H-4λ^4^,8λ^4^-[1,3,2]oxazaborolo[2,3-b][1,3,2]oxazaborol-8-yl)propanoyl)glycinate* (**5o**): Prepared according to GP-A using α-borylaldehyde **1**, 4-methoxybenzylamine **2b**, trans cinnamic acid **4a, and** methyl isocyanoacetate **3e**. Obtained as a brown solid (yield = 38%); ^1^H-NMR (300 MHz, CD_3_OD, 8:2 rotameric mixture) δ 7.73–7.51 (m, 2H), 7.48–7.18 (m, 6H), 7.02–6.78 (m, 3H), 5.23 (m, 0.8H), 4.92 (m, 1.2H), 4.72 (d, *J* = 17.2 Hz, 0.8H), 4.62 (d, *J* = 14.8 Hz, 0.2H), 4.14 (d, *J* = 17.1 Hz, 1H), 4.11 (d, *J* = 16.7 Hz, 1H), 3.98 (d, *J* = 17.1 Hz, 1H), 3.96 (d, *J* = 16.7 Hz, 1H), 3.83 (d, *J* = 12.0 Hz, 2H), 3.74 (s, 3H), 3.69 (s, 3H), 2.98 (s, 2.4H), 2.88 (s, 0.6H), 1.70–1.53 (m, 0.2H), 1.44 (dd, *J* = 14.4, 8.3 Hz, 0.8H), 1.14 (dd, *J* = 14.4, 6.6 Hz, 0.8H), 1.05–0.93 (m, 0.2H), (NH missed); ^13^C-NMR (75 MHz, CD_3_OD, rotameric mixture) δ 177.4–171.8 (5C), 163.3 and 162.9 (1C), 148.8 and 146.8 (1C), 139.0 and 138.5 (1C), 134.1, 133.9, 133.5, 132.6 and 132.4 (2C), 131.9 and 131.8 (2C), 131.5, 122.4 and 120.7 (1C), 117.9 and 117.7 (2C), 65.9, 65.8, 63.0, 60.9, 58.3, 55.2, 45.6, 44.7, 33.3; HR-MS (ESI) *m*/*z*: [M + Na]^+^ calcd for C_28_H_32_BN_3_NaO_9_ 588.2129; found 588.2114.

*Methyl (2-(N-(4-Methoxybenzyl)-4-nitrobenzamido)-3-(4-methyl-2,6-dioxotetrahydro-2H-4λ^4^,8λ^4^-[1,3,2]oxazaborolo[2,3-b][1,3,2]oxazaborol-8-yl)propanoyl)glycinate* (**5p**): Prepared according to GP-A using α-borylaldehyde **1**, 4-methoxybenzylamine **2b**, 4-nitrobenzoic acid **4b, and** methyl isocyanoacetate **3d**. Obtained as a brown solid (yield = 42%); ^1^H-NMR (300 MHz, 115 °C, DMSO-d6) δ 8.19 (d, *J* = 8.2 Hz, 2H), 7.78 (s, 1H), 7.71 (d, *J* = 8.2 Hz, 2H), 7.25–7.05 (m, 2H), 6.79 (d, *J* = 7.9 Hz, 2H), 4.82–4.56 (m, 2H), 4.46 (d, *J* = 15.8 Hz, 1H), 4.17 (d, *J* = 17.0 Hz, 1H), 4.14 (d, *J* = 17.1 Hz, 1H), 3.97 (d, *J* = 17.0 Hz, 1H), 3.95 (d, *J* = 17.1 Hz, 1H), 3.83 (dd, *J* = 10.1, 5.5 Hz, 2H), 3.75 (s, 3H), 3.70 (s, 3H), 2.91 (s, 3H), 1.46–1.12 (m, 2H); ^13^C-NMR (75 MHz, 25 °C, DMSO-d6, rotameric mixture) δ 171.3–168.4 (5C), 158.2 and 157.9 (1C), 147.7 and 147.3 (1C), 143.8 and 143.1 (1C), 130.8, 128.9 (2C), 128.4, 127.6, 123.5 and 123.4 (2C), 113.5 and 113.3 (2C), 61.6 (2C), 59.3, 55.1, 51.8, 49.5, 45.8, 40.9, (B-C_α_ missed); ^11^B-NMR (128 MHz, CD_3_CN) δ 12.17; HR-MS (ESI) *m*/*z*: [M + Na]^+^ calcd for C_26_H_29_BN_4_NaO_11_ 607.1824; found 607.1828.

*(2-(N-(4-Bromophenyl)cinnamamido)-3-(tert-butylamino)-3-oxopropyl)boronic acid* (**6a**): Prepared according to GP-B starting from **5a**. Obtained as a white solid (yield = 97%); ^1^H-NMR (400 MHz, 115 °C, DMSO-d6) δ 7.63 (d, *J* = 8.4 Hz, 2H), 7.51 (d, *J* = 15.5 Hz, 1H), 7.40–7.28 (m, 7H), 6.92 (s, 1H), 6.26 (d, *J* = 15.5 Hz, 1H), 5.20 (dd, *J* = 9.4, 6.1 Hz, 1H), 1.29 (s, 9H), 0.98 (dd, *J* = 15.2, 9.4 Hz, 1H), 0.78 (dd, *J* = 15.2, 6.1 Hz, 1H), (BOH missed); ^13^C-NMR (75 MHz, 25 °C, DMSO-d6, 6:4 rotameric mixture) δ 172.7 and 171.5 (1C), 164.9, 141.6 and 141.1 (1C), 138.6, 134.5, 132.6, 132.0 (2C), 129.9 and 129.8 (1C), 129.0 (3C), 127.7 (2C), 121.5, 119.5 and 119.0 (1C), 58.8 and 56.9 (1C), 51.2 and 50.1 (1C), 28.5 and 28.3 (3C), 19.3; ^11^B-NMR (128 MHz, CD_3_CN) δ 32.01; HR-MS (ESI) *m*/*z*: [C_22_H_24_BrN_2_O_2_B(OMe)_2_ + Na]^+^ calcd for C_24_H_30_BBrN_2_NaO_4_ 523.1380; found 523.1391.

*(3-(tert-butylamino)-2-(N-(4-methoxybenzyl)cinnamamido)-3-oxopropyl)boronic acid* (**6b**): Prepared according to GP-B starting from **5b**. Obtained as a white solid (yield = 78%); ^1^H-NMR (400 MHz, CD_3_OD) δ 7.74 (d, *J* = 15.4 Hz, 1H), 7.60–7.55 (m, 2H), 7.41–7.37 (m, 3H), 7.27 (d, *J* = 8.5 Hz, 2H), 7.11 (d, *J* = 15.4 Hz, 1H), 6.94 (d, *J* = 8.5 Hz, 2H), 5.02 (d, *J* = 16.7 Hz, 1H), 4.70–4.62 (m, 2H), 3.79 (s, 3H), 1.37–1.27 (m, 9H), 1.18–1.14 (m, 1H), 0.88–0.80 (m, 1H), (NH, BOH missed); ^13^C-NMR (75 MHz, CD_3_OD) δ 176.0, 169.8, 161.2, 146.1, 136.4, 131.8, 130.5, 130.3 (2C), 129.9 (2C), 129.6 (2C), 118.7, 115.6 (2C), 62.9, 56.1, 53.8, 52.9, 31.1, 28.9 (3C); ^11^B-NMR (128 MHz, CD_3_CN) δ 31.55; HR-MS (ESI) *m*/*z*: [C_24_H_29_N_2_O_3_B(OMe)_2_ + Na]^+^ calcd for C_26_H_35_BN_2_NaO_5_ 489.2537; found 489.2549.

*(3-(tert-Butylamino)-2-(N-(5,5-dimethylhexyl)cinnamamido)-3-oxopropyl)boronic acid* (**6c**): Prepared according to GP-B starting from **5c**. Obtained as a white solid (yield = 61%); ^1^H-NMR (400 MHz, CD_3_OD) δ 7.75 (d, *J* = 15.4 Hz, 1H), 7.71–7.59 (m, 2H), 7.44 (m, 3H), 7.11 (d, *J* = 15.4 Hz, 1H), 4.50 (m, 1H), 3.99–3.72 (m, 1H), 3.53–3.35 (m, 1H), 1.78–1.48 (m, 2H), 1.48–1.11 (m, 15H), 0.90 (s, 9H), (NH, BOH missed); ^13^C-NMR (75 MHz, CD_3_OD) δ 171.2, 170.2, 146.2, 134.1, 131.9, 130.4 (2C), 129.7 (2C), 118.2, 63.4, 54.3, 52.3, 45.3, 32.1, 31.0, 30.0 (3C), 29.0 (3C), 23.2, (B-C_α_ missed); ^11^B-NMR (128 MHz, CD_3_CN) δ 30.99; HR-MS (ESI) *m*/*z*: [C_24_H_37_N_2_O_2_B(OMe)_2_ + Na]^+^ calcd for C_26_H_43_BN_2_NaO_4_ 481.3214; found 481.3234.

*(2-(N-(4-Bromophenyl)-4-nitrobenzamido)-3-(tert-butylamino)-3-oxopropyl)boronic acid (**6d**):* Prepared according to GP-B starting from **5d**. Obtained as a white solid (yield = 99%); ^1^H-NMR (400 MHz, 115 °C, DMSO-d6, 1:1 rotameric mixture) δ 8.12–7.96 (m, 2H), 7.53 (d, *J* = 8.5 Hz, 1H), 7.48 (d, *J* = 8.5 Hz, 1H), 7.45–7.38 (m, 2H), 7.28–7.18 (m, 2H), 6.91 (s, 1H), 5.29–5.13 (m, 1H), 1.32 (s, 9H), 1.10 (dd, *J* = 15.5, 9.0 Hz, 0.5H), 1.04 (dd, *J* = 14.1, 8.1 Hz, 0.5H), 0.97 (dd, *J* = 8.1, 5.1 Hz, 0.5H), 0.91 (dd, *J* = 15.5, 6.8 Hz, 0.5H), (BOH missed); ^13^C-NMR (75 MHz, 25 °C, DMSO-d6, 2:1 rotameric mixture) δ 170.9, 167.7, 147.4 and 147.2 (1C), 143.4, 138.8, 132.5 (2C), 131.9 and 131.8 (1C), 131.5 (2C), 128.9, 123.1 (2C), 120.8 and 120.7 (1C), 57.7, 50.3, 28.5 and 28.2 (3C), (B-C_α_ missed); ^11^B-NMR (128 MHz, CD_3_CN) δ 31.06; HRMS (ESI) *m*/*z*: [C_20_H_21_BrN_3_O_4_B(OMe)_2_ + Na]^+^ calcd for C_22_H_27_BBrN_3_NaO_6_ 542.1074; found 542.1061.

*(2-(N-Benzyl-4-nitrobenzamido)-3-(tert-butylamino)-3-oxopropyl)boronic acid* (**6e**): Prepared according to GP-B starting from **5e**. Obtained as a white solid (yield = 89%); ^1^H-NMR (400 MHz, 115 °C, DMSO-d6, 7:3 rotameric mixture) δ 8.27–8.16 (m, 2H), 7.82 (s, 0.3H), 7.67 (d, *J* = 8.6 Hz, 0.6H), 7.61 (d, *J* = 8.6 Hz, 1.4H), 7.30–7.16 (m, 5H), 6.59 (s, 0.7H), 4.77 (dd, *J* = 16.3, 9.2 Hz, 1H), 4.72–4.55 (m, 1H), 4.51 (dd, *J* = 16.3, 9.2 Hz, 1H), 1.30 (dd, *J* = 15.5, 6.8 Hz, 1H), 1.24 (s, 9H), 1.13 (dd, *J* = 15.5, 6.8 Hz, 1H), (BOH missed); ^13^C-NMR (75 MHz, 25 °C, DMSO-d6, rotameric mixture) δ 171.2–170.2 (2C), 147.6, 143.6, 139.0, 128.0 (2C), 127.7 (2C), 126.8 (2C), 126.3, 123.7 (2C), 59.9 and 59.4 (1C), 50.3 and 50.0 (1C), 46.5, 28.3 (3C), 18.5; HR-MS (ESI) *m*/*z*: [C_21_H_24_N_3_O_4_B(OMe)_2_ + Na]^+^ calcd for C_23_H_30_BN_3_NaO_6_ 478.2125; found 478.2104. Anal. calcd for C_21_H_26_BN_3_O_6_: C, 59.03; H, 6.13; B, 2.53; N, 9.83; O, 22.47; found: C, 59.33; H, 6.25; N, 9.56.

*(3-(tert-Butylamino)-2-(N-(5,5-dimethylhexyl)-4-nitrobenzamido)-3-oxopropyl)boronic acid* (**6f**): Prepared according to GP-B starting from **5f**. Obtained as a white solid (yield = 95%); ^1^H-NMR (400 MHz, CD_3_OD) δ 8.38 (d, *J* = 8.1 Hz, 2H), 7.69 (d, *J* = 8.1 Hz, 2H), 4.75–4.63 (m, 1H), 3.43–3.24 (m, 2H), 1.69–1.52 (m, 2H), 1.50–1.23 (m, 12H), 1.14–0.99 (m, 3H), 0.81 (s, 9H), (NH, BOH missed); ^13^C-NMR (75 MHz, CD_3_OD) δ 172.0, 171.8, 150.3, 143.7, 129.3 (2C), 125.3 (2C), 61.3, 51.2, 45.1 (2C), 31.9, 31.2, 29.9 (3C), 29.0 (3C), 23.3 (2C); ^11^B NMR (128 MHz, CD_3_CN) δ 31.22; HR-MS (ESI) *m*/*z*: [C_22_H_34_N_3_O_4_B(OMe)_2_ + Na]^+^ calcd for C_24_H_40_BN_3_NaO_6_ 500.2908; found 500.2924.

*(2-(N-(4-Bromophenyl)dec-9-enamido)-3-(tert-butylamino)-3-oxopropyl)boronic acid* (**6g**): Prepared according to GP-B starting from **5g**. Obtained as a white solid (yield = 99%); ^1^H-NMR (400 MHz, 115 °C, DMSO-d6) δ 7.59 (d, *J* = 8.2 Hz, 2H), 7.25 (d, *J* = 8.2 Hz, 2H), 7.12 (s, 1H), 5.87–5.73 (m, 1H), 5.09 (dd, *J* = 9.9, 5.8 Hz, 1H), 4.96 (dd, *J* = 21.4, 13.7 Hz, 2H), 2.08–1.89 (m, 4H), 1.51–1.42 (m, 2H), 1.37–1.14 (m, 17H), 0.86 (dd, *J* = 14.9, 9.9 Hz, 1H), 0.66 (dd, *J* = 14.9, 5.8 Hz, 1H), (BOH missed); ^13^C-NMR (75 MHz, 25 °C, DMSO-d6, rotameric mixture) δ 172.7 and 172.2 (1C), 172.0 and 171.5 (1C), 139.1, 138.8, 132.4 (2C), 131.9 (2C), 121.3 and 121.1 (1C), 114.6, 56.2, 50.0, 34.2, 33.1, 28.5, 28.4 (3C), 28.3, 28.2, 28.1, 24.8, 17.2; ^11^B-NMR (128 MHz, CD_3_CN) δ 31.21; HR-MS (ESI) *m*/*z*: [C_23_H_34_BrN_2_O_2_B(OMe)_2_ + Na]^+^ calcd for C_25_H_40_BBrN_2_NaO_4_ 545.2162; found 545.2150.

*(3-(tert-Butylamino)-2-(N-(4-methoxybenzyl)dec-9-enamido)-3-oxopropyl)boronic acid* (**6h**): Prepared according to GP-B starting from **5h**. Obtained as a white solid (yield = 74%); ^1^H-NMR (400 MHz, CD_3_OD, 8:2 rotameric mixture) δ 7.22 (d, *J* = 8.2 Hz, 2H), 6.95 (d, *J* = 8.2 Hz, 2H), 5.91–5.73 (m, 1H), 5.06–4.88 (m, 2H), 4.76 (d, *J* = 16.7 Hz, 1H), 4.57–4.45 (m, 2H), 3.80 (s, 3H), 2.61–2.41 (m, 2H), 2.12–1.96 (m, 2H), 1.79–1.52 (m, 2H), 1.52–1.18 (m, 17H), 1.10 (dd, *J* = 14.0 Hz, 1H), 0.79 (dd, *J* = 14.0 Hz, 1H), (NH, BOH missed); ^13^C-NMR (75 MHz, CD_3_OD) δ 179.5, 177.9, 163.4, 142.6, 134.8, 132.2, and 131.91 (2C), 117.9 (2C), 117.3, 64.4, 58.3, 55.7, 55.0, 37.3(2C), 33.3–32.5 (4C), 31.1 (3C), 28.9 (2C); ^11^B-NMR (128 MHz, CD_3_CN) δ 31.54; HR-MS (ESI) *m*/*z*: [C_25_H_39_N_2_O_3_B(OMe)_2_ + Na]^+^ calcd for C_27_H_45_BN_2_NaO_5_ 511.3319; found 511.3334.

*(3-(tert-Butylamino)-2-(N-(5,5-dimethylhexyl)dec-9-enamido)-3-oxopropyl)boronic acid* (**6i**): Prepared according to GP-B starting from **5i**. Obtained as a white solid (yield = 99%); ^1^H-NMR (400 MHz, 115 °C, DMSO-d6) δ 7.11 (s, 1H), 5.90–5.73 (m, 1H), 4.97 (dd, *J* = 23.7, 13.7 Hz, 2H), 4.80–4.53 (m, 1H), 3.34–3.17 (m, 2H), 2.32 (t, *J* = 6.6 Hz, 2H), 2.04 (d, *J* = 6.3 Hz, 2H), 1.63–1.43 (m, 6H), 1.43–1.11 (m, 20H), 0.88 (s, 9H), 0.79 (m, 1H), (BOH missed); ^13^C-NMR (75 MHz, 25 °C, CD_3_OD) δ 173.1, 172.3, 138.9, 114.7, 57.6, and 55.1 (1C), 44.6, 43.3 (2C), 33.2 (2C), 32.6 (2C), 30.7 (1C) 30.0 (1C), 29.2 (3C), 28.7 (2C), 28.3 (3C), 25.1 and 24.8 (2C), 22.1 and 21.7 (1C); ^11^B-NMR (128 MHz, CD_3_CN) δ 31.19; HR-MS (ESI) *m*/*z*: [C_25_H_47_N_2_O_2_B(OMe)_2_ + Na]^+^ calcd for C_27_H_53_BN_2_NaO_4_ 503.3996; found 503.4006.

*(3-(Cyclohexylamino)-2-(N-(4-methoxybenzyl)cinnamamido)-3-oxopropyl)boronic acid* (**6j**): Prepared according to GP-B starting from **5j**. Obtained as a white solid (yield = 83%); ^1^H-NMR (400 MHz, 115 °C, DMSO-d6, 6:4 rotameric mixture) δ 7.65 (d, *J* = 7.1 Hz, 1H), 7.58–7.46 (m, 3H), 7.43 (d, *J* = 7.1 Hz, 1H), 7.39–7.29 (m, 2H), 7.24–7.13 (m, 2H), 6.96–6.79 (m, 2H), 5.18–5.12 (m, 0.4H), 4.84 (d, *J* = 17.6 Hz, 0.6H), 4.77–4.69 (m, 0.6H), 4.62 (d, *J* = 15.2 Hz, 0.4H), 4.56 (d, *J* = 17.6 Hz, 0.6H), 4.49 (d, *J* = 15.2 Hz, 0.4H), 3.70 (s, 3H), 3.48–3.37 (m, 1H), 1.64–1.44 (m, 4H), 1.32 (dd, *J* = 15.2 Hz, 1H), 1.25–1.00 (m, 6H), 0.85 (dd, *J* = 15.2, 5.9 Hz, 0.5H), 0.77 (dd, *J* = 15.2, 5.9 Hz, 0.5H), (NH, BOH missed); ^13^C-NMR (75 MHz, 25 °C, DMSO-d6, 6:4 rotameric mixture) δ 170.5 and 169.9 (1C), 166.5 and 166.2 (1C), 158.1 and 158.0 (1C), 141.4, 135.0, and 135.0 (1C), 131.3 and 131.2 (2C), 129.6, 128.8 (3C), 127.8 (2C), 127.6, 119.6, 113.8, 113.5, 55.5, 55.0, 47.8, and 47.6 (1C), 47.1, 32.1 (2C), 29.0, 25.2, 24.3 (2C); ^11^B-NMR (128 MHz, CD_3_CN) δ 31.40; HR-MS (ESI) *m*/*z*: [C_26_H_32_N_2_O_4_B(OMe) + Na]^+^ calcd for C_27_H_35_BN_2_NaO_5_ 501.2537; found 501.2549.

*(3-(Cyclohexylamino)-2-(N-(4-methoxybenzyl)-4-nitrobenzamido)-3-oxopropyl)boronic acid* (**6k**): Prepared according to GP-B starting from **5k**. Obtained as a white solid (yield = 66%); ^1^H-NMR (400 MHz, CD_3_OD, 6:4 rotameric mixture) δ 8.45–8.25 (m, 2H), 7.70 (d, *J* = 8.0 Hz, 2H), 7.31 (d, *J* = 7.4 Hz, 1H), 7.09 (d, *J* = 7.4 Hz, 1H), 6.94–6.76 (m, 2H), 5.27 (d, *J* = 16.2 Hz, 0.4H), 4.79–4.64 (m, 0.6H), 4.64–4.50 (m, 1.2H), 4.46 (d, *J* = 16.2 Hz, 0.4H), 4.41–4.29 (m, 0.4H), 3.81 (s, 1.2H), 3.78 (s, 1.8H), 3.71–3.48 (m, 1H), 1.93–1.70 (m, 4H), 1.70–1.54 (m, 2H), 1.47–1.05 (m, 5H), 1.05–0.74 (m, 1H), (NH, BOH missed); ^13^C NMR (75 MHz, CD_3_OD, 6:4 rotameric mixture) δ 174.0 and 173.6 (1C), 169.6 and 169.5 (1C), 161.3 and 160.7 (1C), 150.2, 144.4 and 143.6 (1C), 132.2, 130.5, 130.1, 129.5, 129.1, 125.2 (2C), 115.5, 115.2, 61.4, and 60.8 (1C), 56.0, 54.2, 51.6, and 50.6 (1C), 33.8 (2C), 31.0, 26.8, 26.3 (2C); ^11^B-NMR (128 MHz, CD_3_CN) δ 31.58; HR-MS (ESI) *m*/*z*: [C_24_H_28_N_3_O_5_B(OMe)_2_ + Na]^+^ calcd for C_26_H_34_BN_3_NaO_7_ 534.2388; found 534.2373.

*(2-(N-(4-Methoxybenzyl)cinnamamido)-3-oxo-3-(pentylamino)propyl)boronic acid* (**6l**): Prepared according to GP-B starting from **5l**. Obtained as a white solid (yield = 99%); ^1^H-NMR (400 MHz, DMSO-d6, rotameric mixture) δ 7.62–7.26 (m, 7H), 7.18 (d, *J* = 7.7 Hz, 2H), 6.88 (m, 3H), 5.29–5.07 (m, 0.6H), 4.82 (d, *J* = 17.2 Hz, 0.6H), 4.79–4.73 (m, 0.4H), 4.70 (d, *J* = 17.2 Hz, 0.2H), 4.55 (t, *J* = 8.1 Hz, 1.2H), 3.72 (s, 3H), 3.16–2.85 (m, 2H), 1.53–0.99 (m, 6H), 0.99–0.61 (m, 5H), (BOH missed); ^13^C-NMR (75 MHz, DMSO-d6, rotameric mixture) δ 171.3 and 170.9 (1C), 166.4 and 165.1 (1C), 158.5 and 158.1 (1C), 141.8 and 141.1 (1C), 135.3 and 135.0 (1C), 131.1 and 130.3 (1C), 129.6, 128.8 (2C), 128.4, 128.0 and 127.8 (2C), 127.5, 119.6 and 118.7 (1C), 113.9 and 113.4 (2C), 55.6, 55.0, 47.3, 28.6 (3C), 21.9, 15.7, 13.9; ^11^B NMR (128 MHz, CD_3_CN) δ 31.20; HR-MS (ESI) *m*/*z*: [C_25_H_31_N_2_O_3_B(OMe)_2_ + Na]^+^ calcd for C_27_H_37_BN_2_NaO_5_ 503.2693; found 503.2704.

*(2-(N-(4-Methoxybenzyl)-4-nitrobenzamido)-3-oxo-3-(pentylamino)propyl)boronic acid* (**6m**): Prepared according to GP-B starting from **5m**. Obtained as a white solid (yield = 64%); ^1^H-NMR (400 MHz, CD_3_OD, 6:4 rotameric mixture) δ 8.41–8.26 (m, 2H), 7.74 (d, *J* = 7.2 Hz, 2H), 7.31 (d, *J* = 7.2 Hz, 1H), 7.12 (d, *J* = 7.2 Hz, 1H), 6.94–6.82 (m, 2H), 5.26 (d, *J* = 15.7 Hz, 0.4H), 4.73–4.65 (m, 0.5H), 4.55 (dd, *J* = 15.7 Hz, 1.2H), 4.45–4.41 (m, 0.5H), 4.37 (d, *J* = 15.8 Hz, 0.4H), 3.81 (s, 1.3H), 3.78 (s, 1.7H), 3.28–3.21 (m, 1.1H), 3.19–3.08 (m, 0.9H), 1.61–1.42 (m, 2H), 1.42–1.22 (m, 6H), 0.96–0.90 (m, 3H), (NH, BOH missed); ^13^C-NMR (101 MHz, CD_3_OD, rotameric mixture) δ 176.1, 173.4, 162.5, 149.2, 143.2, 136.0, 129.2 (2C), 128.3 (2C), 124.0 (2C), 114.4 (2C), 61.6 and 61.0 (1C), 55.1, 54.5, 40.3, 29.9, 29.4, 29.2, 22.6. (B-C_α_ missed); ^11^B-NMR (128 MHz, CD_3_CN) δ 31.59; HR-MS (ESI) *m*/*z*: [C_23_H_28_N_3_O_5_B(OMe)_2_ + Na]^+^ calcd for C_25_H_34_BN_3_NaO_7_ 522.2388; found 522.2401.

*(3-(Benzylamino)-2-(N-(4-methoxybenzyl)-4-nitrobenzamido)-3-oxopropyl)boronic acid* (**6n**): Prepared according to GP-B starting from **5n**. Obtained as a white solid (yield = 97%); ^1^H-NMR (400 MHz, CD_3_OD) δ 8.31–8.17 (m, 2H), 7.64 (d, *J* = 5.9 Hz, 2H), 7.41–7.23 (m, 6H), 7.06 (d, *J* = 7.3 Hz, 1H), 6.91 (d, *J* = 7.7 Hz, 1H), 6.81 (d, *J* = 7.3 Hz, 1H), 4.62–4.21 (m, 5H), 3.81 (s, 3H), 1.44–1.19 (m, 2H), (NH, BOH missed); ^13^C-NMR (75 MHz, CD_3_OD) δ 169.8, 169.4, 167.0, 158.9, 150.1, 142.8, 138.2, 130.3, 130.2, 129.9 (2C), 129.1 (2C), 128.7 (2C), 125.1 (3C), 115.4, 115.3, 61.6, 56.1, 53.7, 44.6, 31.0; ^11^B-NMR (128 MHz, CD_3_CN) δ 30.99; HR-MS (ESI) *m*/*z*: [C_25_H_24_N_3_O_5_B(OMe)_2_ + Na]^+^ calcd for C_27_H_30_BN_3_NaO_7_ 542.2075; found 542.2058.

*(3-((2-Methoxy-2-oxoethyl)amino)-2-(N-(4-methoxybenzyl)cinnamamido)-3-oxopropyl)boronic acid* (**6o**): Prepared according to GP-B starting from **5o**. Obtained as a brown solid (yield = 99%); ^1^H-NMR (400 MHz, CD_3_OD) δ 7.79 (d, *J* = 15.3 Hz, 1H), 7.59–7.53 (m, 2H), 7.43–7.34 (m, 3H), 7.26 (d, *J* = 8.2 Hz, 2H), 7.08 (d, *J* = 15.3 Hz, 1H), 6.94 (d, *J* = 8.2 Hz, 2H), 5.03–4.93 (m, 2H), 4.74 (d, *J* = 17.2 Hz, 1H), 3.95 (d, *J* = 9.4 Hz, 2H), 3.79 (s, 3H), 3.75 (s, 3H), 1.03–0.86 (m, 2H), (NH, BOH missed); ^13^C-NMR (75 MHz, CD_3_OD) δ 175.2, 171.9, 170.5, 161.1, 146.5, 136.3, 131.8, 130.3 (2C), 129.6 (2C), 129.5 (2C), 128.6, 118.6, 115.6 (2C), 60.6, 56.0, 52.9, 51.9, 42.4, 31.0; ^11^B-NMR (128 MHz, CD_3_CN) δ 31.50; HR-MS (ESI) *m*/*z*: [C_23_H_25_N_2_O_5_B(OMe)_2_ + Na]^+^ calcd for C_25_H_31_BN_2_NaO_7_ 505.2122; found 505.2135.

*(3-((2-Methoxy-2-oxoethyl)amino)-2-(N-(4-methoxybenzyl)-4-nitrobenzamido)-3-oxopropyl)boronic acid* (**6p**): Prepared according to GP-B starting from **5p**. Obtained as a brown solid (yield = 91%); ^1^H-NMR (400 MHz, CD_3_OD, rotameric mixture) δ 8.44–8.22 (m, 2H), 7.91–7.64 (m, 2H), 7.46–6.75 (m, 4H), 5.38 (d, *J* = 14.8 Hz, 0.3H), 5.22–4.97 (m, 0.4H), 4.74–4.64 (m, 0.3H), 4.64–4.44 (m, 1.3H), 4.41–4.31 (m, 0.7H), 4.02–3.89 (m, 2H), 3.78 (s, 3H), 3.77 (s, 3H), 1.71–1.48 (m, 1H), 1.48–1.25 (m, 1H), (NH, BOH missed); ^13^C-NMR (101 MHz, CD_3_OD, rotameric mixture) δ 172.6, 171.8, 170.9, 159.7, 149.1, 143.2, and 142.9 (1C), 131.2, 129.3, and 129.1 (2C), 128.4 (2C), 124.0 (2C), 114.2 (2C), 60.6, 55.0, 52.0, 42.6, 41.2, 30.0; ^11^B NMR (128 MHz, CD_3_CN) δ 31.57; HR-MS (ESI) *m*/*z*: [C_21_H_22_N_3_O_7_B(OMe)_2_ + Na]^+^ calcd for C_23_H_28_BN_3_NaO_9_ 524.1816; found 524.1827.

*N-(tert-Butyl)-3-(4-methyl-2,6-dioxotetrahydro-2H-4λ^4^,8λ^4^-[1,3,2]oxazaborolo[2,3-b][1,3,2]oxazaborol-8-yl)-2-(2-oxoazetidin-1-yl)propenamide* (**8a**): Prepared according to GP-C using α-borylaldehyde **1**, β-alanine **7a** and tert-butyl isocyanide **3a**. Obtained as a white solid (yield = 72%); ^1^H-NMR (400 MHz, CD_3_OD) δ 4.35 (dd, *J* = 9.1, 6.2 Hz, 1H)), 4.18 (d, *J* = 17.1 Hz, 1H), 4.17 (d, *J* = 16.9 Hz, 1H), 4.02 (d, *J* = 17.1 Hz, 1H), 3.99 (d, *J* = 16.9 Hz, 1H), 3.47 (dd, *J* = 8.4, 4.9 Hz, 1H), 3.41 (dd, *J* = 8.4, 4.9 Hz, 1H), 3.01 (s, 3H), 2.94–2.86 (m, 2H), 1.36 (s, 9H), 1.22 (dd, *J* = 14.4, 9.1 Hz, 1H), 1.13 (dd, *J* = 14.4, 6.2 Hz, 1H), (NH missed); ^13^C-NMR (101 MHz, CD_3_OD) δ 171.8 and 171.7 (1C), 169.9, 169.5 (2C), 62.3 and 62.2 (2C), 54.3, 53.8, 51.5, 38.4, 35.3, 30.0, 28.1 (3C); ^11^B-NMR (128 MHz, CD_3_CN) δ 12.15; HR-MS (ESI) *m*/*z*: [M + Na]^+^ calcd for C_15_H_24_BN_3_NaO_6_ 376.1656; found 376.1647.

*N-(tert-Butyl)-3-(4-methyl-2,6-dioxotetrahydro-2H-4λ^4^,8λ^4^-[1,3,2]oxazaborolo[2,3-b][1,3,2]oxazaborol-8-yl)-2-((S)-2-oxo-4-phenylazetidin-1-yl)propenamide* (**8b**): Prepared according to GP-C using α-borylaldehyde **1**, S-β-phenylalanine **7b** and tert-butyl isocyanide **3a**. Obtained as a white solid (yield = 36%); ^1^H-NMR (400 MHz, CD_3_OD, 3:2 diasteroisomeric mixture, presence of rotamers) δ 7.58–7.15 (m, 5H), 4.86 (dd, *J* = 5.3, 2.3 Hz, 0.1H), 4.78 (dd, *J* = 5.3, 2.3 Hz, 0.3H), 4.70 (ddd, *J* = 10.8, 5.3, 2.3 Hz, 0.6H), 4.32–4.28 (m, 0.2H), 4.18–4.11 (m, 2.6H), 4.02–3.94 (m, 2H), 3.87–3.83 (m, 0.2H), 3.44–3.35 (m, 1H), 3.00 (s, 1.8H), 2.99 (s, 1.2H), 2.83–2.75 (m, 1H), 1.55–1.06 (m, 11H), (NH missed); ^13^C-NMR (75 MHz, CD_3_OD, 3:2 diasteroisomeric mixture, presence of rotamers) δ 173.2–170.5 (4C), 140.6 and 140.4 (1C), 130.2 (2C), 129.9 and 129.7 (1C), 128.3 (2C), 63.5, 63.4, and 63.3 (1C), 57.3–57.0 (1C), 47.3 and 46.2 (1C), 46.9 and 46.7 (1C), 32.1, 31.0, 29.1, and 29.0 (3C), 24.0; HR-MS (ESI) *m*/*z*: [M + Na]^+^ calcd for C_21_H_28_BN_3_NaO_6_ 452.1969; found 452.1985.

*Methyl (2S)-1-(1-(tert-butylamino)-3-(4-methyl-2,6-dioxotetrahydro -2H-4λ^4^,8λ^4^-[1,3,2]oxazaborolo[2,3-b][1,3,2]oxazaborol-8-yl)-1-oxopropan-2-yl)-4-oxoazetidine-2-carboxylate (**8c**): Prepared according to GP-C using α-borylaldehyde***1**, (3S)-3-ammonio-4-methoxy-4- oxobutanoate **7c** and tert-butyl isocyanide **3a**. Obtained as a white solid (yield = 52%); ^1^H-NMR (400 MHz, 115 °C, DMSO-d6, 1:1 diasteroisomeric mixture) δ 7.71 (s, 0.5H), 7.56 (s, 0.5H), 4.38–4.33 (m, 0.5H), 4.21–4.13 (m, 3H), 4.06–3.94 (m, 2.5H), 3.72 (s, 1.5H), 3.61 (s, 1.5H), 3.12 (dd, *J* = 14.2, 5.8 Hz, 1H), 2.91 (s, 1.5H), 2.89 (s, 1.5H), 2.78 (dd, *J* = 14.2, 2.0 Hz, 1H), 1.26 (s, 4.5H), 1.22 (s, 4.5H), 1.15 (d, *J* = 8.1 Hz, 0.5H), 1.10–0.99 (m, 1H), 0.93 (dd, *J* = 14.6, 8.1 Hz, 0.5H); ^13^C-NMR (75 MHz, 25 °C, DMSO-d6, 1:1 diasteroisomeric mixture) δ 172.1 and 171.3 (1C), 169.0, 168.8, 168.6, 165.8, and 165.0 (1C), 61.8 (2C), 55.2 and 52.3 (1C), 52.1 and 51.7 (1C), 51.0 and 49.0 (1C), 50.2 and 50.1 (1C), 45.7, 41.4, and 40.8 (1C), 29.0, 28.3 (3C); HR-MS (ESI) *m*/*z*: [M + Na]^+^ calcd for C_17_H_26_BN_3_NaO_8_ 434.1711; found 434.1723.

*(3-(tert-Butylamino)-3-oxo-2-(2-oxoazetidin-1-yl)propyl)boronic acid* (**9a**): Prepared according to **GP-B** starting from **8a**. Obtained as a white solid (yield = 99%); ^1^H-NMR (400 MHz, CD_3_OD) δ 4.35 (t, *J* = 7.8 Hz, 1H, CH), 3.41–3.30 (m, 2H, CH_2_-N), 2.92–2.86 (m, 2H, CH_2_-CO), 1.35 (s, 9H, *tert*-Bu), 1.21 (dd, *J* = 15.7, 7.8 Hz, 2H, CH_2_-B), (NH, BOH missed); ^13^C-NMR (101 MHz, CD_3_OD) δ 172.1 (CO), 169.3 (CO), 54.3 (CH-N), 51.7 ((CH_3_)_3_C-N), 38.1 (CH_2_-N), 35.3 (CH_2_-CO), 30.0 (CH_2_-B), 28.0 (3C, (CH_3_)_3_C); ^11^B-NMR (128 MHz, CD_3_OD) δ 28.84; HR-MS (ESI) *m*/*z*: [M + Na]^+^ calcd for C_10_H_19_BN_2_NaO_4_ 265.1336; found 265.1357.

*(3-(tert-Butylamino)-3-oxo-2-((S)-2-oxo-4-phenylazetidin-1-yl)propyl)boronic acid* (**9b**): Prepared according to GP-B starting from **8b**. Obtained as a white solid (yield = 99%); ^1^H-NMR (400 MHz, 115 °C, DMSO-d6, 6:4 diastereoisomeric mixture) δ 7.48–7.26 (m, 5H, Ph), 6.87 (s, 0.4H, NH), 6.71 (s, 0.6H, NH), 4.83–4.75 (m, 0.4H, CH-Ph), 4.72–4.62 (m, 0.6H, CH-Ph), 4.12–3.99 (m, 1H, CH), 3.28 (dd, *J* = 14.5, 5.4 Hz, 1H, CH-CO), 2.74–2.63 (m, 1H, CH-CO), 1.44–1.11 (m, 10H, tert-Bu, CH-B), 0.98 (d, *J* = 7.4 Hz, 1H, CH-B), (BOH missed); ^13^C-NMR (75 MHz, 25 °C, DMSO-d6, 6:4 diastereoisomeric mixture) δ 170.6 and 169.9 (1C, CO), 167.5 and 167.1 (1C, CO), 140.3 and 139.6 (1C, C(Ph)), 128.6 (2C, CH(Ph)), 127.9 (CH(Ph)), 126.6 (2C, CH(Ph)), 54.8–52.9 (1C, CH-N), 50.1 ((CH_3_)_3_C-N), 45.7 (CH_2_-CO), 31.6–29.5 (1C, CH-Ph), 29.0 (CH_2_-B), 28.4 and 28.2 (3C, (CH_3_)_3_C); ^11^B-NMR (128 MHz, CD_3_CN) δ 31.09; HR-MS (ESI) *m*/*z*: [C_16_H_21_N_2_O_2_B(OMe)_2_ + Na]^+^ calcd for C_18_H_27_BN_2_NaO_4_ 369.1962; found 369.1970.

*(3-(tert-Butylamino)-2-((S)-2-(methoxycarbonyl)-4-oxoazetidin-1-yl)-3-oxopropyl)boronic acid* (**9c**): Prepared according to GP-B starting from **8c**. Obtained as a white solid (yield = 99%); ^1^H-NMR (400 MHz, CD_3_OD, 1:2 diastereoisomeric mixture) δ 4.42-4.21 (m, 2H, CH-N, CH-CO_2_), 3.83 and 3.80 (s, 3H, OCH_3_), 3.23 (dd, *J* = 14.4, 5.6 Hz, 1H, CH-CO), 2.94 (q, *J* = 14.4, 11.8 Hz, 1H, CH-CO), 1.47–1.21 (m, 11H, tert-Bu, CH_2_-B), (NH, BOH missed); ^13^C-NMR (75 MHz, CD_3_OD, 1:2 diastereoisomeric mixture) δ 173.6 (CO_2_), 172.8 (CO), 169.5 (CO), 57.4 and 55.9 (1C, CH-N), 53.4 and 53.2 (1C, CH-CO_2_), 52.8 ((CH_3_)_3_C-N), 51.8 (CH_3_O), 42.3 (CH_2_-CO), 31.0 (CH_2_-B), 29.0 (3C, (CH_3_)_3_C); ^11^B-NMR (128 MHz, CD_3_CN) δ 31.24; HR-MS (ESI) *m*/*z*: [C_12_H_19_N_2_O_4_B(OMe)_2_+Na]^+^ calcd for C_14_H_25_BN_2_NaO_6_ 351.1703; found 351.1724.

*8-((1-(Cyclohexylmethyl)-1H-imidazol-5-yl) methyl)-4-methyldihydro-4λ^4^,8λ^4^-[1,3,2]oxazaborolo[2,3-b][1,3,2]oxazaborole-2,6(3H,5H)-dione* (**10**): Prepared according to GP-D. Obtained as a brown solid (yield = 32%); ^1^H-NMR (300 MHz, (CD_3_)_2_CO) δ 7.65 (s, 1H, CH(Im)), 6.81 (s, 1H, CH(Im)), 4.27 (d, *J* = 16.9 Hz, 2H, CH_2_-N(MIDA)), 4.05 (d, *J* = 16.9 Hz, 2H, CH_2_-N(MIDA)), 3.88 (d, *J* = 7.4 Hz, 2H, CH_2_-N(Im)), 3.18 (s, 3H, N-CH_3_), 1.88–1.52 (m, 9H, CH and 4 CH_2_(Cy)), 1.26–1.15 (m, 2H, CH_2_(Cy)), 1.12–0.93 (m, 2H, CH_2_-B); ^13^C-NMR (101 MHz, CD_3_OD) δ 169.4 (2C, CO_2_), 130.7 (CH(Im)), 129.3 (C(Im)), 118.1 (CH(Im)), 61.5 (2C, CH_2_-N (MIDA)), 52.4 and 52.1 (1C, CH_2_-N), 42.2 (CH_3_-N (MIDA)), 38.5 (CH(Cy)), 30.5 (2C, CH_2_(Cy)), 30.0 (CH_2_(Cy)), 26.5 (CH_2_(Cy)), 25.9 (2C, CH_2_(Cy), CH_2_-B); HR-MS (ESI) *m*/*z*: [M + H]^+^ calcd for C_16_H_25_BN_3_O_4_ 334.1938; found 334.1927.

Single crystal X-ray diffraction analysis of compounds **5a** and **6d**: Single crystals of **5a** and **6d** were obtained by slow evaporation from the mother liquor. All X-ray data collections were performed at room temperature with a Bruker AXS Smart 3-circle diffractometer equipped with an APEX-II CCD detector. Graphite–monochromated Mo Kα radiation (λ = 0.71073 Å) at a nominal power of 50 kV × 30 mA of the sealed X-ray tube was employed. Highly-redundant ω-scans (Δω = 0.25 deg) at variable φ angles were performed, resulting in 100% complete spheres of data up to 2θ = 46.5 deg (**5a**) and 2θ = 52.7 deg (**6d**). Diffraction patterns were corrected by absorption and beam anisotropy using SADABS [31], and then phased by direct methods with Shelx [32]. Both compounds crystallized in centrosymmetric space groups (**5a**: P2_1_/c; **6d**: P$\bar{1}$) as 1:1 racemates. The interested reader can find full details of the diffraction analysis in the Supporting Information.

### 4.6. In Vitro Antibacterial Susceptibility Assays

The potential of each molecule described herein to increase the activity of a β-lactam antibiotic was investigated using recombinant isogenic *E. coli* strains producing different types of β-lactamases, including the class A ESBL CTX-M-15 and the KPC-2 carbapenemase, the *Enterobacter cloacae* AmpC enzyme, the plasmid-encoded class C CMY-2, and several OXA-typ enzymes (OXA-10 and the carbapenemases OXA-23, OXA-40, and OXA-48). The β-lactamase genes were cloned in the pLB-II vector as previously described [33], and subsequently used to transform *E. coli* DH5α. In addition, clinical isolates present in our collection of antibiotic-resistant clinical isolates were also tested. Minimal inhibitory concentrations (MICs) of ampicillin, imipenem, and cefepime were determined in triplicate by the broth microdilution method in Mueller-Hinton broth, according to the Clinical Laboratory Standards Institute guidelines [34], in the absence and presence of 16 or 32 μg/mL of the tested compound. Plates were incubated aerobically at 35 ± 1 °C for 18–24 h before reading.

### 4.7. Molecular Modeling Studies, Model System, and MD Setup

The OXA-23/Mer complex computational model was built taking into account the crystal structures acquired at different pH values (Protein Data Bank entries 4JF4 and 4JF6) [35,36], as reported on our previous paper in which the meropenem hydrolysis mechanism was predicted by QM/MM simulations [37]. Here, the previously optimized OXA-23 model was fully solvated without the presence of meropenem into the catalytic site, in order to be ready for the covalent docking of the compound under investigation. The TIP3P [38] model was employed to describe the solvent molecules’ effect, and the OPLS3e force fields [39] were applied to simulate the enzyme atoms. The neutrality of the system was ensured by adding two sodium ions; in fact, after the calculation of the enzyme charge, the “protein preparation tool” of Maestro inserted the sodium ions on the protein surface where the negative charge was the highest. Then, MD simulations were accomplished by performing the following steps: (1) 100 ps of Brownian dynamics under isocore conditions (NVT) at a temperature of 10 K, restraining the enzyme heavy atoms; (2) 12 ps long MD simulations NVT, at the same temperature, while restraining the solute heavy atoms; (3) 12 ps long MD simulations in an isothermal–isobaric ensemble (NPT) at a temperature of 10 K, with restraints on the solute heavy atoms; (4) 24 ps of MD simulations in NPT conditions with no restraints; (5) 250 ns long MD simulations in NPT conditions. Finally, by visual inspection with VMD, [40] we ensured that the thermalization and the MD simulations did not cause any structural distortion.

### 4.8. Covalent Docking

The structure of compound **6e** was drawn by the “build” tool of Maestro software and prepared for docking by the LigPrep module of Maestro (2019-4 release). This tool generates both enantiomers of the ligand, checks and corrects any geometrical distortions, assigns the OPLS3e force field to the atoms, and performs the energy minimization of the ligand model. Covalent docking on Ser79 residue of OXA-23 was performed by “CovDock” module of Glide [41]. The default parameters of docking were adopted for this calculation in which the boronic acid’s electrophile attack on the reactive residue Ser79 was simulated. The attained docking results highlighted that the R enantiomer of compound **6e** acquired the highest score. Thus, the complex composed by the docking solution with the highest score in the catalytic site of OXA-23 was submitted to geometrical optimization and MD simulations, following the protocol previously adopted for the OXA-23 model. Finally, the MD trajectory was carefully analyzed by the “simulation interactions diagram” module of Maestro software, in order to evaluate the stability of the ligand binding pose in the catalytic site of the β-lactamase.

## Figures and Tables

**Table 1:** In vitro antimicrobial susceptibility testing performed on both isogenic *Escherichia coli* laboratory strains and clinical isolates producing various β-lactamases (BLs) in the presence of fixed concentrations (16 or 32 µg/mL) of the selected compounds (AMP, ampicillin; IPM, imipenem; FEP, cefepime).

Strain	BL Produced	MIC (µg/mL) *^a^*				
Isogenic strains *^b^*		AMP	AMP + 6m	AMP + 6b	AMP + 6k	AMP + 6e
*E. coli* DH5α(pLBII-CTX-M-15)	CTX-M-15	512	512	512	512	512
*E. coli* DH5α(pLBII-KPC-2)	KPC-2	256	256	256	256	256
*E. coli* DH5α(pLBII-AmpC-EC)	*Enterobacter cloacae* AmpC	128	128	128	128	128
*E. coli* DH5α(pLBII-CMY-2)	CMY-2	128	128	128	128	64
*E. coli* DH5α(pLBII-OXA-10)	OXA-10	512	512	512	512	512
*E. coli* DH5α(pLBII-OXA-23)	OXA-23	512	256	256	256	128
*E. coli* DH5α(pLBII-OXA-40)	OXA-24/40	256	128	128	128	128
*E. coli* DH5α(pLBII-OXA-40)	OXA-48	64	64	64	64	64
Clinical isolates *^c^*		IPM	IPM + **6m**	IPM + **6b**	IPM + **6k**	IPM + **6e**
*E. coli* SI-44	KPC-3, CTX-M-15, TEM-1	4	4	2	4	2
*K. pneumoniae* SI-109	KPC-3, SHV, TEM-1	16	16	16	16	16
		FEP	FEP + 6m	FEP + 6b	FEP + 6k	FEP + 6e
*E. coli* 26sm02	CMY-2	2	1	1	2	2

*^a^* Minimal Inhibitory Concentration (MIC) values determined in triplicate. *^b^* Compounds tested at a fixed concentration of 16 µg/mL. *^c^* Compounds tested at a fixed concentration of 32 µg/mL.

## Supplementary Materials

The following are available at https://www.mdpi.com/2079-6382/9/5/249/s1. ^1^H and ^13^C-NMR spectra of all products; ^11^B-NMR spectra of compounds **6a**–**p**, **9a**–**c**; Single crystal X-ray diffraction analysis of compounds **5a** and **6d**.

## References

[1] Marvin Antonio S.-U. (2019). Chemico-Biological Activity and Medicinal Chemistry of Boron-Containing Compounds. Curr. Med. Chem..

[2] Ban H.S., Nakamura H. (2015). Boron-Based Drug Design. Chem. Rec..

[3] Smoum R., Rubinstein A., Dembitsky V.M., Srebnik M. (2012). Boron containing compounds as protease inhibitors. Chem. Rev..

[4] Fernandes G.F.S., Denny W.A., Dos Santos J.L. (2019). Boron in drug design: Recent advances in the development of new therapeutic agents. Eur. J. Med. Chem..

[5] Zhou J., Stapleton P., Haider S., Healy J. (2018). Boronic acid inhibitors of the class A beta-lactamase KPC-2. Bioorg. Med. Chem..

[6] Rojas L.J., Taracila M.A., Papp-Wallace K.M., Bethel C.R., Caselli E., Romagnoli C., Winkler M.L., Spellberg B., Prati F., Bonomo R.A. (2016). Boronic Acid Transition State Inhibitors Active against KPC and Other Class A beta-Lactamases: Structure-Activity Relationships as a Guide to Inhibitor Design. Antimicrob. Agents Chemother..

[7] Hamrick J.C., Docquier J.D., Uehara T., Myers C.L., Six D.A., Chatwin C.L., John K.J., Vernacchio S.F., Cusick S.M., Trout R.E.L. (2020). VNRX-5133 (Taniborbactam), a Broad-Spectrum Inhibitor of Serine- and Metallo-beta-Lactamases, Restores Activity of Cefepime in Enterobacterales and Pseudomonas aeruginosa. Antimicrob. Agents Chemother..

[8] Lopez A., Clark T.B., Parra A., Tortosa M. (2017). Copper-Catalyzed Enantioselective Synthesis of beta-Boron beta-Amino Esters. Org. Lett..

[9] Andrés P., Ballano G., Calaza M.I., Cativiela C. (2016). Synthesis of α-aminoboronic acids. Chem. Soc. Rev..

[10] Diaz D.B., Scully C.C., Liew S.K., Adachi S., Trinchera P., St Denis J.D., Yudin A.K. (2016). Synthesis of Aminoboronic Acid Derivatives from Amines and Amphoteric Boryl Carbonyl Compounds. Angew Chem. Int. Ed. Engl..

[11] He Z., Yudin A.K. (2011). Amphoteric alpha-boryl aldehydes. J. Am. Chem. Soc..

[12] Tan J., Cognetta A.B., Diaz D.B., Lum K.M., Adachi S., Kundu S., Cravatt B.F., Yudin A.K. (2017). Multicomponent mapping of boron chemotypes furnishes selective enzyme inhibitors. Nat. Commun..

[13] Adachi S., Cognetta A.B., Niphakis M.J., He Z., Zajdlik A., St Denis J.D., Scully C.C., Cravatt B.F., Yudin A.K. (2015). Facile synthesis of borofragments and their evaluation in activity-based protein profiling. Chem. Commun..

[14] Šterman A., Sosič I., Gobec S., Časar Z. (2019). Synthesis of aminoboronic acid derivatives: An update on recent advances. Org. Chem. Front..

[15] Kaldas S.J., Rogova T., Nenajdenko V.G., Yudin A.K. (2018). Modular Synthesis of beta-Amino Boronate Peptidomimetics. J. Org. Chem..

[16] Herrera R.P., Marqués-López E. (2015). Multicomponent Reactions: Concepts and Applications for Design and Synthesis.

[17] Rainoldi G., Begnini F., de Munnik M., Lo Presti L., Vande Velde C.M.L., Orru R., Lesma G., Ruijter E., Silvani A. (2018). Sequential Multicomponent Strategy for the Diastereoselective Synthesis of Densely Functionalized Spirooxindole-Fused Thiazolidines. ACS Comb. Sci..

[18] Lesma G., Meneghetti F., Sacchetti A., Stucchi M., Silvani A. (2014). Asymmetric Ugi 3CR on isatin-derived ketimine: Synthesis of chiral 3, 3-disubstituted 3-aminooxindole derivatives. Beilstein J. Org. Chem..

[19] Stucchi M., Gmeiner P., Huebner H., Rainoldi G., Sacchetti A., Silvani A., Lesma G. (2015). Multicomponent Synthesis and Biological Evaluation of a Piperazine-Based Dopamine Receptor Ligand Library. ACS Med. Chem. Lett..

[20] Stucchi M., Cairati S., Cetin-Atalay R., Christodoulou M.S., Grazioso G., Pescitelli G., Silvani A., Yildirim D.C., Lesma G. (2015). Application of the Ugi reaction with multiple amino acid-derived components: Synthesis and conformational evaluation of piperazine-based minimalist peptidomimetics. Org. Biomol. Chem..

[21] Lesma G., Cecchi R., Crippa S., Giovanelli P., Meneghetti F., Musolino M., Sacchetti A., Silvani A. (2012). Ugi 4-CR/Pictet-Spengler reaction as a short route to tryptophan-derived peptidomimetics. Org. Biomol. Chem..

[22] Silvani A., Lesma G., Crippa S., Vece V. (2014). Multicomponent access to novel dihydroimidazo[1′,5′:1,2]pyrido[3,4-b]indol-2-ium salts and indoles by means of Ugi/Bischler–Napieralski/heterocyclization two step strategy. Tetrahedron.

[23] Lesma G., Bassanini I., Bortolozzi R., Colletto C., Bai R., Hamel E., Meneghetti F., Rainoldi G., Stucchi M., Sacchetti A. (2015). Complementary isonitrile-based multicomponent reactions for the synthesis of diversified cytotoxic hemiasterlin analogues. Org. Biomol. Chem..

[24] Lawrence K., Flower S.E., Kociok-Kohn G., Frost C.G., James T.D. (2012). A simple and effective colorimetric technique for the detection of boronic acids and their derivatives. Anal. Meth..

[25] Janvier P., Sun X., Bienayme H., Zhu J. (2002). Ammonium chloride-promoted four-component synthesis of pyrrolo[3,4-b]pyridin-5-one. J. Am. Chem. Soc..

[26] CCDC 1995920 Contains the Supplementary Crystallographic Data for This Paper. The Cambridge Crystallographic Data Centre. www.ccdc.cam.ac.uk/structures.

[27] Knapp D.M., Gillis E.P., Burke M.D. (2009). A General Solution for Unstable Boronic Acids: Slow-Release Cross-Coupling from Air Stable MIDA Boronates. J. Am. Chem. Soc..

[28] CCDC 1995919 Contains the Supplementary Crystallographic Data for This Paper. The Cambridge Crystallographic Data Centre. www.ccdc.cam.ac.uk/structures.

[29] (2020). Schrödinger Release 2019-4.

[30] Bowers K.J., Chow D.E., Xu H., Dror R.O., Eastwood M.P., Gregersen B.A., Klepeis J.L., Kolossvary I., Moraes A., Sacerdoti D. (2006). Scalable Algorithms for Molecular Dynamics Simulations on Commodity Clusters. Proc. ACM/IEEE Conf. Supercomput..

[31] Bruker S. (2008). ADABS.

[32] Sheldrick G.M. (2015). SHELXT—Integrated Space-group and Crystal-structure Determination. Acta Cryst..

[33] Borgianni L., Vandenameele J., Matagne A., Bini L., Bonomo R.A., Frere J.M., Rossolini G.M., Docquier J.D. (2010). Mutational analysis of VIM-2 reveals an essential determinant for metallo-beta-lactamase stability and folding. Antimicrob. Agents Chemother..

[34] Weinstein M.P. (2015). MD M07-Methods for Dilution Antimicrobial Susceptibility Tests for Bacteria That Grow Aerobicall.

[35] Smith C.A., Antunes N.T., Stewart N.K., Toth M., Kumarasiri M., Chang M., Mobashery S., Vakulenko S.B. (2013). Structural basis for carbapenemase activity of the OXA-23 beta-lactamase from Acinetobacter baumannii. Chem. Biol..

[36] Wang X., Minasov G., Shoichet B.K. (2002). Evolution of an antibiotic resistance enzyme constrained by stability and activity trade-offs. J. Mol. Biol..

[37] Sgrignani J., Grazioso G., De Amici M. (2016). Insight into the Mechanism of Hydrolysis of Meropenem by OXA-23 Serine-beta-lactamase Gained by Quantum Mechanics/Molecular Mechanics Calculations. Biochemistry.

[38] Jorgensen W.L., Chandrasekhar J., Madura J.D., Impey R.W., Klein M.L. (1983). Comparison of simple potential functions for simulating liquid water. J. Chem. Phys..

[39] Roos K., Wu C., Damm W., Reboul M., Stevenson J.M., Lu C., Dahlgren M.K., Mondal S., Chen W., Wang L. (2019). OPLS3e: Extending Force Field Coverage for Drug-Like Small Molecules. J. Chem. Theory Comput..

[40] Humphrey W., Dalke A., Schulten K. (1996). VMD: Visual molecular dynamics. J. Mol. Graph..

[41] Friesner R.A., Murphy R.B., Repasky M.P., Frye L.L., Greenwood J.R., Halgren T.A., Sanschagrin P.C., Mainz D.T. (2006). Extra precision glide: Docking and scoring incorporating a model of hydrophobic enclosure for protein-ligand complexes. J. Med. Chem..

