# Supplementary material for: Isonitrile-Based Multicomponent Synthesis of β-Amino Boronic Acids as β-Lactamase Inhibitors
Source: Antibiotics (Basel). 2020 May 12;9(5):249. doi: 10.3390/antibiotics9050249 (PMC7277116; doi:10.3390/antibiotics9050249)
Supplement: Supplementary file 1 [file antibiotics-09-00249-s001.pdf]

Supporting information

for

Isonitrile-based Multicomponent Synthesis of β-Amino Boronic Acids as β-lactamase Inhibitors

Emanuele Bassini,[a] Stefano Gazzotti,[a] Filomena Sannio,[b] Leonardo Lo Presti,[a] Jacopo Sgrignani,[c] Jean-Denis Docquier,[b] Giovanni Grazioso[d] and Alessandra Silvani[a,]*

[a]*Dipartimento di Chimica, Università degli Studi di Milano, Via Golgi 19, 20133 Milan, Italy.* [b]*Dipartimento di Biotecnologie Mediche, Università degli Studi di Siena, Viale Bracci 16, 53100 Siena, Italy.* [c]*Istituto di Ricerca in Biomedicina (IRB), Università della Svizzera Italiana (USI), Via V. Vela 6, CH-6500 Bellinzona, Switzerland.* [d]*Dipartimento di Scienze Farmaceutiche, Università degli Studi di Milano, Via L. Mangiagalli 25, 20133 Milan, Italy*

Table of contents

1. ^{1}H and ^{13}C NMR spectra of MIDA-protected Ugi-4CR products (**5a-p**)………………………..S2-S17
2. ^{1}H and ^{13}C NMR spectra of Ugi-4CR products (**6a-p**)………………………………………….…….S18-S33
3. ^{1}H and ^{13}C NMR spectra of MIDA-protected Ugi-4C-3CR products (**8a-c**)…………………S34-S36
4. ^{1}H and ^{13}C NMR spectra of Ugi-4C-3CR products (**9a-c**)…………………………………….…..S37-S39
5. ^{1}H and ^{13}C NMR spectra of Van Leusen-3CR product (**10**)………………………………………….S40
6. ^{11}B NMR spectra of Ugi-4CR products (**6a-p**)………………………………………………………..S41-S48
7. ^{11}B NMR spectra of Ugi-4C-3CR products (**9a-c**)…………………………………………………….S49-S50
8. Single crystal X-ray diffraction analysis of compound **5a** and **6d**………………………………..S51-S56

1. ^{1}H and ^{13}C NMR spectra of MIDA-protected Ugi-4CR products (5a-p)

^{1}H NMR (400 MHz, CD_3OD) of compound **5a**

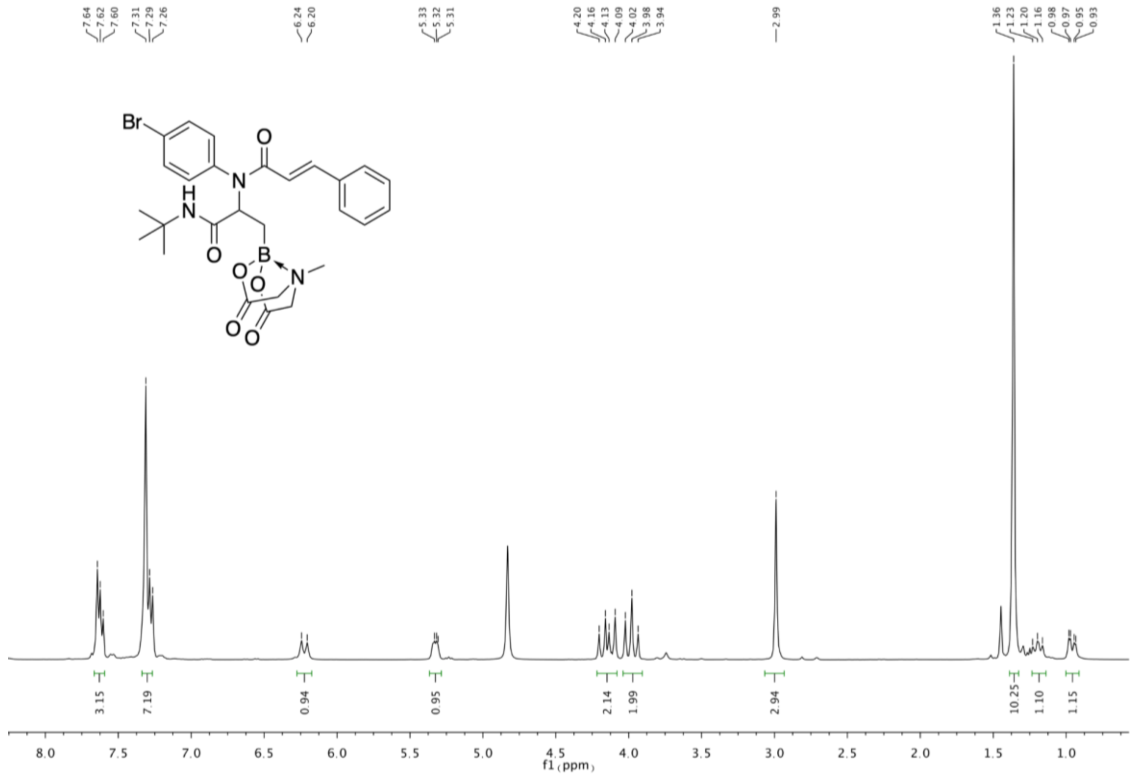

^{13}C NMR (101 MHz, CD_3OD) of compound **5a**

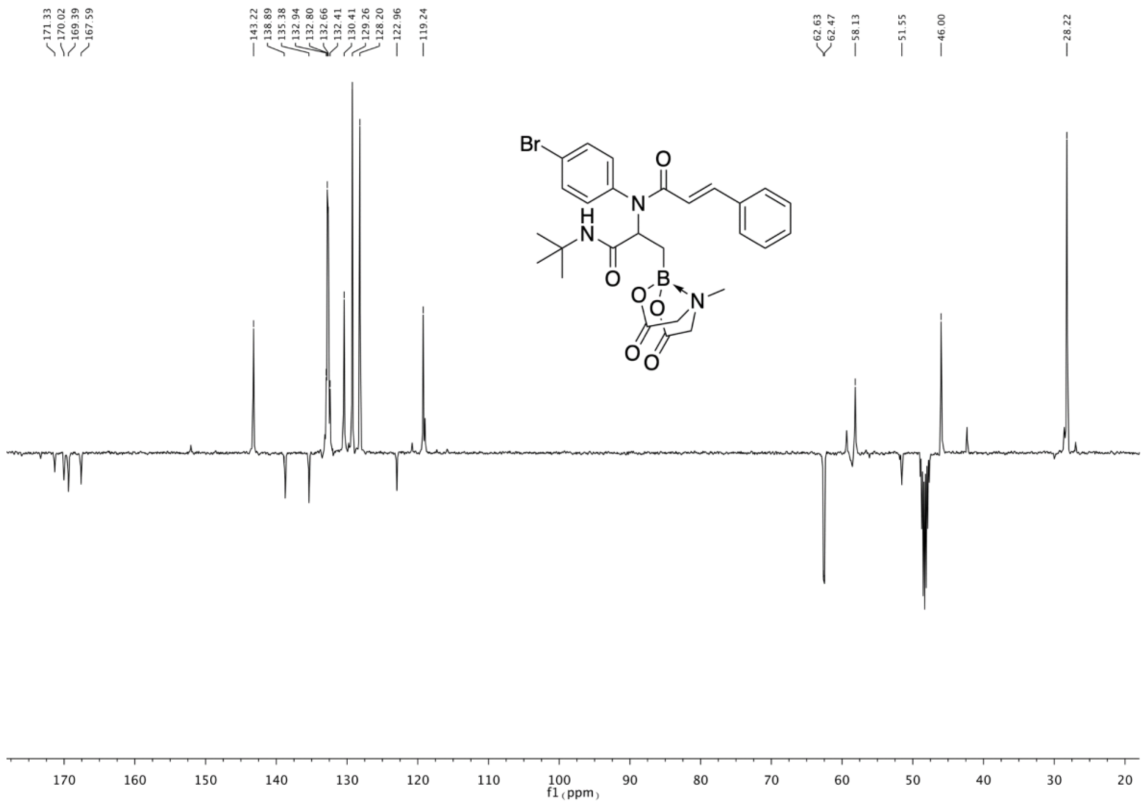

^{1}H NMR (400 MHz, 115°C, DMSO) of compound **5b**

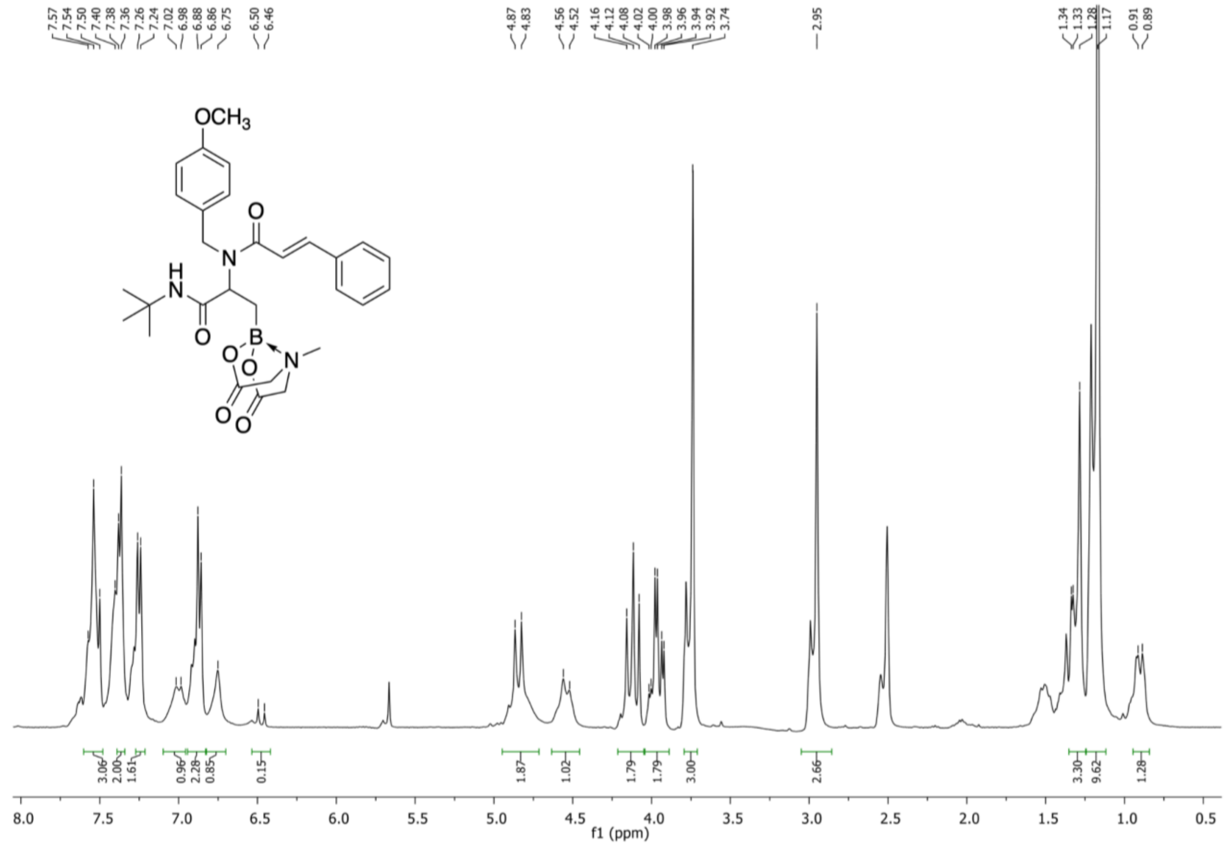

^{13}C NMR (101 MHz, 25°C, DMSO, 6:4 rotameric mixture) of compound **5b**

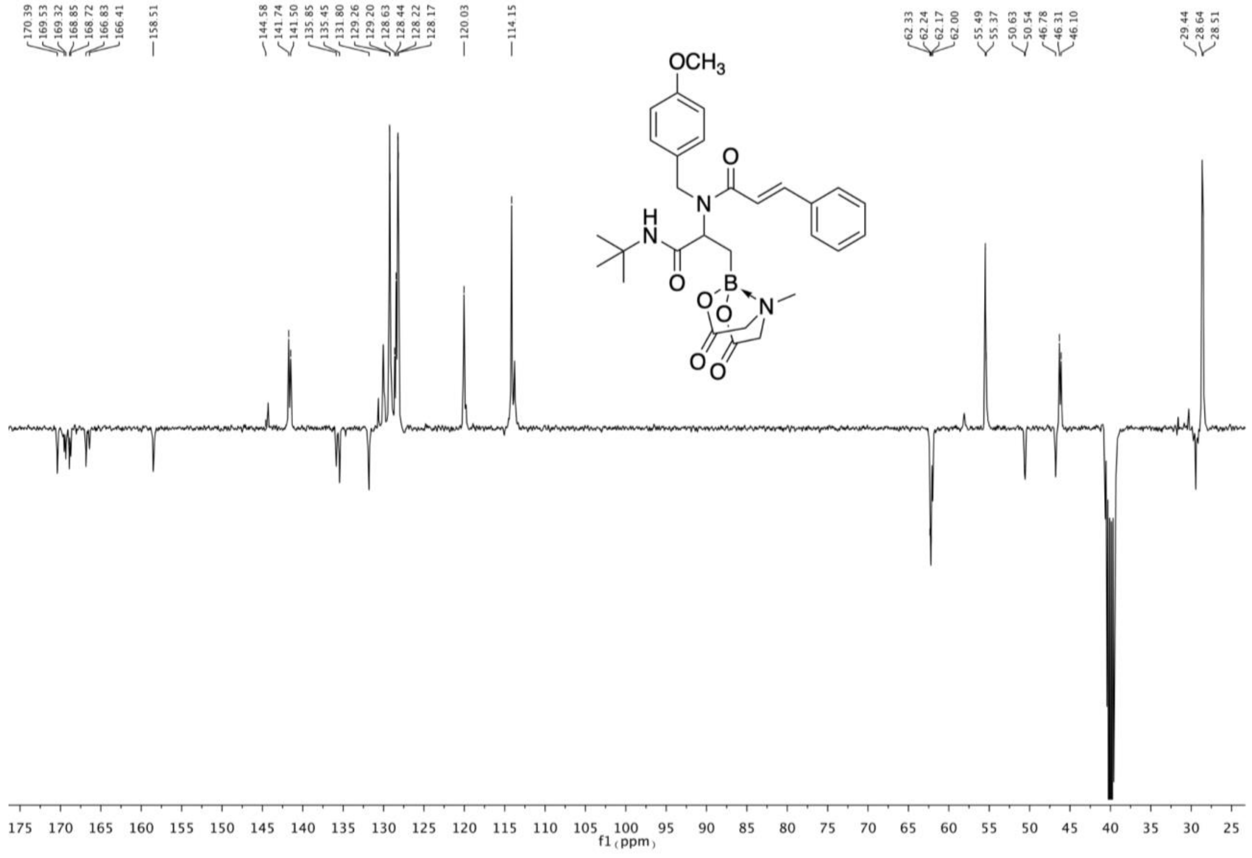

^{1}H NMR (400 MHz, CD_3OD) of compound **5c**

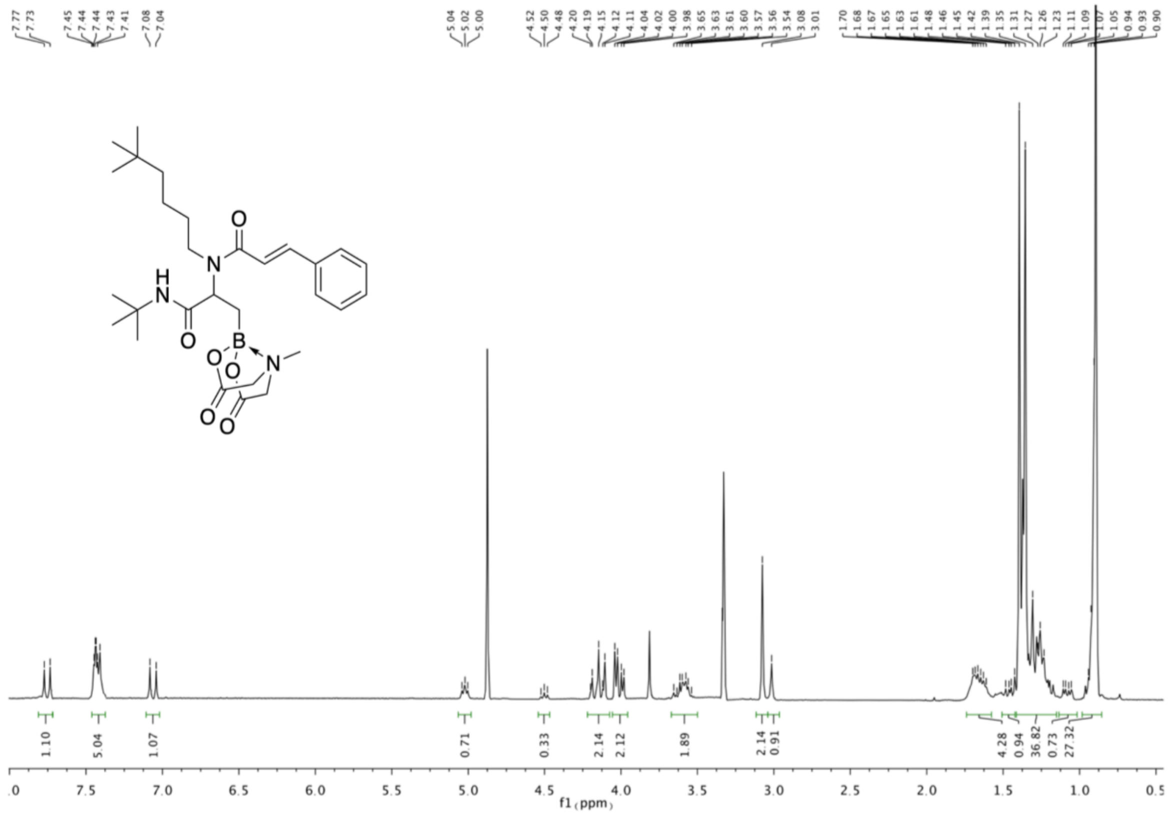

^{13}C NMR (101 MHz, CD_3OD) of compound **5c**

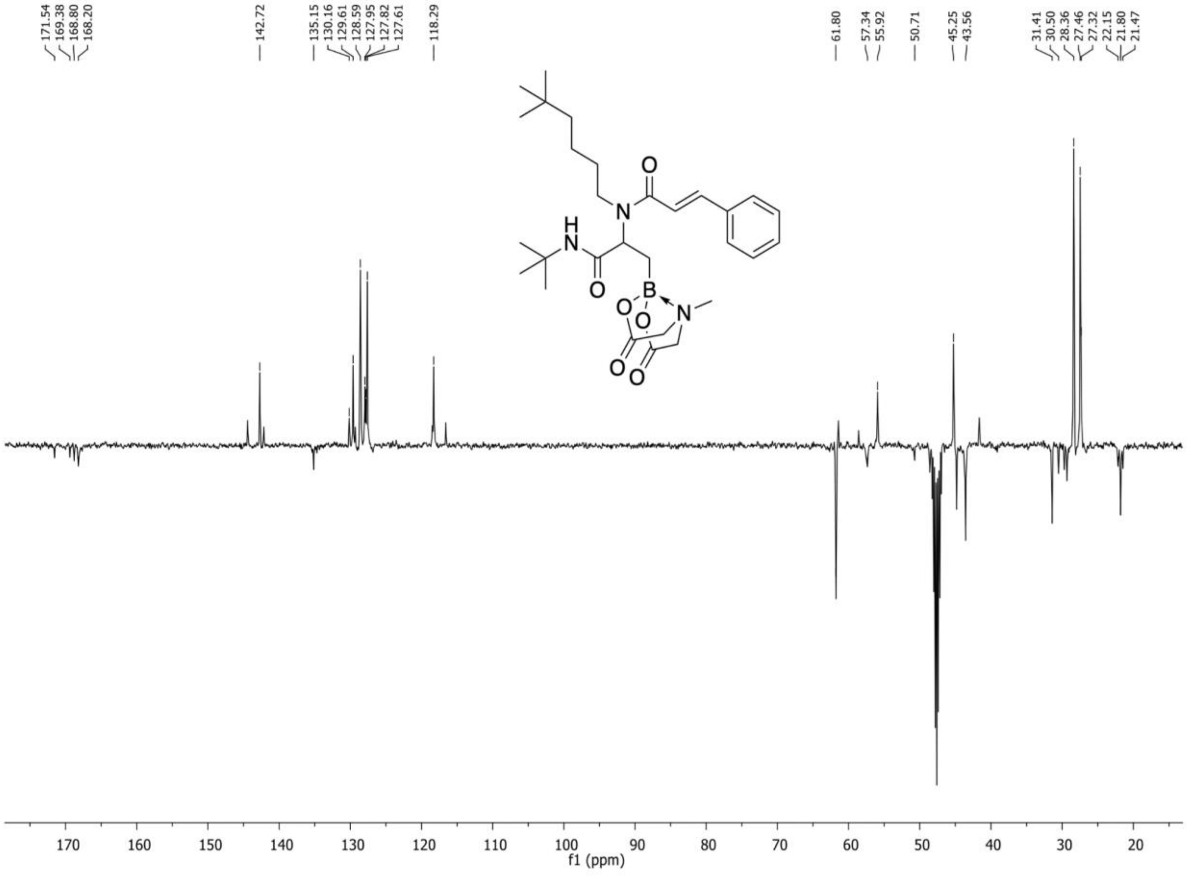

^{1}H NMR (400 MHz, CD_3OD) of compound **5d**

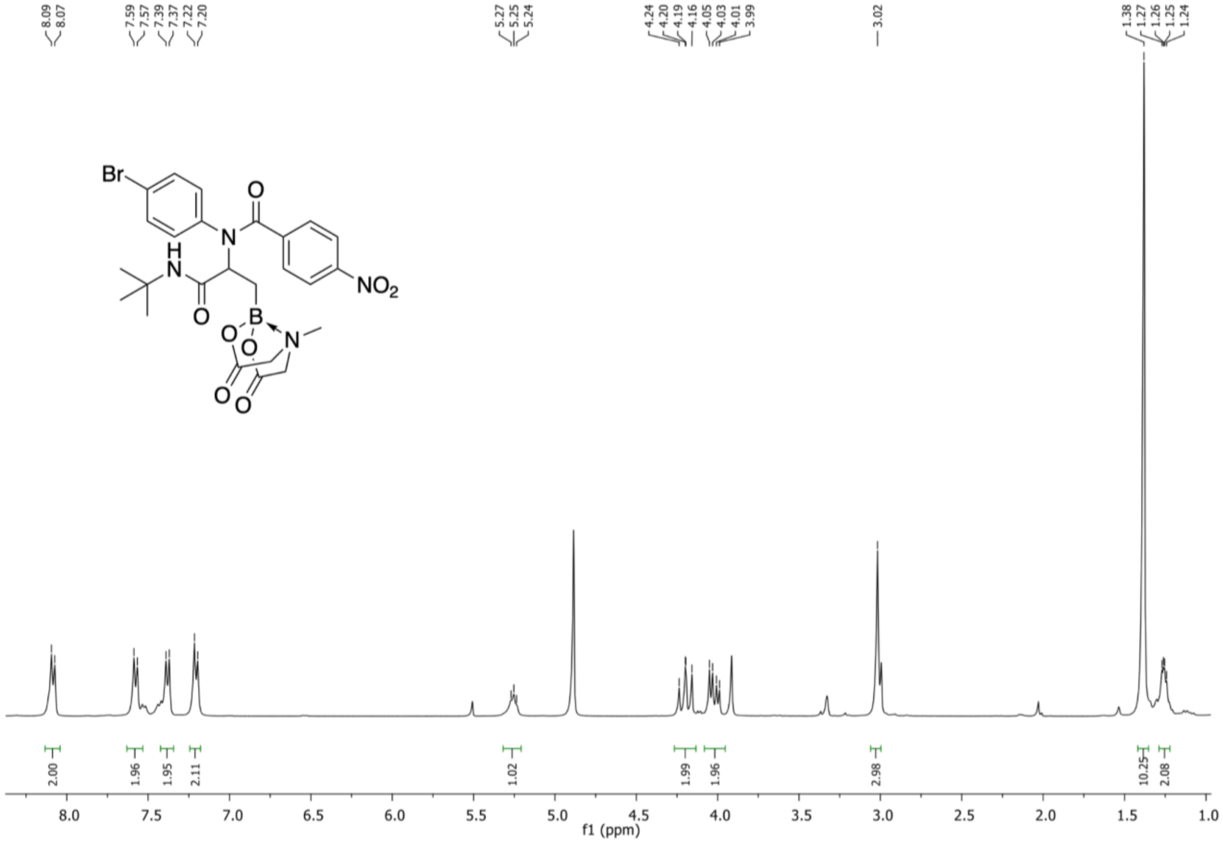

^{13}C NMR (75 MHz, CD_3OD, 2:1 rotameric mixture) of compound **5d**

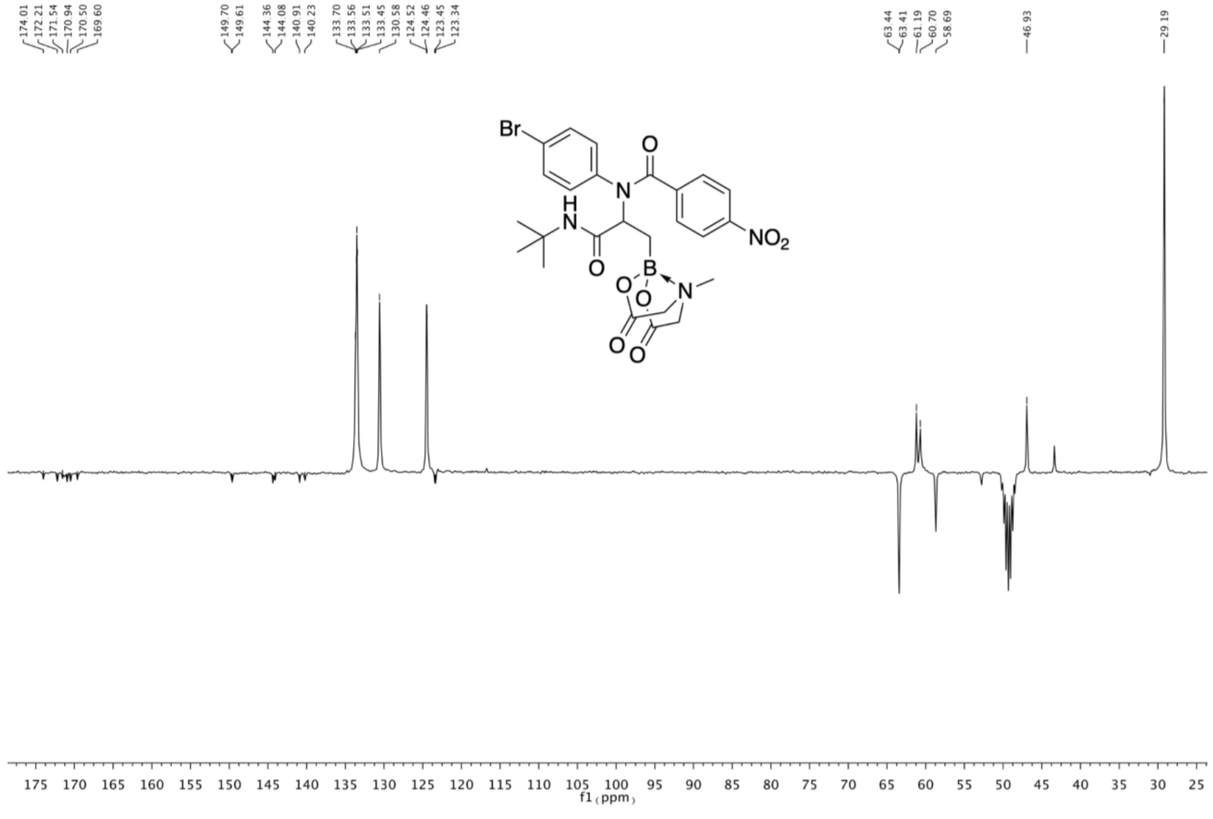

^{1}H NMR (400 MHz, CD_3OD) of compound **5e**

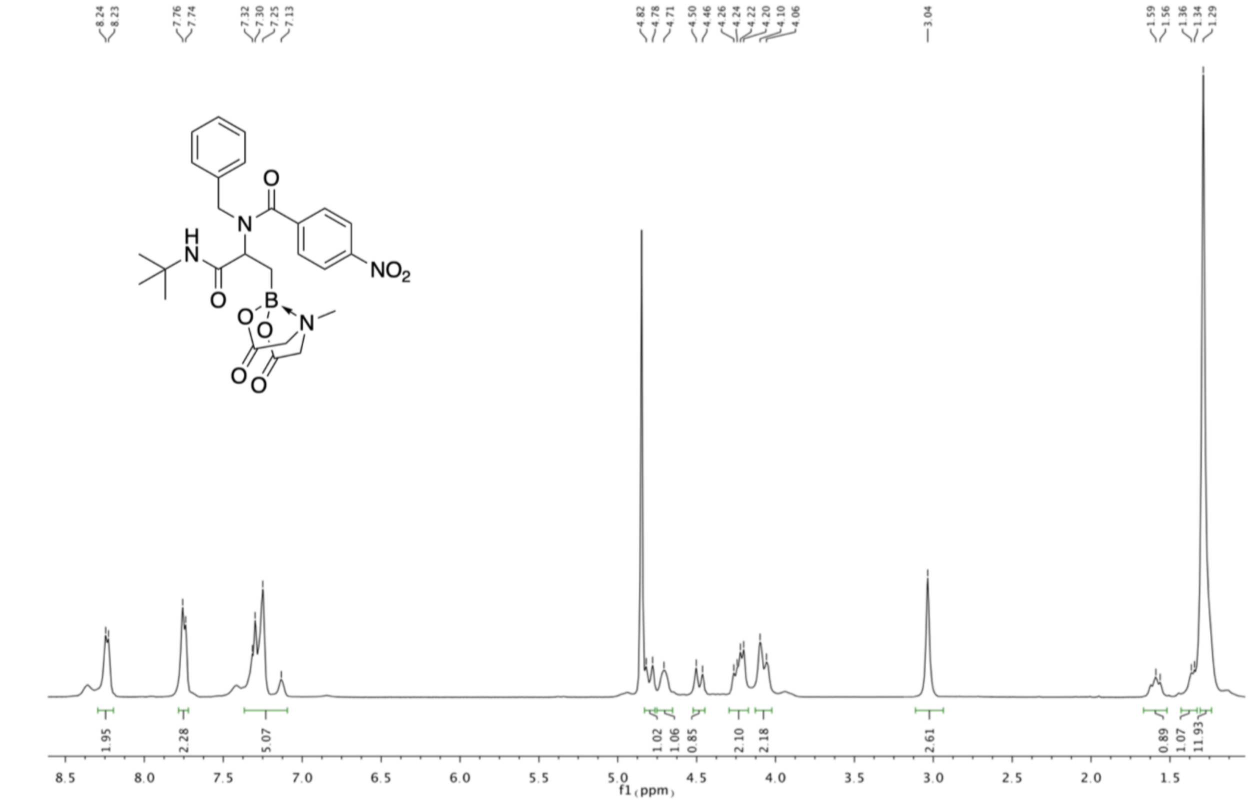

^{13}C NMR (101 MHz, CD_3OD) of compound **5e**

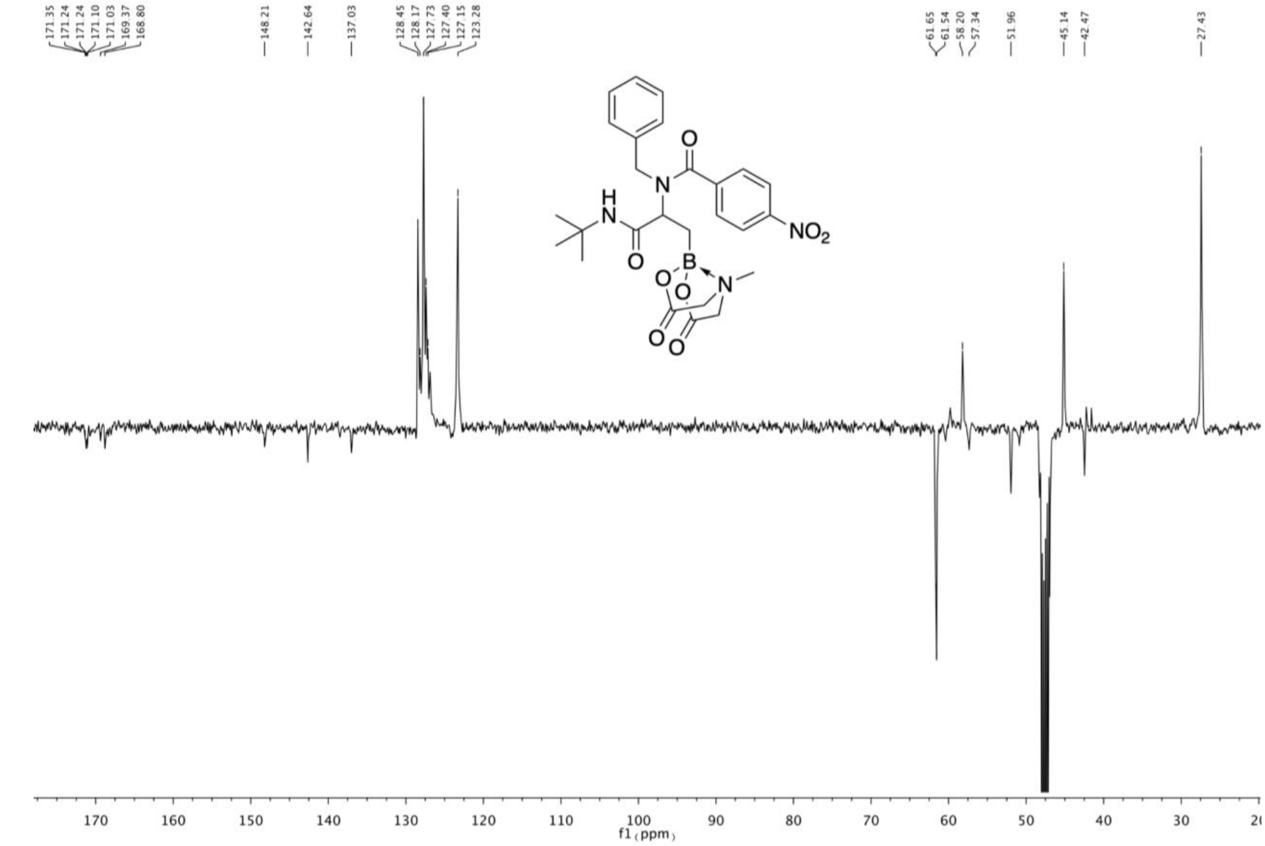

^{1}H NMR (400 MHz, CD_3OD) of compound **5f**

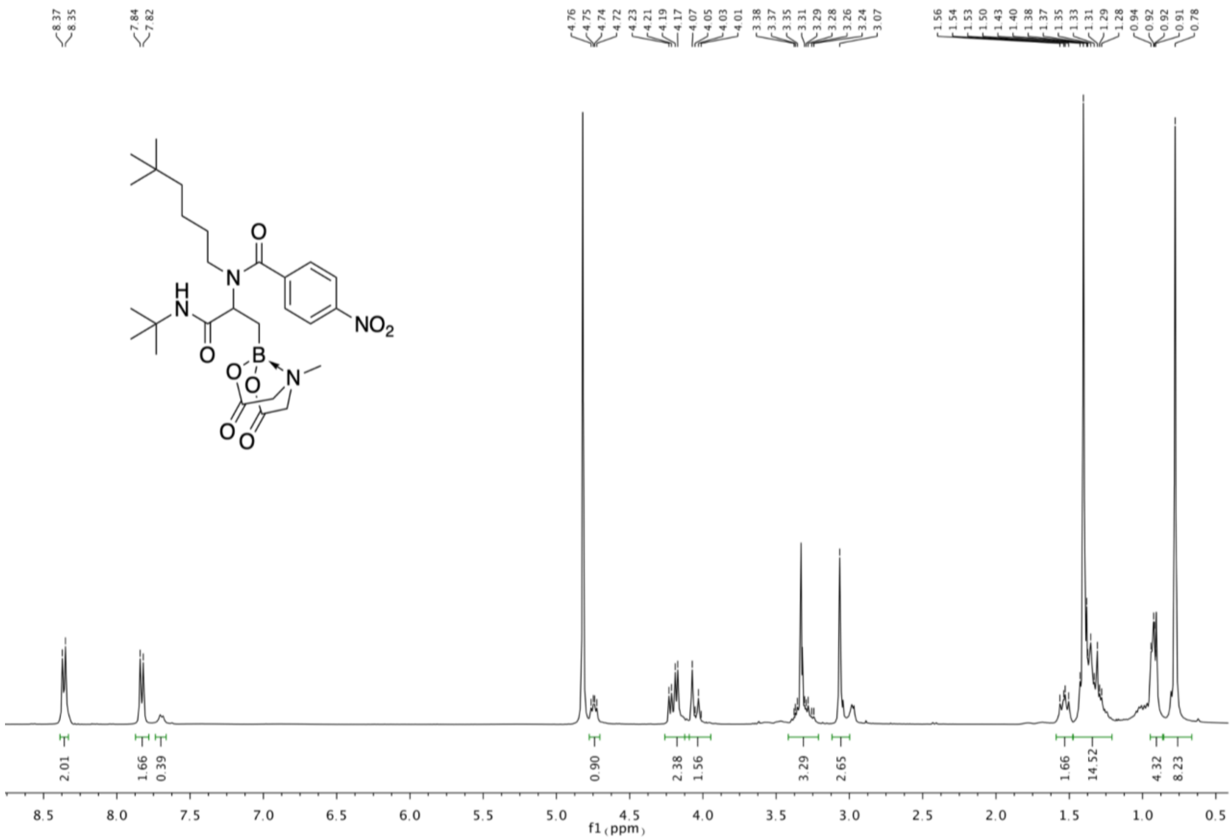

^{13}C NMR (101 MHz, CD_3OD) of compound **5f**

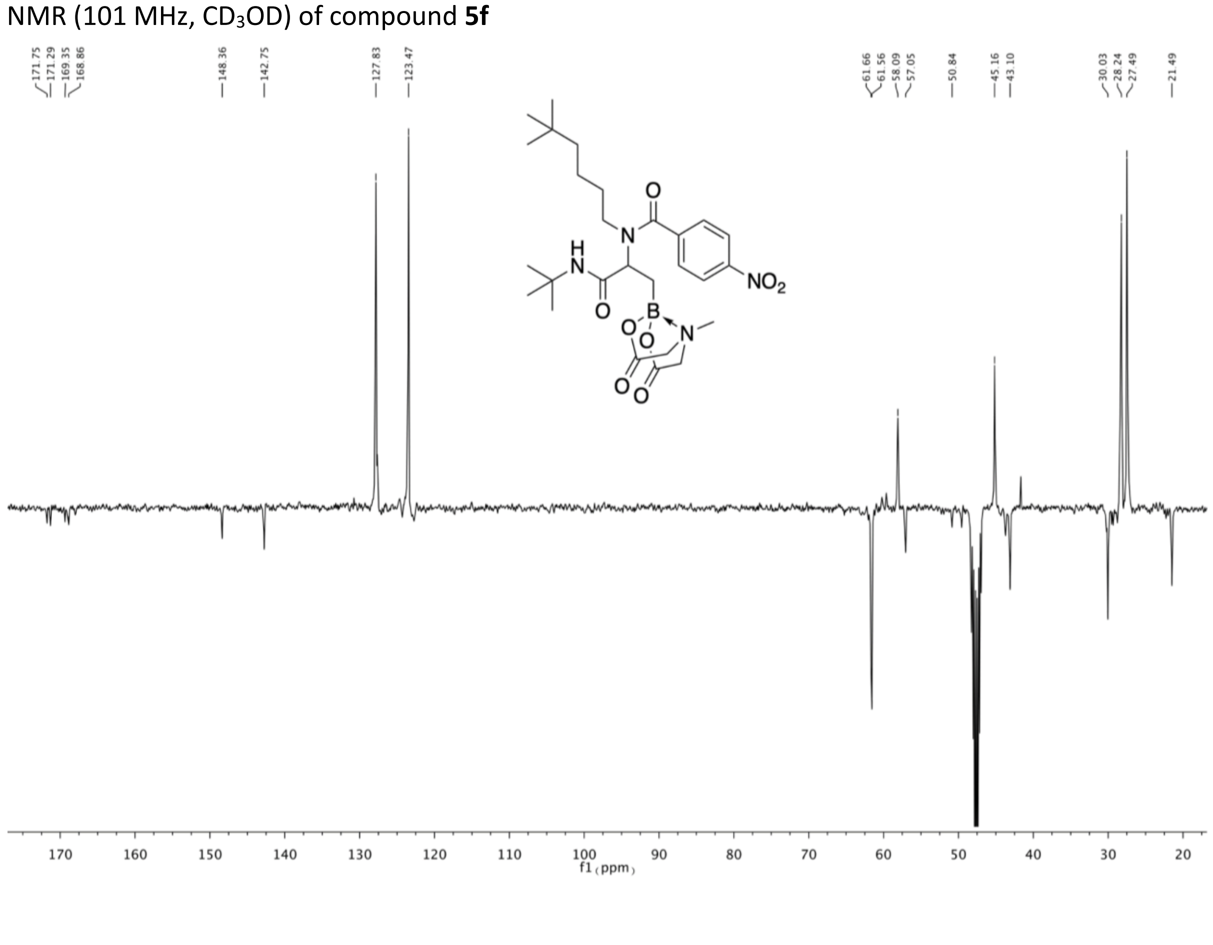

^{1}H NMR (400 MHz, CD_3OD) of compound **5g**

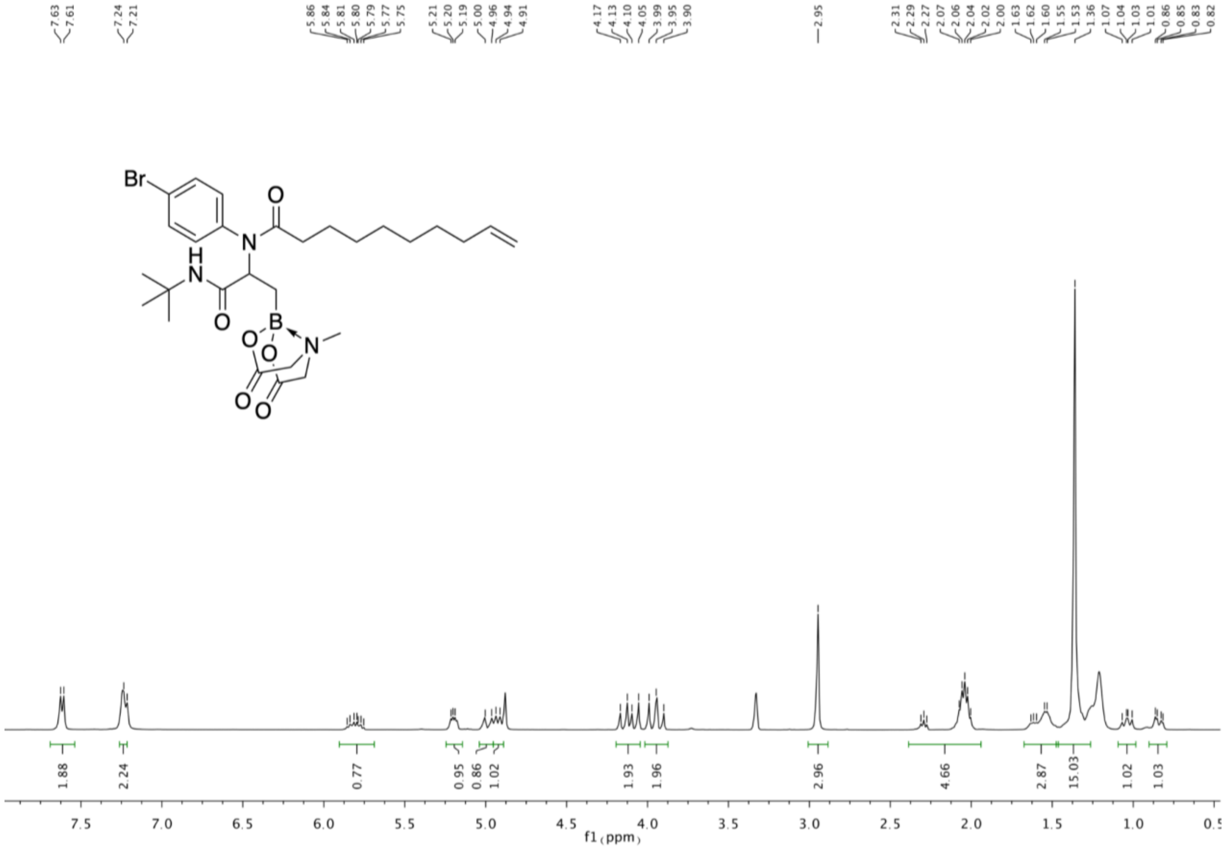

^{13}C NMR (75 MHz, CD_3OD) of compound **5g**

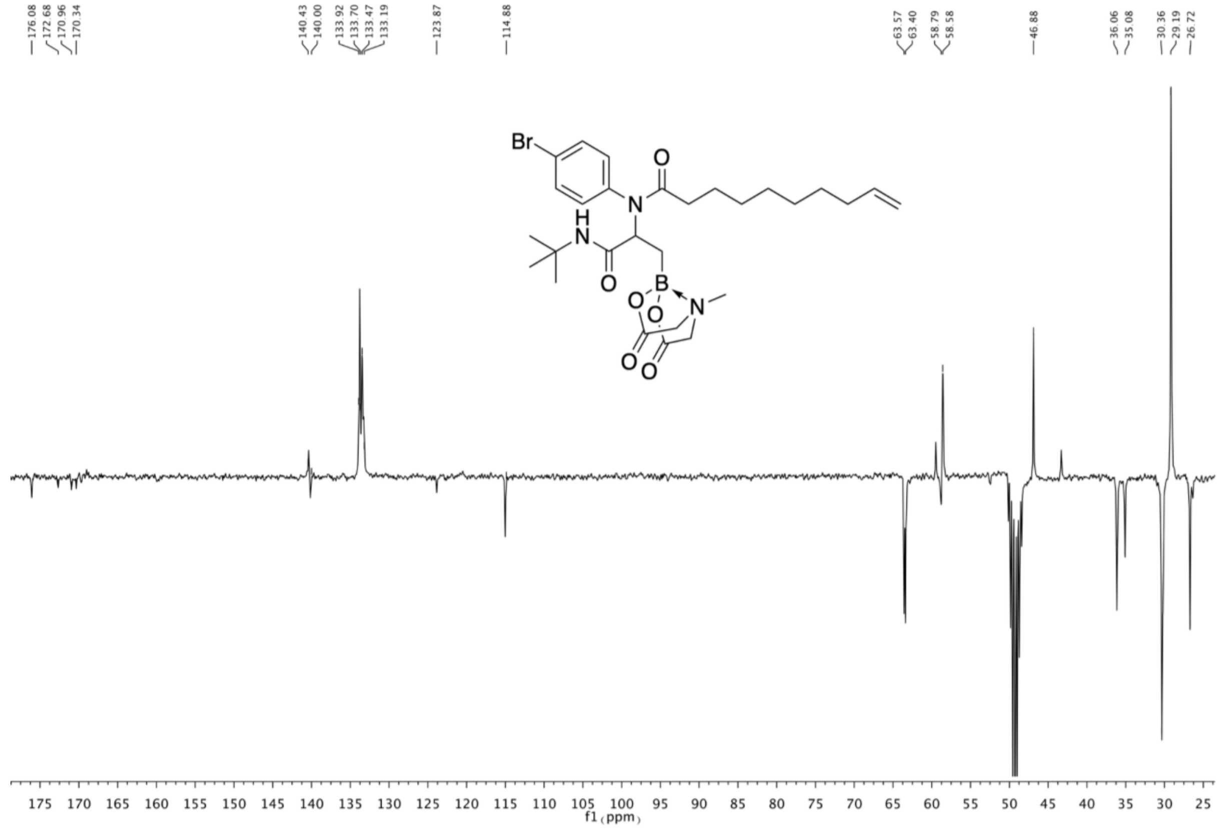

^{1}H NMR (400 MHz, 115°C, DMSO) of compound **5h**

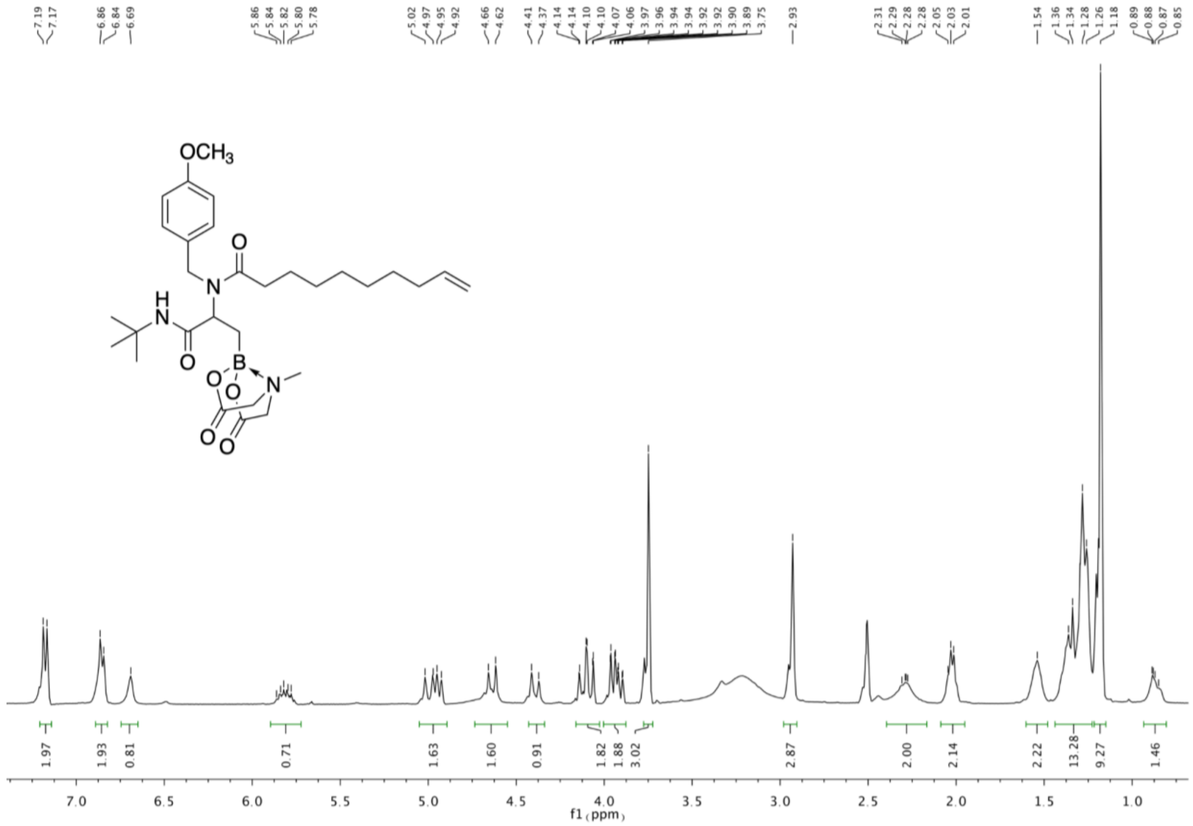

^{13}C NMR (101 MHz, 25°C, DMSO, 1:1 rotameric mixture) of compound **5h**

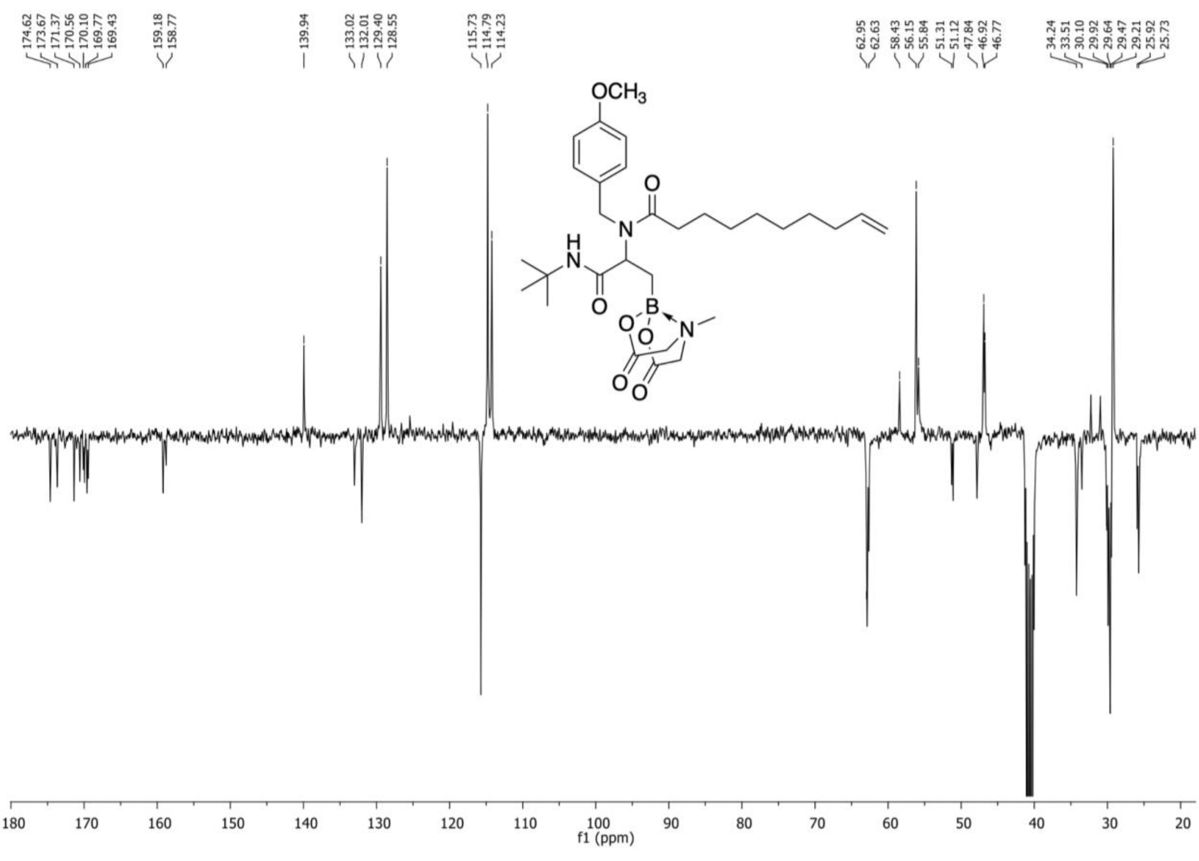

^{1}H NMR (400 MHz, DMSO, 6:4 rotameric mixture) of compound **5i**

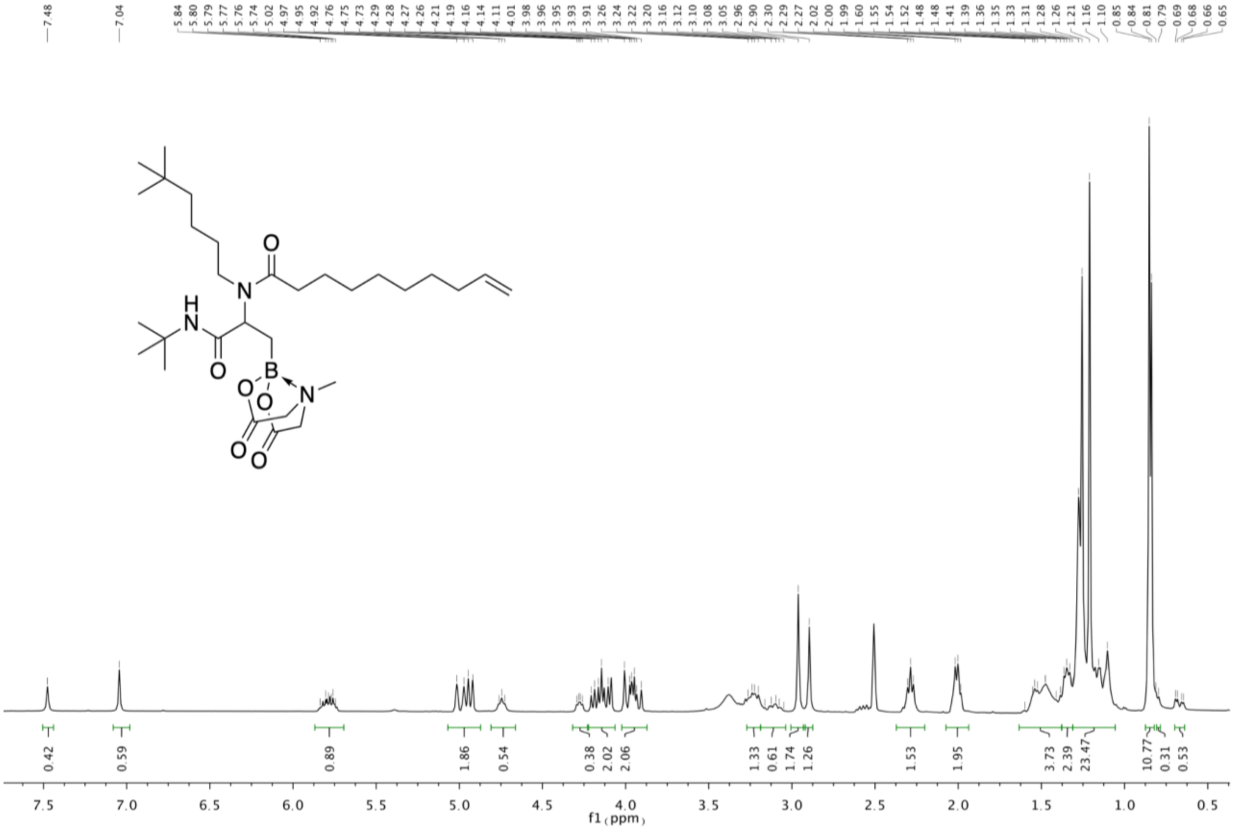

^{13}C NMR (101 MHz, DMSO, 6:4 rotameric mixture) of compound **5i**

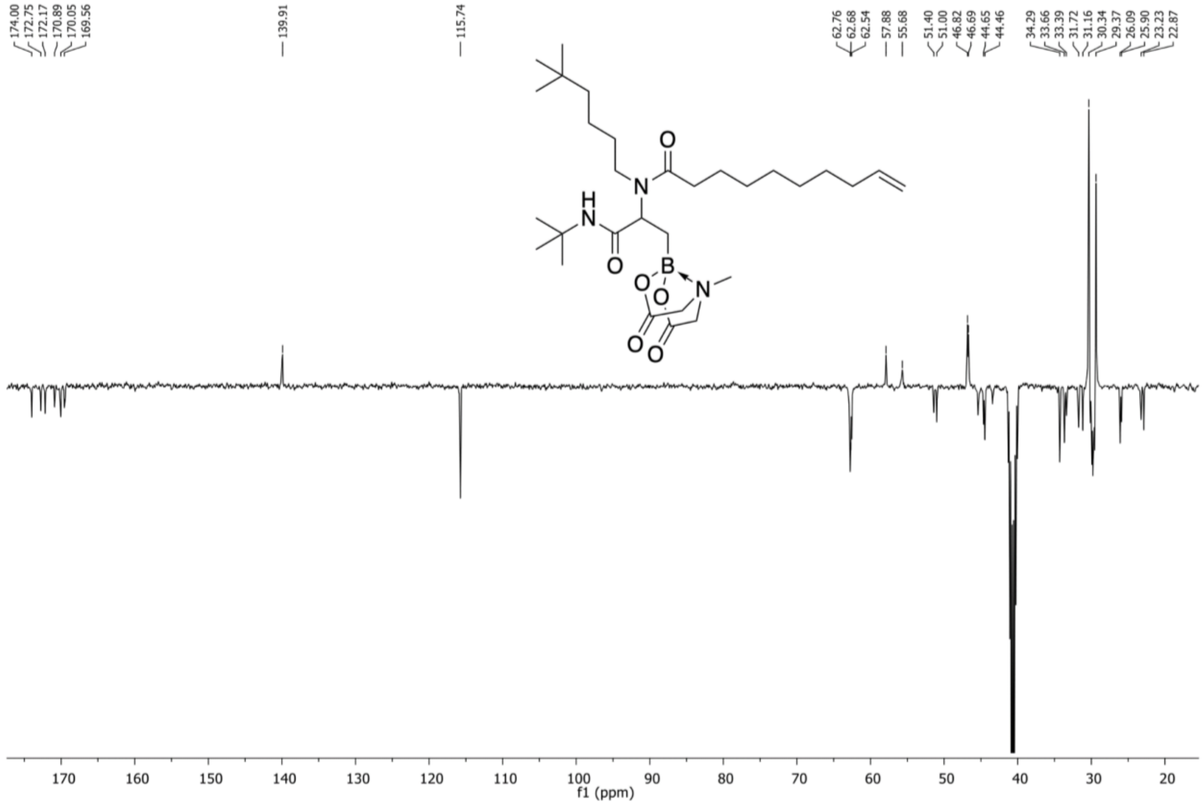

^{1}H NMR (400 MHz, CD$_3$OD) of compound **5j**

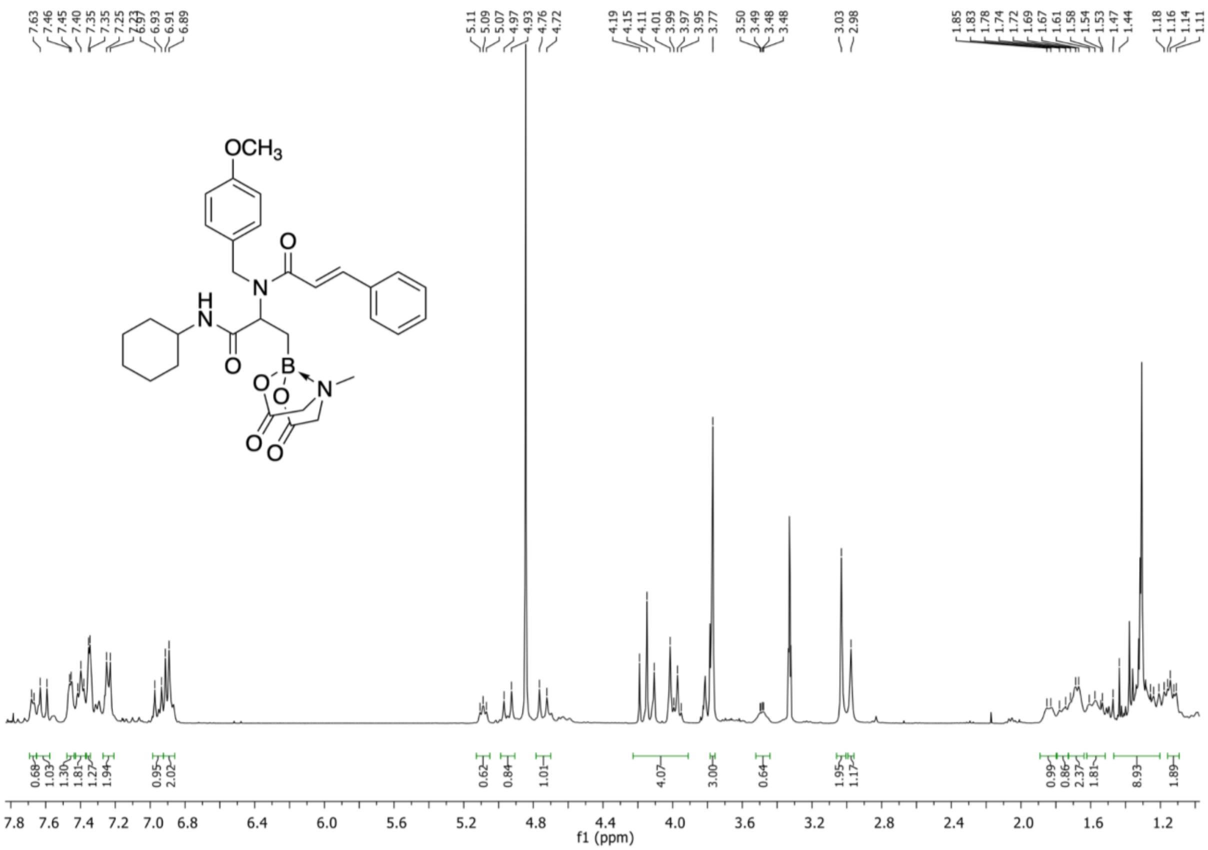

^{13}C NMR (75 MHz, CD$_3$OD) of compound **5j**

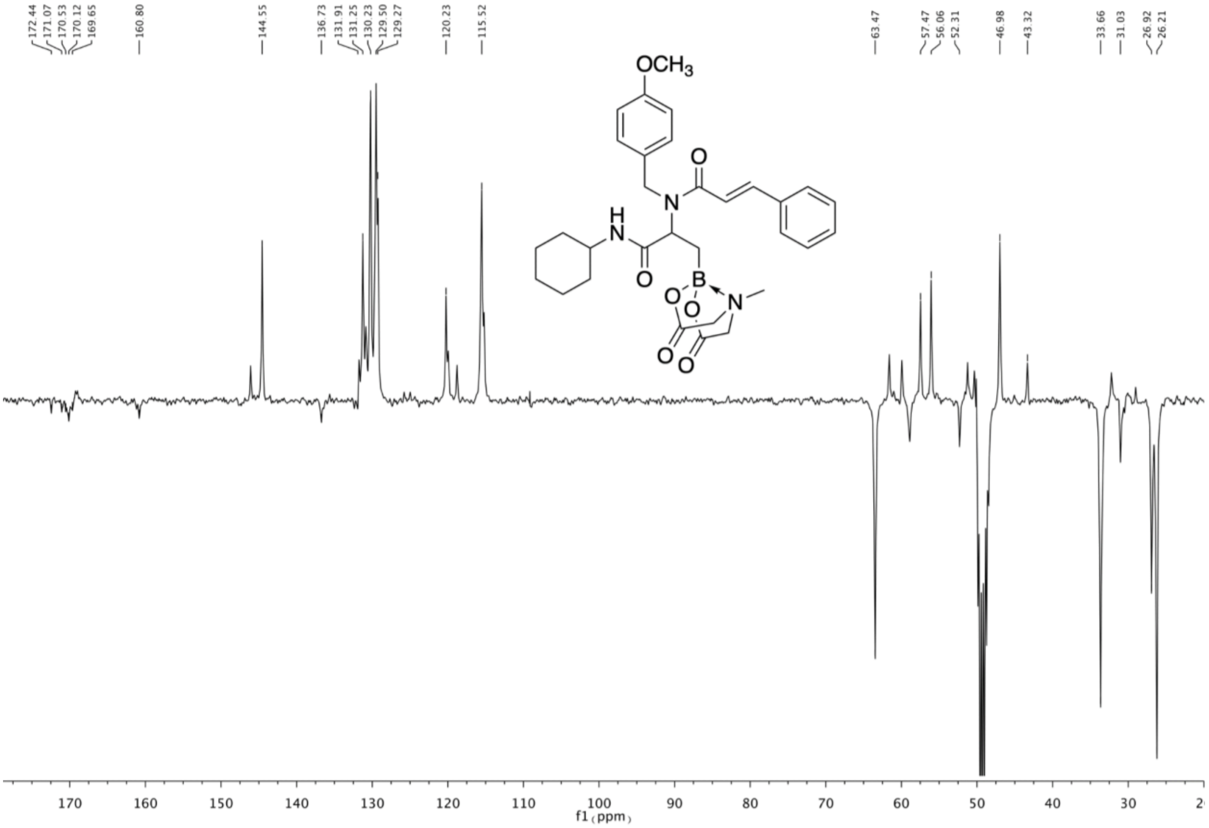

^{1}H NMR (400 MHz, CD_3OD, 6:4 rotameric mixture) of compound **5k**

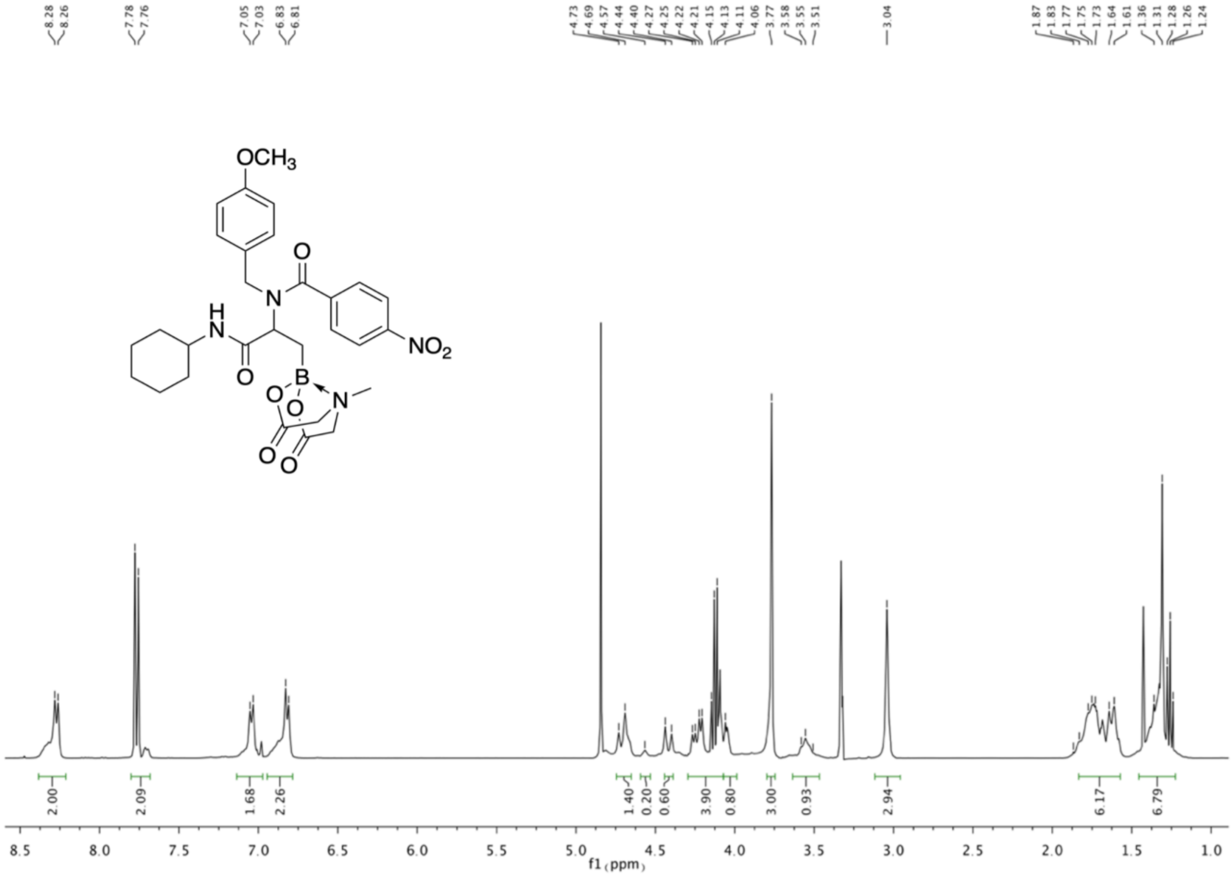

^{13}C NMR (101 MHz, CD_3OD) of compound **5k**

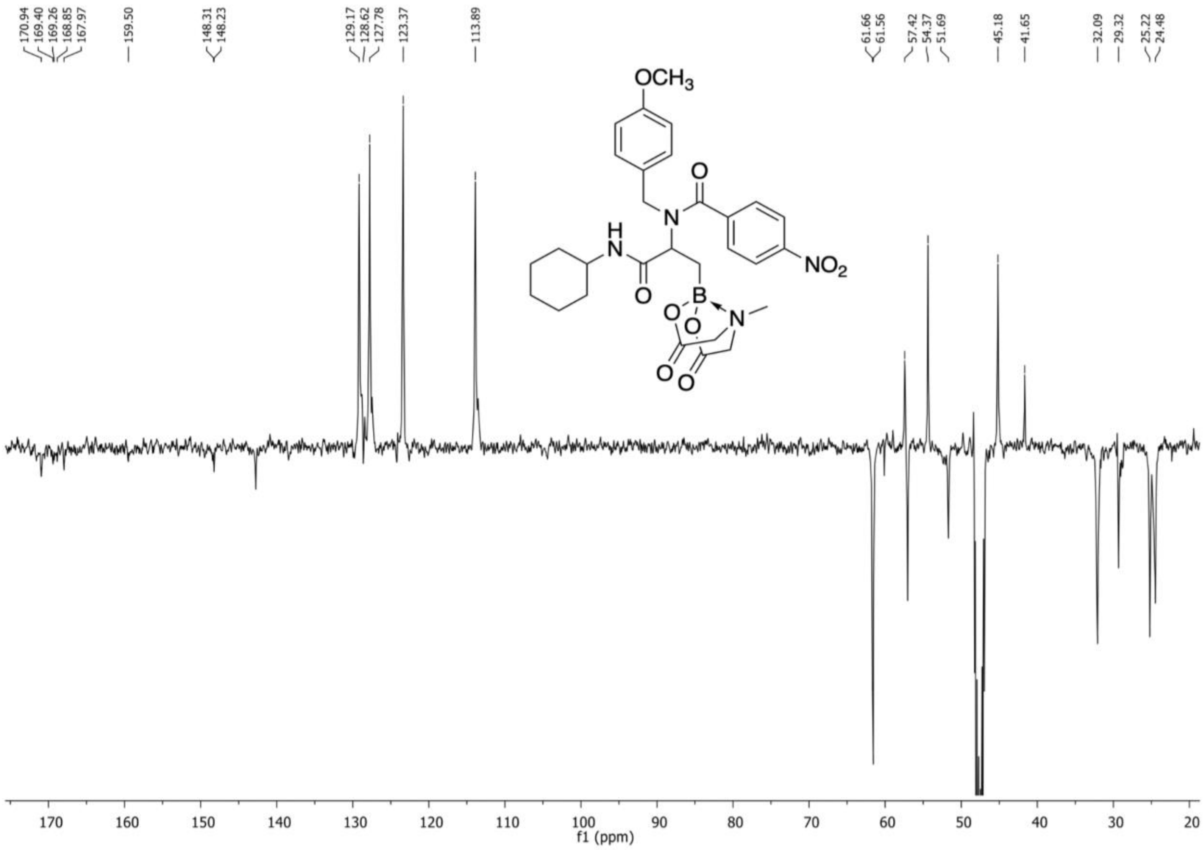

^{1}H NMR (400 MHz, CD_3OD, rotameric mixture) of compound **5l**

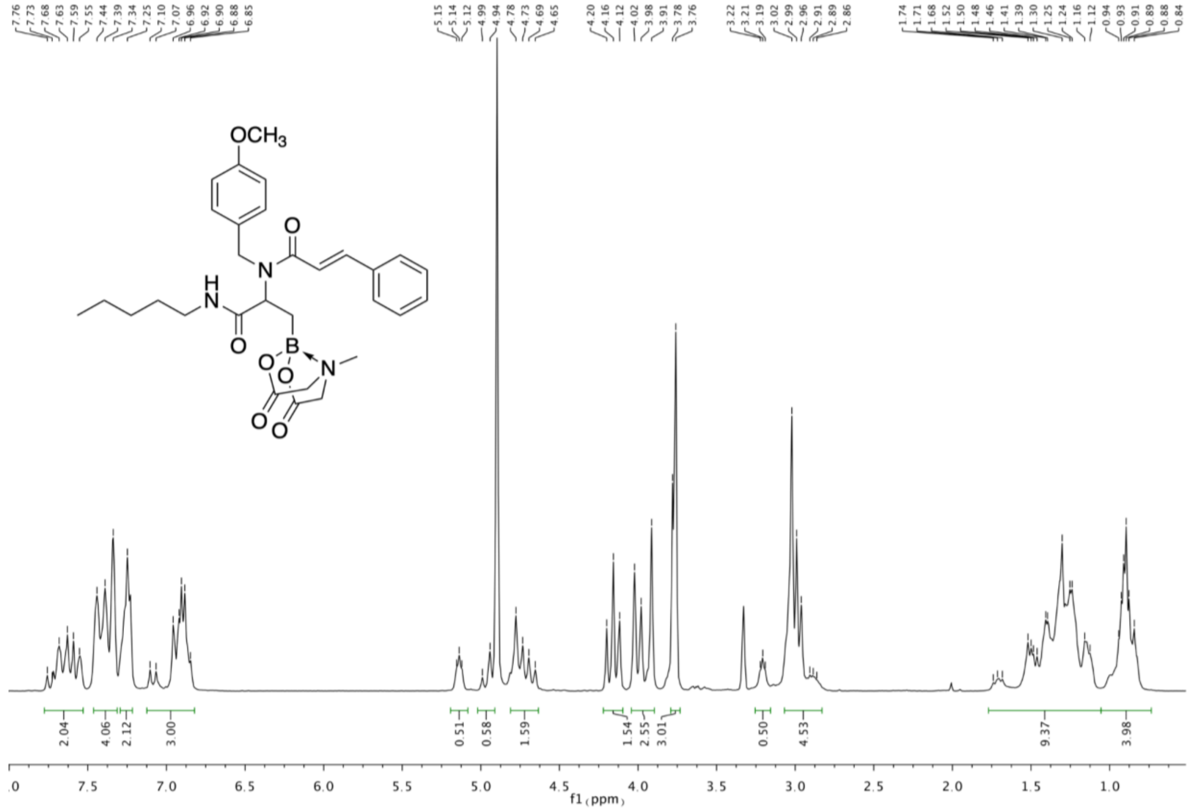

^{13}C NMR (101 MHz, CD_3OD, rotameric mixture) of compound **5l**

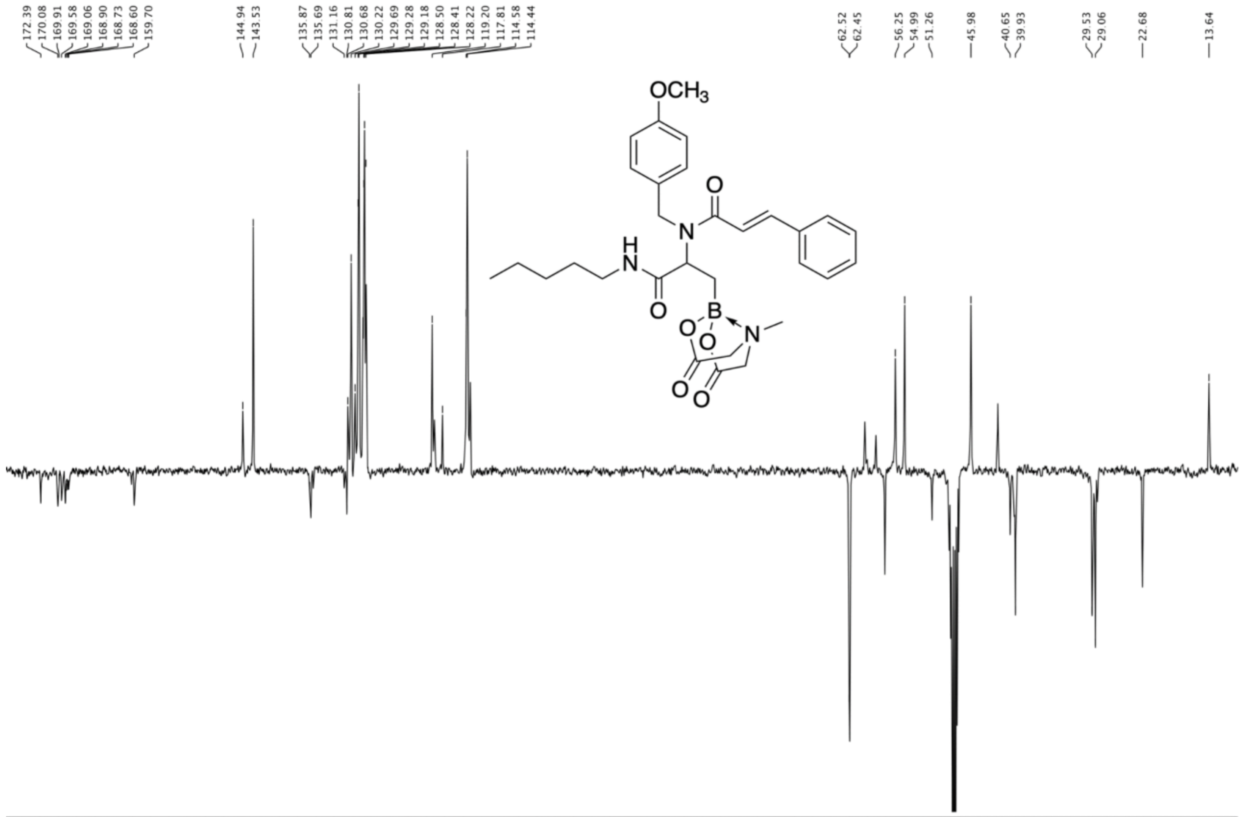

^{1}H NMR (400 MHz, 115°C, DMSO, 9:1 rotameric mixture) of compound **5m**

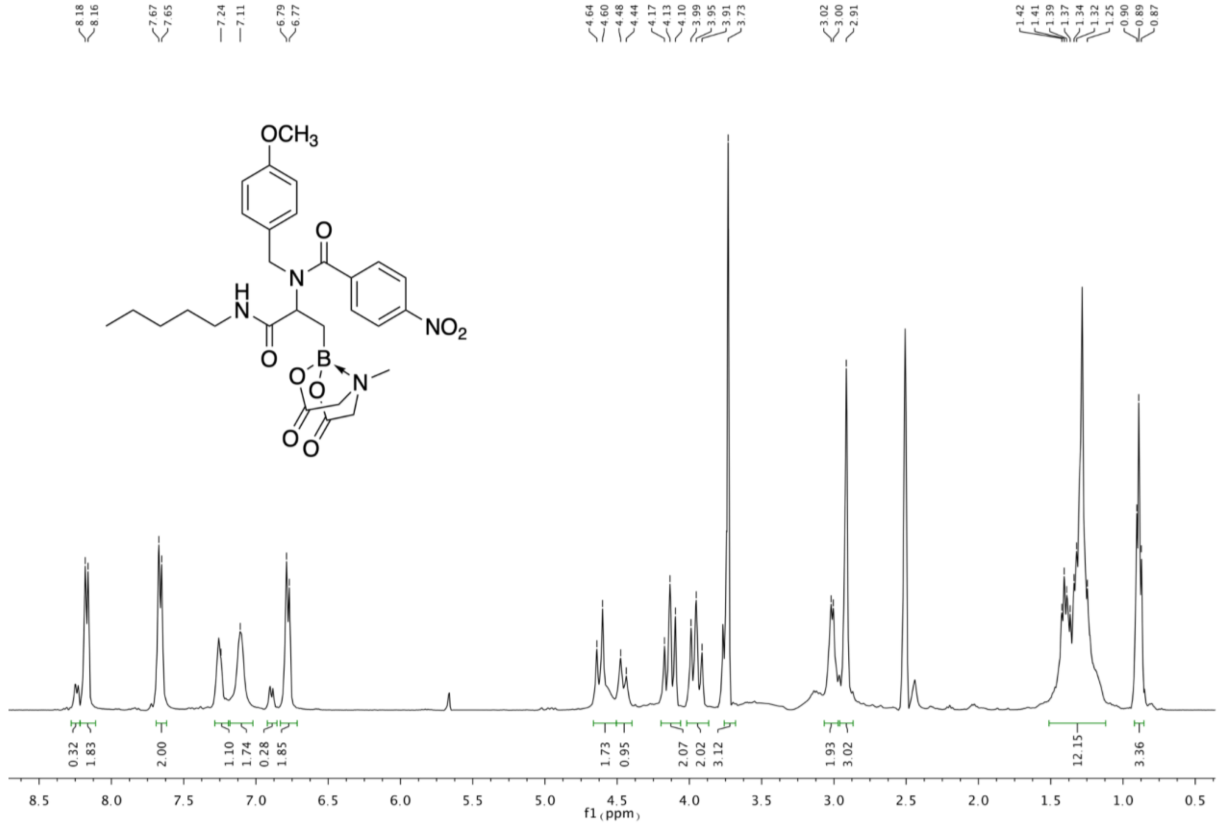

^{13}C NMR (101 MHz, 25°C, DMSO, 7:3 rotameric mixture) of compound **5m**

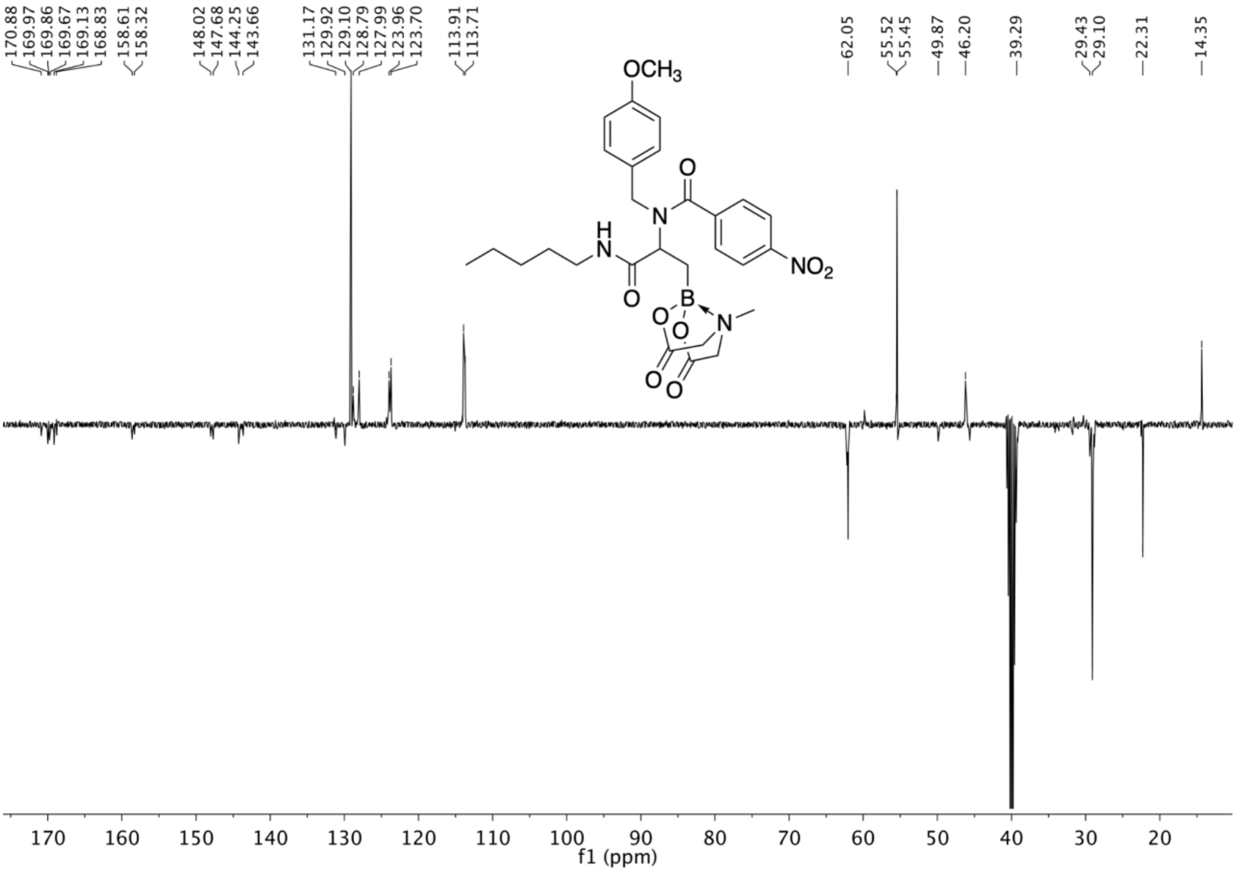

^{1}H NMR (400 MHz, CD_3OD) of compound **5n**

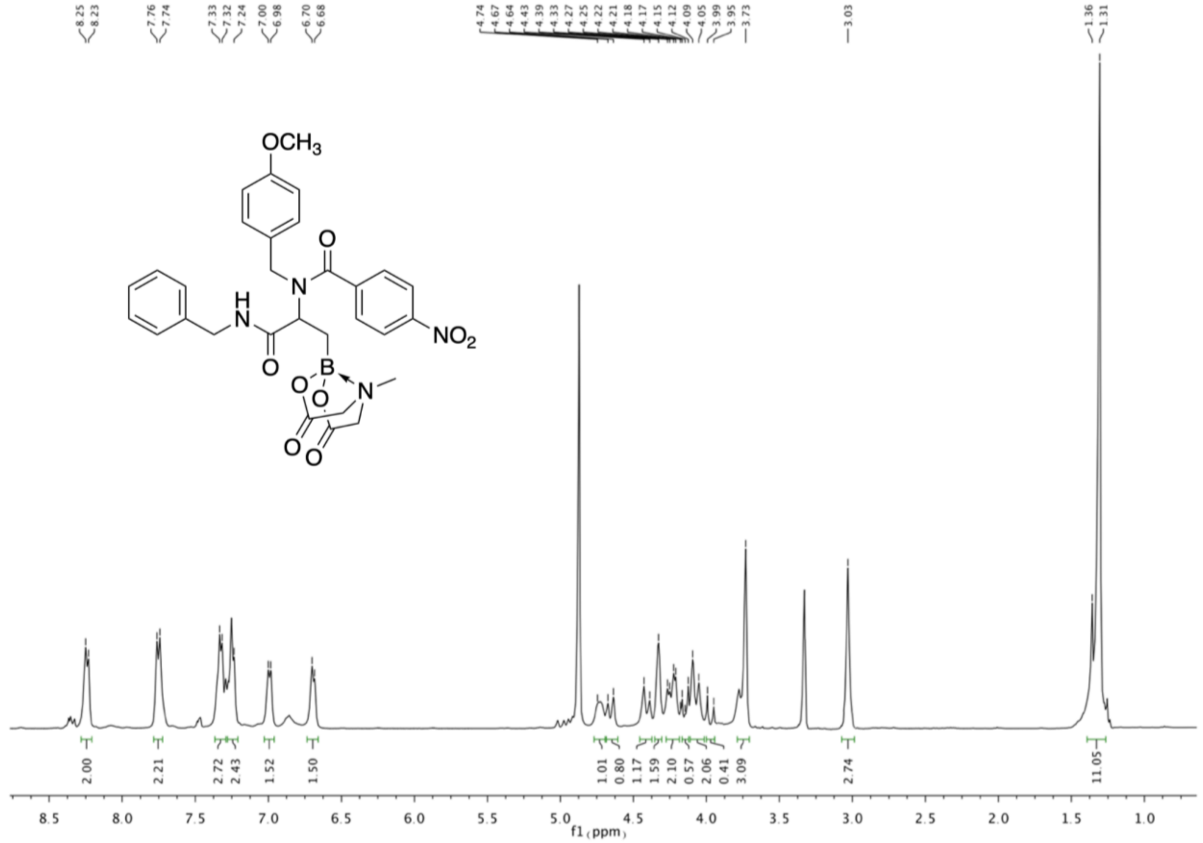

^{13}C NMR (101 MHz, CD_3OD) of compound **5n**

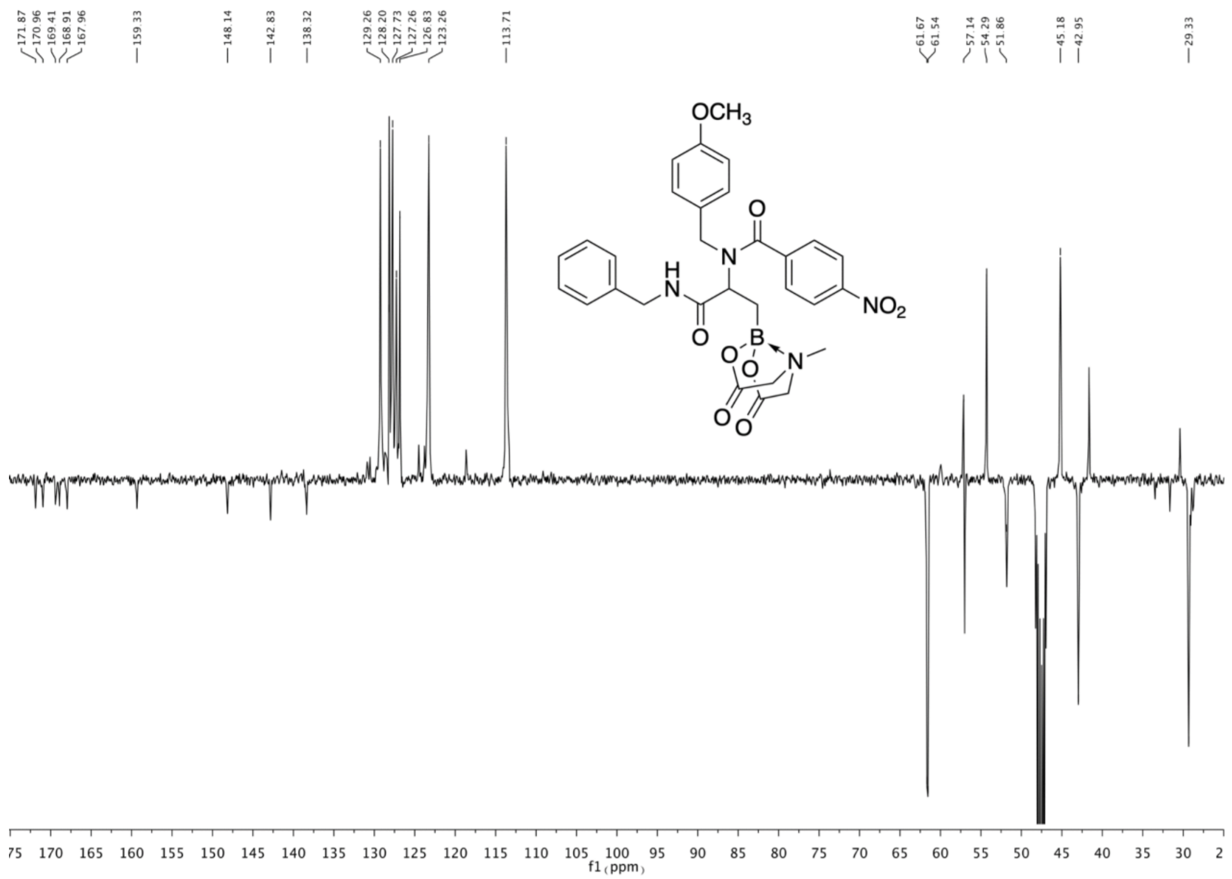

^{1}H NMR (300 MHz, CD_3OD, 8:2 rotameric mixture) of compound **5o**

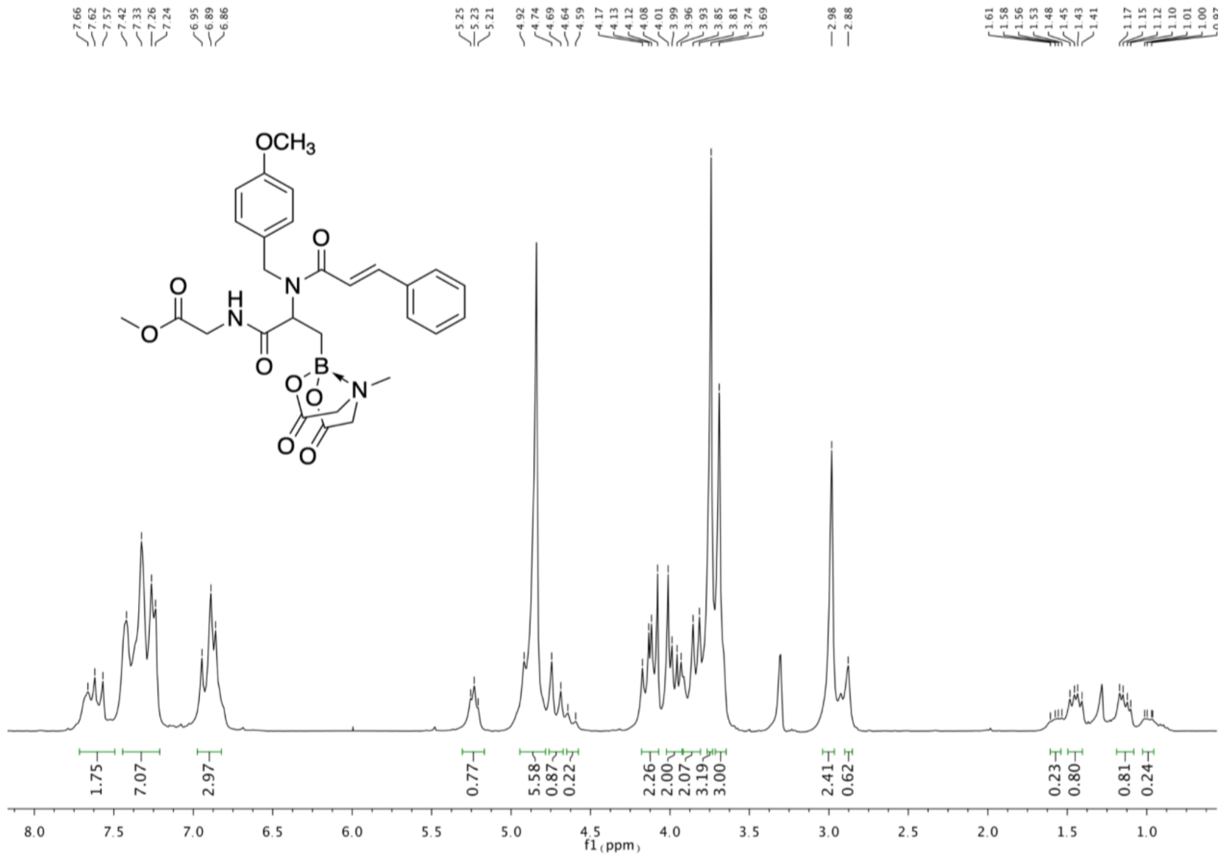

^{13}C NMR (75 MHz, CD_3OD, rotameric mixture) of compound **5o**

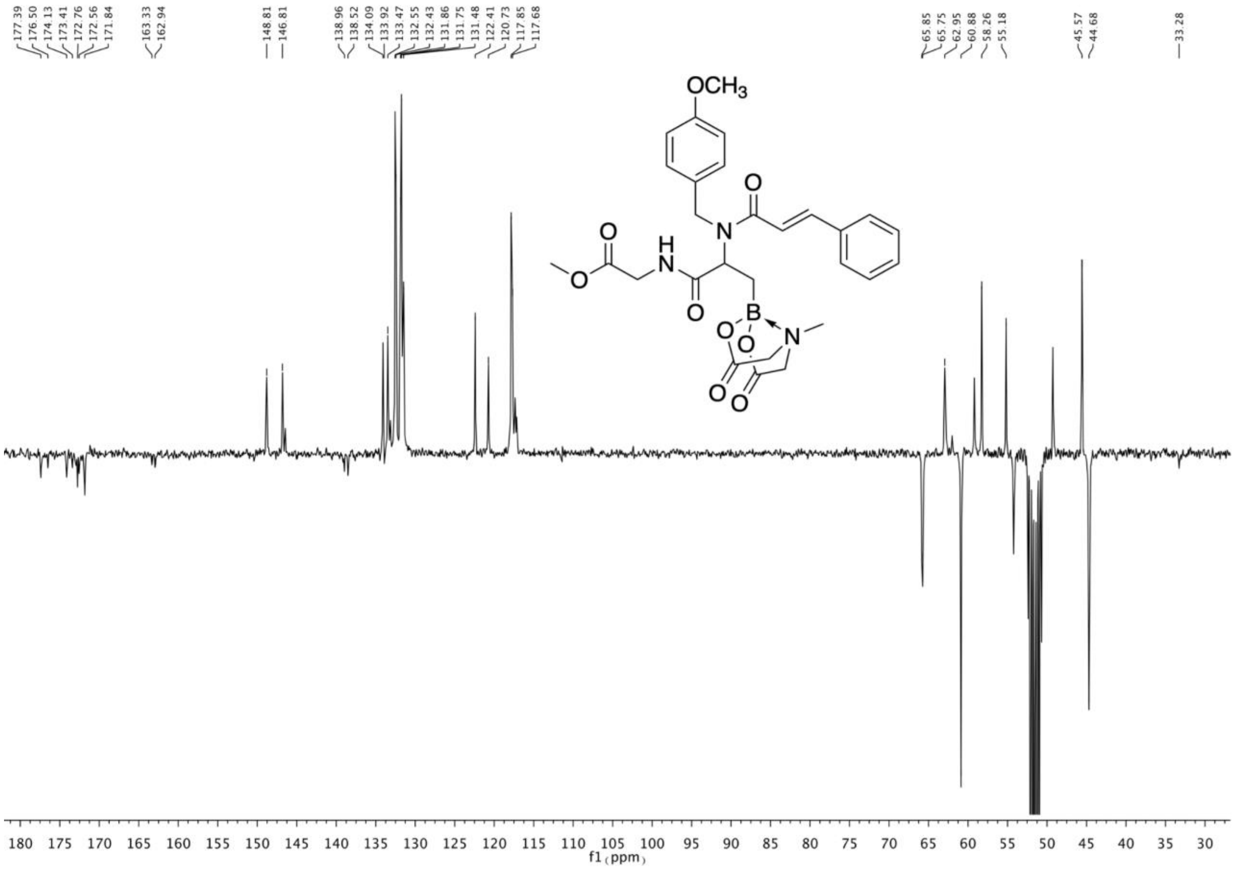

^{1}H NMR (300 MHz, 115°C, DMSO) of compound **5p**

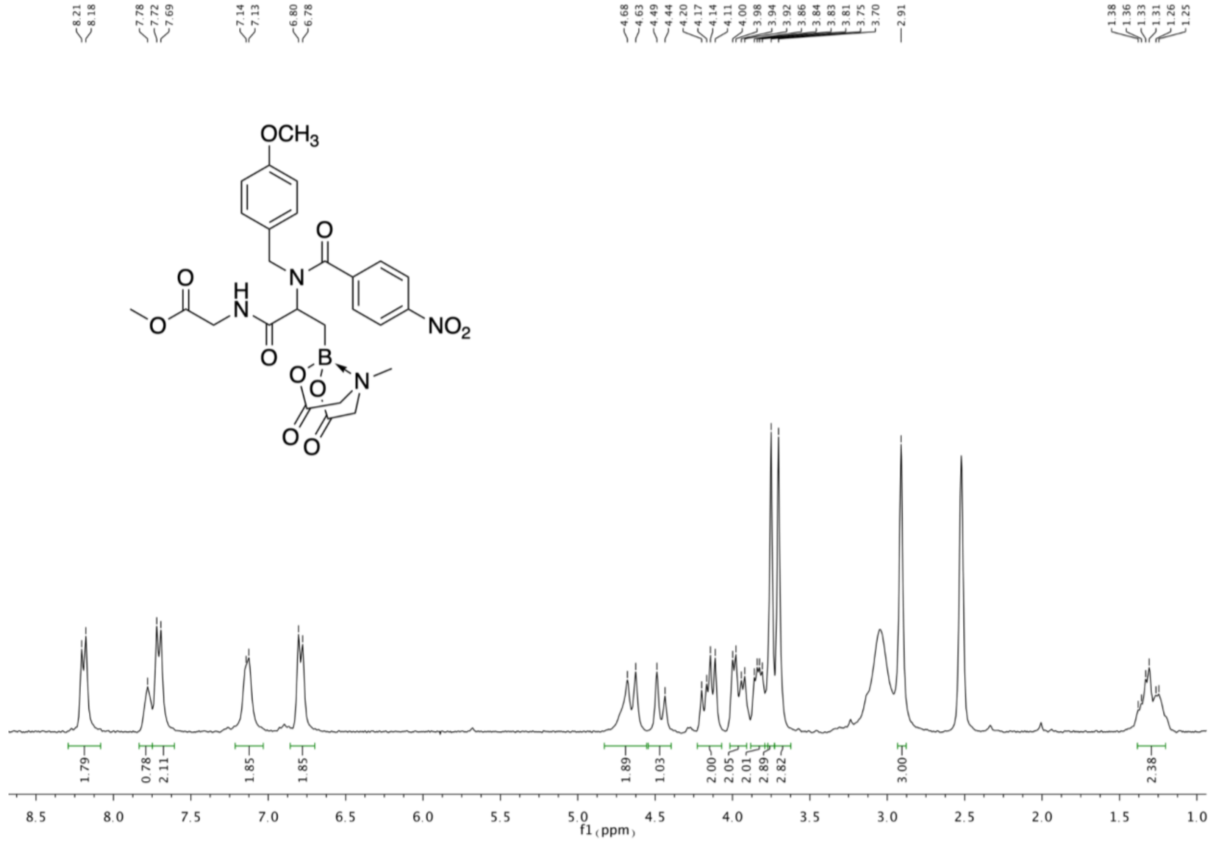

^{13}C NMR (75 MHz, 25°C, DMSO, rotameric mixture) of compound **5o**

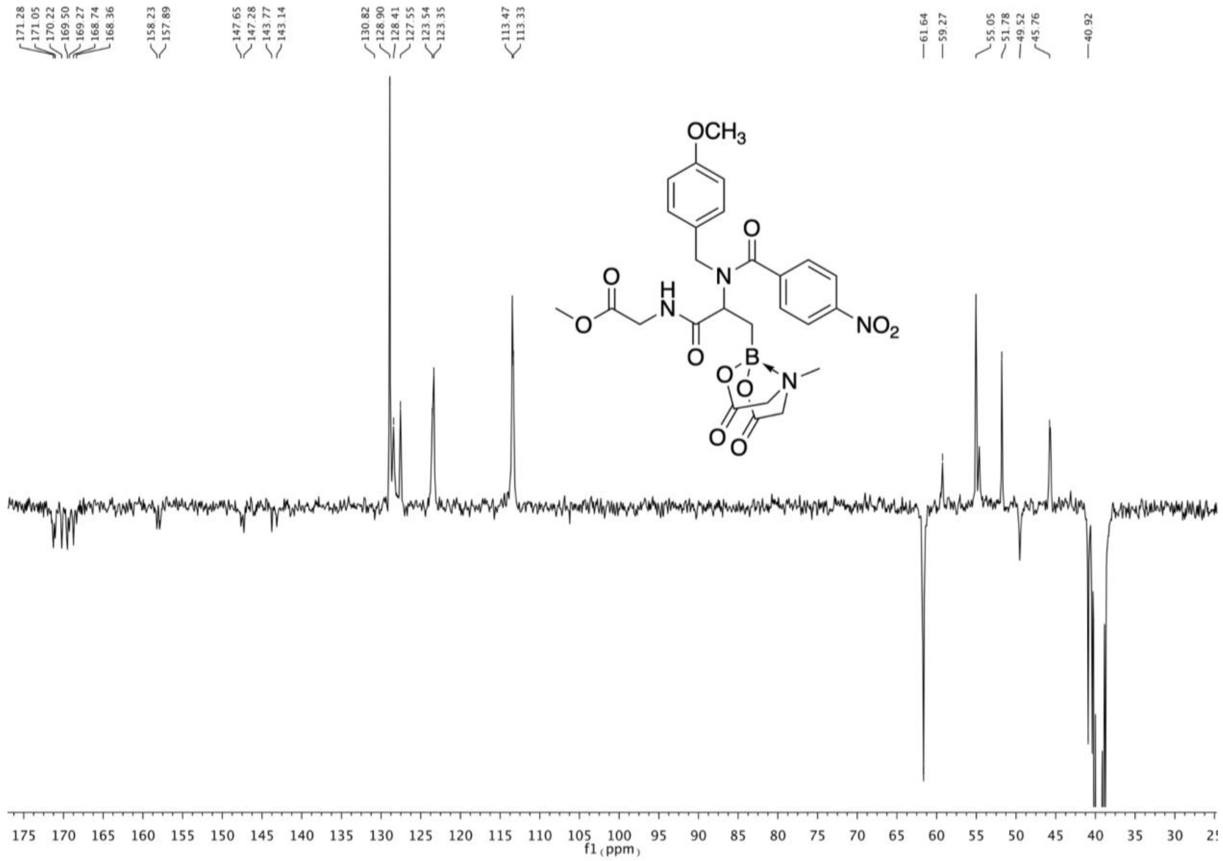

2. ^{1}H and ^{13}C NMR spectra of Ugi-4CR products (6a-p)

^{1}H NMR (400 MHz, 115°C, DMSO) of compound **6a**

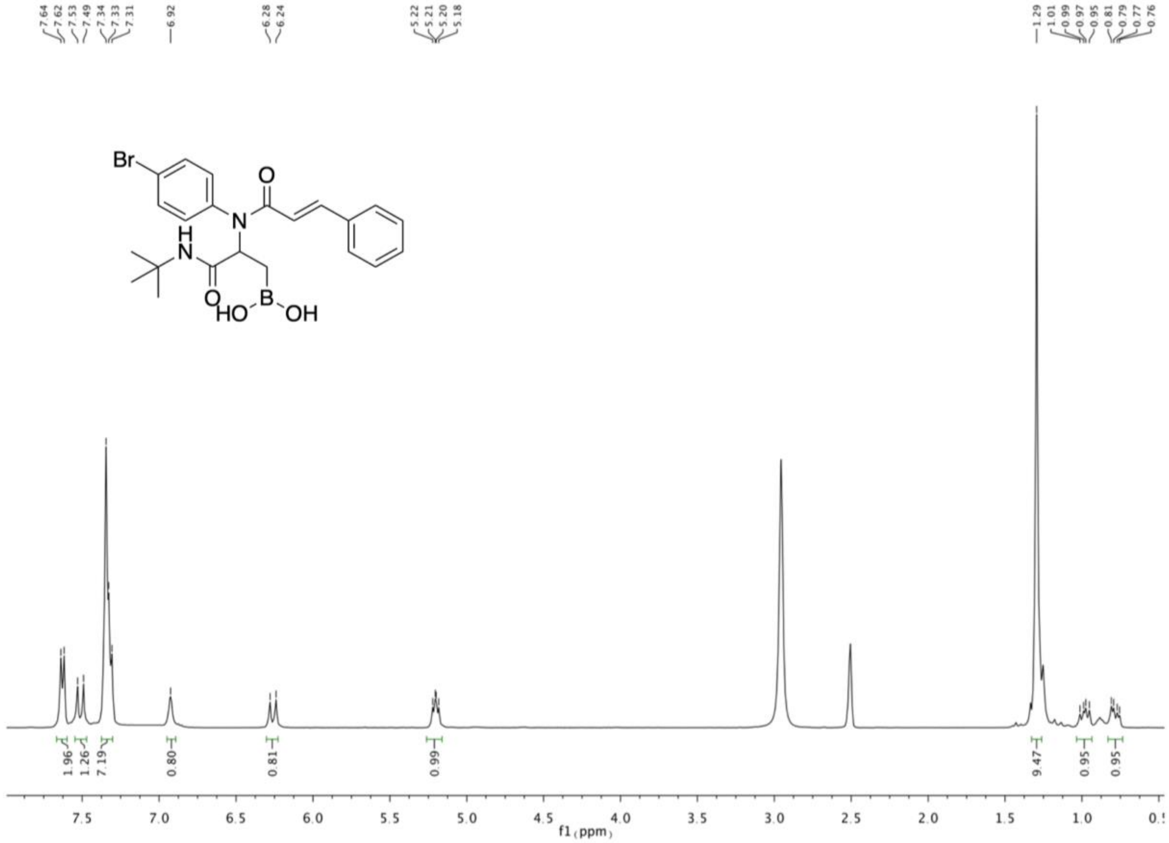

^{13}C NMR (75 MHz, 25°C, DMSO, 6:4 rotameric mixture) of compound **6a**

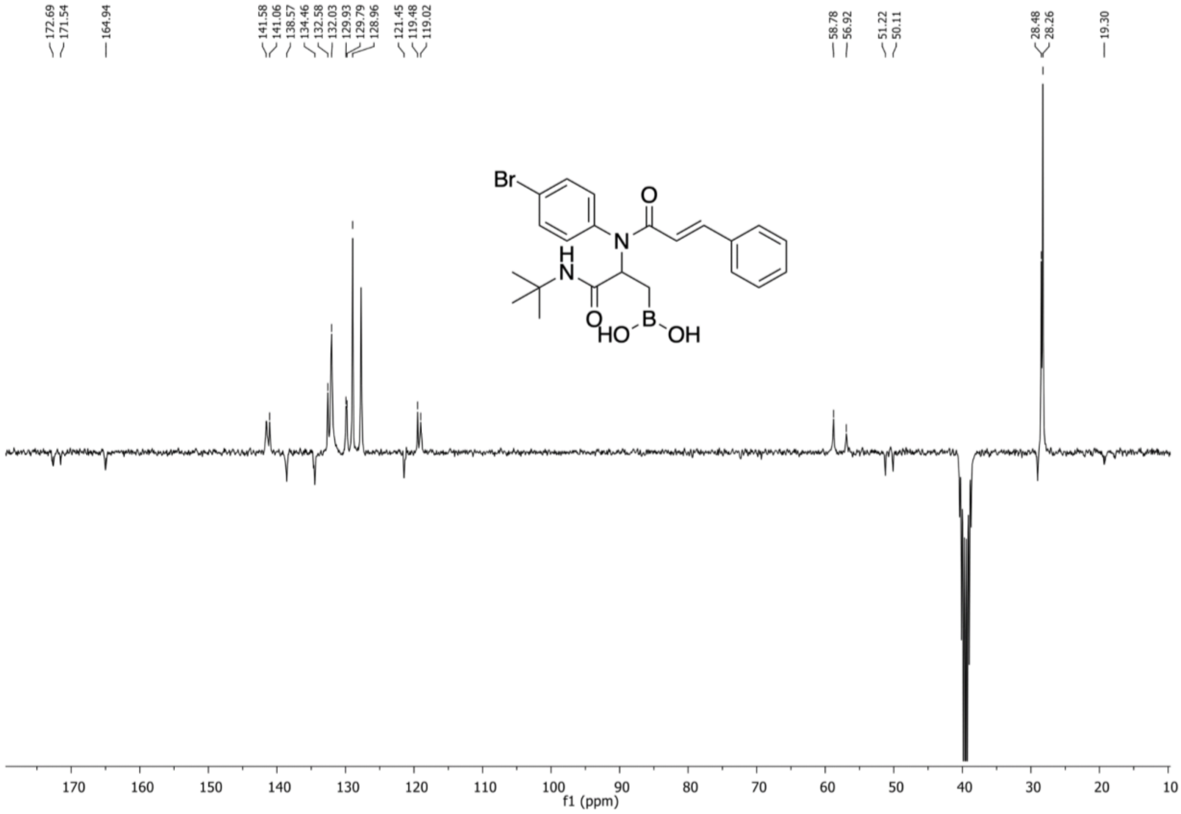

^{1}H NMR (400 MHz, CD_3OD) of compound **6b**

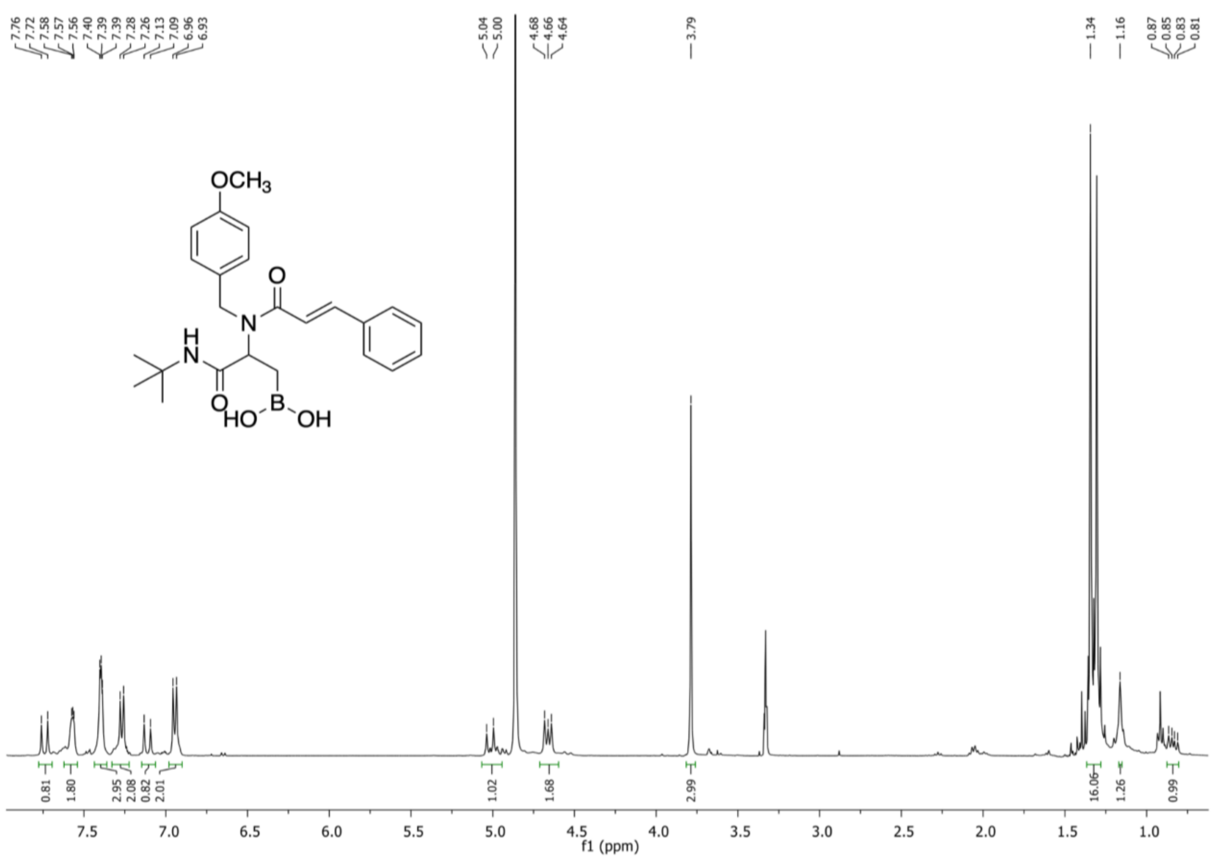

^{13}C NMR (75 MHz, CD_3OD) of compound **6b**

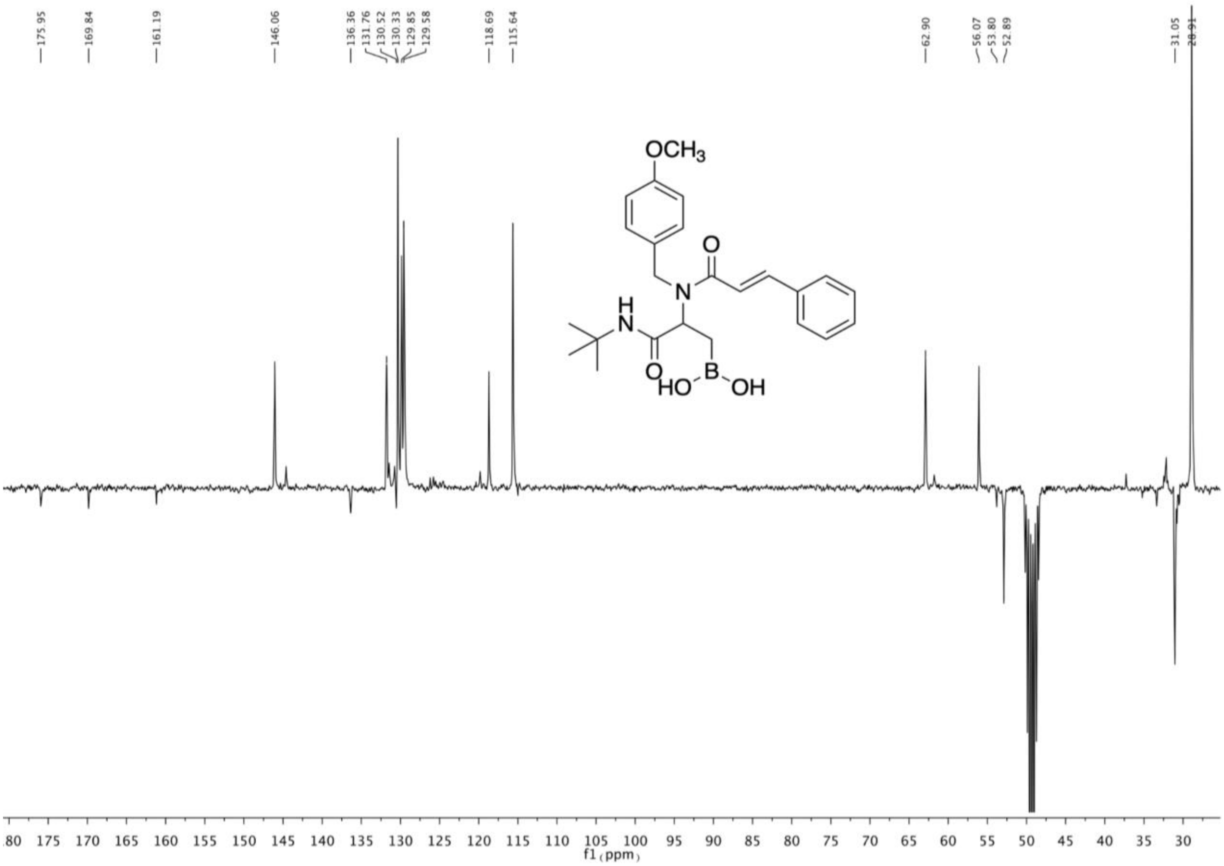

^{1}H NMR (400 MHz, CD_3OD) of compound **6c**

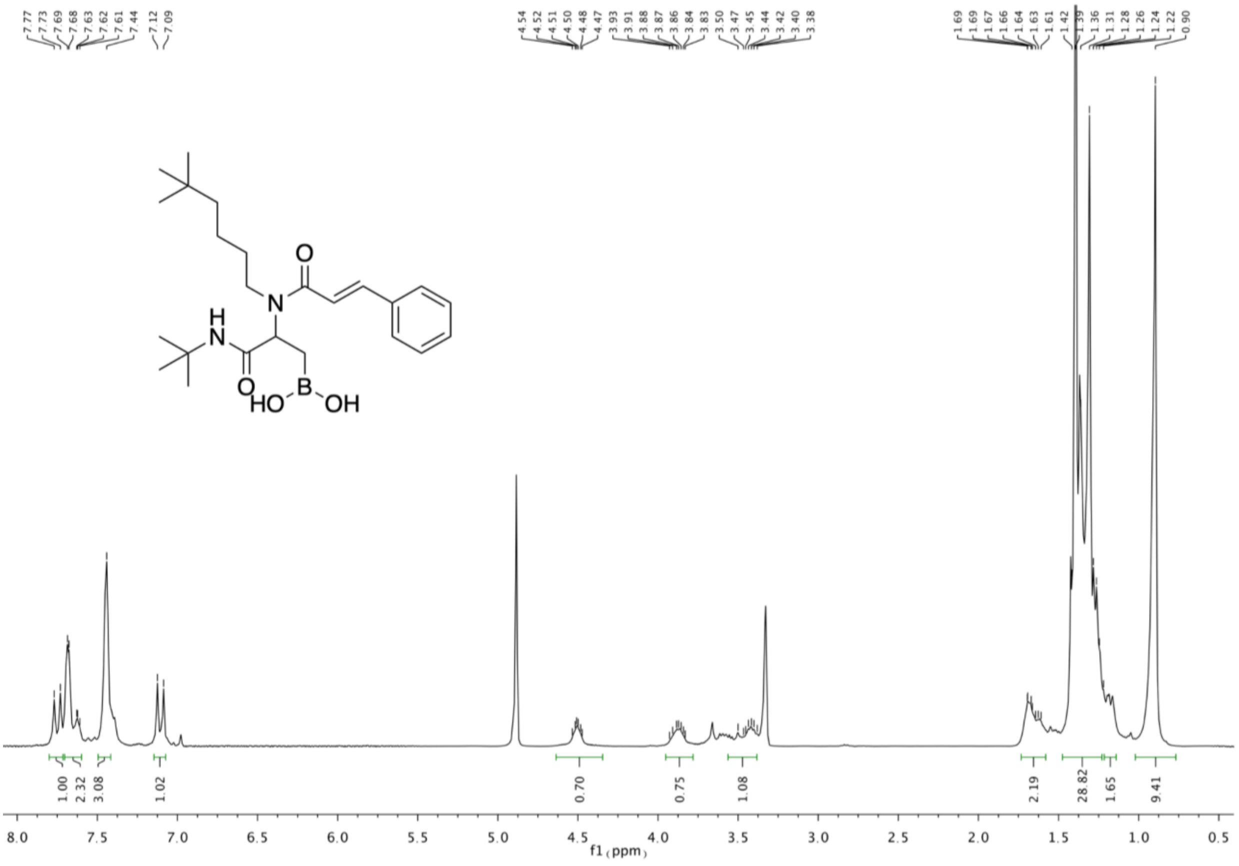

^{13}C NMR (75 MHz, CD_3OD) of compound **6c**

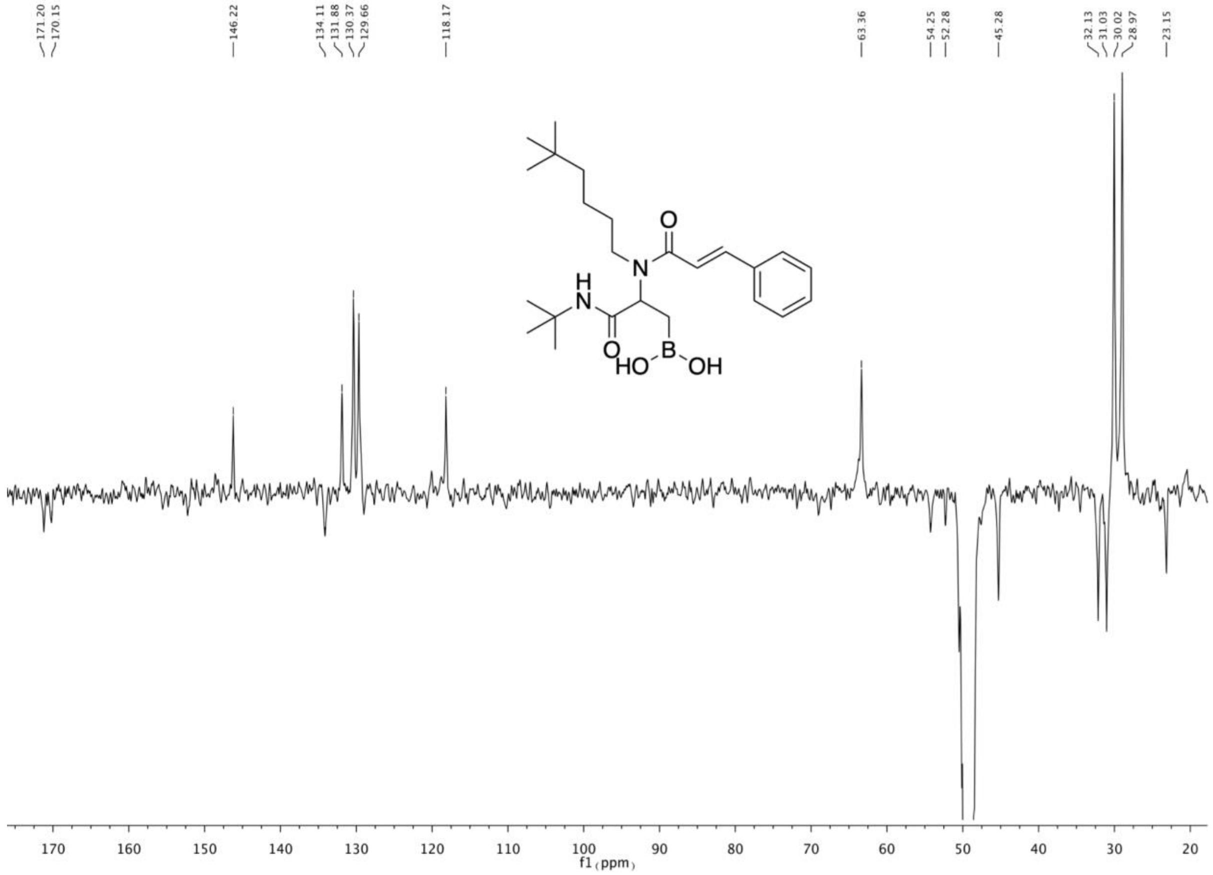

^{1}H NMR (400 MHz, 115°C, DMSO, 1:1 rotameric mixture) of compound **6d**

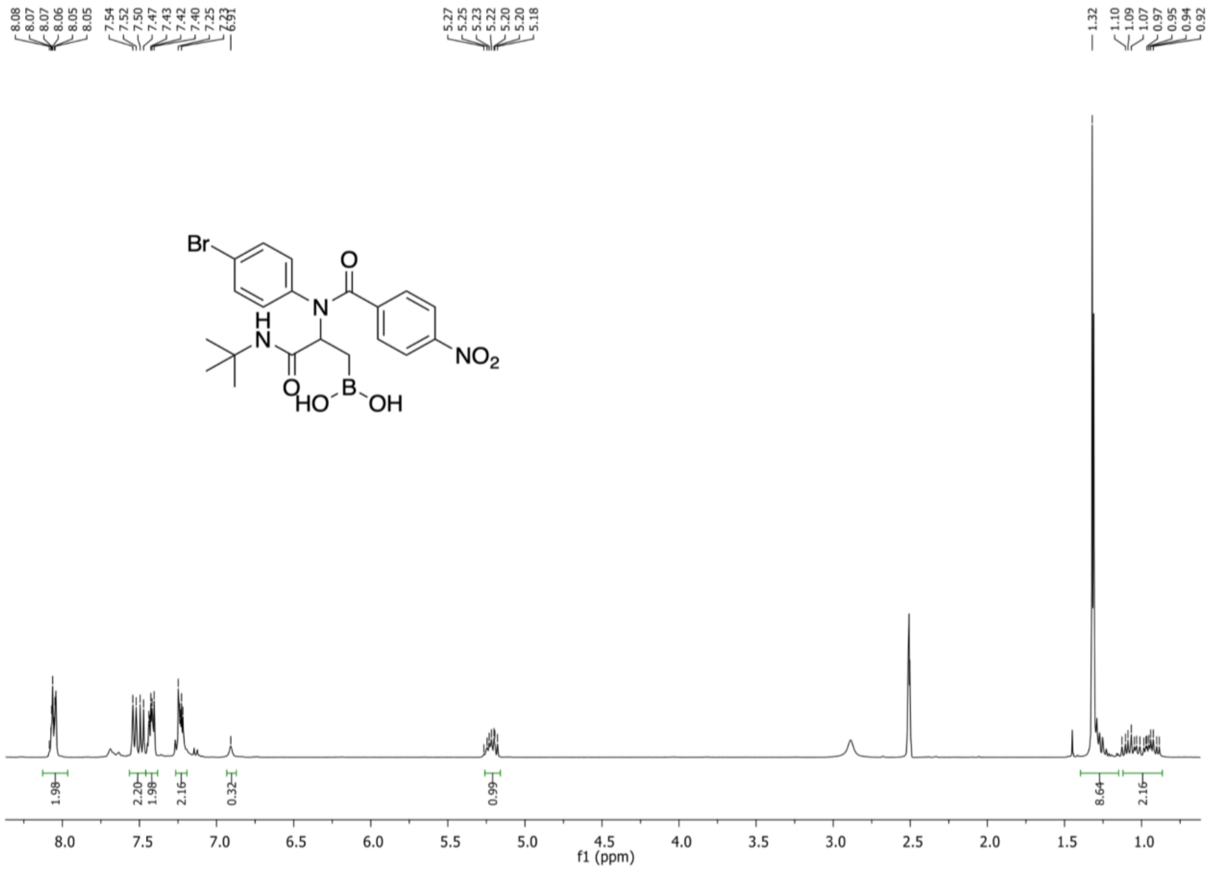

^{13}C NMR (75 MHz, 25°C, DMSO, 2:1 rotameric mixture) of compound **6d**

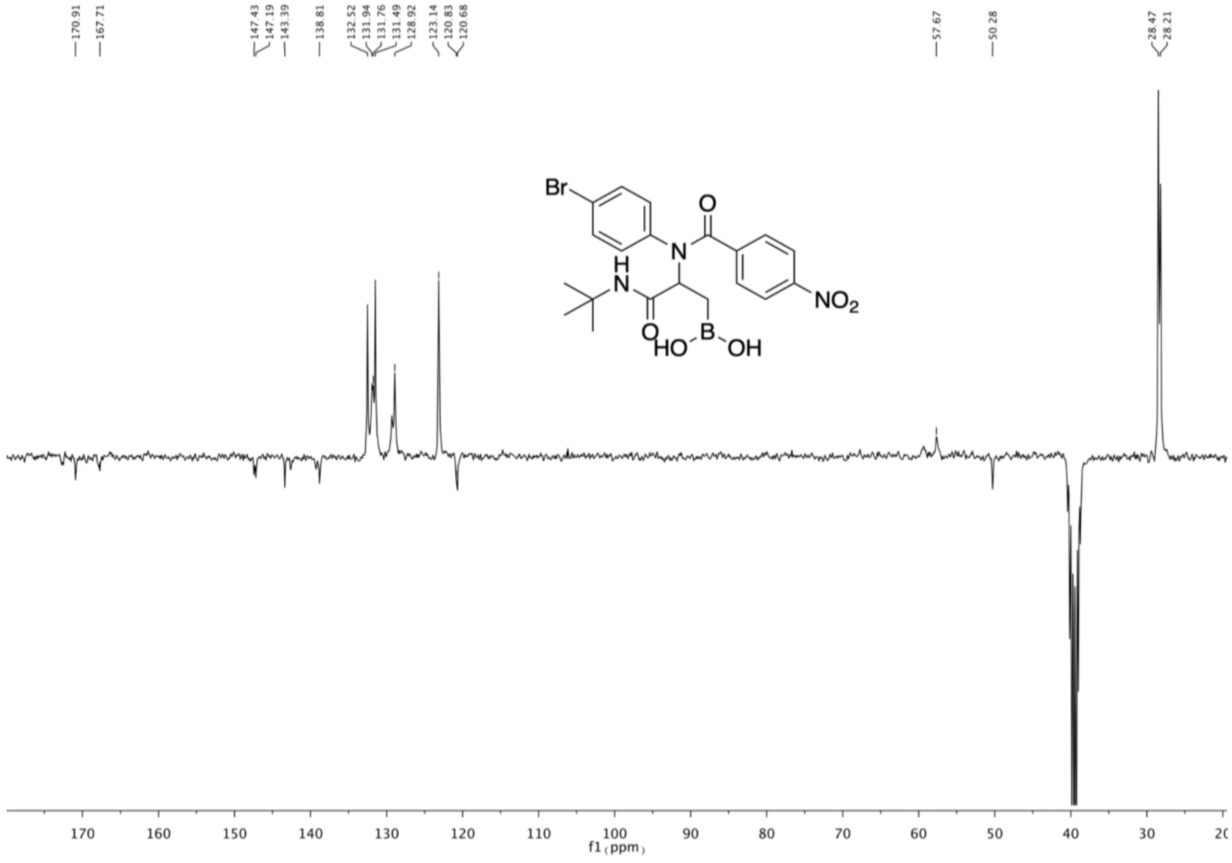

^{1}H NMR (400 MHz, 115°C, DMSO, 7:3 rotameric mixture) of compound **6e**

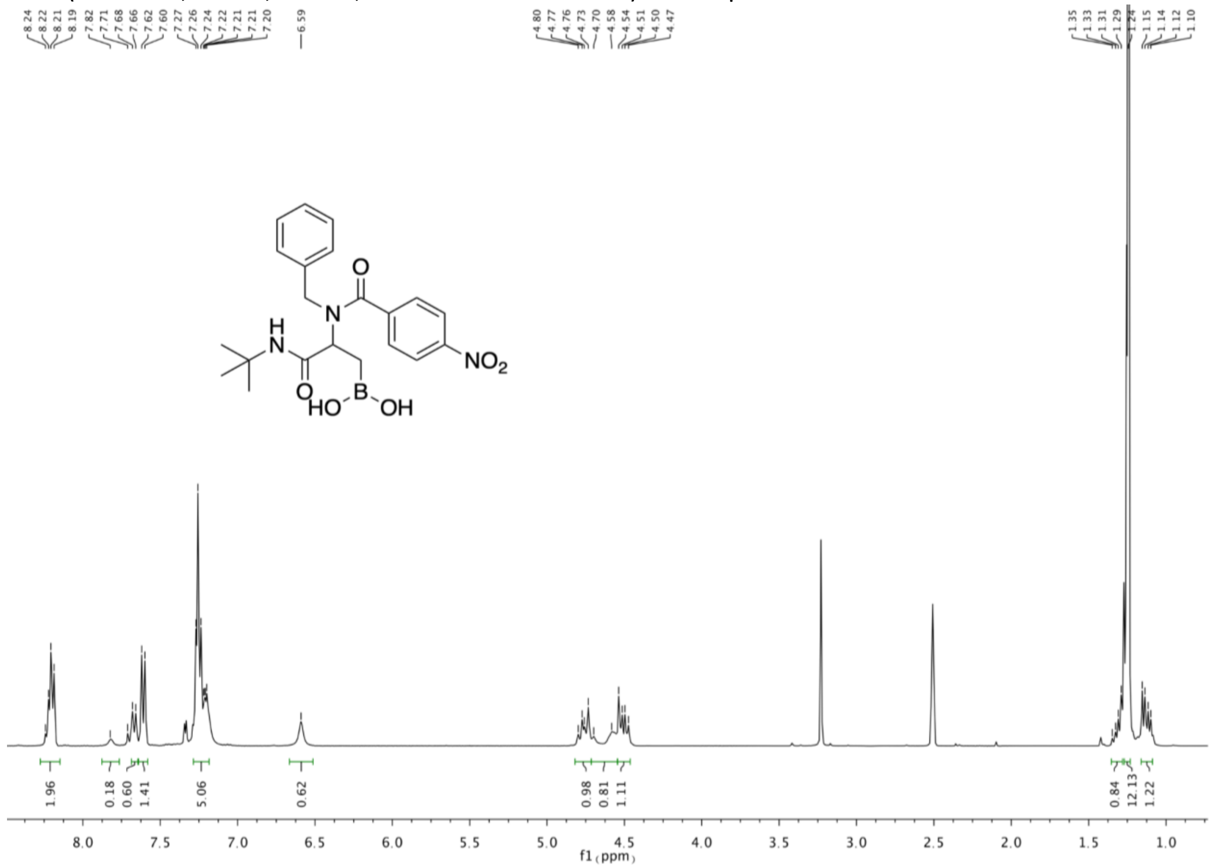

^{13}C NMR (75 MHz, 25°C, DMSO, rotameric mixture) of compound **6e**

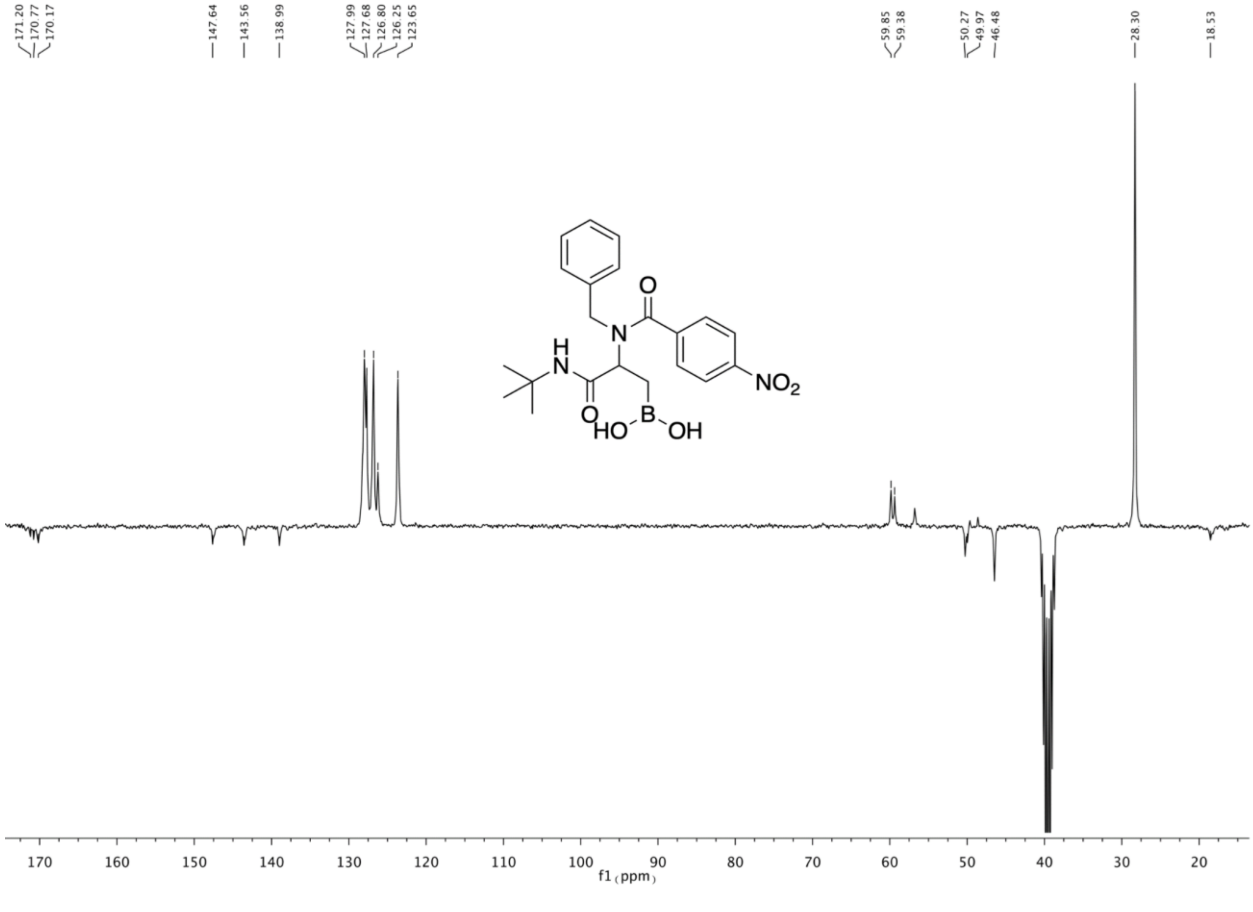

^{1}H NMR (400 MHz, CD_3OD) of compound **6f**

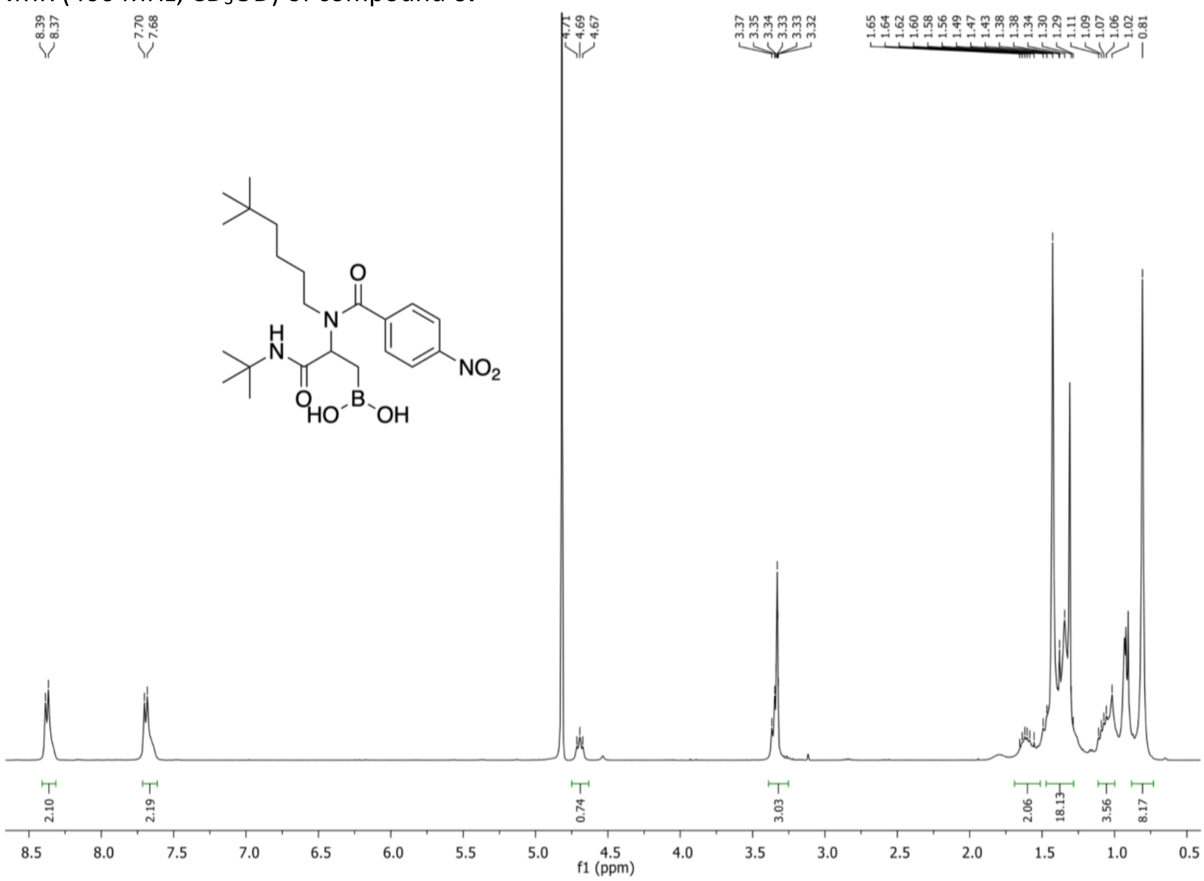

^{13}C NMR (75 MHz, CD_3OD) of compound **6f**

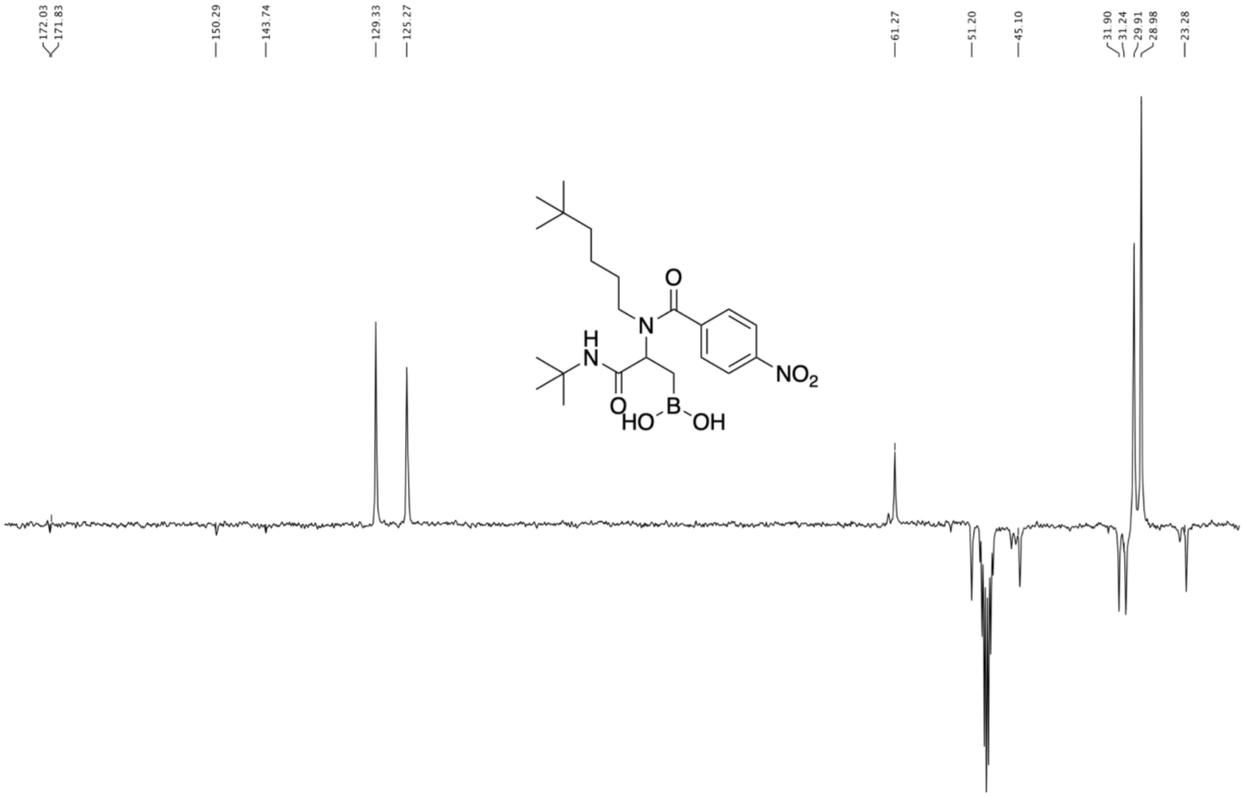

^{1}H NMR (400 MHz, 115°C, DMSO) of compound **6g**

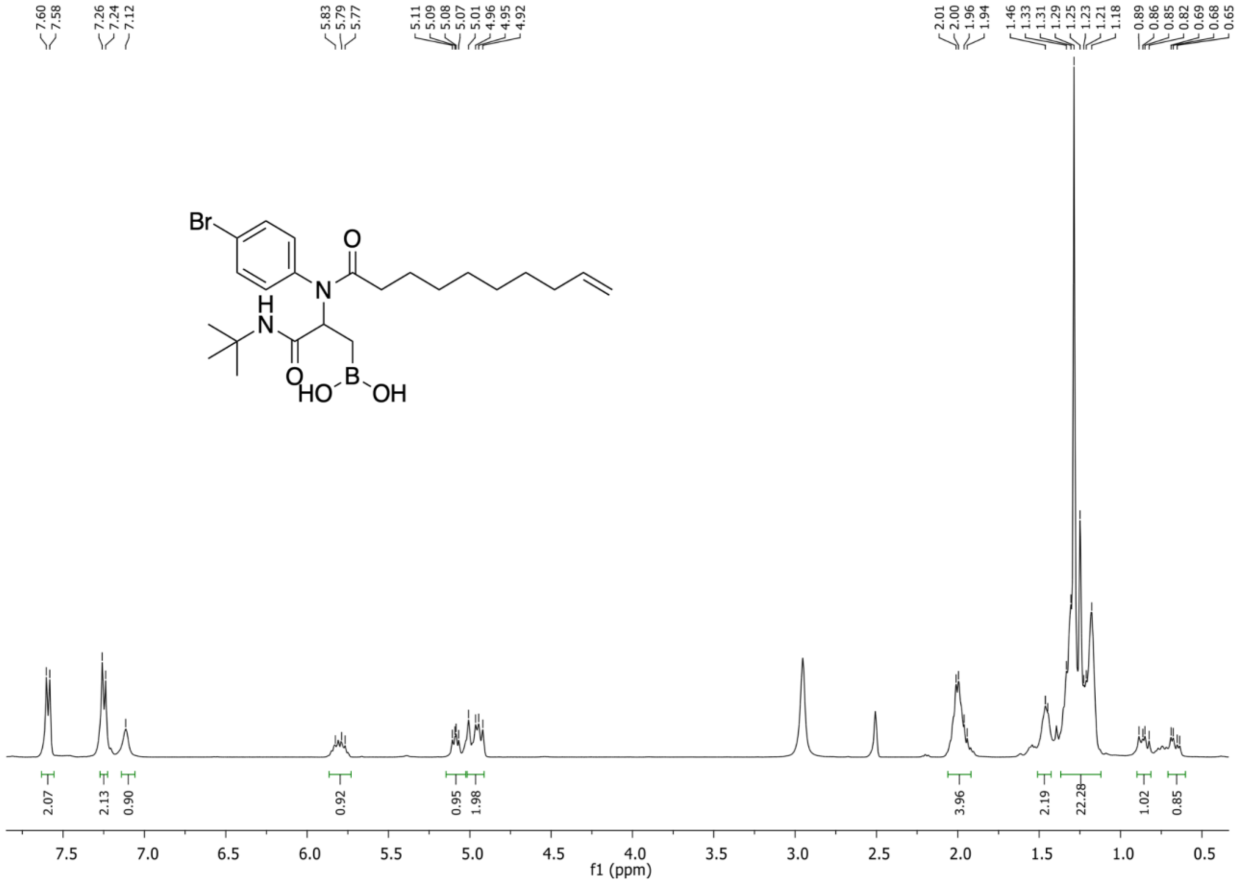

^{13}C NMR (75 MHz, 25°C, DMSO, rotameric mixture) of compound **6g**

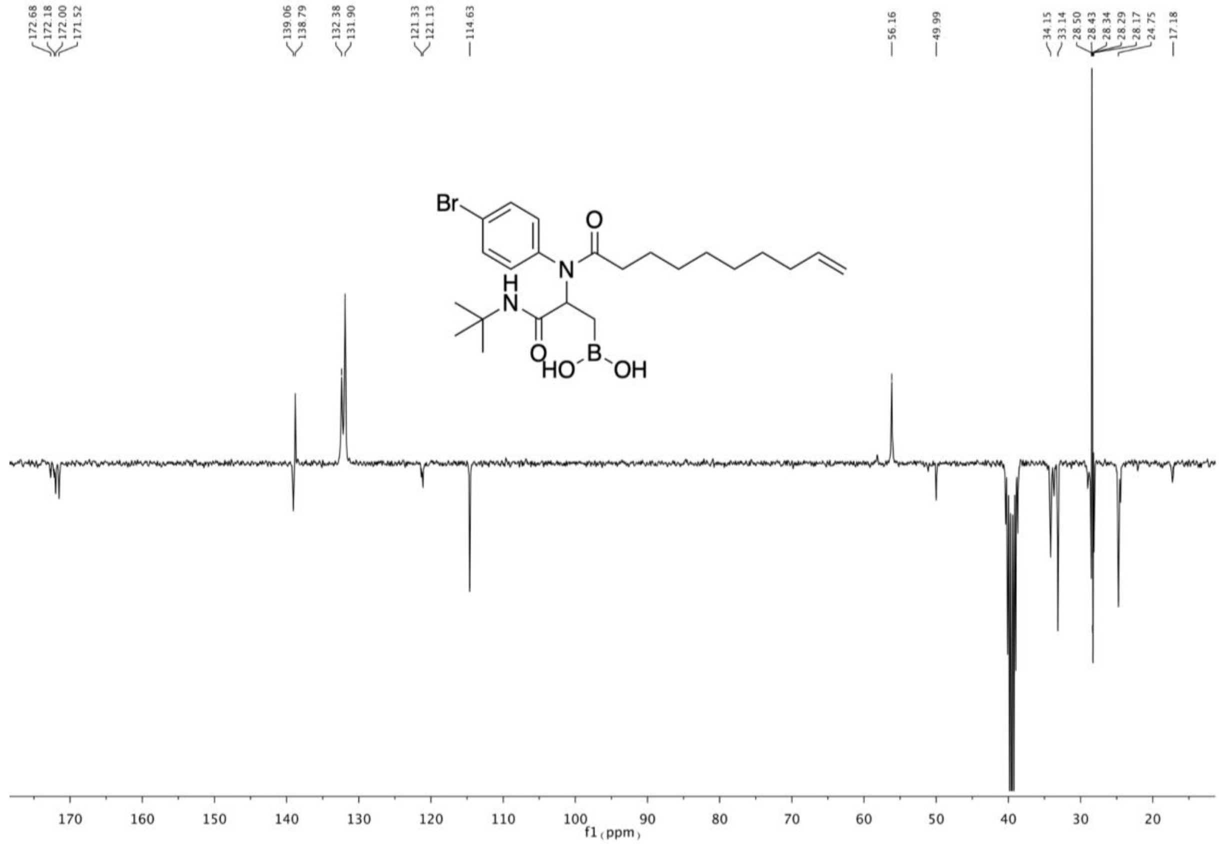

^{1}H NMR (400 MHz, CD_3OD, 8:2 rotameric mixture) of compound **6h**

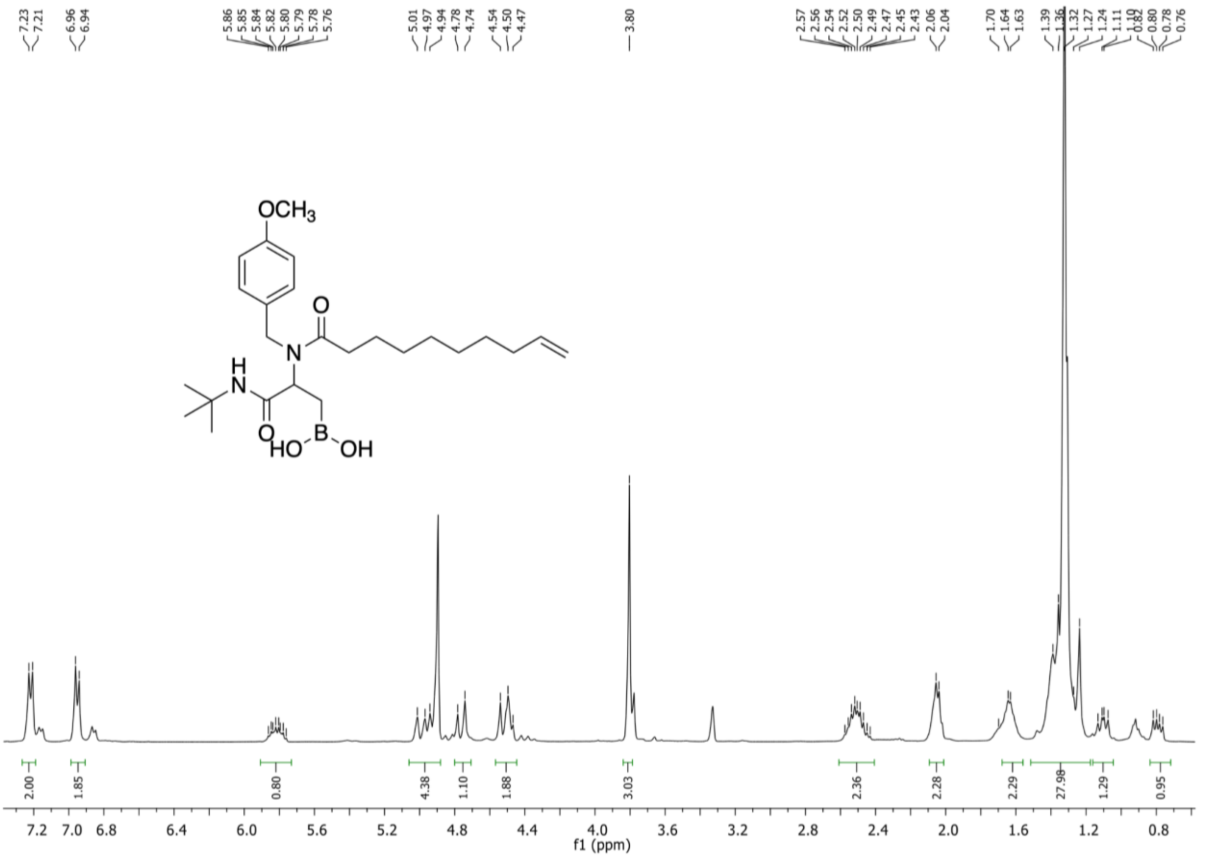

^{13}C NMR (75 MHz, CD_3OD) of compound **6h**

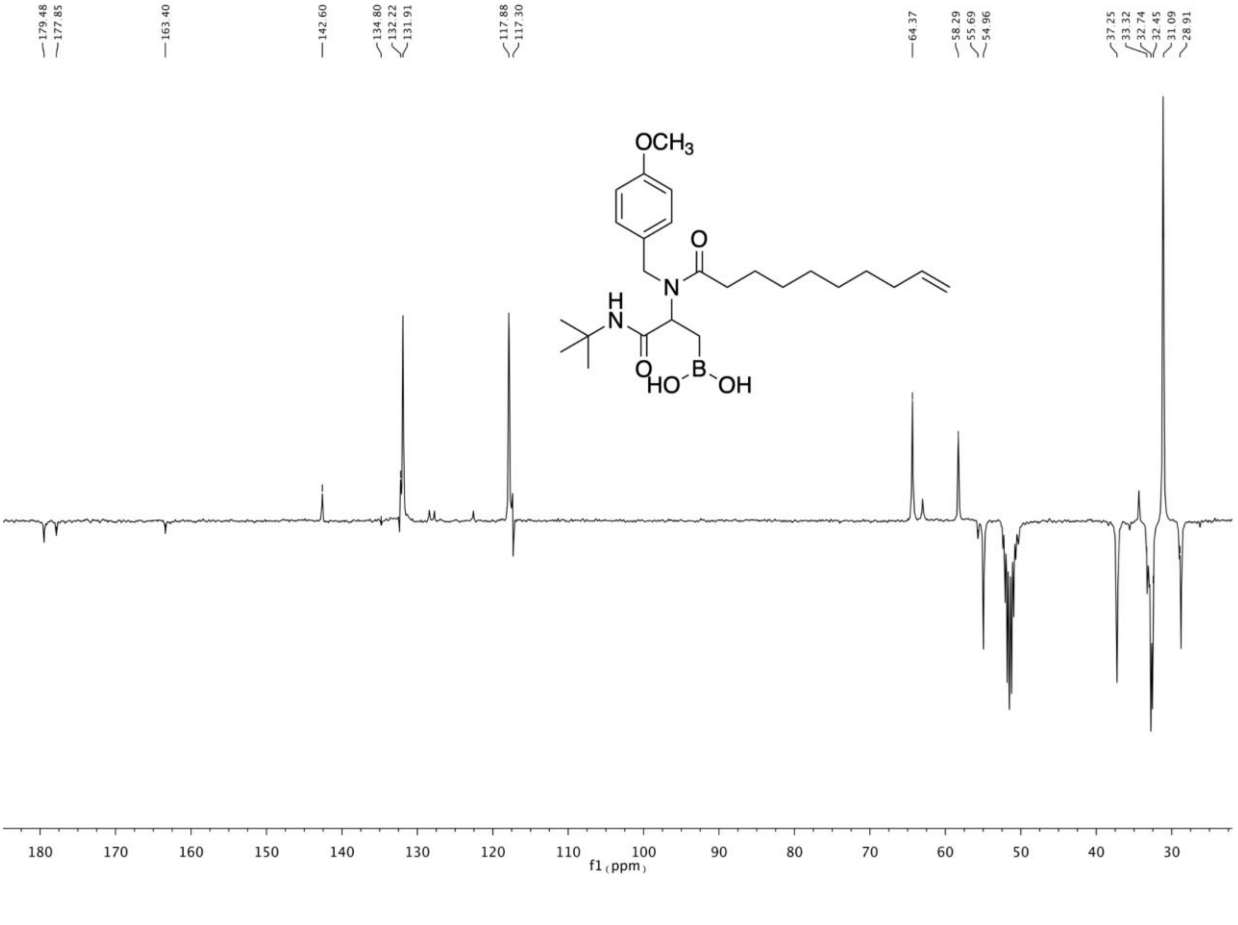

^{1}H NMR (400 MHz, 115°C, DMSO) of compound **6i**

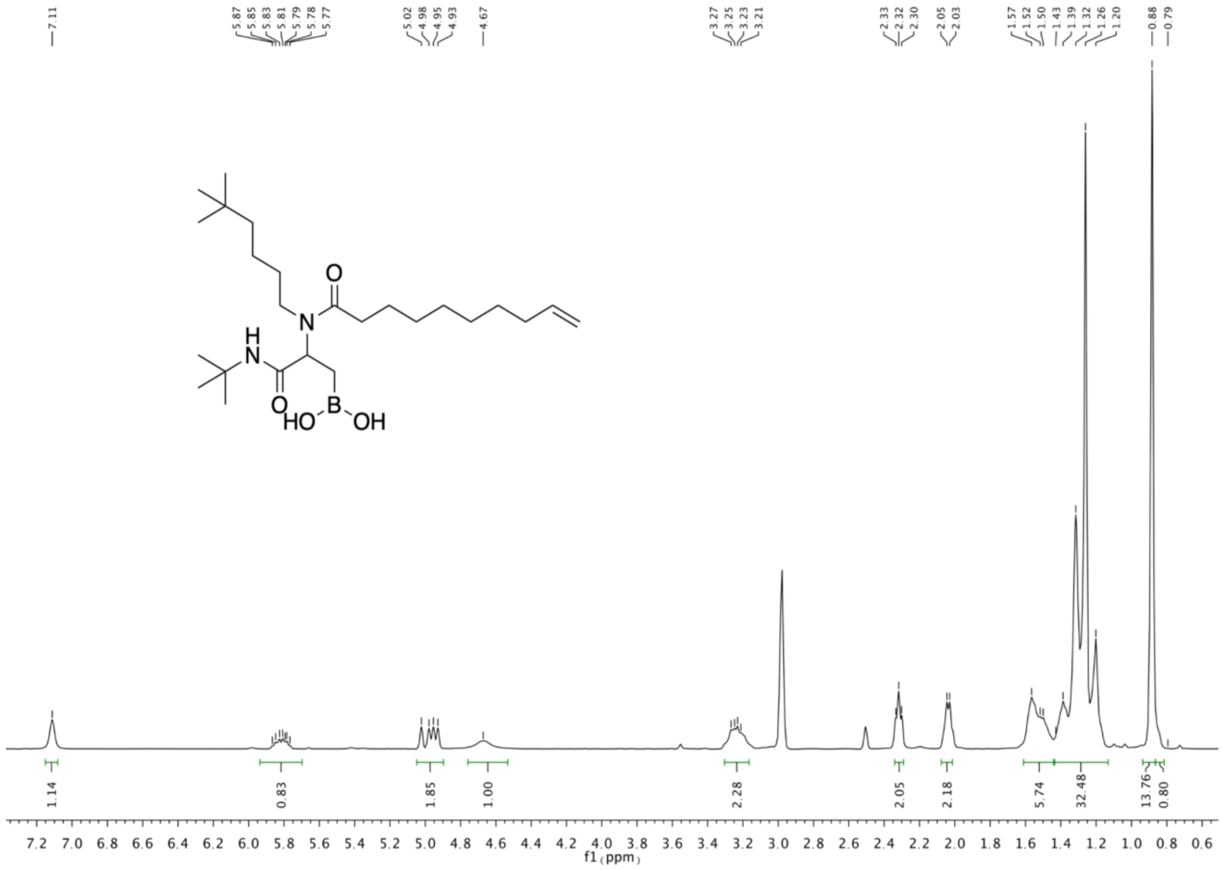

^{13}C NMR (75 MHz, 25°C, CD_3OD) of compound **6i**

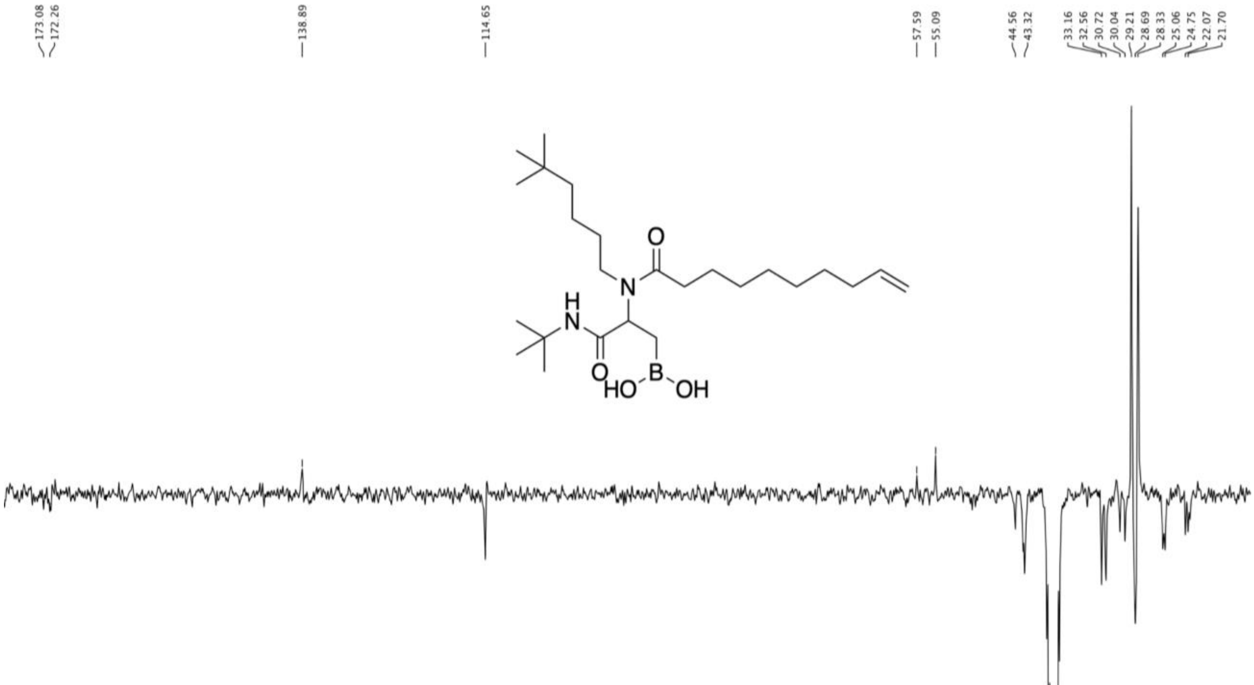

^{1}H NMR (400 MHz, 115°C, DMSO, 6:4 rotameric mixture) of compound **6j**

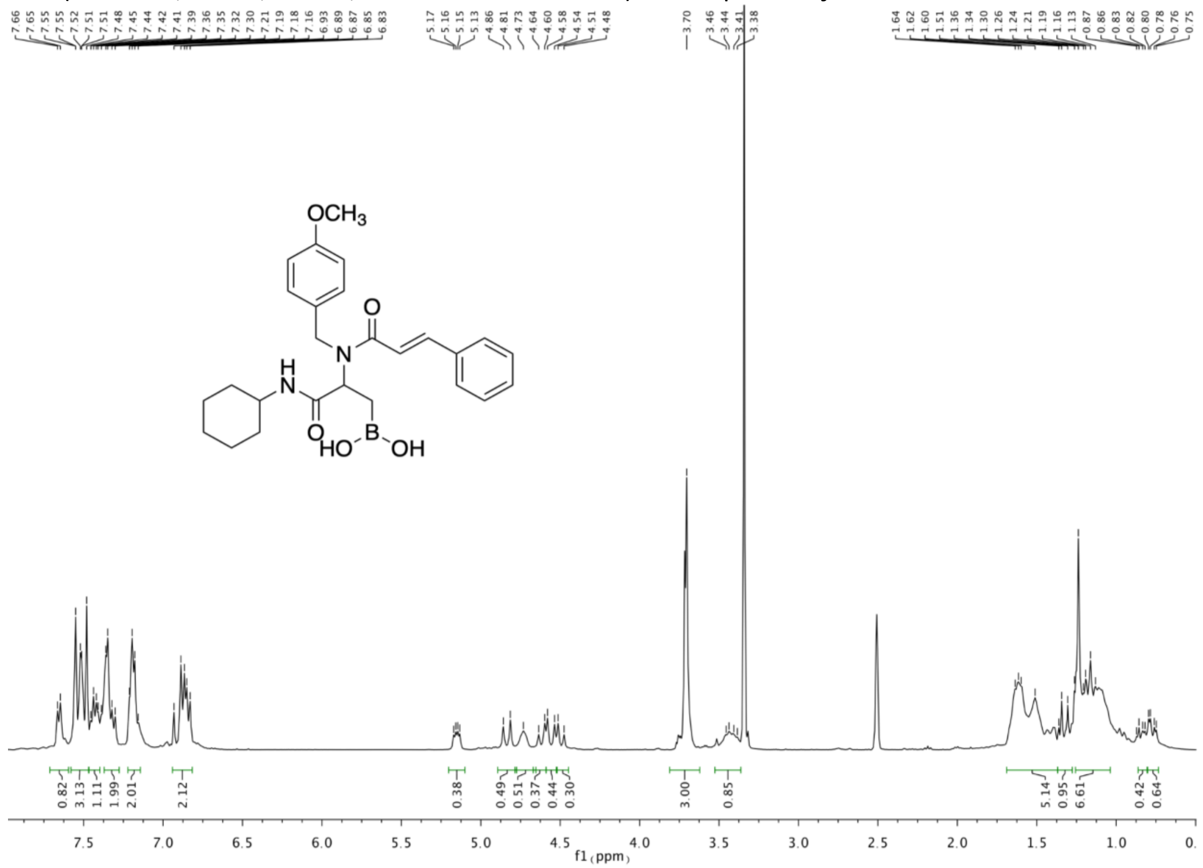

^{13}C NMR (75 MHz, 25°C, DMSO, 6:4 rotameric mixture) of compound **6j**

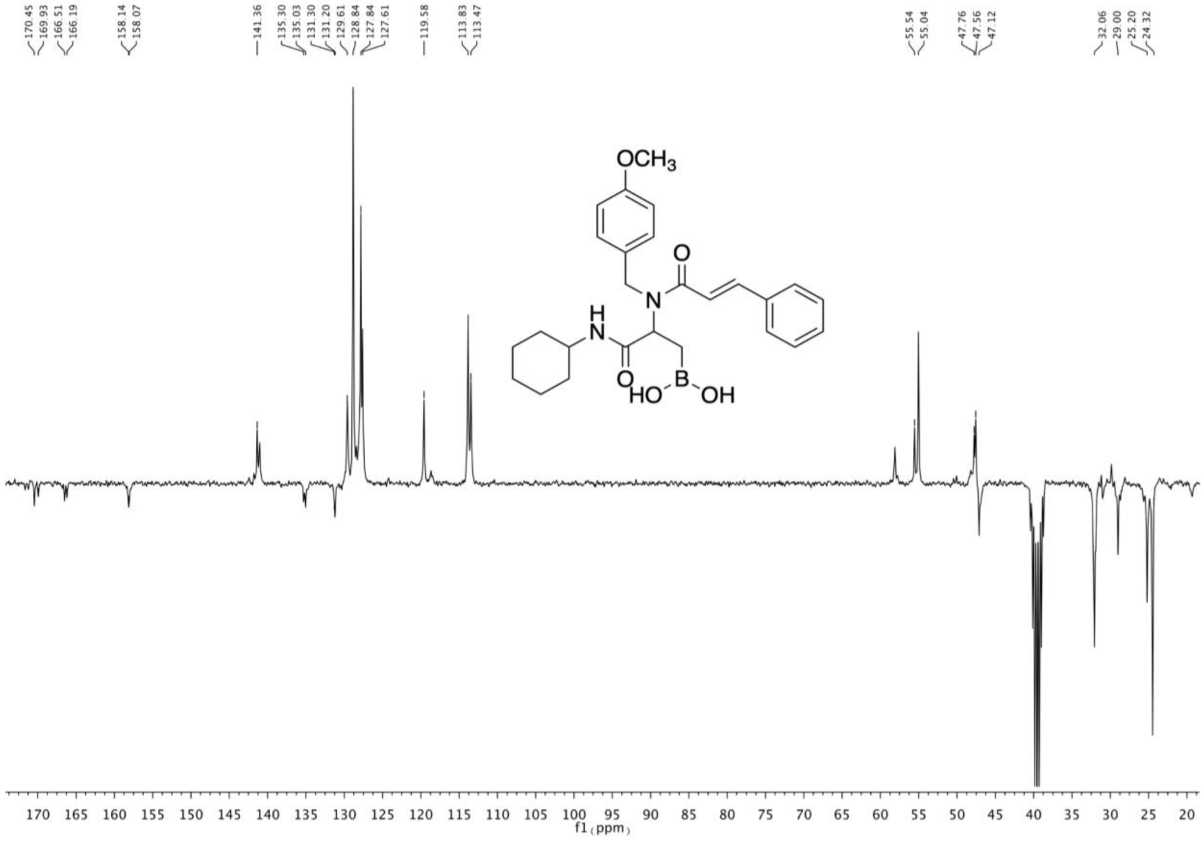

^{1}H NMR (400 MHz, CD_3OD, 6:4 rotameric mixture) of compound **6k**

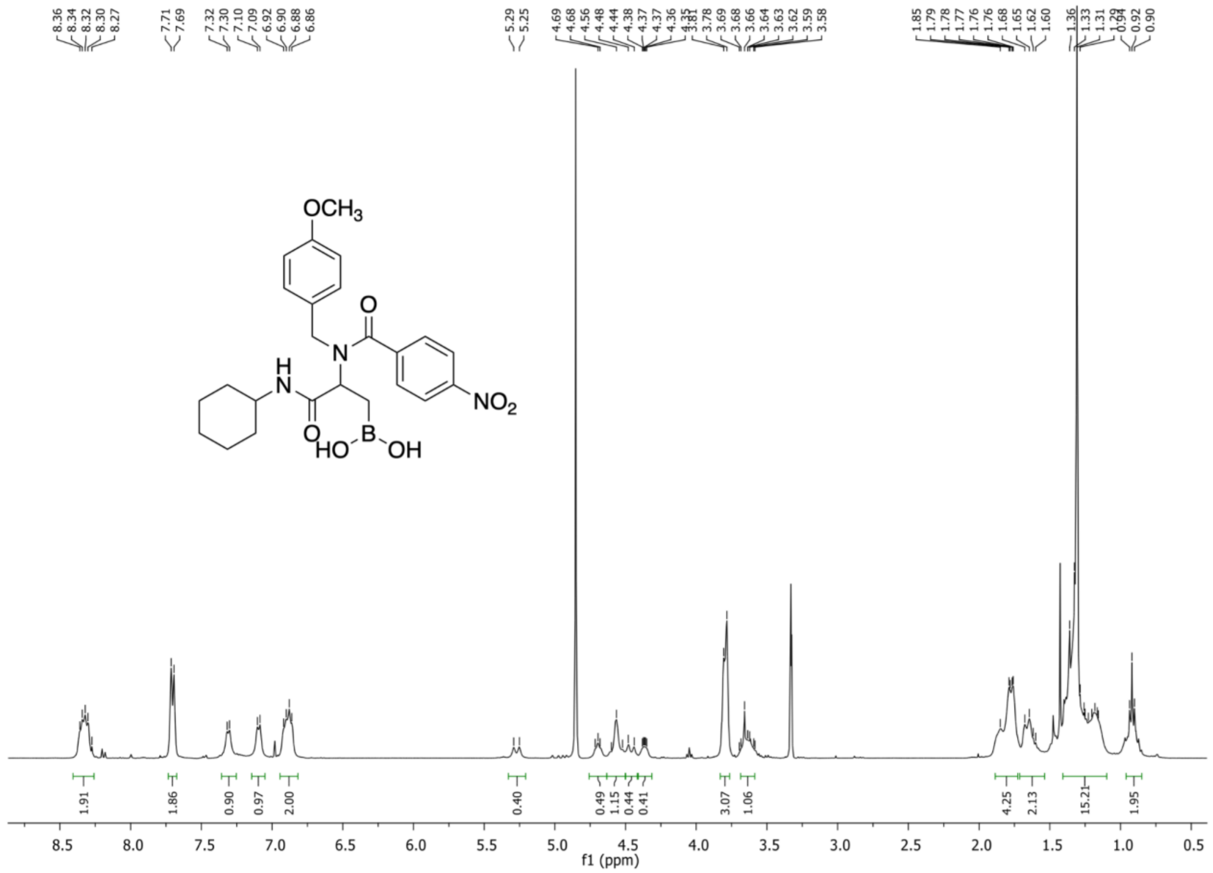

^{13}C NMR (75 MHz, CD_3OD, 6:4 rotameric mixture) of compound **6k**

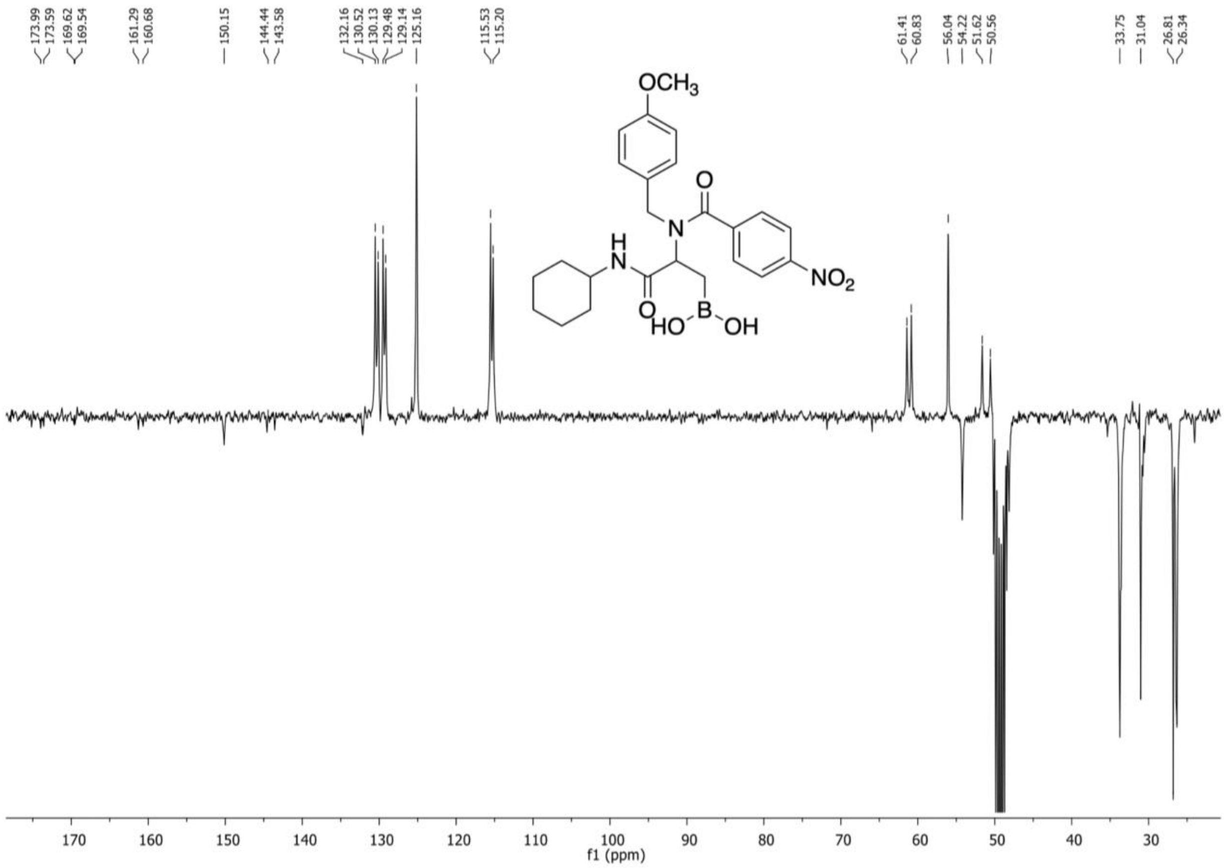

^{1}H NMR (400 MHz, DMSO, rotameric mixture) of compound **6l**

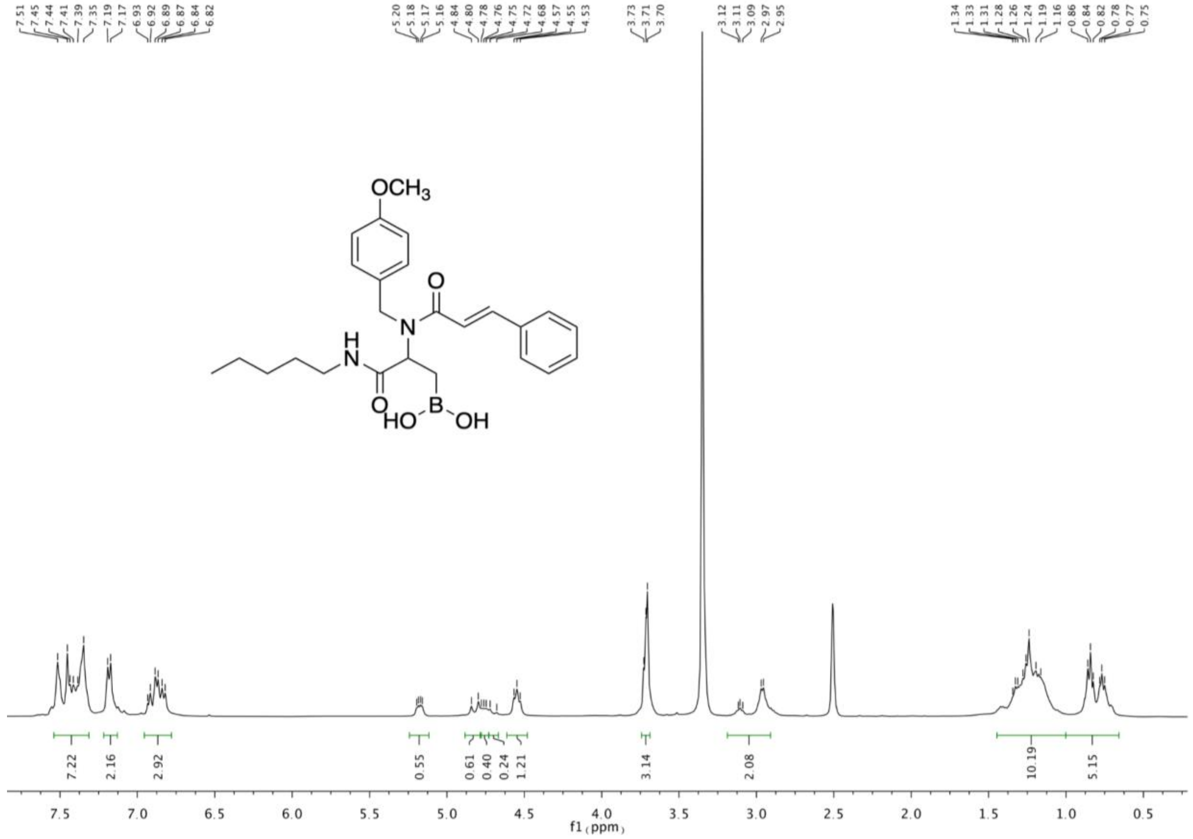

^{13}C NMR (75 MHz, DMSO, rotameric mixture) of compound **6l**

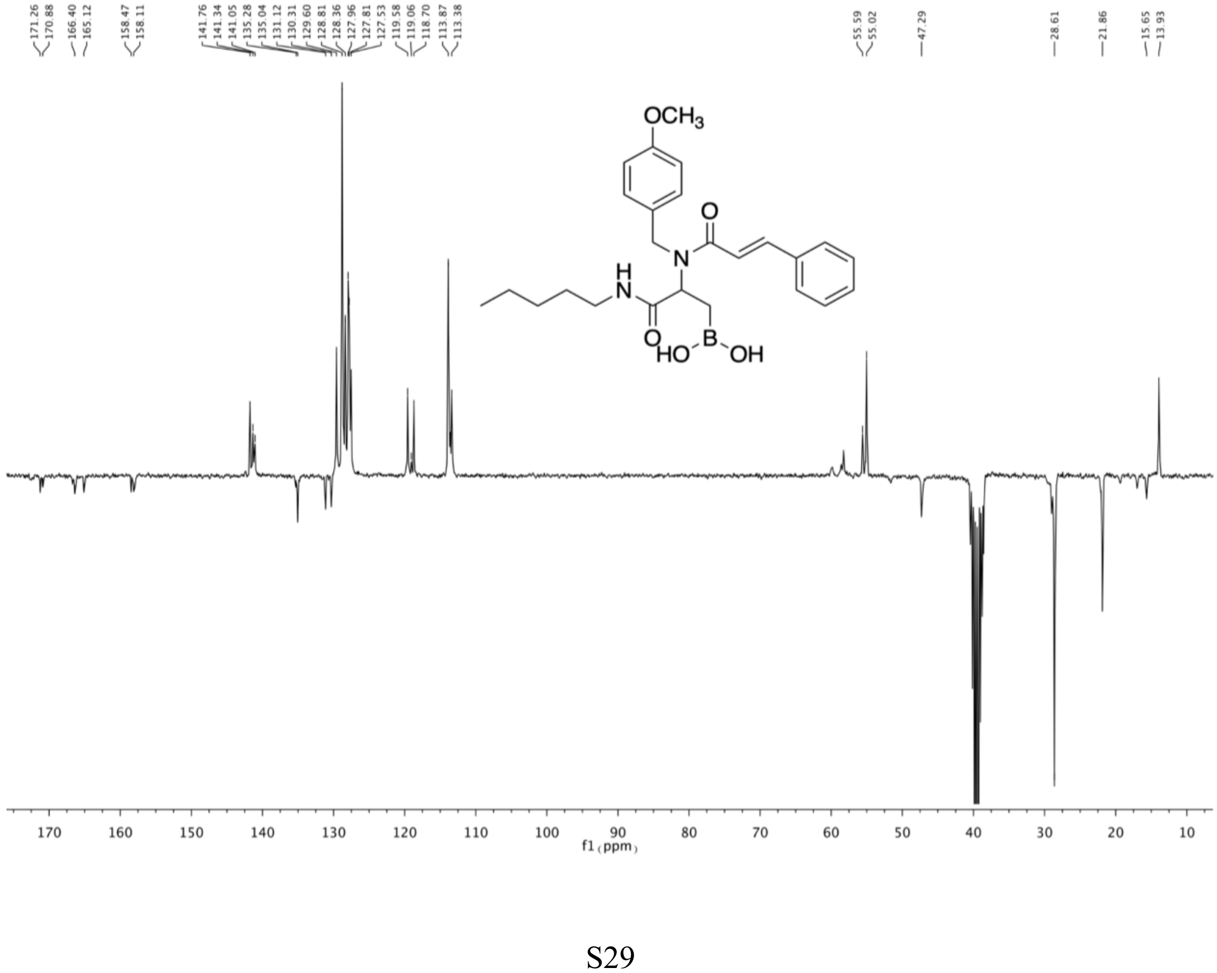

^{1}H NMR (400 MHz, CD_3OD, 6:4 rotameric mixture) of compound **6m**

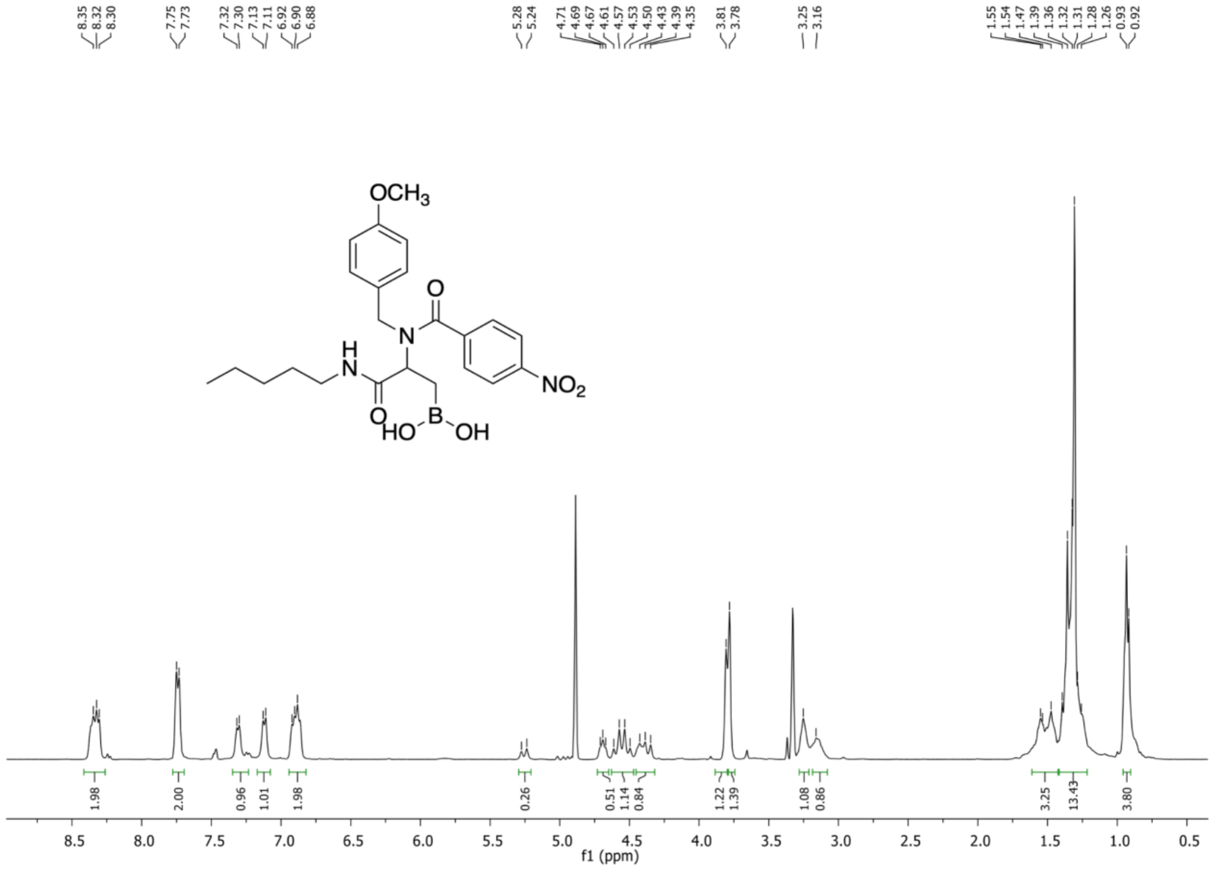

^{13}C NMR (101 MHz, CD_3OD, rotameric mixture) of compound **6m**

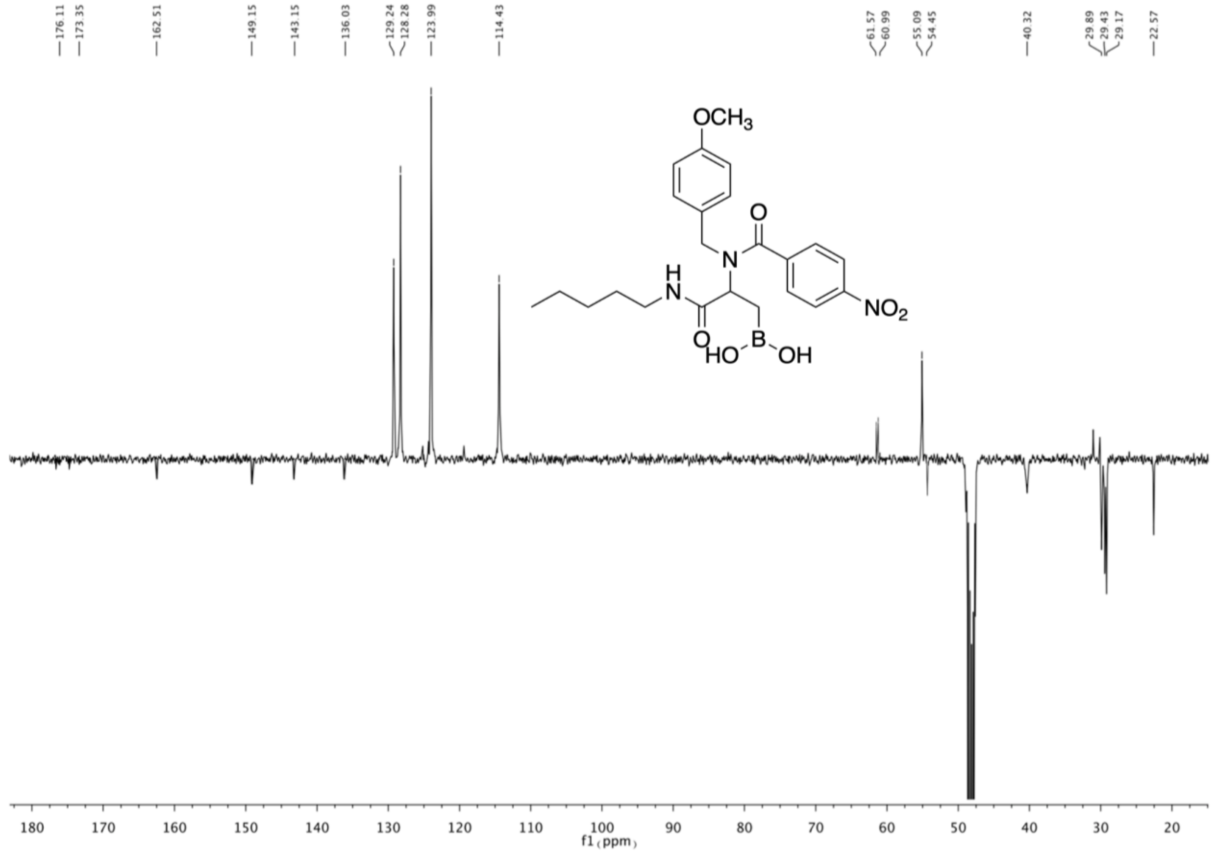

^{1}H NMR (400 MHz, CD_3OD) of compound **6n**

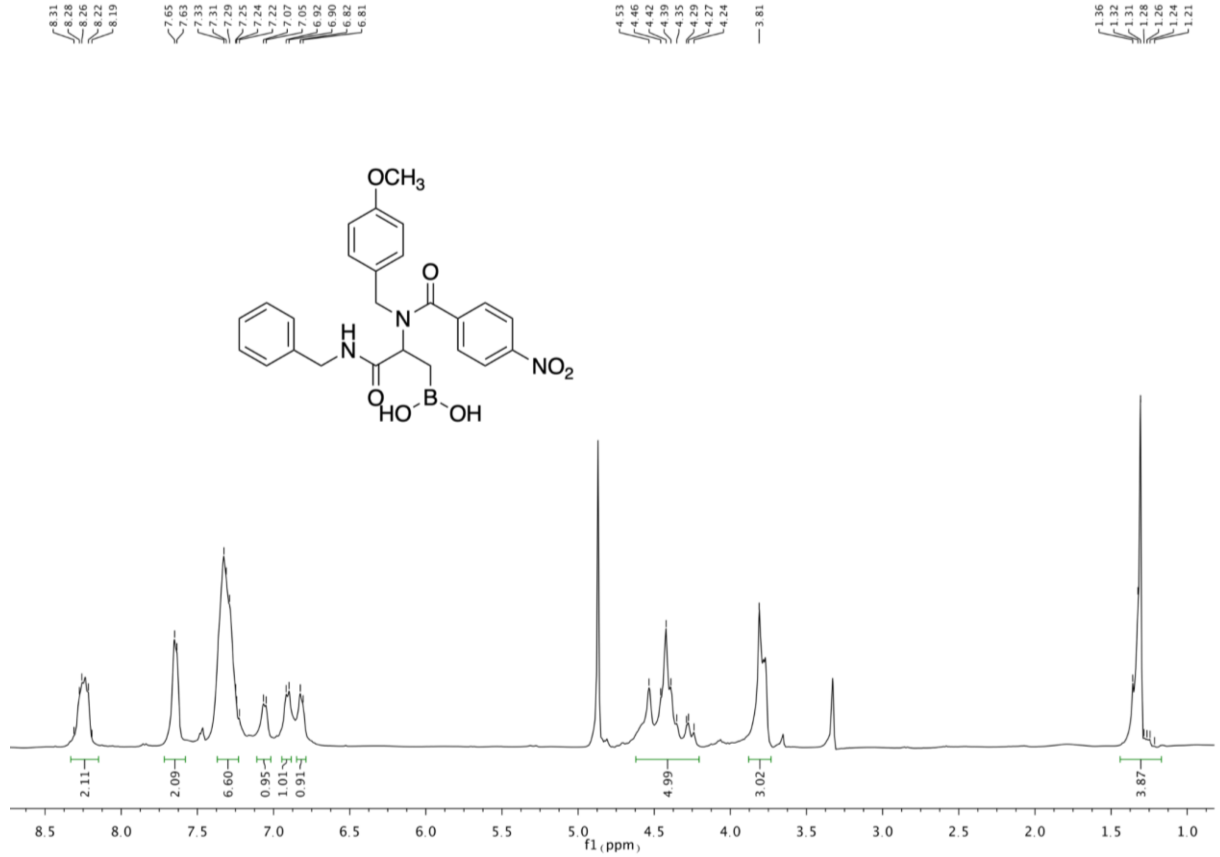

^{13}C NMR (75 MHz, CD_3OD) of compound **6n**

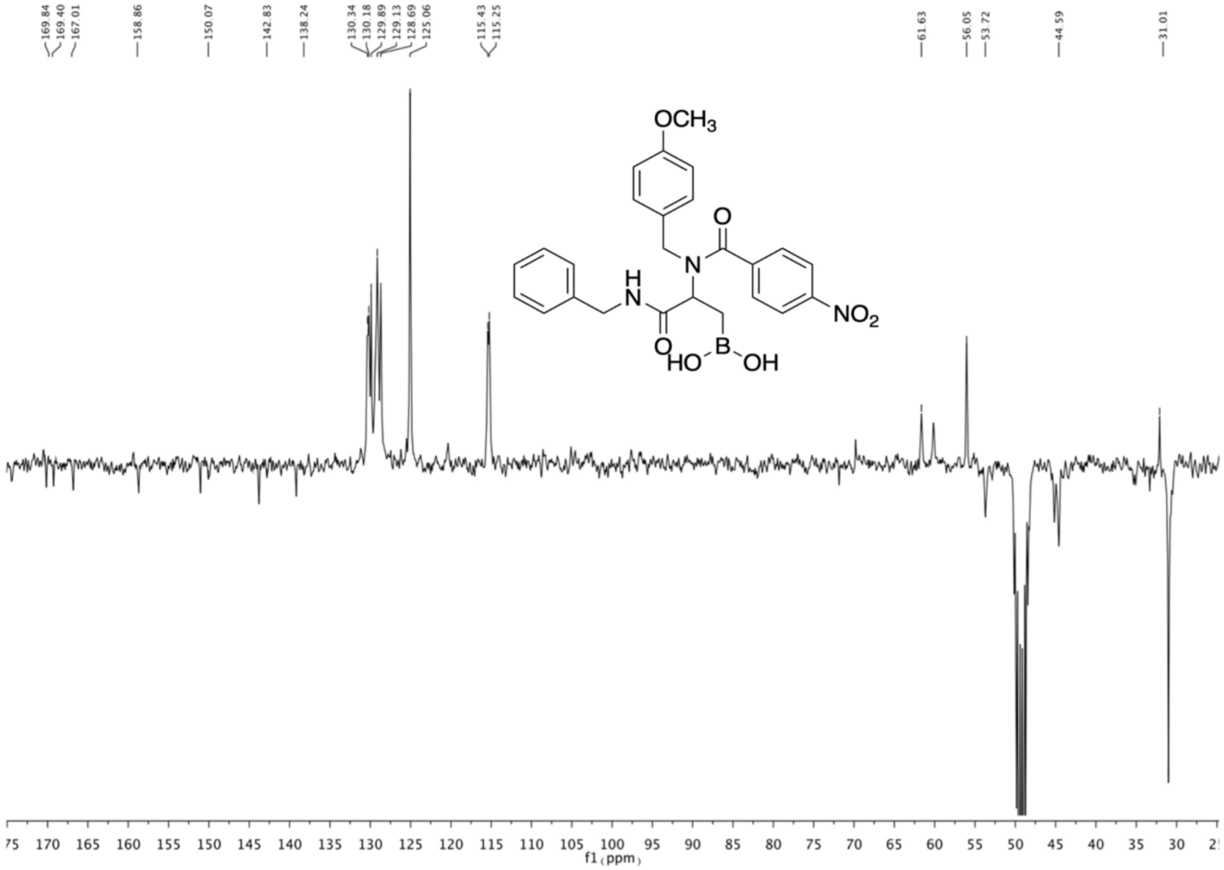

^{1}H NMR (400 MHz, CD_3OD) of compound **6o**

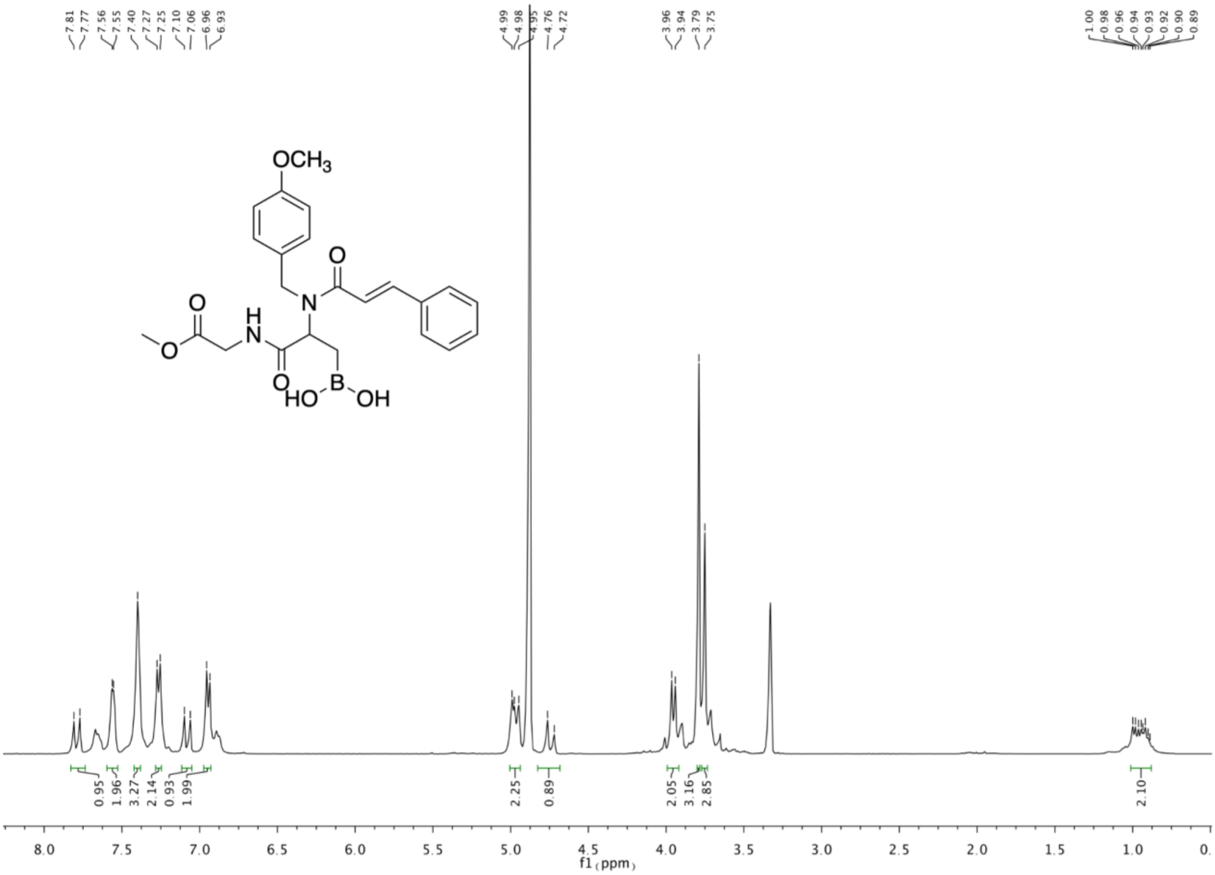

^{13}C NMR (75 MHz, CD_3OD) of compound **6o**

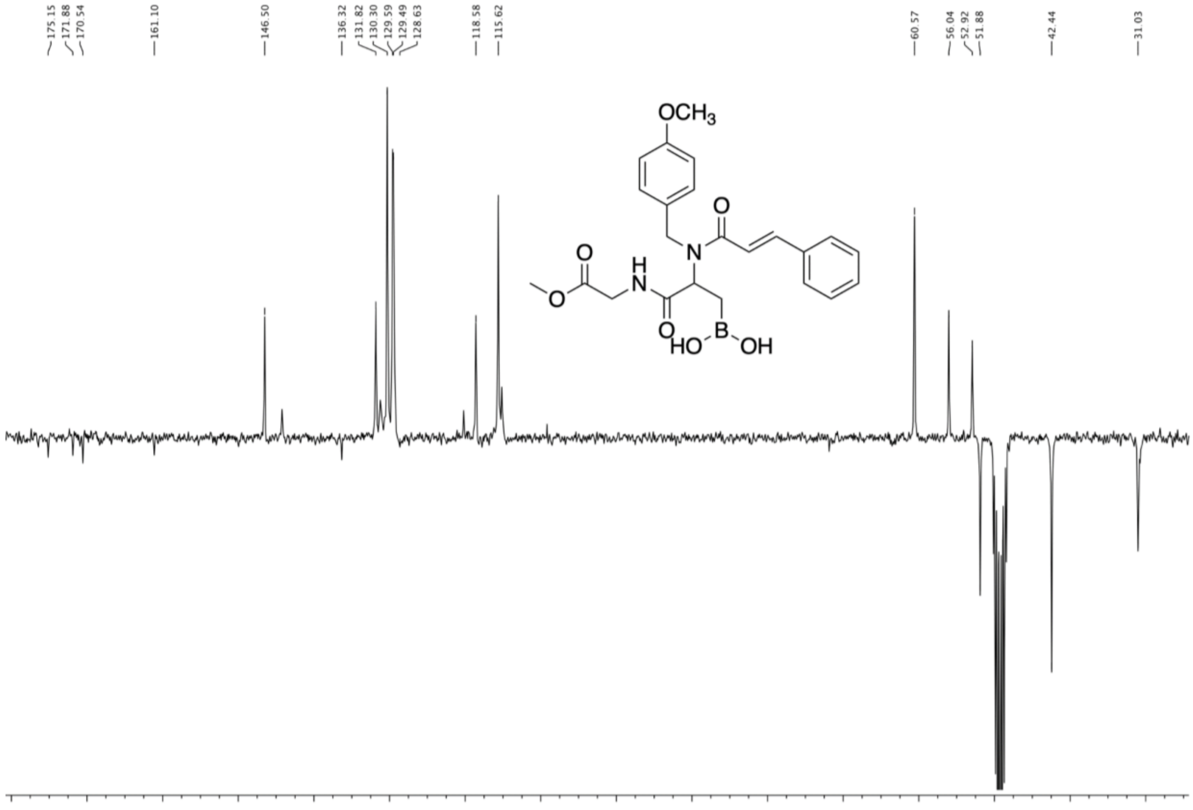

^{1}H NMR (400 MHz, CD_3OD, rotameric mixture) of compound **6p**

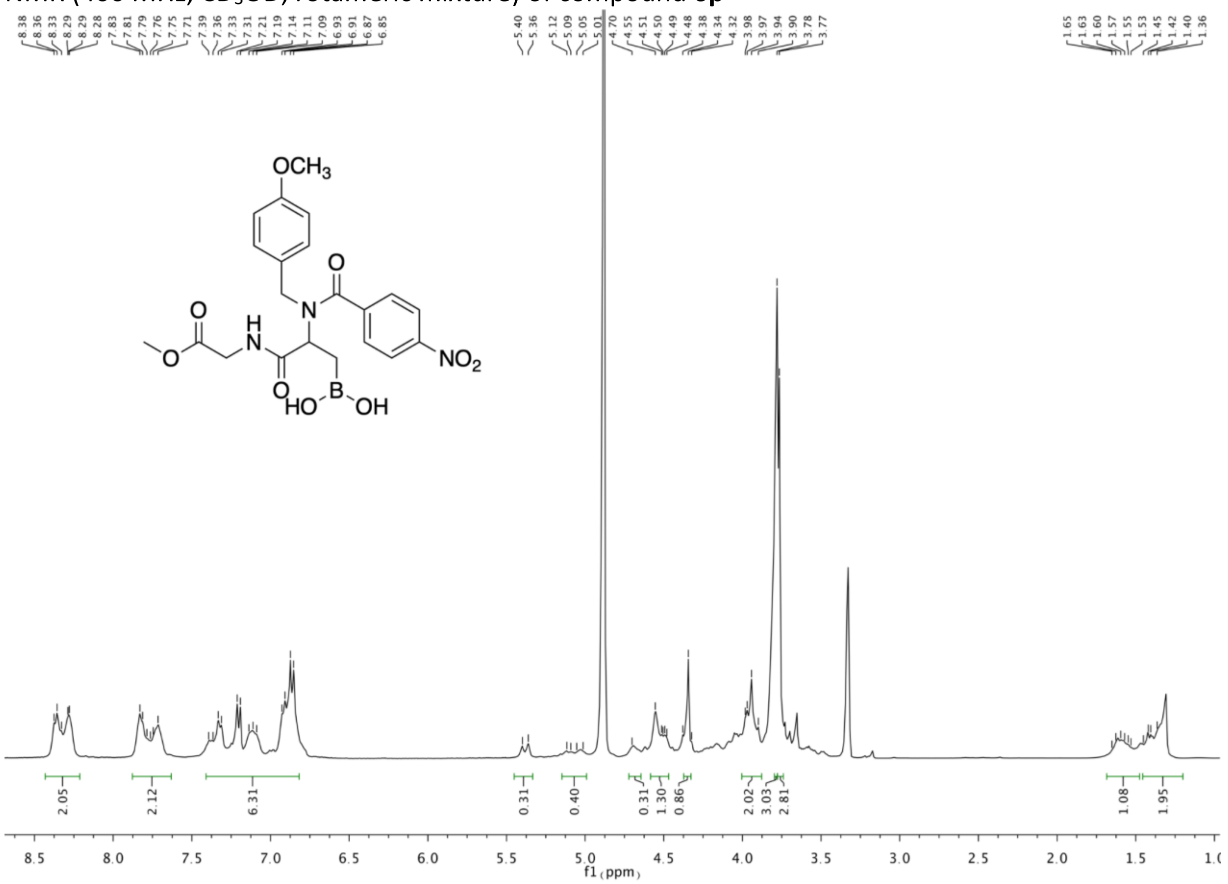

^{13}C NMR (101 MHz, CD_3OD, rotameric mixture) of compound **6p**

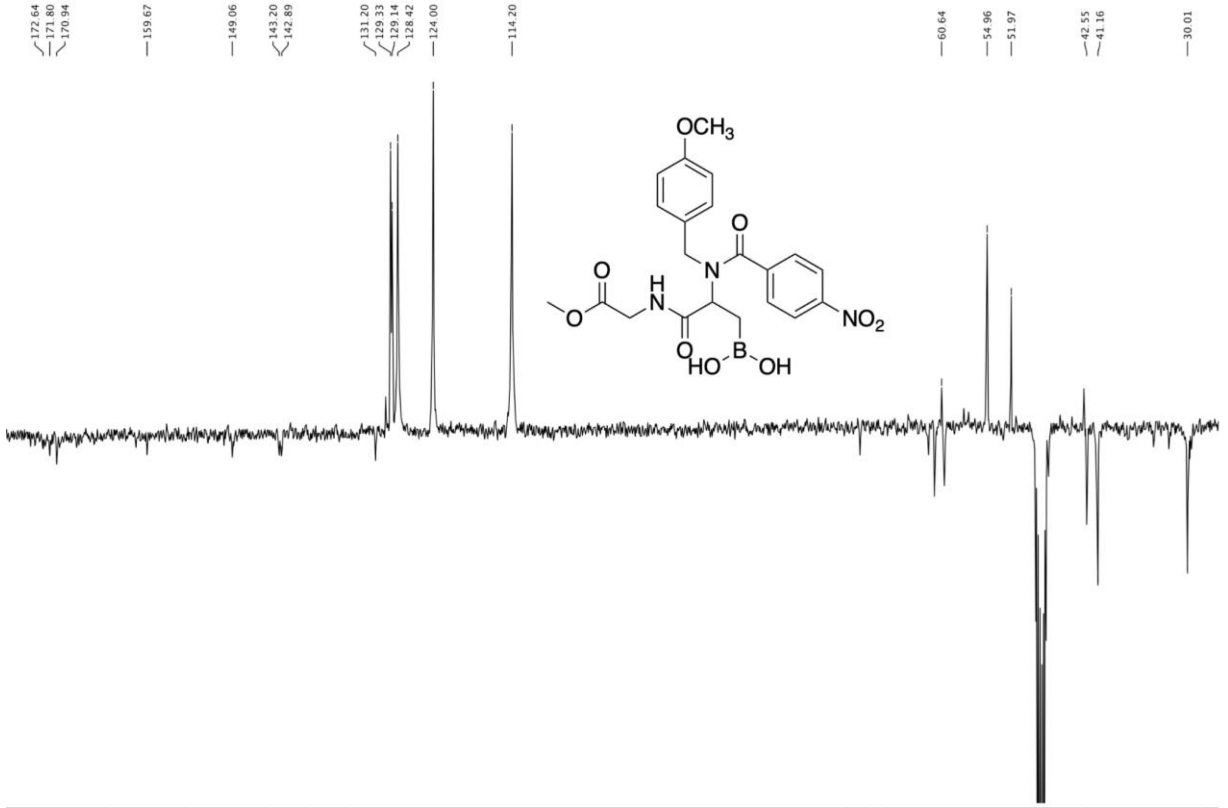

3. ^{1}H and ^{13}C NMR spectra of MIDA-protected Ugi-4C-3CR products (8a-c)

^{1}H NMR (400 MHz, CD_3OD) of compound **8a**

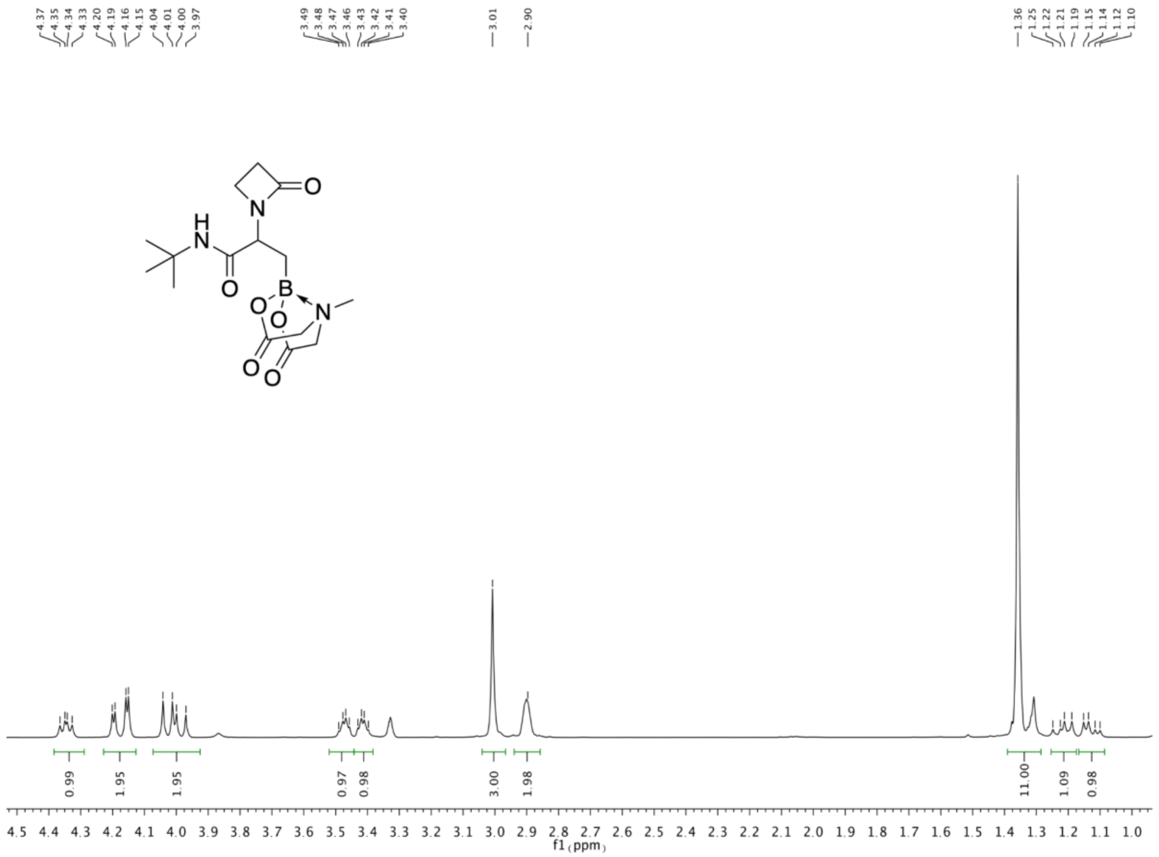

^{13}C NMR (101 MHz, CD_3OD) of compound **8a**

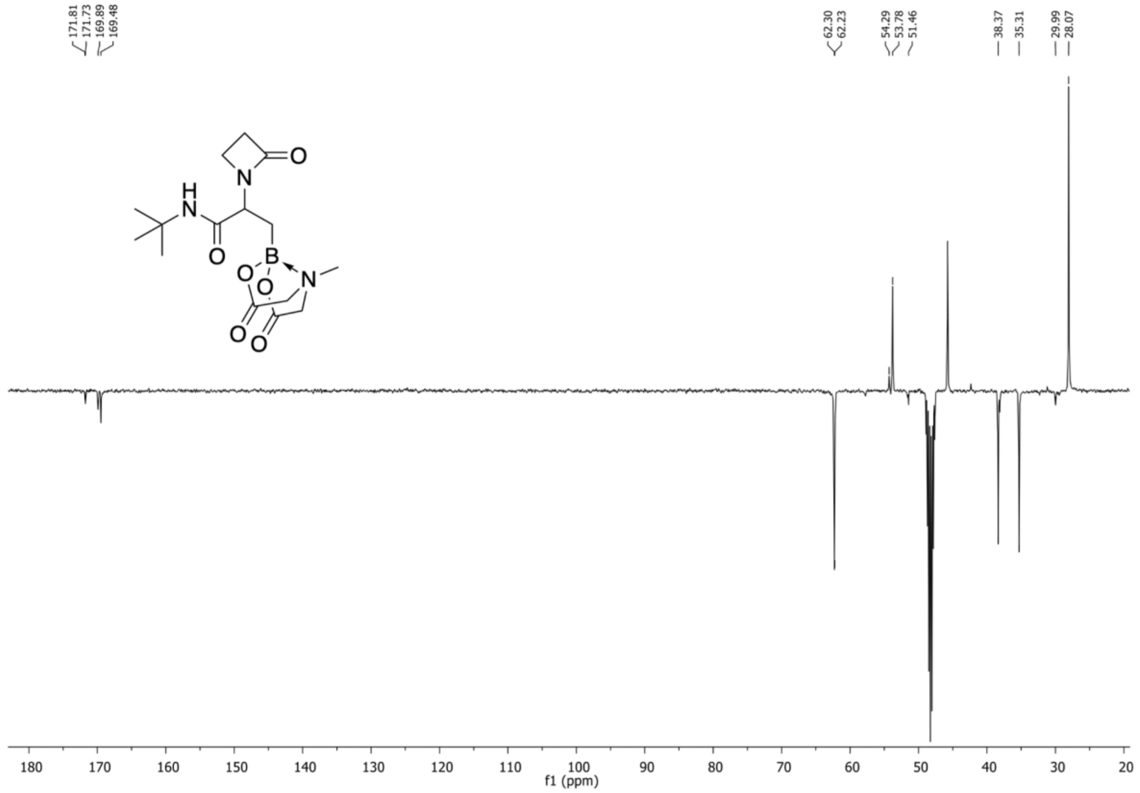

^{1}H NMR (400 MHz, CD_3OD, 6:4 diasteroisomeric mixture of rotamers) of compound **8b**

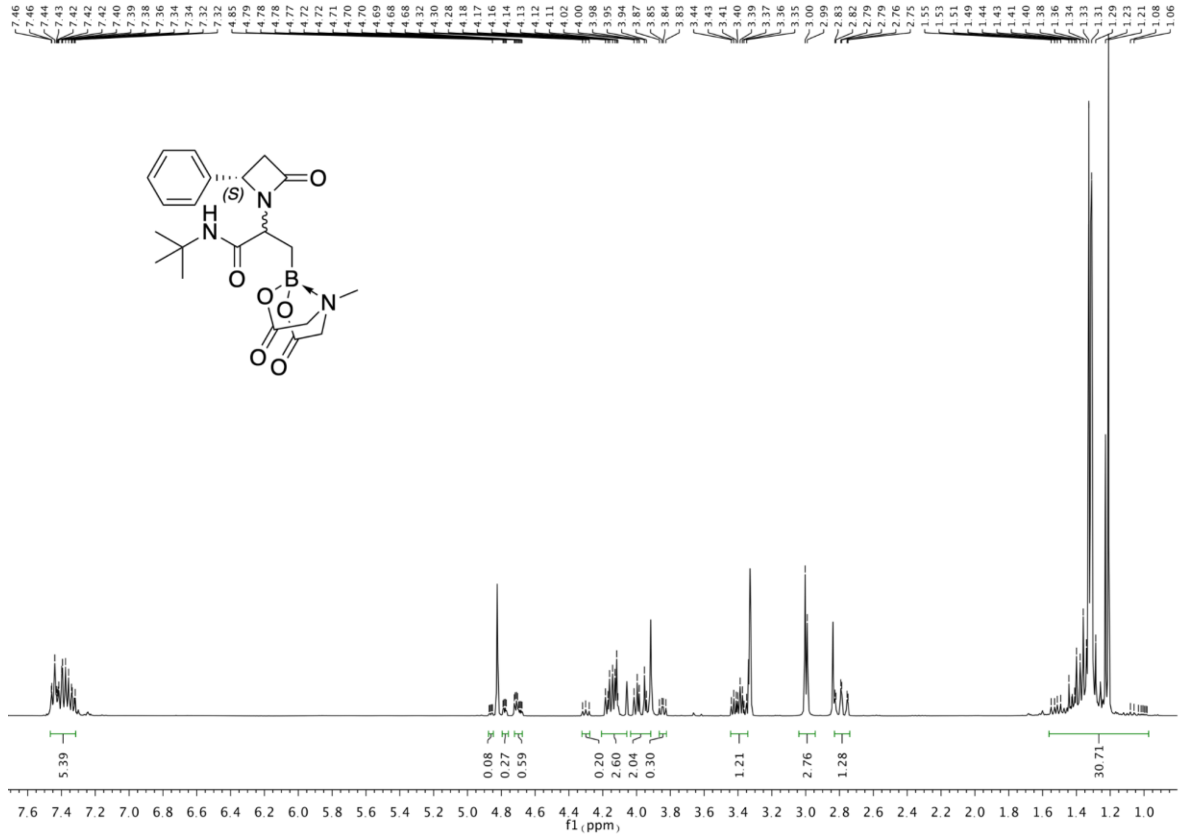

^{13}C NMR (75 MHz, CD_3OD, diasteroisomeric mixture of rotamers) of compound **8b**

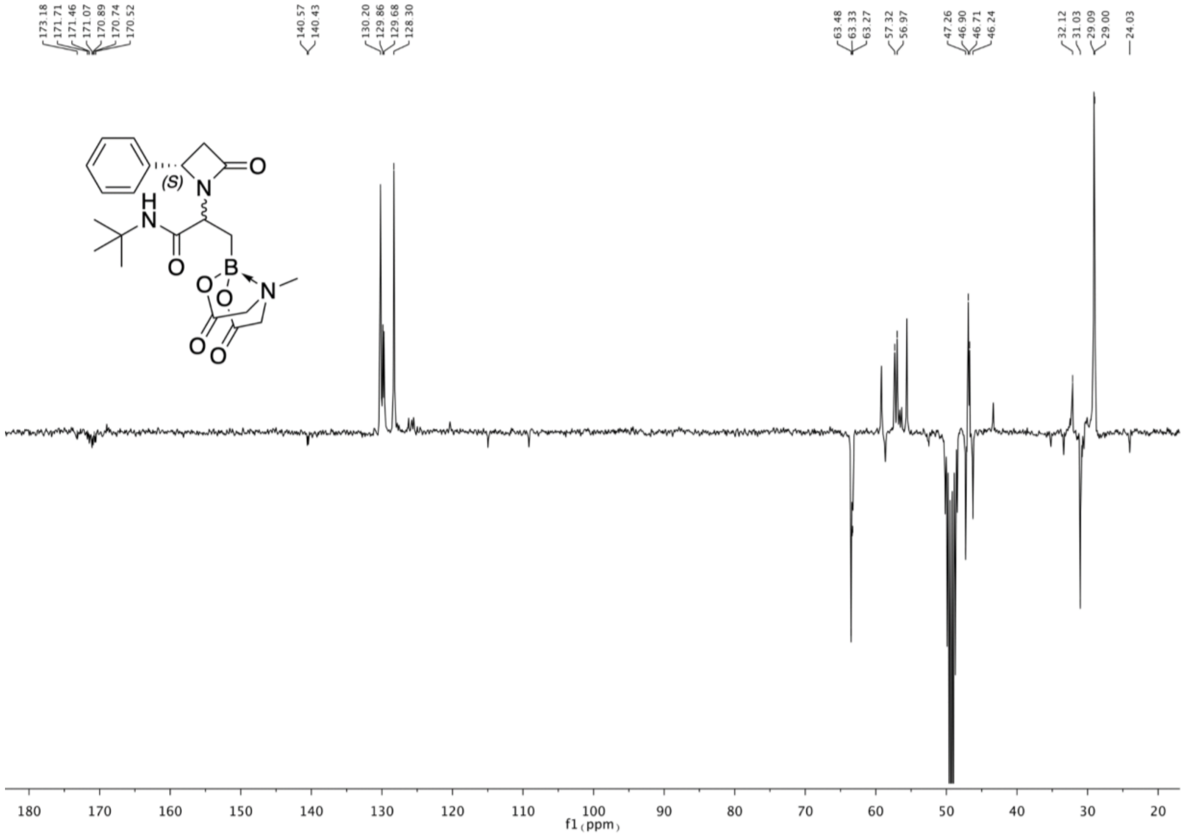

^{1}H NMR (400 MHz, 115°C, DMSO, 1:1 diasteroisomeric mixture) of compound **8c**

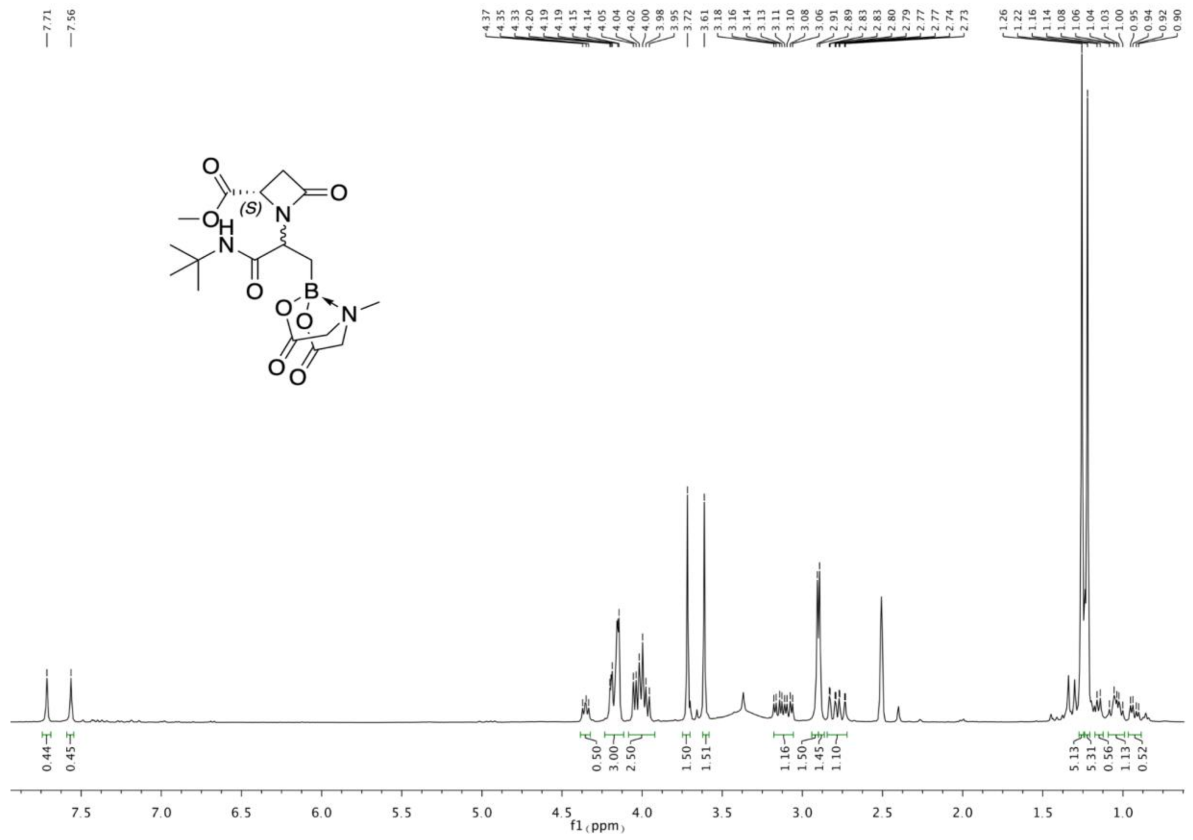

^{3}C NMR (75 MHz, 25°C, DMSO, 1:1 diasteroisomeric mixture) of compound **8c**

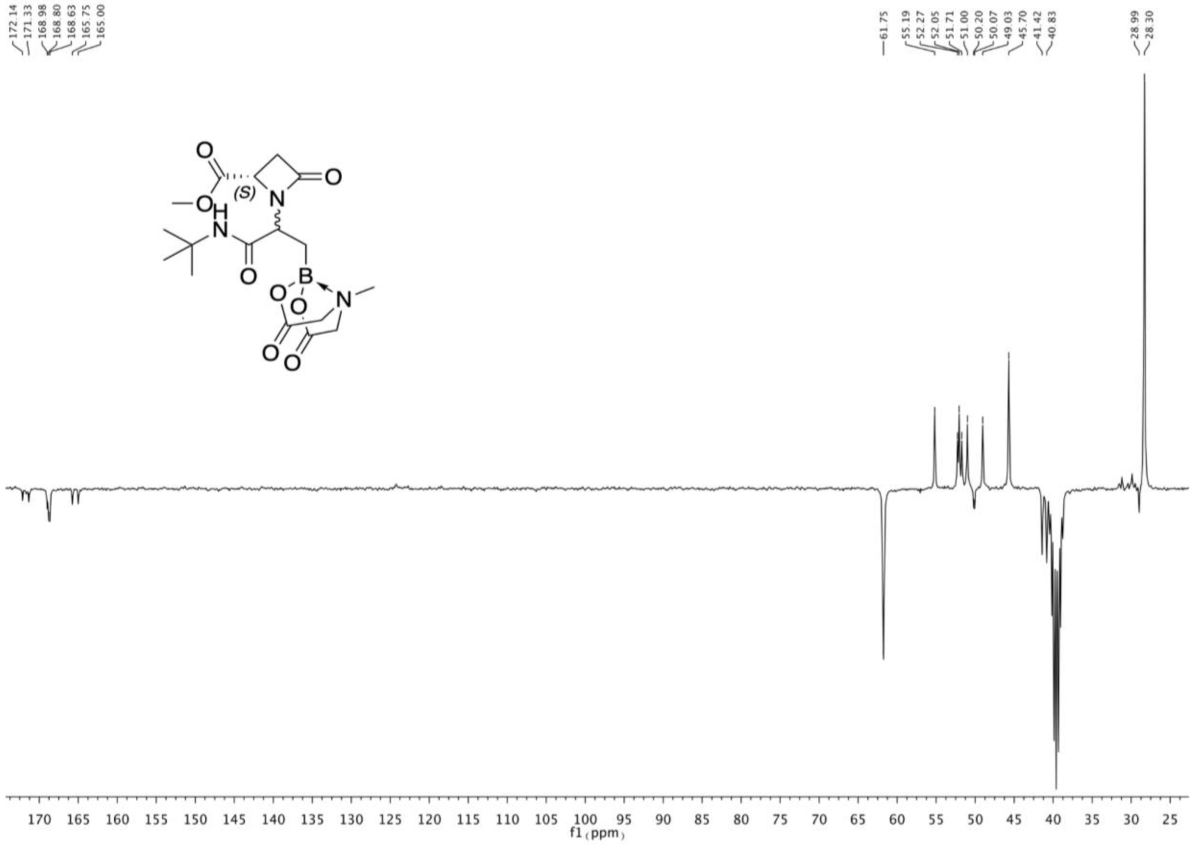

4. ^{1}H and ^{13}C NMR spectra of Ugi-4C-3CR products (9a-c)

^{1}H NMR (400 MHz, CD_3OD) of compound **9a**

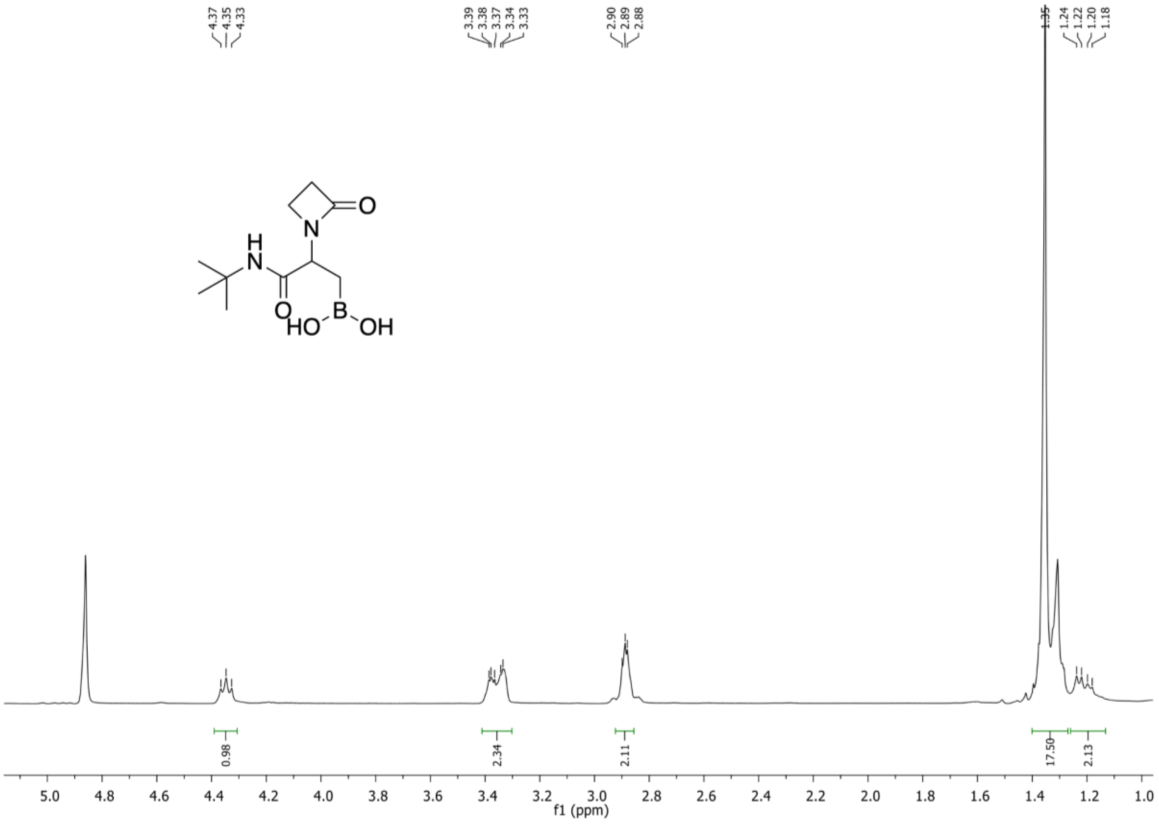

^{13}C NMR (101 MHz, CD_3OD) of compound **9a**

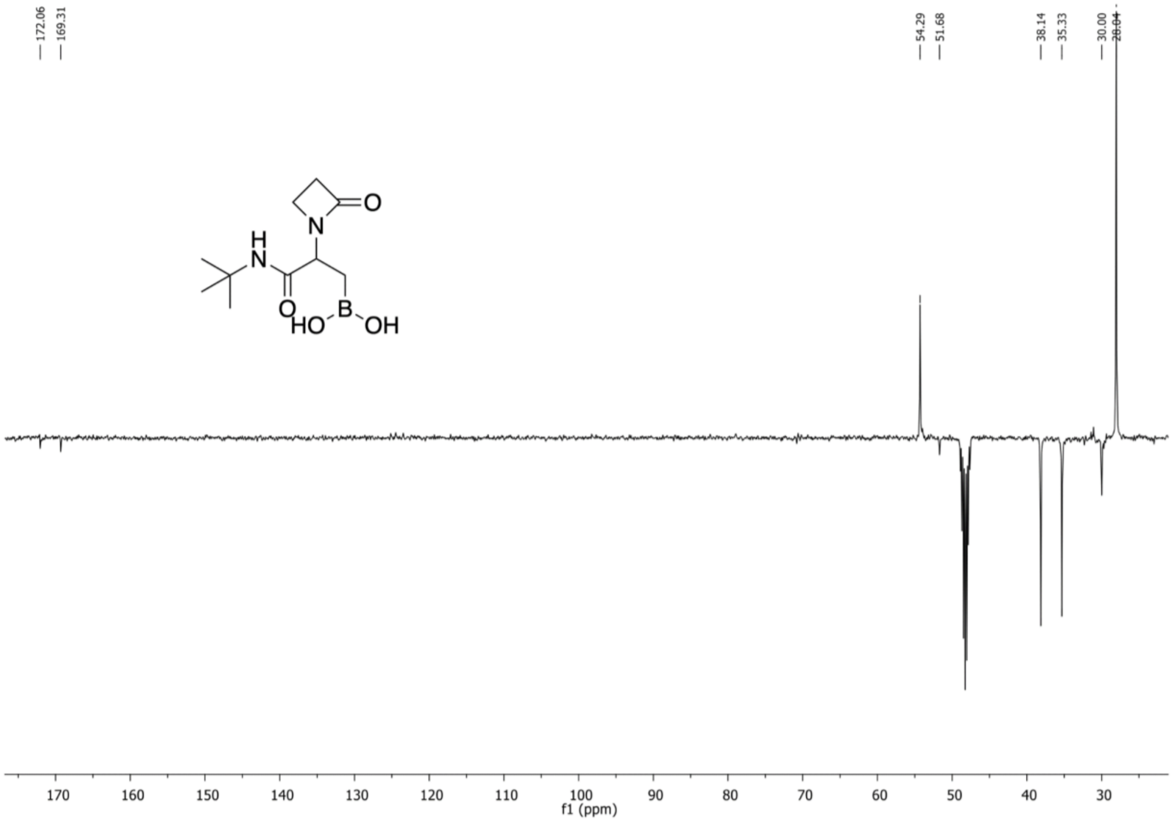

^{1}H NMR (400 MHz, 115°C, DMSO, 6:4 diastereoisomeric mixture) of compound **9b**

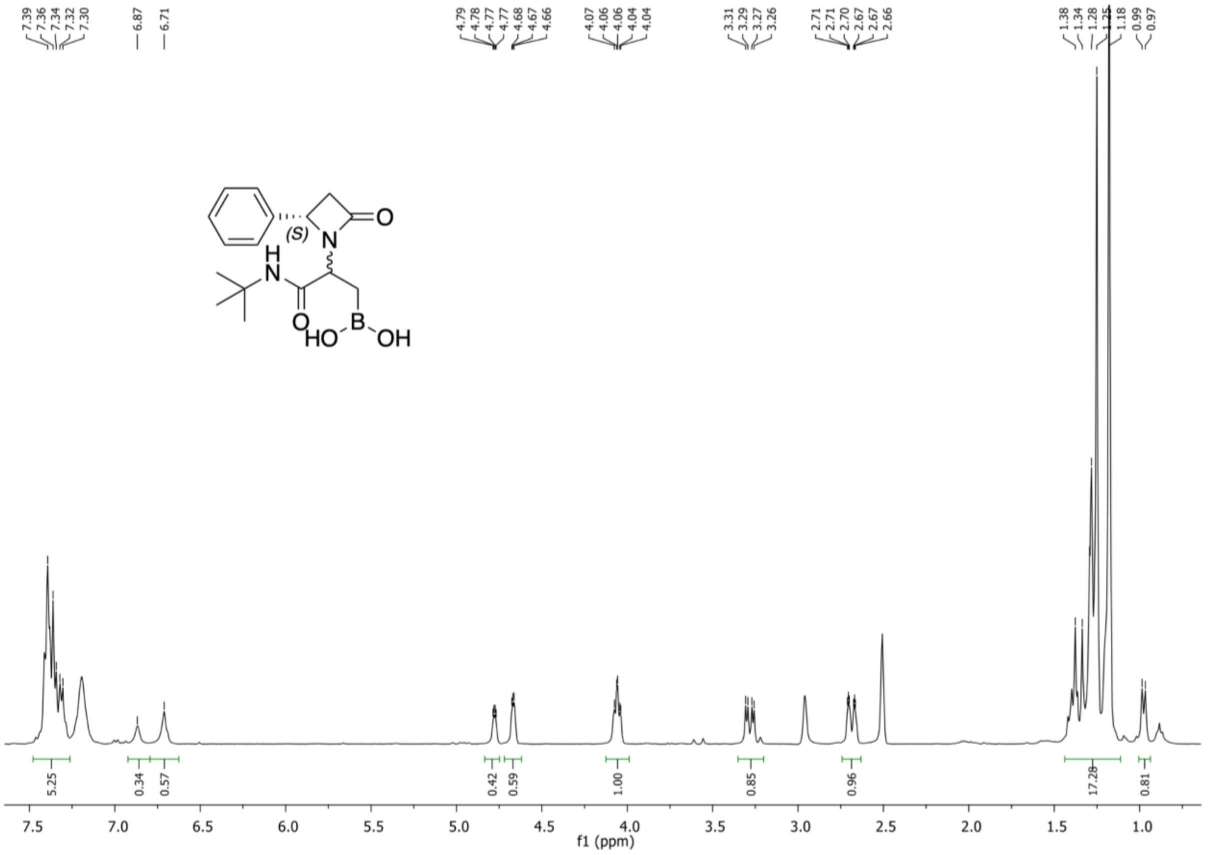

^{13}C NMR (75 MHz, 25°C, DMSO, 6:4 diastereoisomeric mixture) of compound **9b**

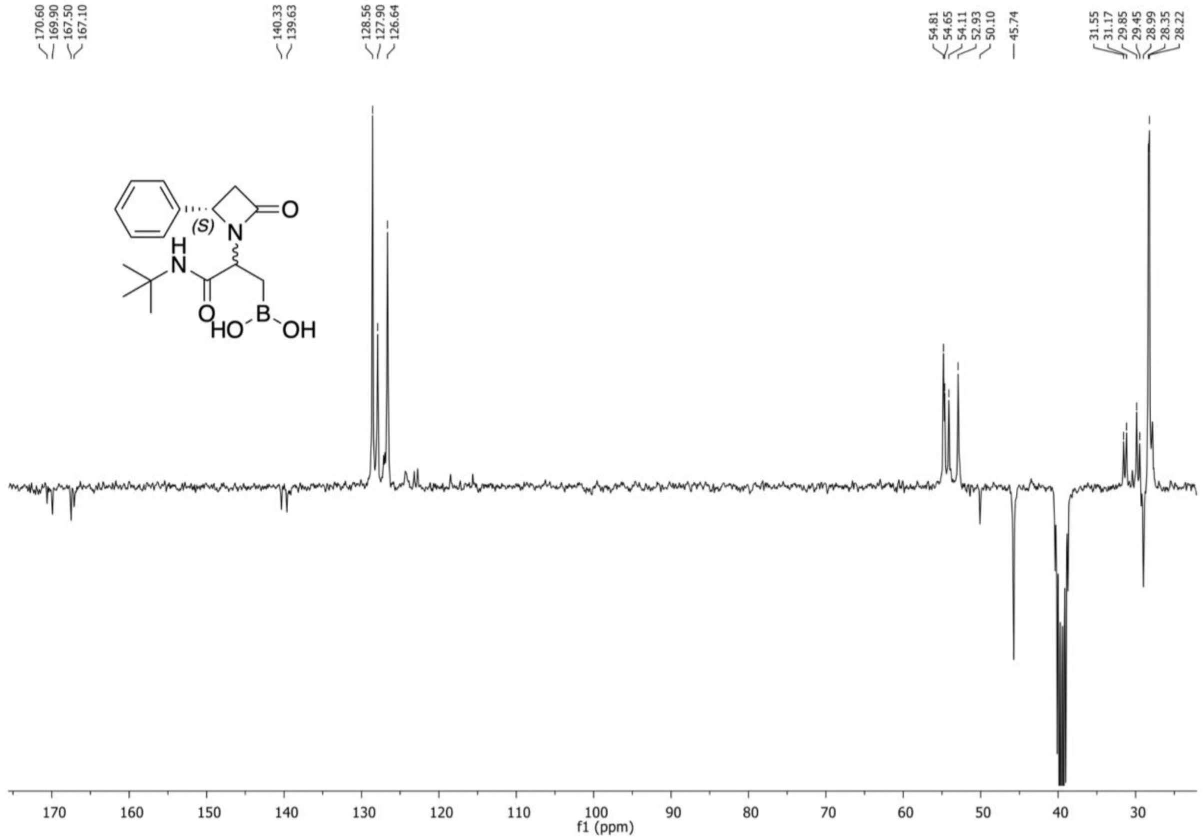

^{1}H NMR (400 MHz, CD_3OD, 1:2 diastereoisomeric mixture) of compound **9c**

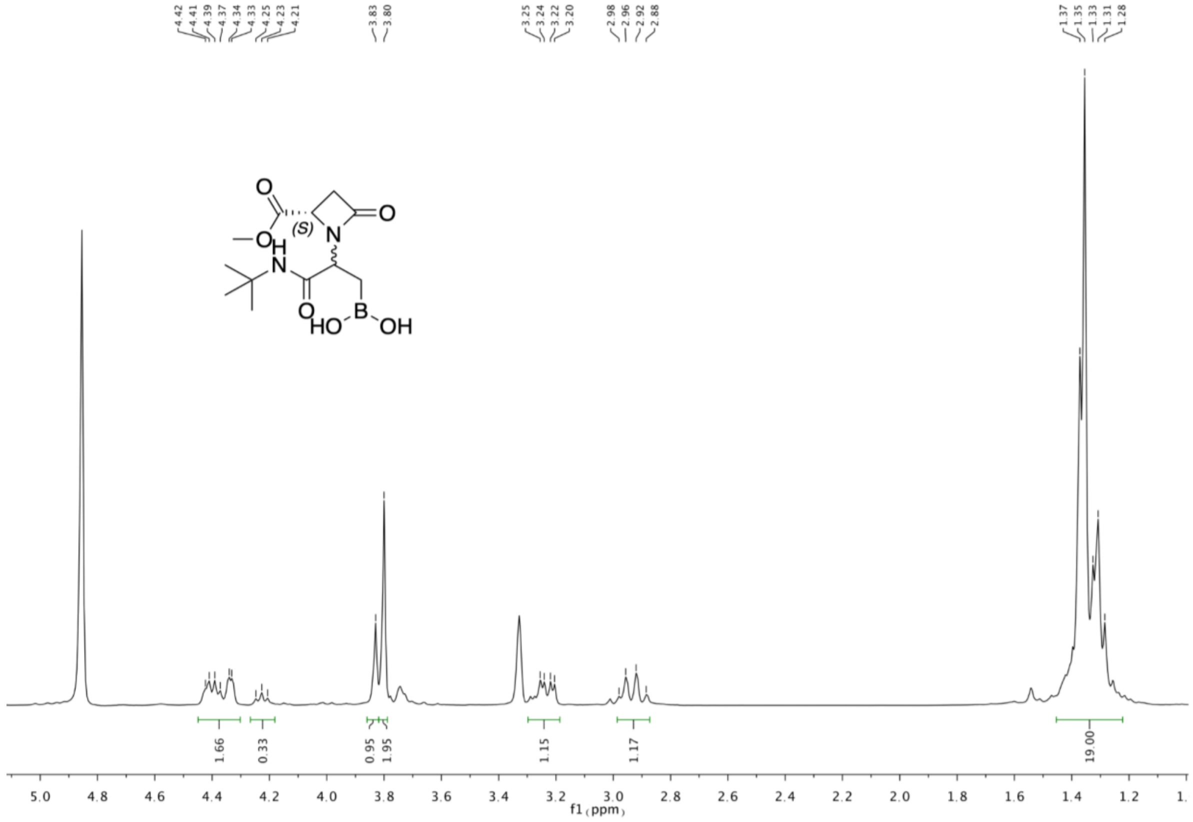

^{13}C NMR (75 MHz, CD_3OD, 1:2 diastereoisomeric mixture) of compound **9c**

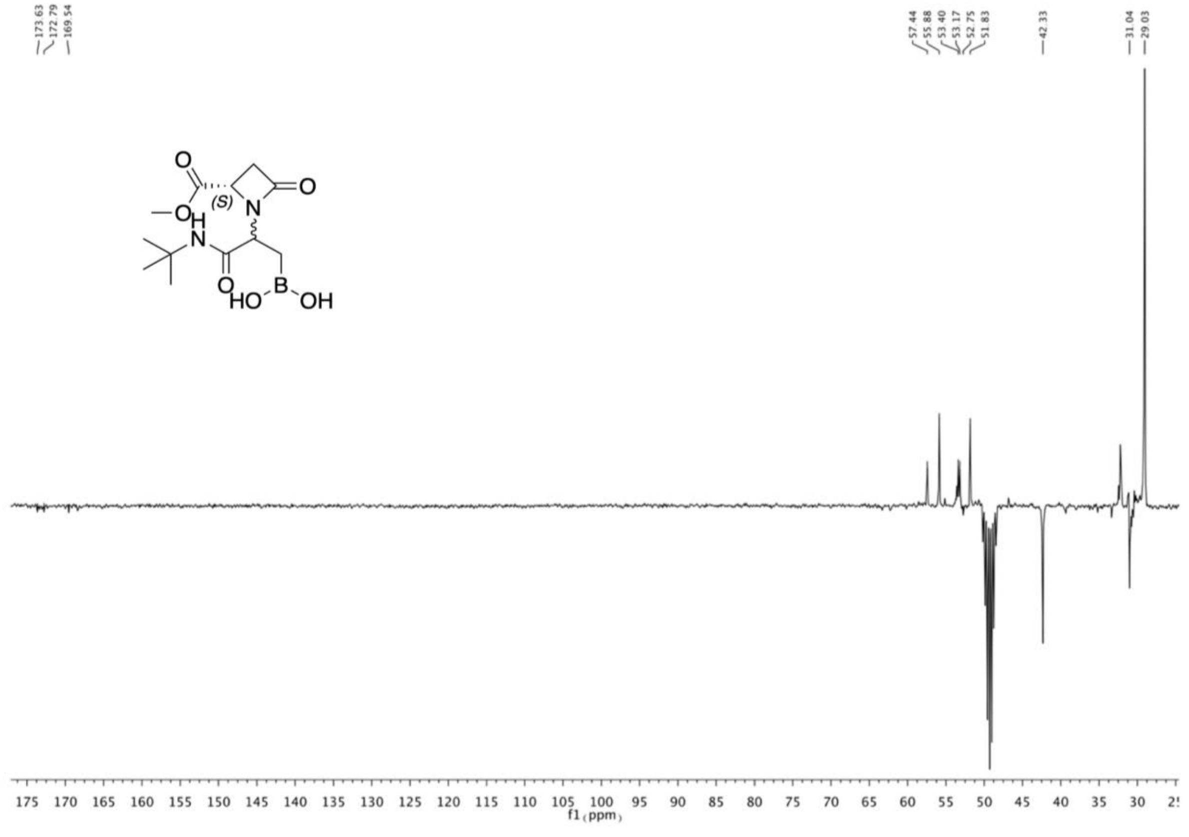

5. ¹H and ¹³C NMR spectra of Van Leusen-3CR product (10)

¹H NMR (300 MHz, $(CD_3)_2CO$) of compound **10**

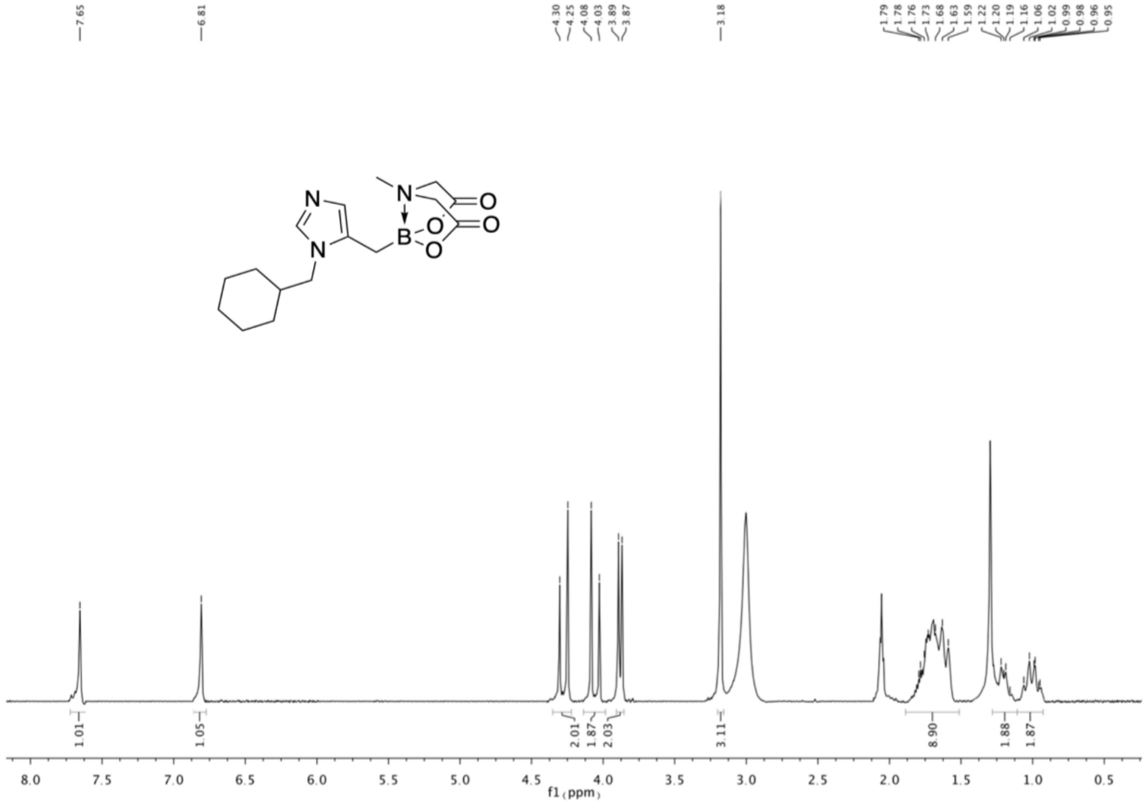

¹³C NMR (101 MHz, CD_3OD) of compound **10**

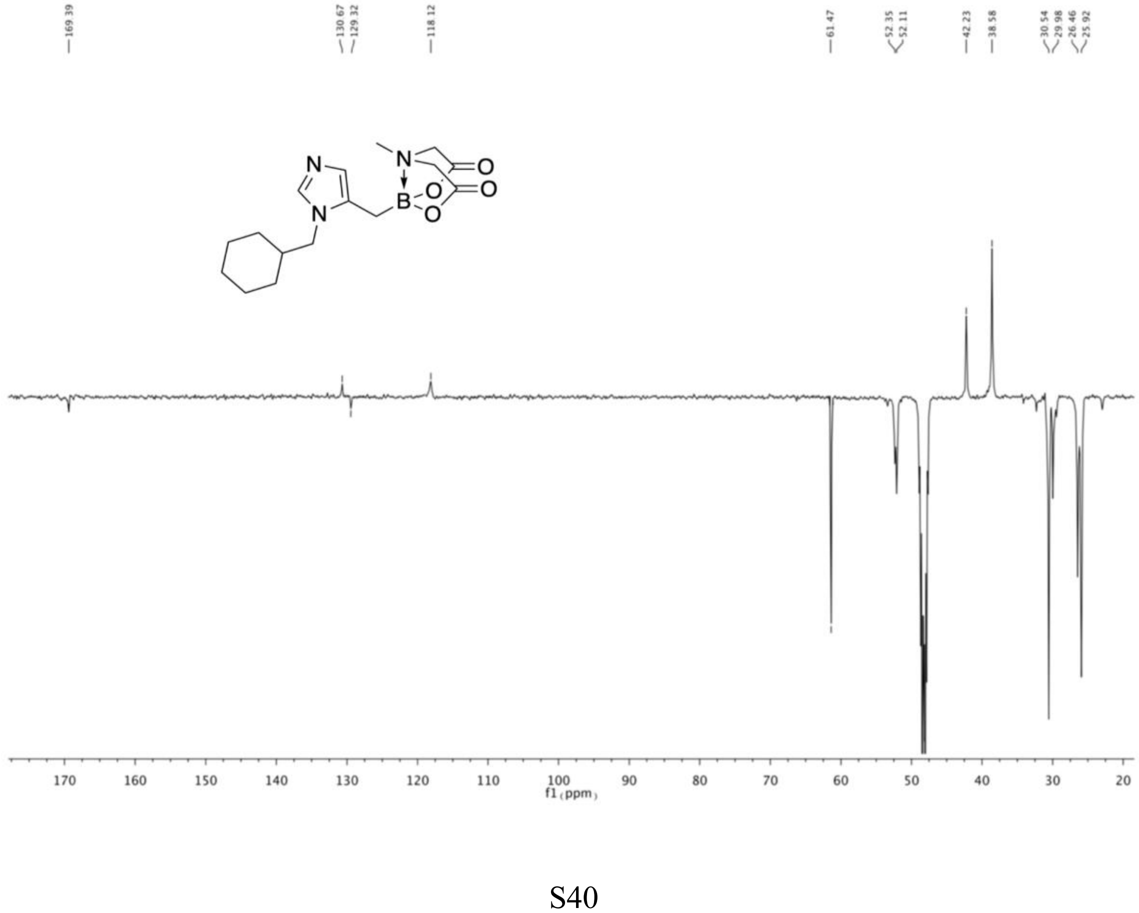

6. ^{11}B NMR spectra of Ugi-4CR products (6a-p)

^{11}B NMR (128 MHz, CD_3CN) of compound **6a**

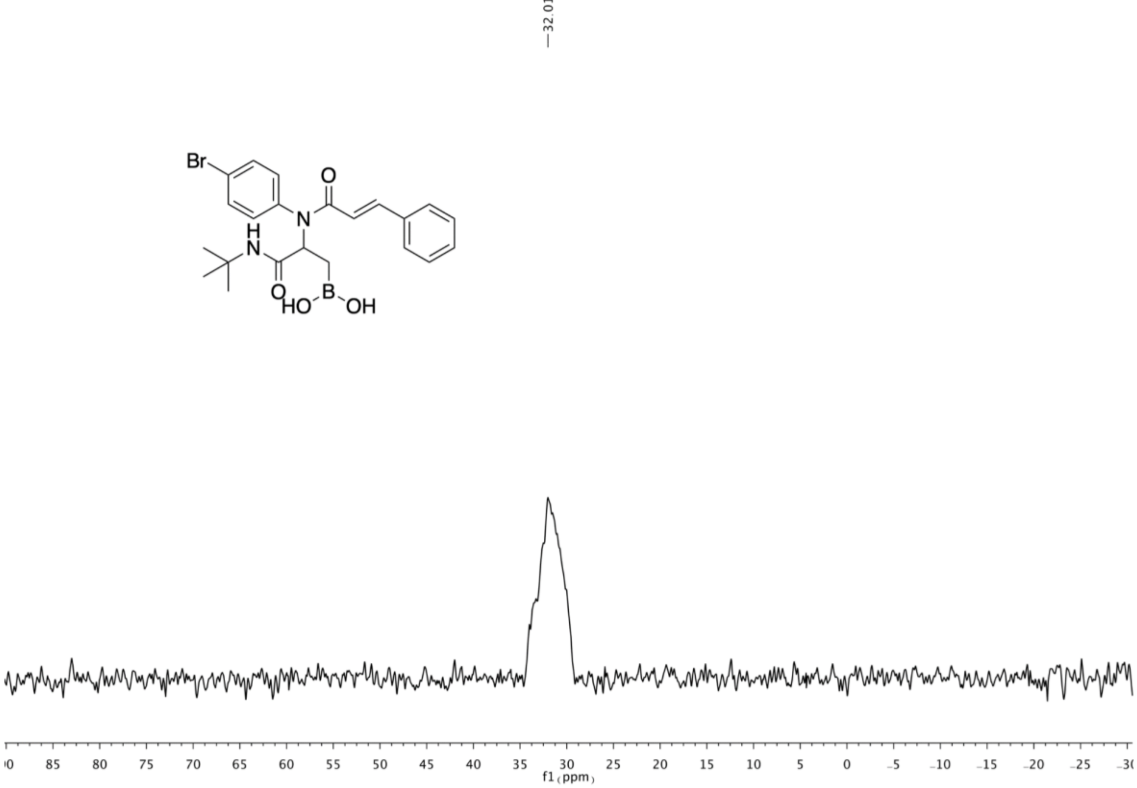

^{11}B NMR (128 MHz, CD_3CN) of compound **6b**

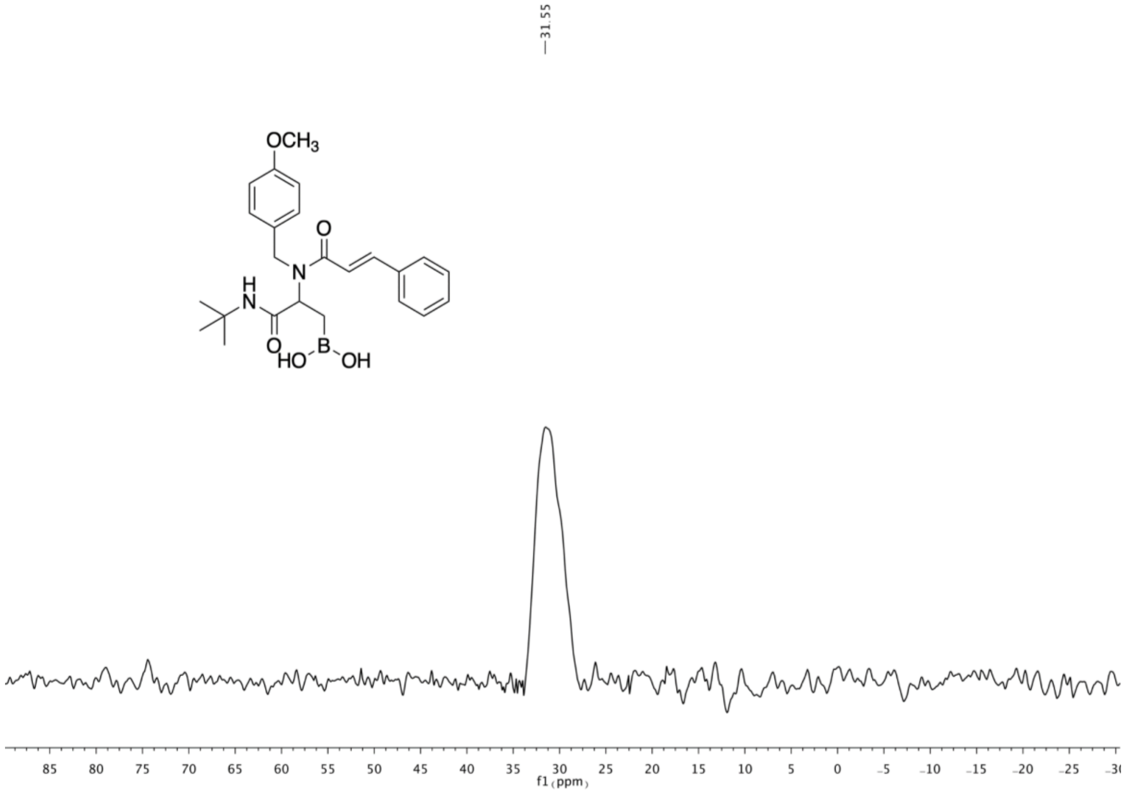

^{11}B NMR (128 MHz, CD_3CN) of compound **6c**

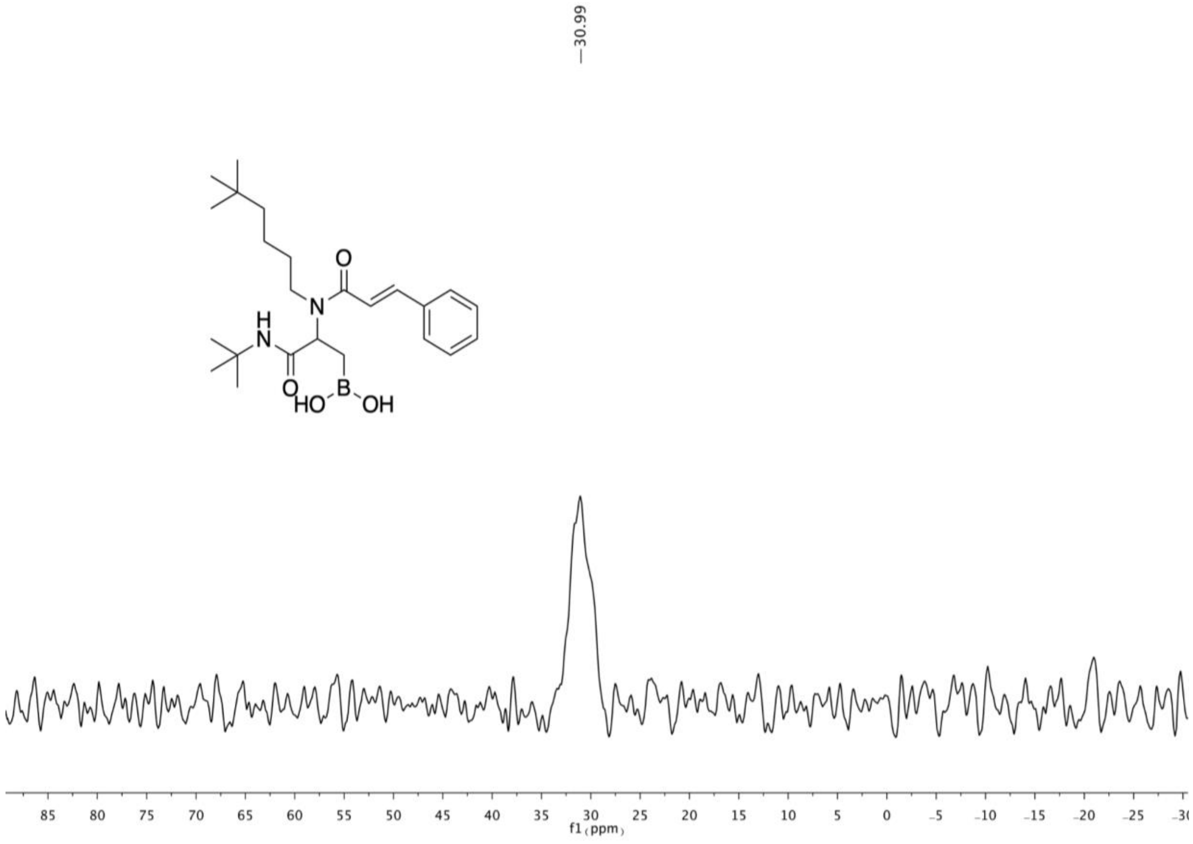

^{11}B NMR (128 MHz, CD_3CN) of compound **6d**

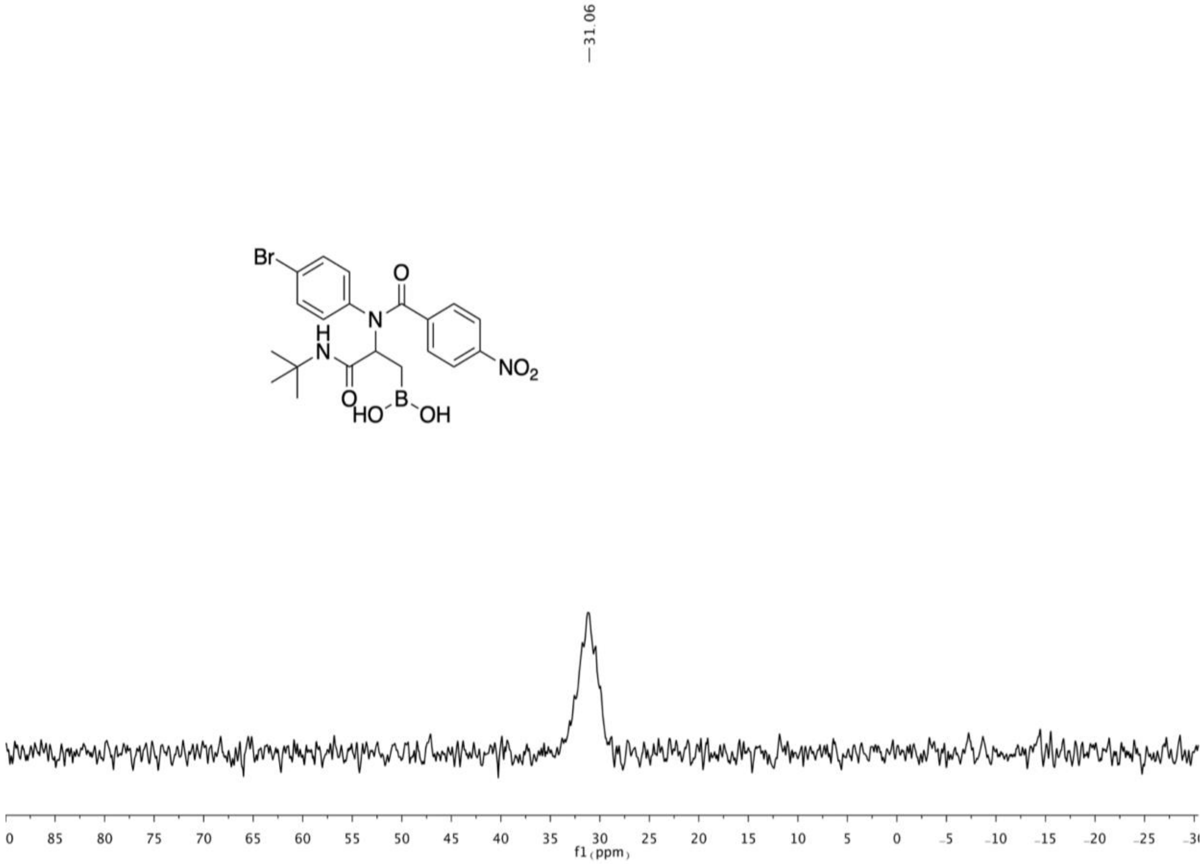

^{11}B NMR (128 MHz, CD_3CN) of compound **6e**

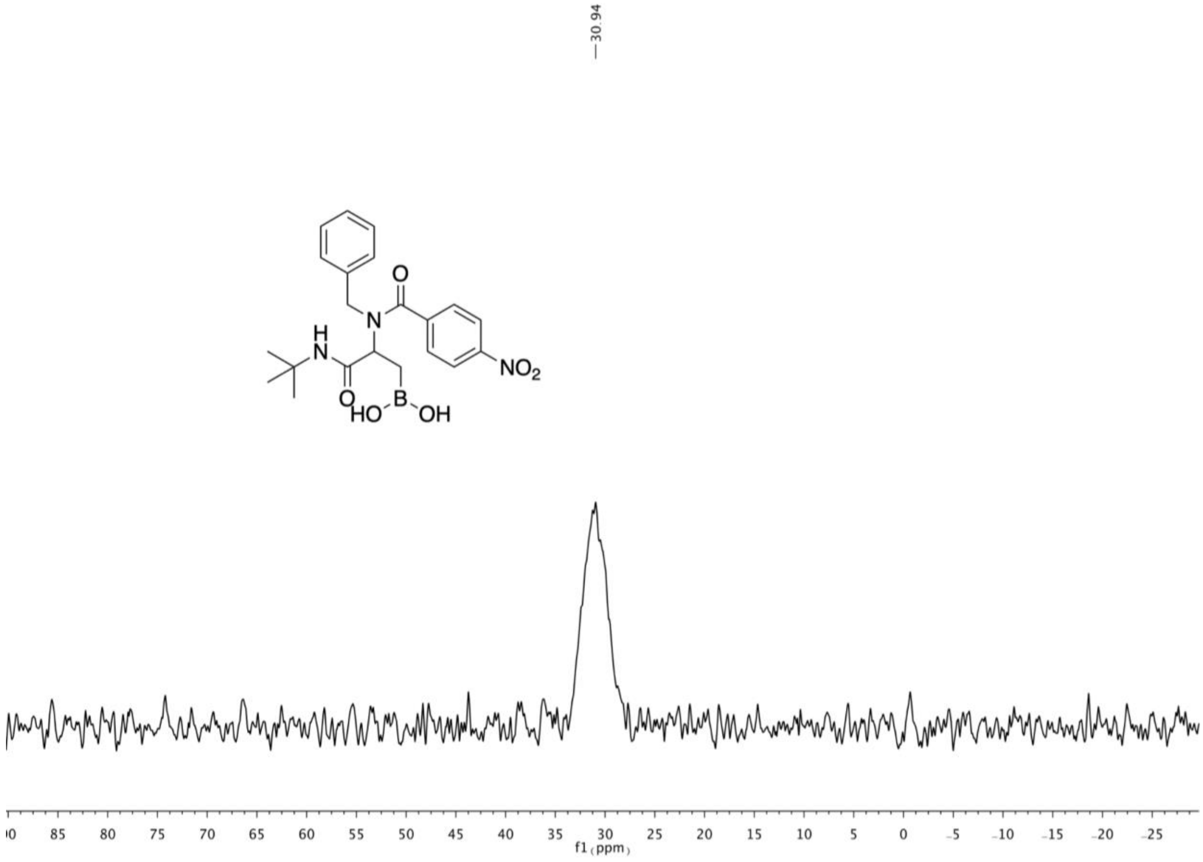

^{11}B NMR (128 MHz, CD_3CN) of compound **6f**

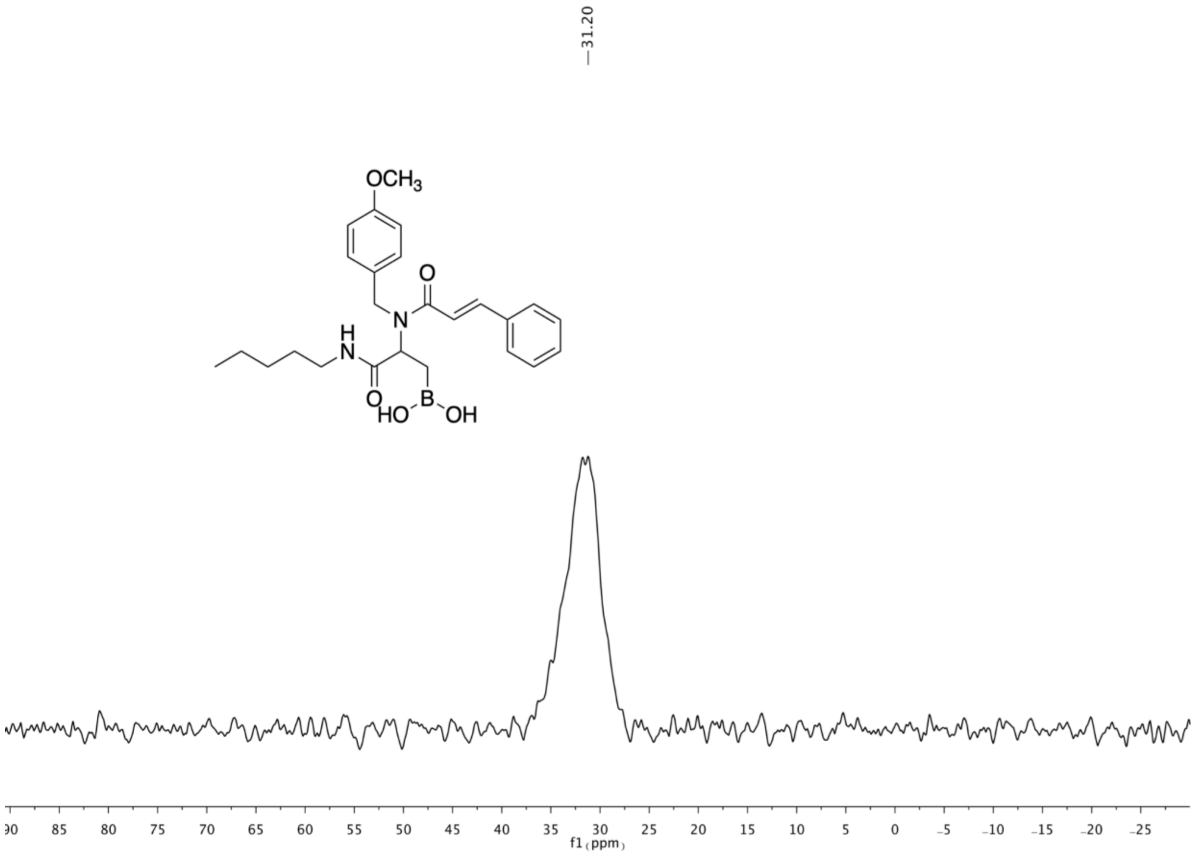

^{11}B NMR (128 MHz, CD_3CN) of compound **6g**

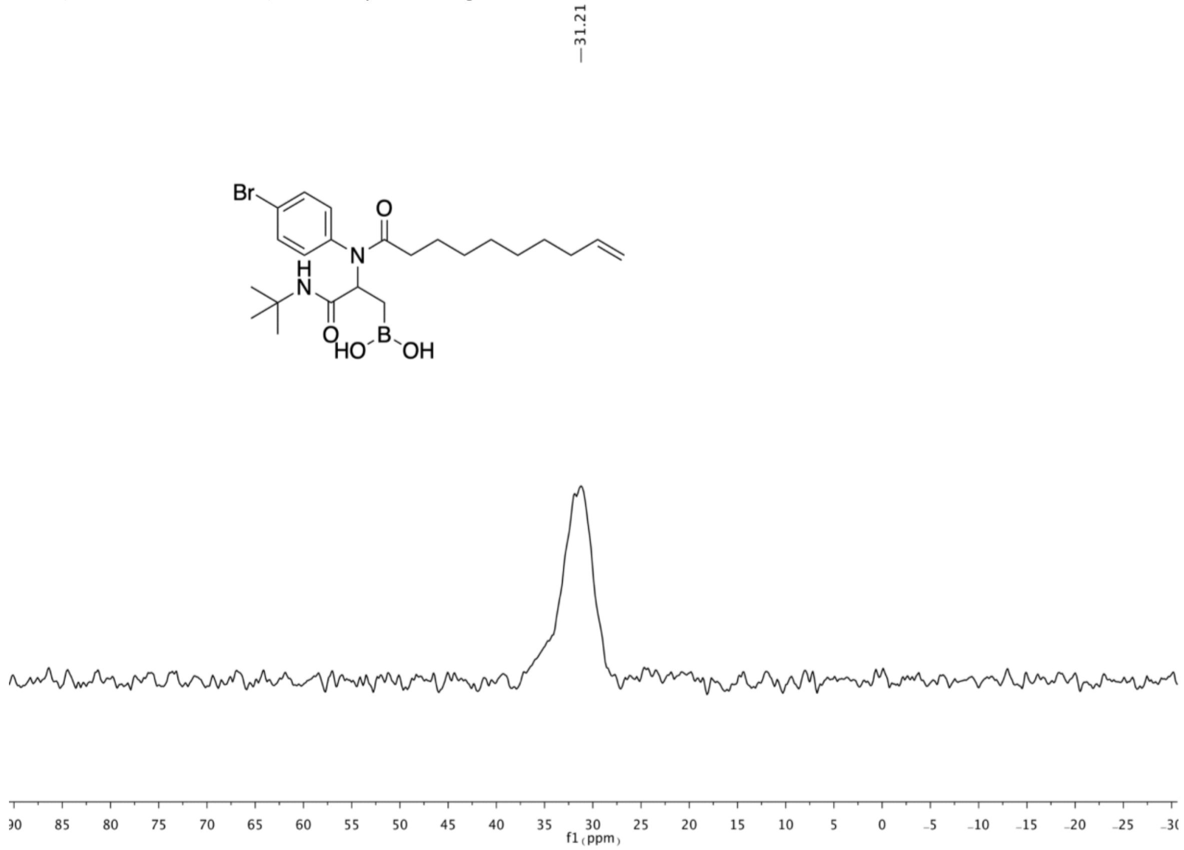

^{11}B NMR (128 MHz, CD_3CN) of compound **6h**

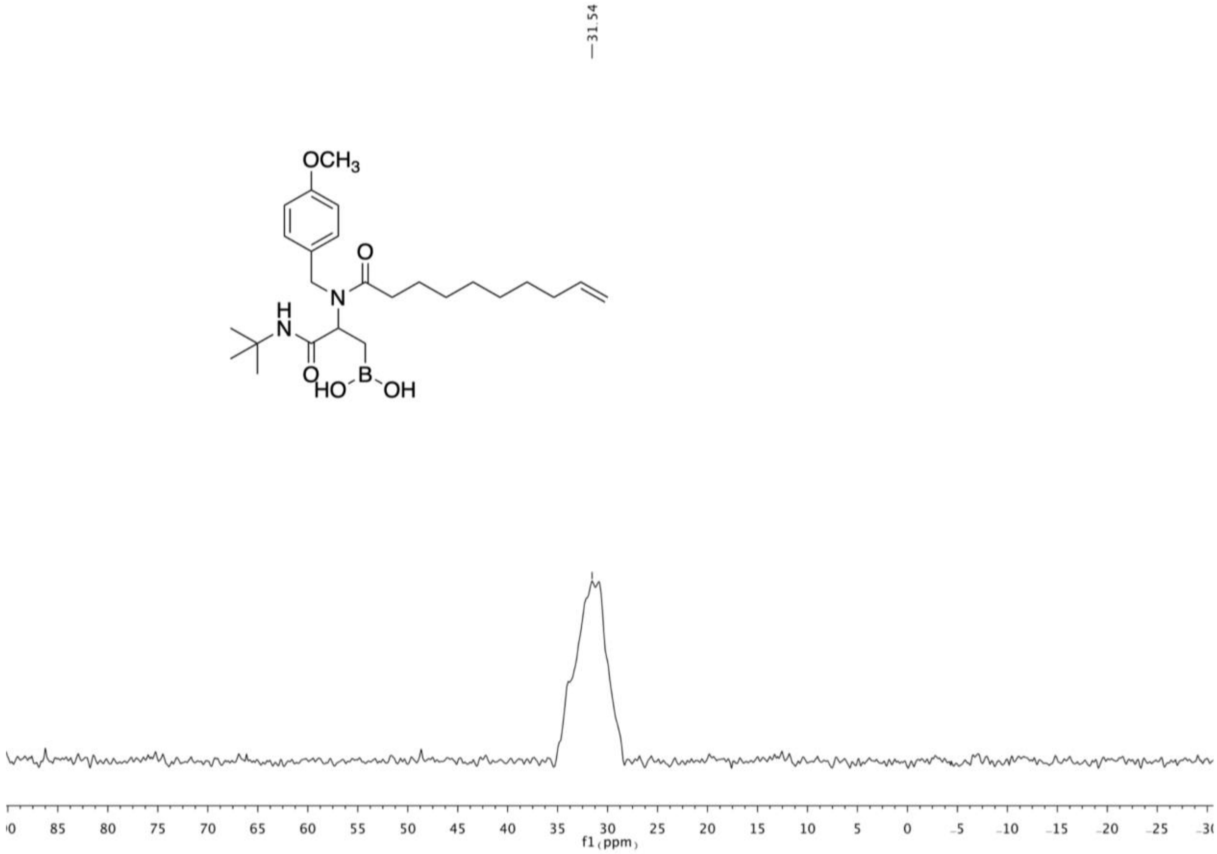

^{11}B NMR (128 MHz, CD_3CN) of compound **6i**

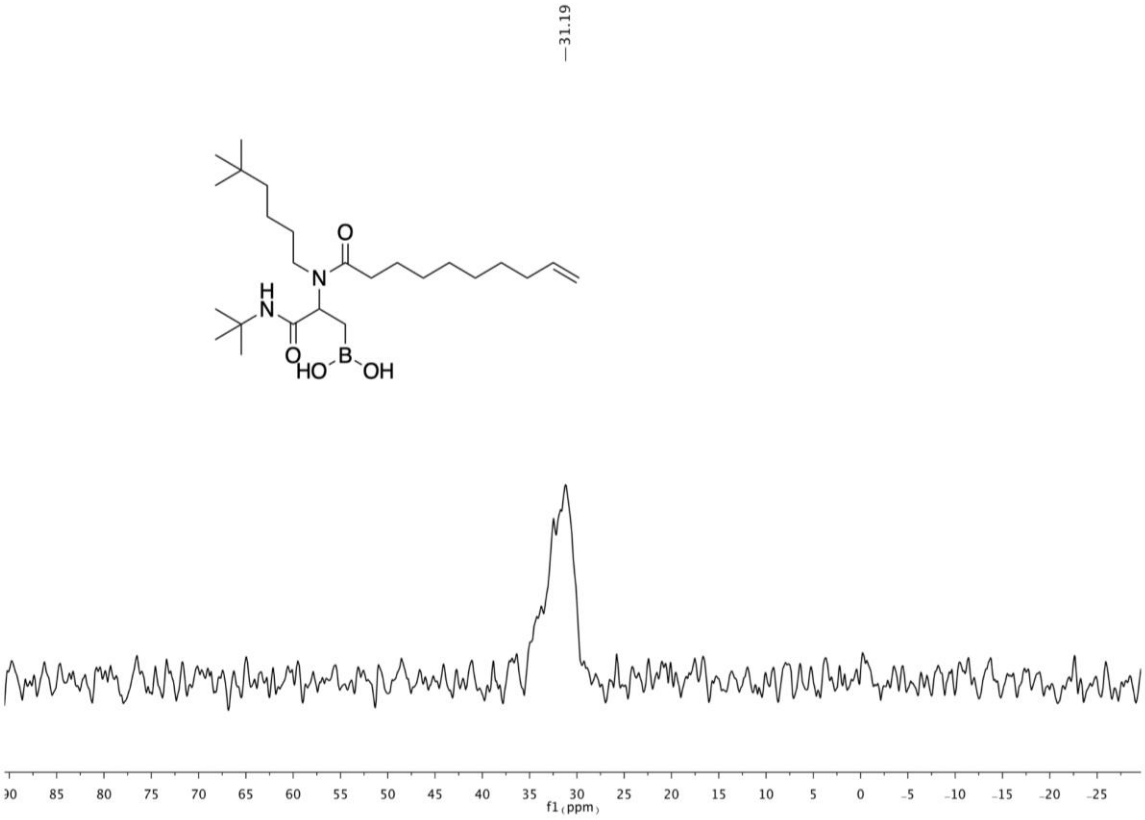

^{11}B NMR (128 MHz, CD_3CN) of compound **6j**

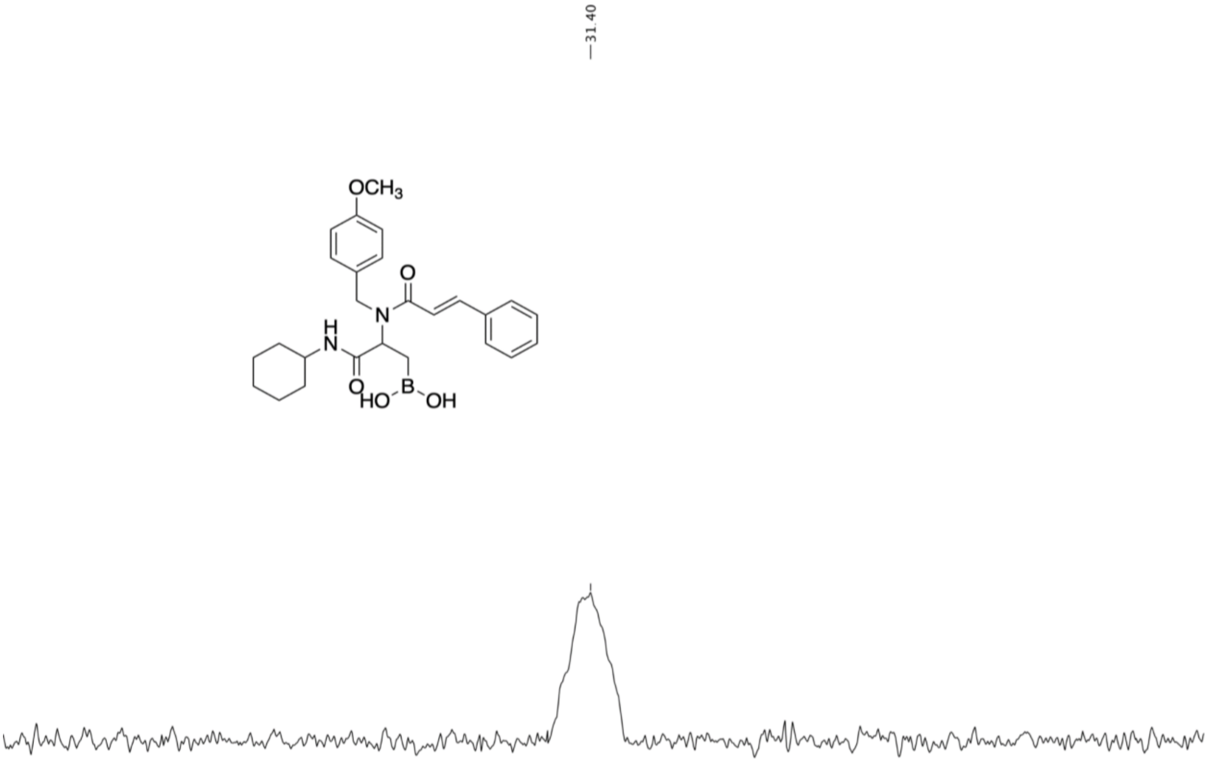

^{11}B NMR (128 MHz, CD_3CN) of compound **6k**

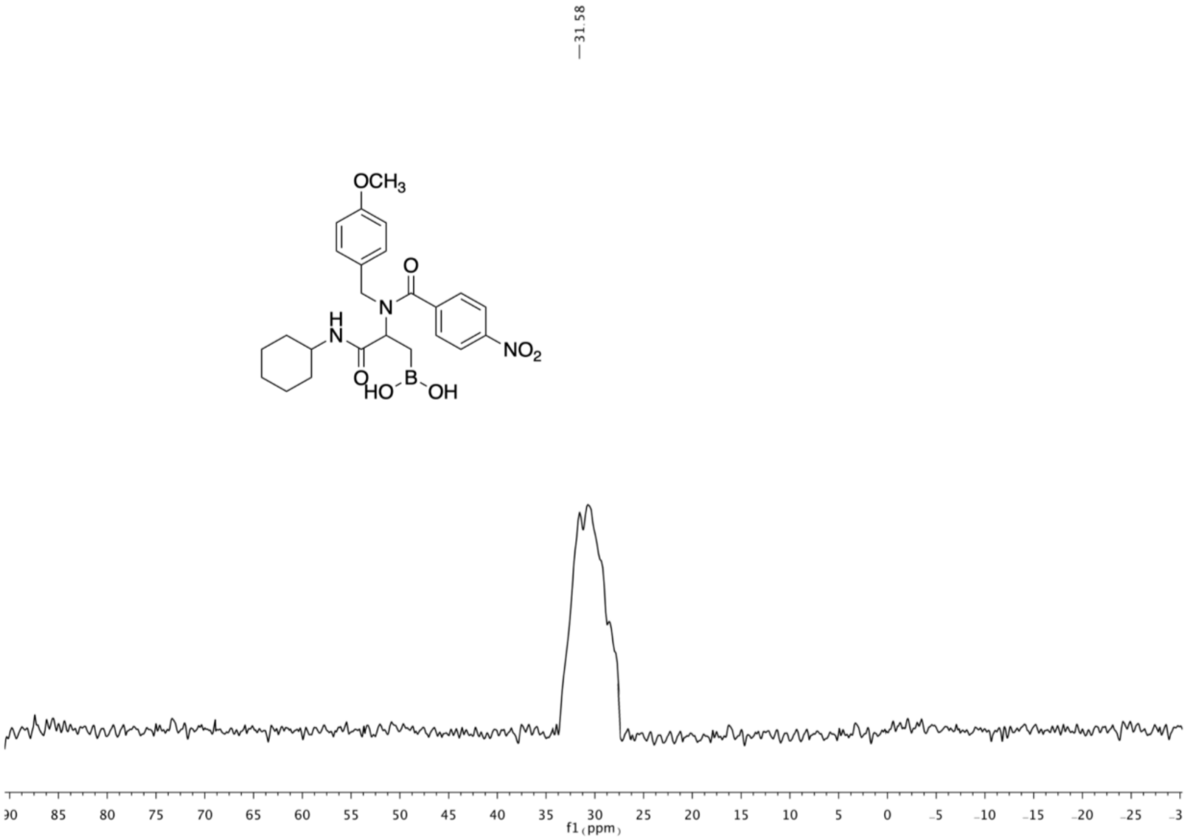

^{11}B NMR (128 MHz, CD_3CN) of compound **6l**

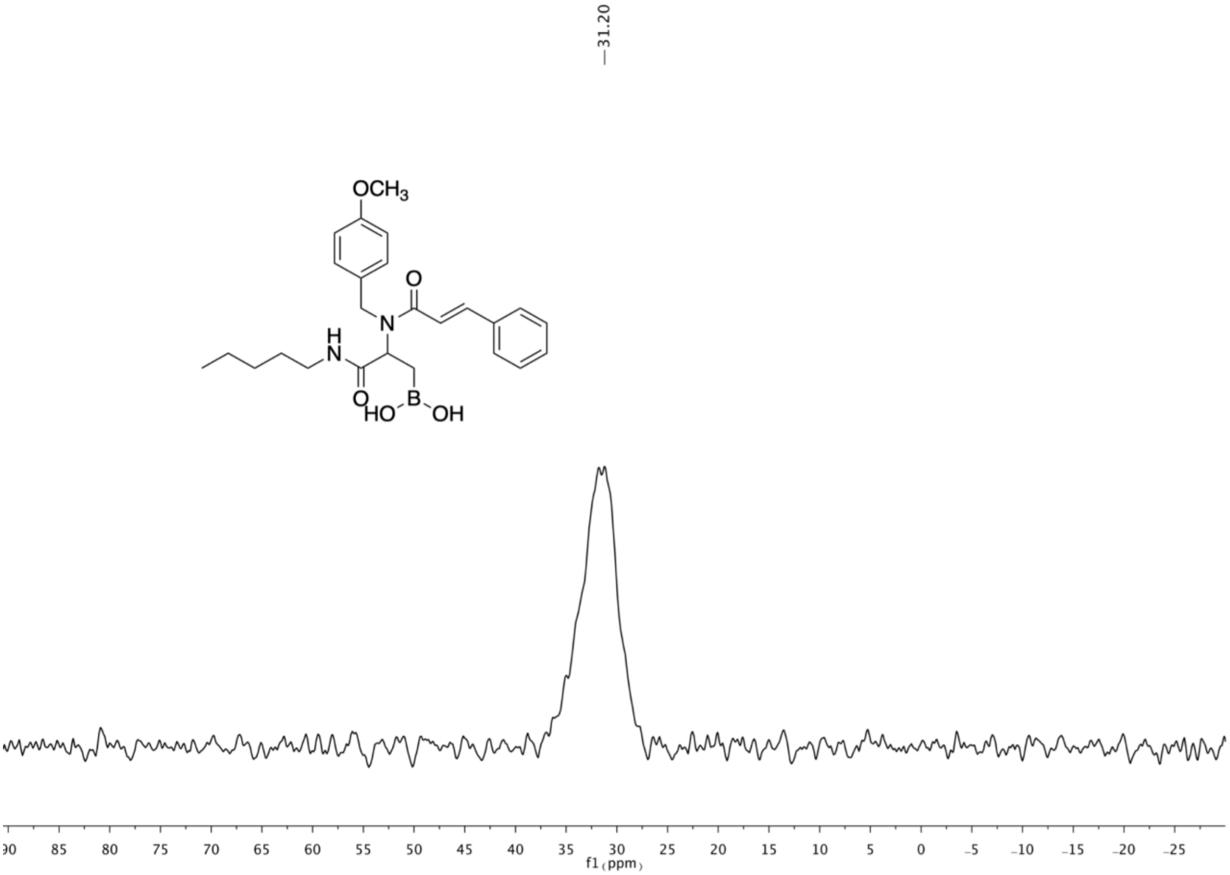

^{11}B NMR (128 MHz, CD_3CN) of compound **6m**

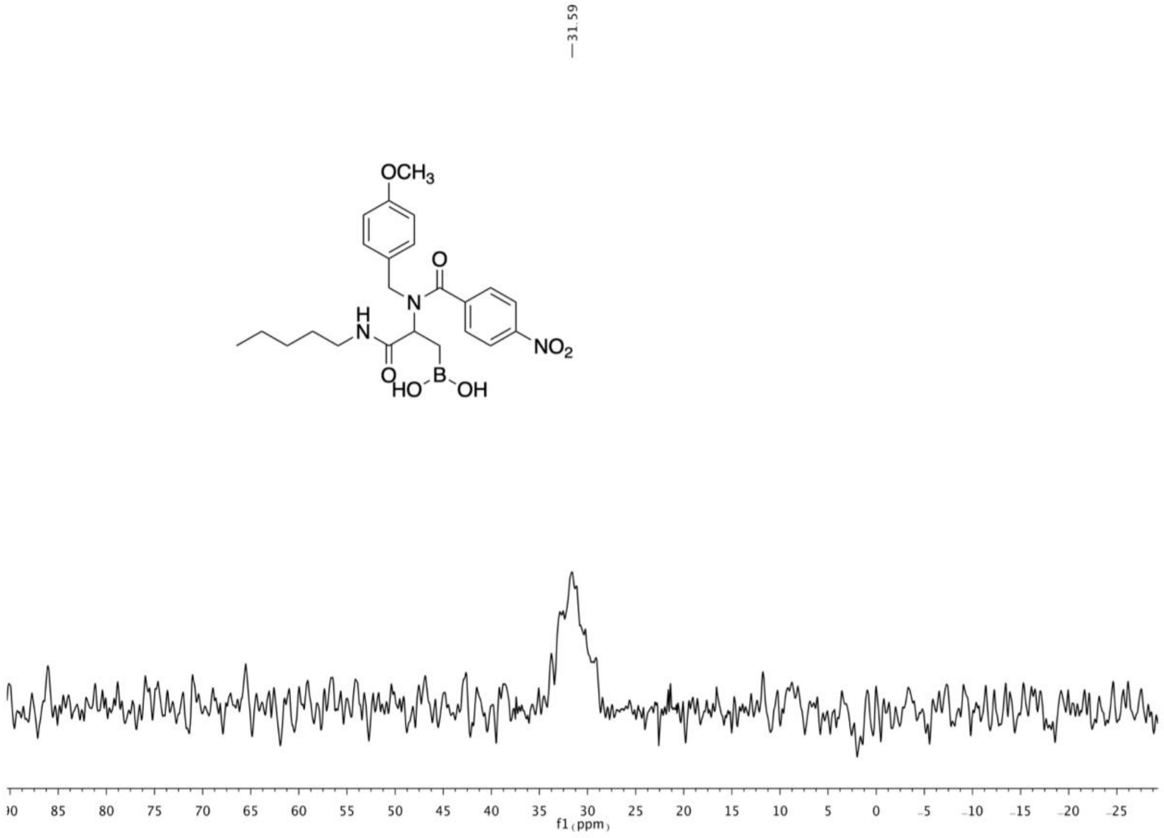

^{11}B NMR (128 MHz, CD_3CN) of compound **6n**

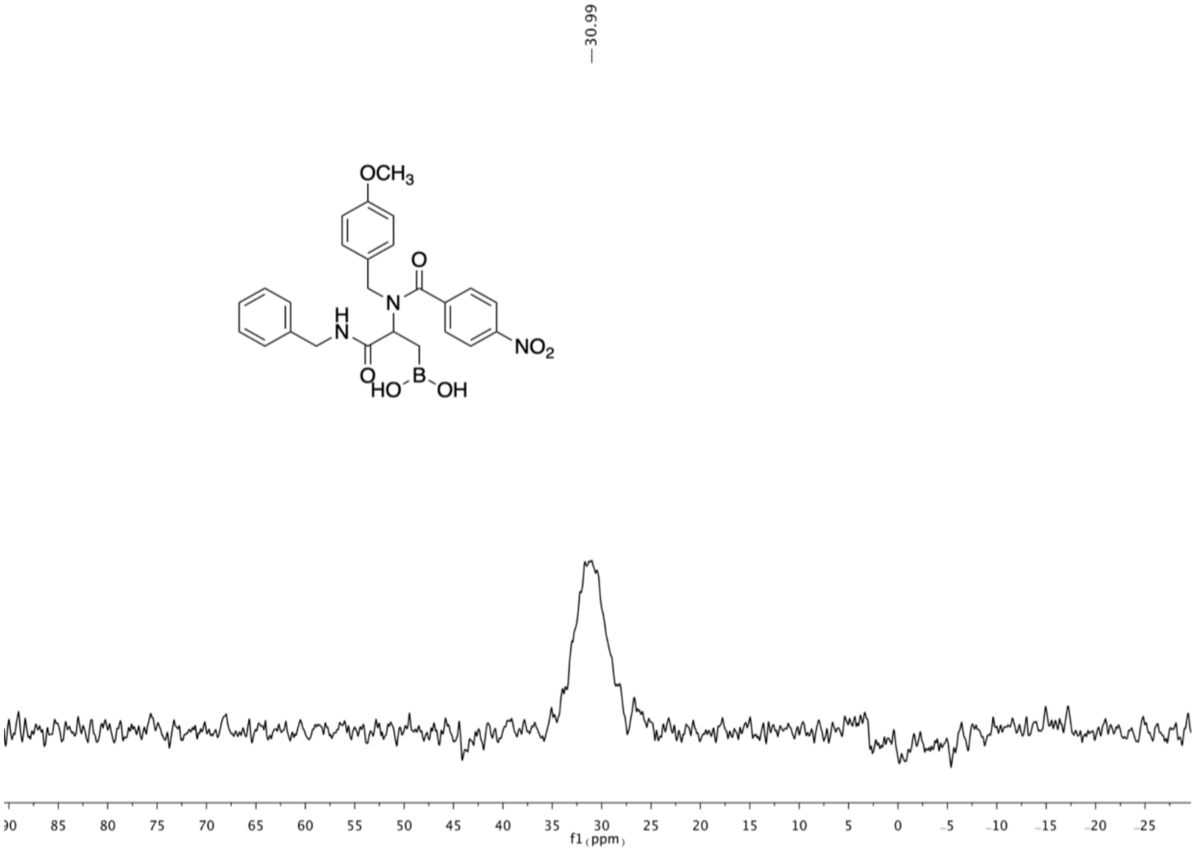

^{11}B NMR (128 MHz, CD_3CN) of compound **6o**

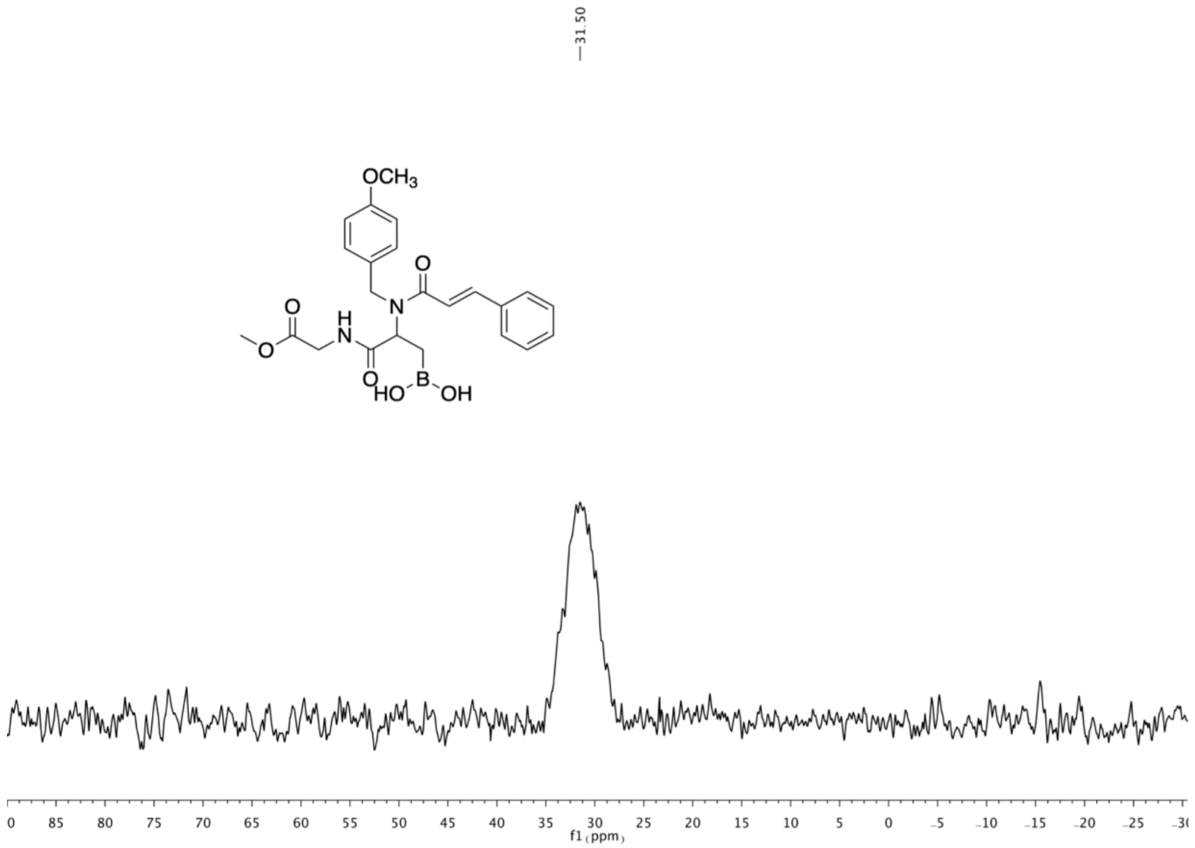

^{11}B NMR (128 MHz, CD_3CN) of compound **6p**

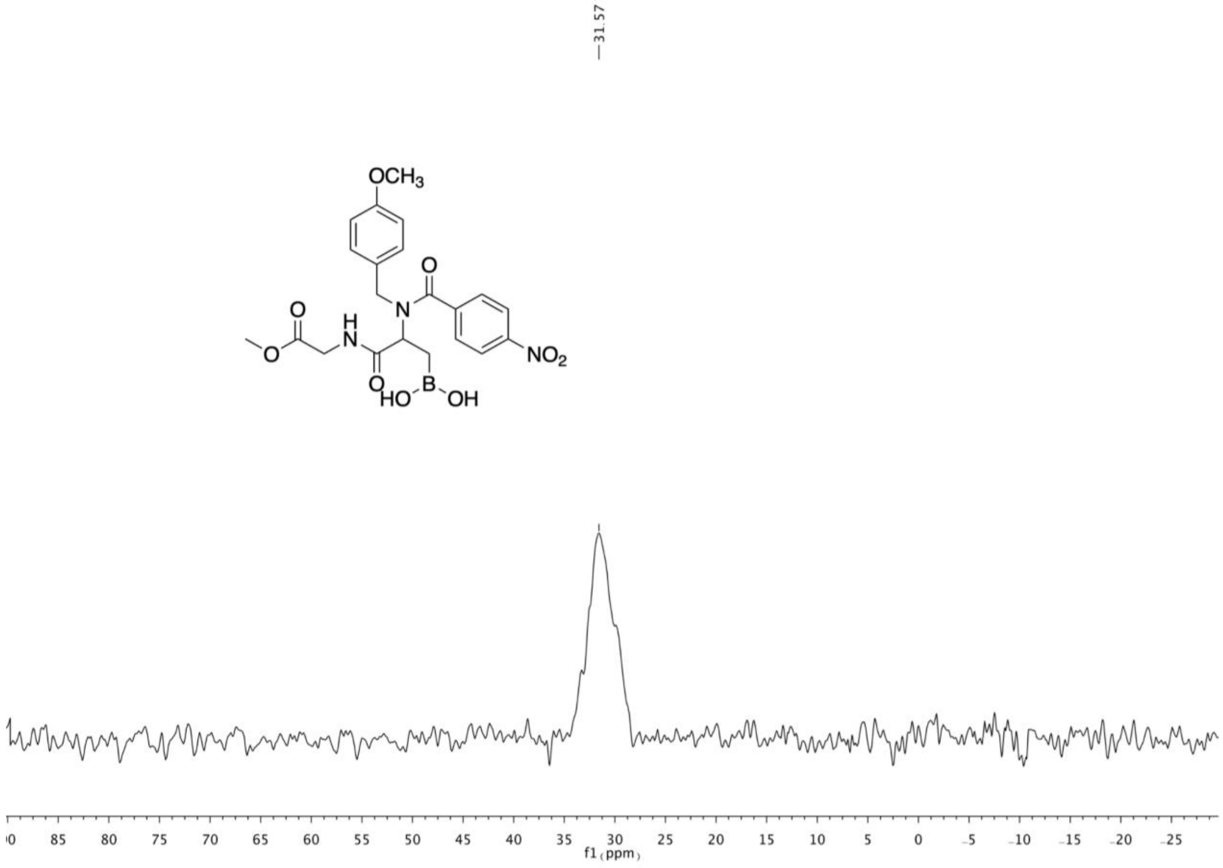

7. ^{11}B NMR spectra of Ugi-4C-3CR products (9a-c)

^{11}B NMR (128 MHz, CD_3OD) of compound **9a**

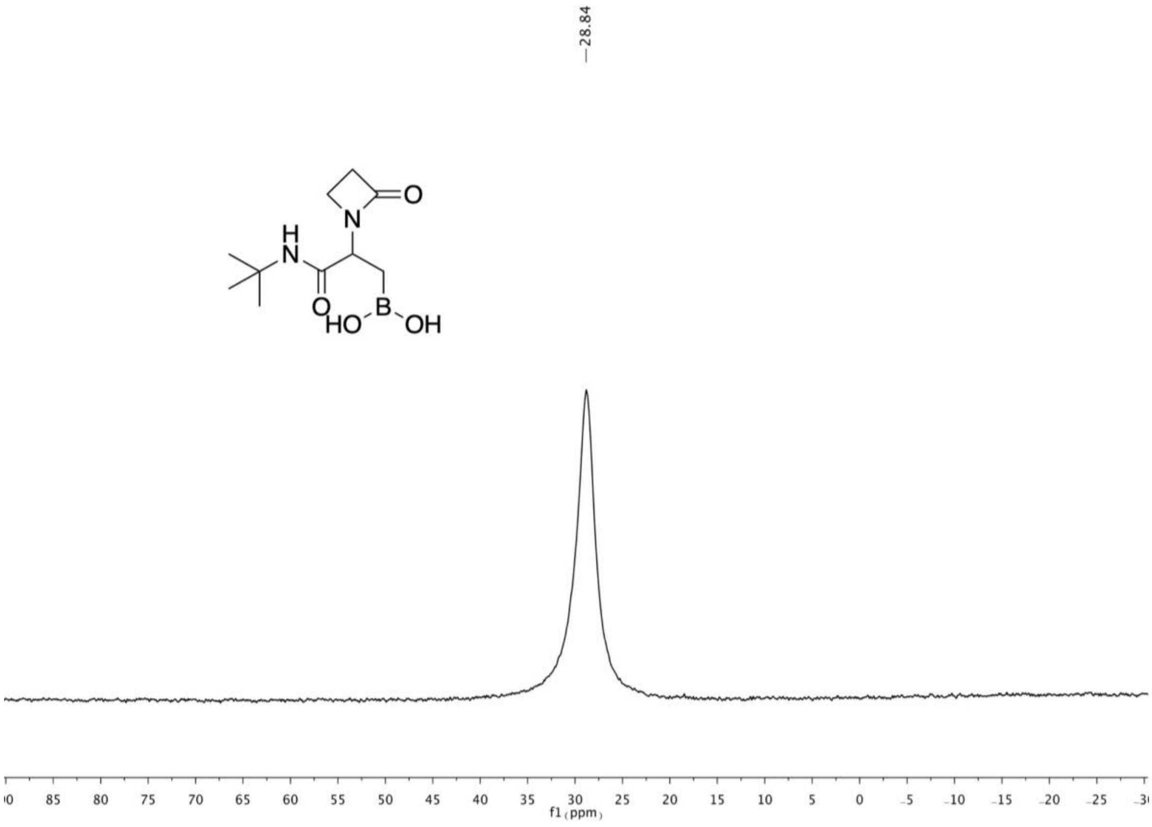

^{11}B NMR (128 MHz, CD_3CN) of compound **9b**

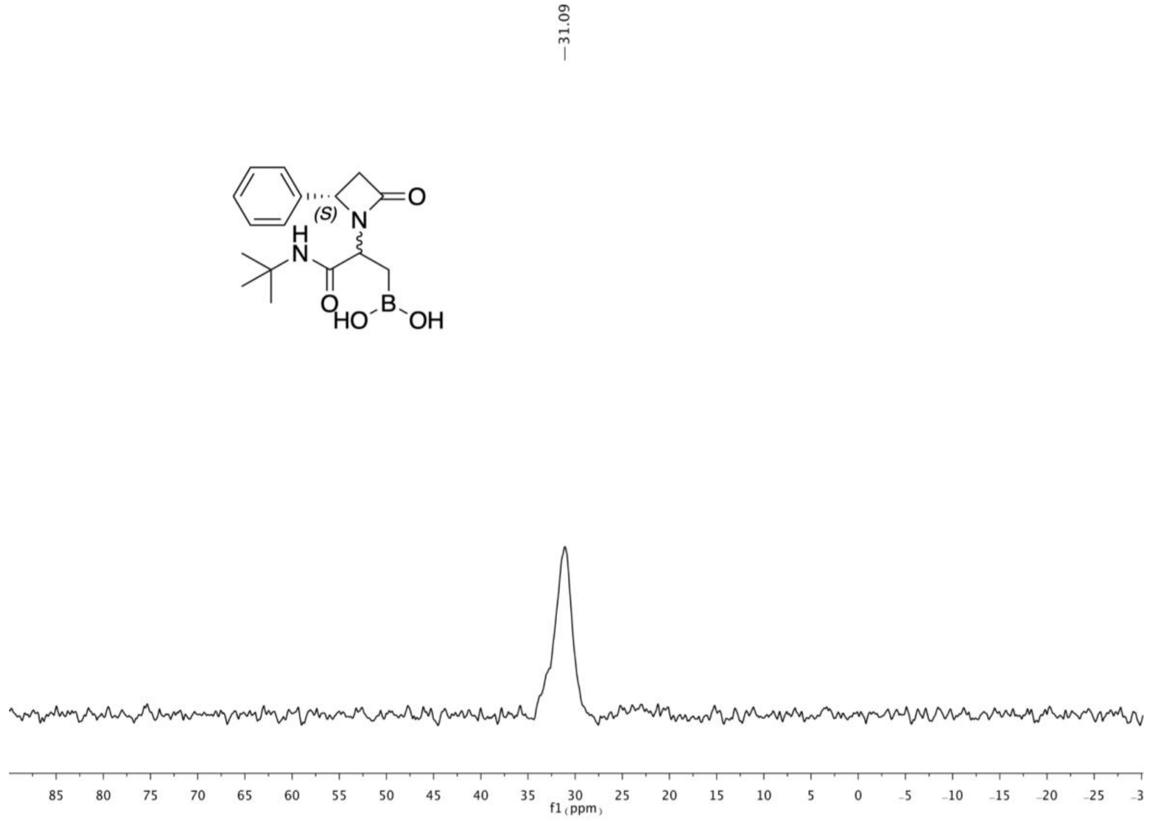

^{11}B NMR (128 MHz, CD_3CN) of compound **9c**

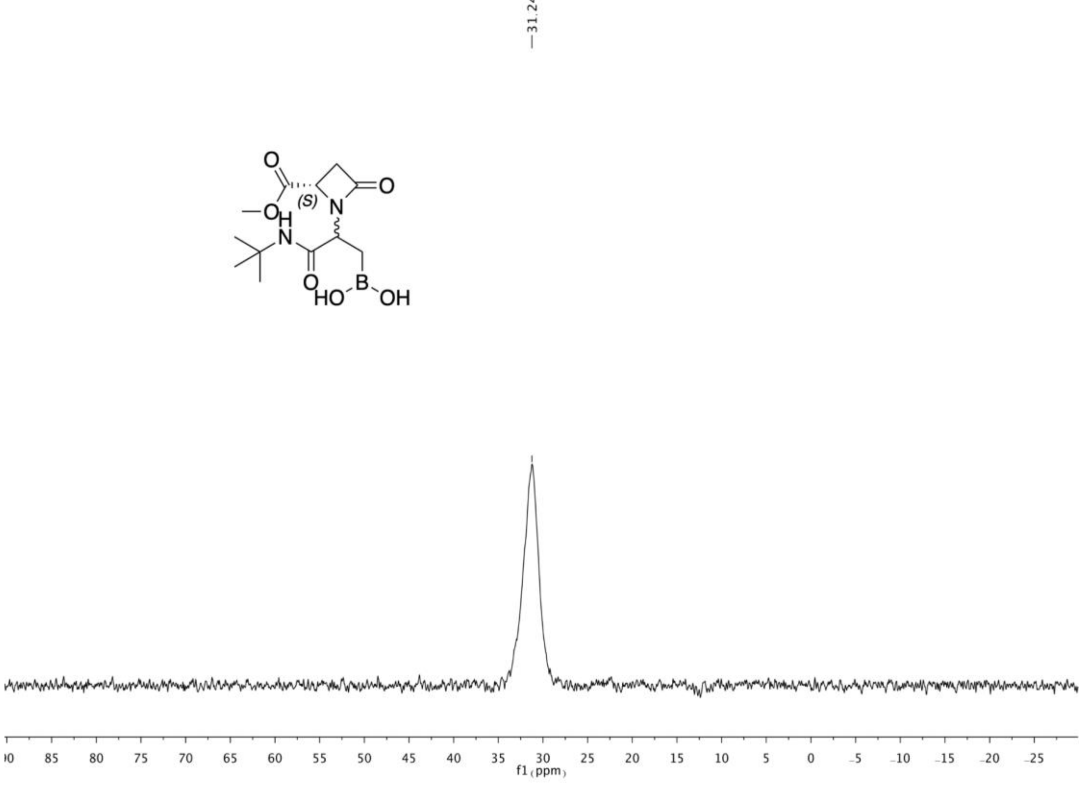

8. Single crystal X-ray diffraction analysis

Compound 5a

Crystalline **5a** was obtained by slow evaporation directly from the mother liquor and used without further crystallization. An X-ray quality single crystal was cut from an agglomerate through a stainless steel micro-blade and polished by mechanical ablation in a perfluorinated oil drop. The sample consists of a colourless transparent needle, with dimensions ≈ 0.175 x 0.100 x 0.050 mm (Figure S1). It shows pleochroism from colourless to green under polarized light and was mounted on the top of a capillary fibre with perfluorinated oil.

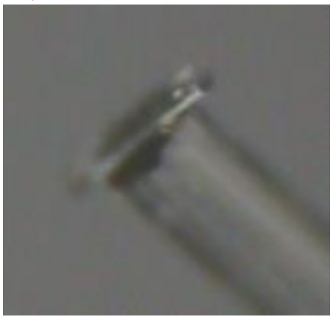

Figure S1. Sample of **5a** employed for the X-ray diffraction analysis, mounted on the top of a capillary fibre.

The X-ray data collection was performed at room temperature with a Bruker AXS Smart 3-circle diffractometer equipped with an APEX-II CCD detector. Graphite–monochromated Mo Kα radiation (λ = 0.71073 Å) at a nominal power of 50 kV x 30 mA of the normal–focus sealed X–ray tube was employed, with the sample held at 50 mm from the source. High–redundant ω–scans ($\Delta\omega$ =0.25 deg) at variable ϕ angles were performed, resulting in 100 % complete spheres of data up to 2θ = 46.5 deg. Data collection and scaling was handled by the SAINT+ software [1]. The final dataset consisted of 31081 measured reflections, corresponding to 4045 symmetry–independent data, of which 2822 had $I > 2\sigma(I)$. Diffraction patterns were corrected by absorption and beam anisotropy using SADABS [2].

The compound crystallizes in the monoclinic centrosymmetric $P2_1/c$ space group with 1 molecule in the asymmetric unit. The unit cell at room temperature is: a = 19.4865(13) Å, b = 13.3604(9) Å, c = 11.2111 (7), β = 105.171(7) deg, V = 2816.9(4) Å^3 as estimated from 1851 intense reflections among 4.9 and 31.3 deg in 2ϑ (final integration result).

The diffraction pattern was phased with direct methods by Shelx[3] and the final least–squares model converged to R1(F) = 0.0391 for 2822 $F_o > 4\sigma(F_o)$, 0.0685 for all the 4045 independent data, with largest Fourier residuals $\Delta\rho_{MAX/MIN}$ = +0.26/ –0.34 e/Å^3.

The final model for molecular structure and packing is reported in Figures S2–S3. CCDC 1995920 contains the supplementary crystallographic data for this paper. These data can be obtained free of charge from The Cambridge Crystallographic Data Centre via www.ccdc.cam.ac.uk/structures.

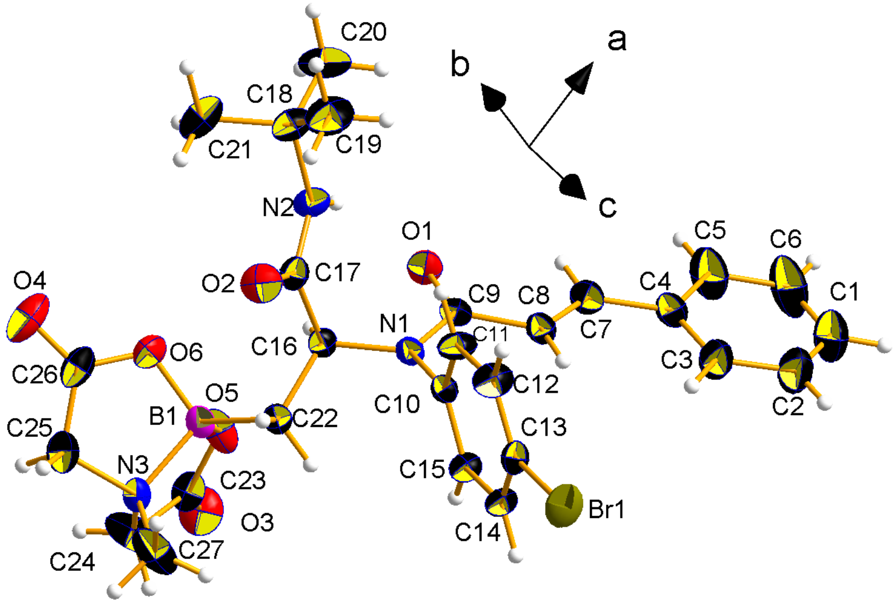

Figure S2. Asymmetric unit of **5a**, with the atom-numbering scheme and crystallographic cell axes. Thermal ellipsoids at RT were drawn at the 30 % probability level.

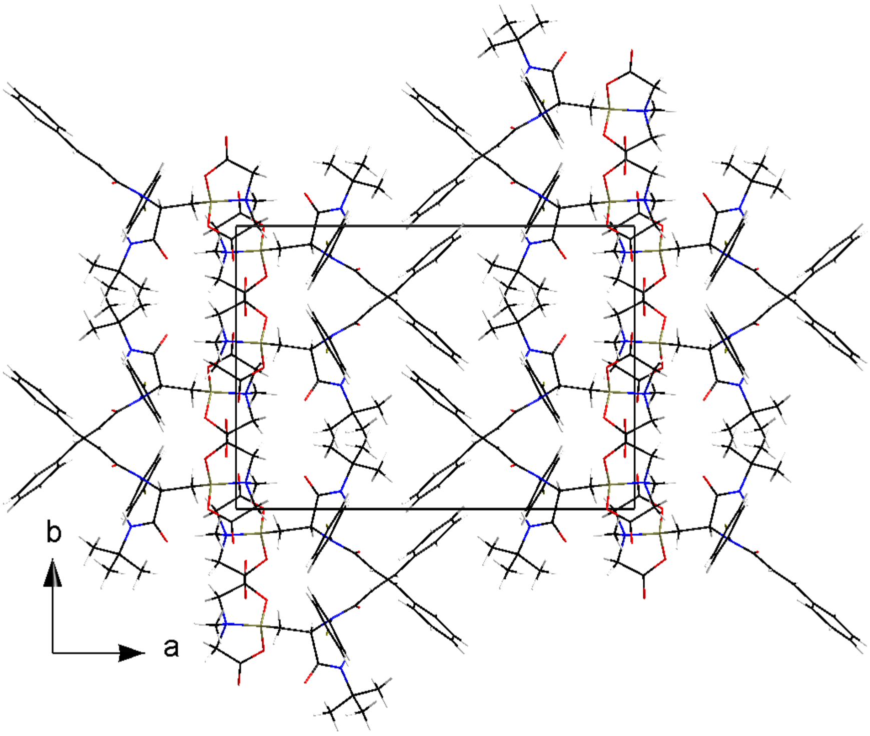

Figure S3. Wires–stick representation of the crystal packing of **5a** at RT, as seen down the *c* cell axis.

5a is chiral and crystallizes in the centric space group $P2_1/c$ as a racemate, with 4 formulae in cell and 1 molecule in the asymmetric unit. Figure S2 shows the configuration of the chiral centre C16 (S). Also the R enantiomer is present in the crystal in a 1:1 ratio.

The N–B dative bond is uncommon, as it is represented only in 8257 deposited structures (0.83 %) within the Cambridge Structural Database v.5.40. In the present structure, $d_{B\text{–}N}$ = 1.646(5) Å. Boron is also connected with two identical oxygen atoms at $d_{B\text{–}O}$ = 1.468(5) Å.

Figure S3 shows the main packing motifs in the (*a*,*b*) plane. **5a** lacks of strong hydrogen bond (HB) donors, apart an amide –NH that is involved in a intramolecular contact with the O1 amide carbonyl (Figure S4, Table S1). Accordingly, the packing is essentially dominated by dispersive–repulsive

balance and electrostatics. The molecule assumes an extended conformation, with cumbersome phenyl rings mutually orthogonal to each other. The latter are thus allocated in the free space along the *a* direction (Figure S3). Stacking interactions are set up in the (*a*,*b*) plane, which form infinite ladders of differently oriented steps along *b*. All the 5-membered rings are almost perfectly flat, with no evident ring puckering.

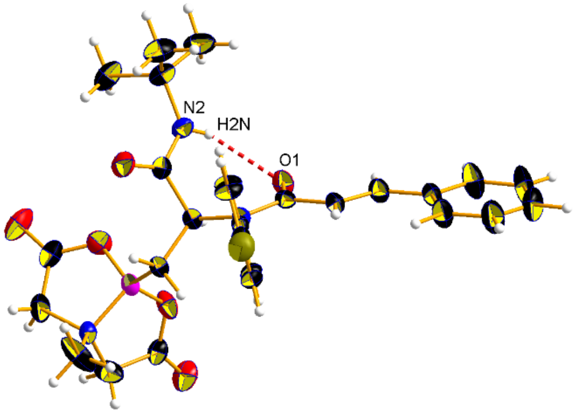

Figure S4. Intramolecular N2–H2N···O1 HB contact. See Table S1 for the geometrical parameters.

Table 1. Geometric parameters for intramolecular hydrogen-bonded contact (Figure S4) in **5a** at room temperature. Values in Å e deg, with estimated standard deviations reported in parentheses.

N-H···O	D-H	H···A	D···A	D-H···A	Symmetry operation
N2–H2N···O1	0.786(4)	2.29(4)	2.981(4)	147(4)	x, y, z (intramolecular)

Compound 6d

Crystalline **6d** was obtained by slow evaporation directly from the mother liquor and used without further crystallization. An X-ray quality single crystal was cut from a large agglomerate through a stainless steel micro-blade and polished by mechanical ablation in a perfluorinated oil drop. The sample consists of a colourless transparent plate, with dimensions ≈ 0.150 x 0.150 x 0.050 mm (Figure S5). It shows pleochroism from colourless to green/red under polarized light and was mounted on the top of a capillary fibre with perfluorinated oil.

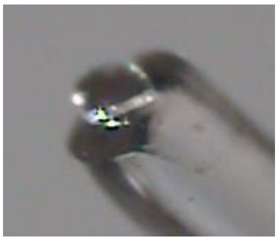

Figure S5. Sample of **6d** employed for the X-ray diffraction analysis, mounted on the top of a capillary fibre.

The X-ray data collection was performed at room temperature with a Bruker AXS Smart 3-circle diffractometer equipped with an APEX-II CCD detector. Graphite–monochromated Mo Kα radiation (λ = 0.71073 Å) at a nominal power of 50 kV x 30 mA of the normal–focus sealed X–ray tube was employed, with the sample held at 50 mm from the source. High–redundant ω–scans ($\Delta\omega$ =0.25 deg) at variable ϕ angles were performed, resulting in 100 % complete spheres of data up to 2θ = 52.7 deg. Data collection and scaling was handled by the SAINT+ software[1]. The final dataset consisted of 16500 measured reflections, corresponding to 4755 symmetry–independent data with R_{int} = 0.0308, of which 2827 had $I > 2\sigma(I)$. Diffraction patterns were corrected by absorption and beam anisotropy using SADABS[2].

The compound crystallizes in the monoclinic centrosymmetric $P\bar{1}$ space group with 1 molecule in the asymmetric unit. The unit cell at room temperature is: a = 9.6147(4) Å, b = 9.6707(4) Å, c = 12.9214(5) Å, α = 83.850(2) deg, β = 78.977(2) deg, γ = 83.615(2) deg, V = 1167.41(8) $Å^3$, as estimated from 2433 intense reflections among 4.9 and 42.3 deg in 2ϑ (final integration result).

The diffraction pattern was phased with direct methods by Shelx[4] and the final least–squares model converged to R1(F) = 0.0392 for 2827 $F_o > 4\sigma(F_o)$, 0.0860 for all the 4755 independent data, with largest Fourier residuals $\Delta\rho_{MAX/MIN}$ = +0.30/ –0.26 e/$Å^3$.

The final model for molecular structure and packing is reported in Figures S6–S7. CCDC 1995919 contains the supplementary crystallographic data for this paper. These data can be obtained free of charge from The Cambridge Crystallographic Data Centre via www.ccdc.cam.ac.uk/structures.

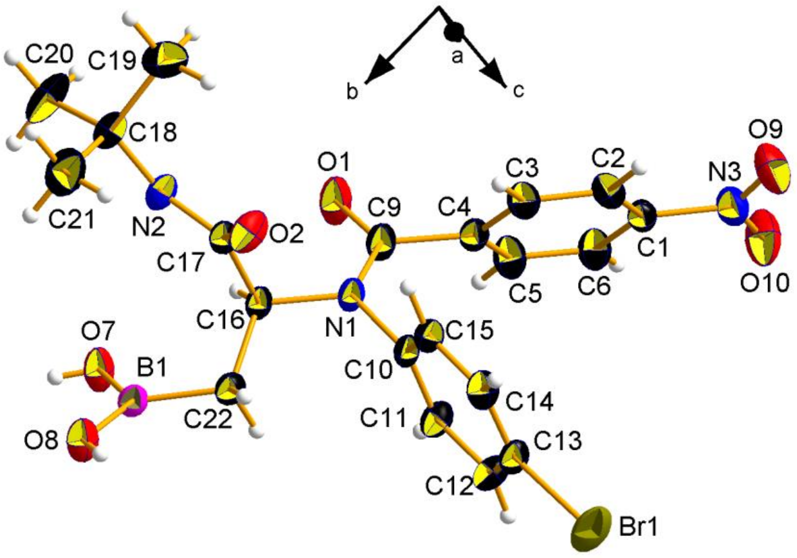

Figure S6. Asymmetric unit of **6d**, with the atom-numbering scheme and crystallographic cell axes. Thermal ellipsoids at RT were drawn at the 30 % probability level.

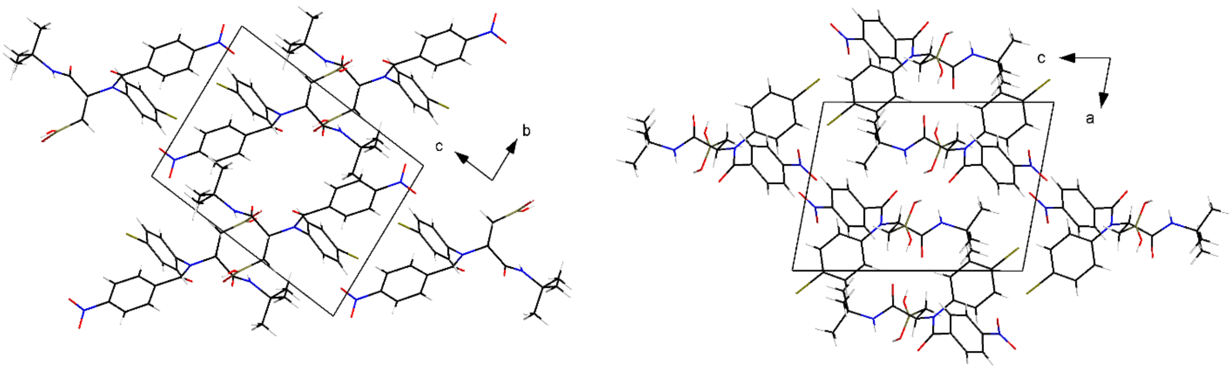

(a) (b)

Figure S7. Wires–stick representation of the crystal packing of **6d** at RT, as seen along the *a* (a) and *b* (b) cell axes.

6d is chiral and crystallizes in the centric space group $P\bar{1}$ as a racemate, with 2 formulae in cell and 1 molecule in the asymmetric unit. Figure S6 shows the configuration of the chiral centre C16 (S). Also the R enantiomer is present in the crystal in a 1:1 ratio.

Figure S7 shows the main packing motifs in the (*b,c*) and (*a*,*c*) planes. At variance with **5a**, **6d** contains strong hydrogen bond donors B–OH, which are all involved in extended networks. Inversion–related molecules form cyclic hydrogen–bonded dimers through symmetric B–OH···O=C interactions that involve the O2 carbonyl (Figure S8, Table S2). Different dimers are connected by somewhat weaker B–OH···O=C HB contacts set up with the carbonyl O1 (Figure S8, Table S2). This latter interaction is assisted by N2–H2N···O7 weaker hydrogen bonds (Table S2). Overall, this results in infinite mono–dimensional ribbons along the *a* axis (see also Figure S7b), which leave a small void volume in the (*b*,*c*) plane (Figure S7a). The geometrical analysis, based on superposition of atomic van der Waals spheres, locates two connected void regions at [x, y, z] = 0.258 0.492 0.543 and [x, y, z] = 0.742 0.508 0.457, each 19(2) Å^3 large (Figure S9). Altogether, they could barely allocate a hydrogen–bonded water molecule (V ~ 40 Å^3), but the Fourier analysis shows that this structure does not reasonably contain any guest molecule. Likely, this small void is made energetically possible thanks to compensation provided by strong H···O interactions.

Table S2. Geometric parameters for intramolecular hydrogen-bonded contact (Figure 4) in BE42 at room temperature. Values in Å and deg, with estimated standard deviations reported in parentheses.

D–H···O	D–H	H···A	D···A	D-H···A	Symmetry operation
N2–H2N···O7	0.74(3)	2.30(3)	2.996(3)	157(3)	−1–x, 2–y, 1–z
O7–H7O···O1	0.71(3)	2.10(3)	2.755(3)	153(3)	−1–x, 2–y, 1–z
O8–H8O···O2	0.67(3)	2.04(3)	2.704(3)	169(3)	−x, 2–y, 1–z (cyclic)

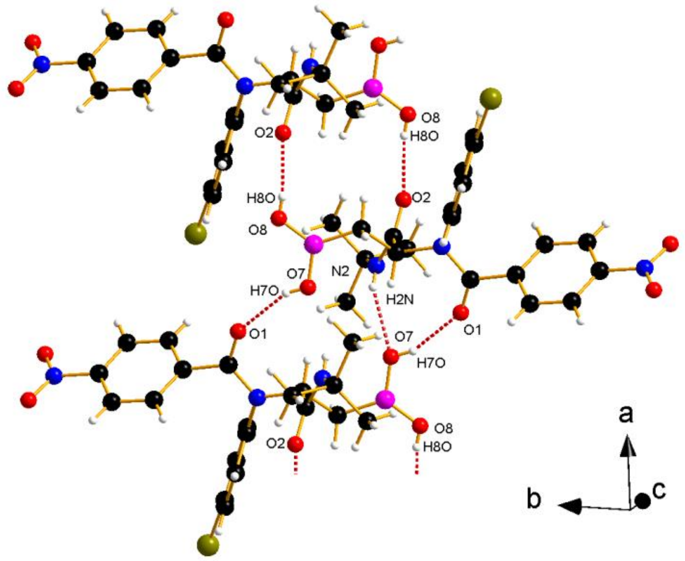

Figure S8. Hydrogen bond patterns of **6d** at room temperature. See Table S2 for the geometrical parameters.

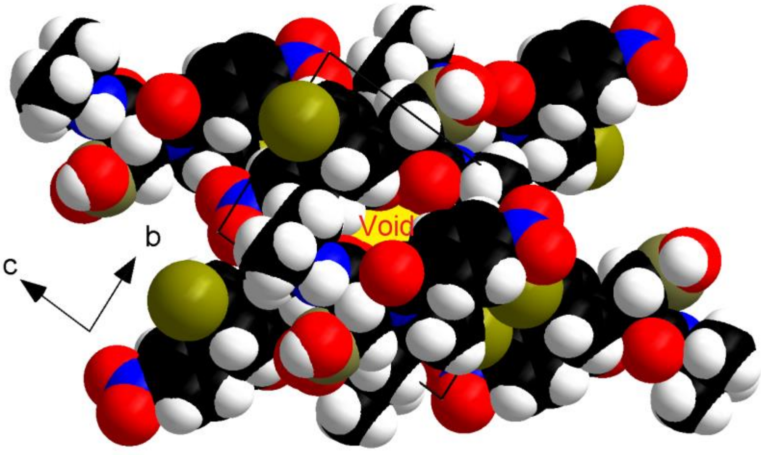

Figure S9. Same as **Figure S7**a, with molecules represented with van der Waals envelopes. The void region approximately at the centre of the (b,c) plane is highlighted in yellow.

References

[1] Bruker (2012). *SAINT+*. Bruker AXS Inc., Madison, Wisconsin, USA.
[2] Bruker (2008). *SADABS*. Bruker AXS Inc., Madison, Wisconsin, USA.
[3] Sheldrick, G. M. Acta Cryst. 2015, C71, 3-8.
[4] Sheldrick, G. M. Acta Cryst. 2015, C71, 3-8.
